# Evolution Analysis of the Fasciclin-Like Arabinogalactan Proteins in Plants Shows Variable Fasciclin-AGP Domain Constitutions

**DOI:** 10.3390/ijms20081945

**Published:** 2019-04-20

**Authors:** Jiadai He, Hua Zhao, Zhilu Cheng, Yuwei Ke, Jiaxi Liu, Haoli Ma

**Affiliations:** 1 College of Agronomy, Northwest A&F University, Xianyang 712100, Shaanxi, China; 2015014922@nwsuaf.edu.cn (J.H.); zhaohua362@nwsuaf.edu.cn (H.Z.); 2015014872@nwsuaf.edu.cn (J.L.); 2 College of Landscape Architecture and Arts, Northwest A&F University, Xianyang 712100, Shaanxi, China; czl980525@163.com; 3 College of Life Sciences, Northwest A&F University, Xianyang 712100, Shaanxi, China; keyuwei98@163.com

**Keywords:** fasciclin-like AGP, FLA, evolution, phylogeny

## Abstract

The fasciclin-like arabinogalactan proteins (FLAs) play important roles in plant development and adaptation to the environment. FLAs contain both fasciclin domains and arabinogalactan protein (AGP) regions, which have been identified in several plants. The evolutionary history of this gene family in plants is still undiscovered. In this study, we identified the *FLA* gene family in 13 plant species covering major lineages of plants using bioinformatics methods. A total of 246 *FLA* genes are identified with gene copy numbers ranging from one (*Chondrus crispus*) to 49 (*Populus trichocarpa*). These FLAs are classified into seven groups, mainly based on the phylogenetic analysis of plant FLAs. All FLAs in land plants contain one or two fasciclin domains, while in algae, several FLAs contain four or six fasciclin domains. It has been proposed that there was a divergence event, represented by the reduced number of fasciclin domains from algae to land plants in evolutionary history. Furthermore, introns in *FLA* genes are lost during plant evolution, especially from green algae to land plants. Moreover, it is found that gene duplication events, including segmental and tandem duplications are essential for the expansion of *FLA* gene families. The duplicated gene pairs in *FLA* gene family mainly evolve under purifying selection. Our findings give insight into the origin and expansion of the *FLA* gene family and help us understand their functions during the process of evolution.

## 1. Introduction

The cell wall plays an important role in plant growth and development by providing structural support and protection, and acting as a filtering mechanism. Although cell wall proteins account for less than 10% of the cell wall mass, they are predominantly involved in the wall structure, support, signaling, and interactions with other wall components and with the plasma membrane [1,2]. Hydroxyproline-rich glycoproteins (HRGPs) are a major group of cell wall glycoproteins that play important roles in plant growth and development [3].

HRGPs are characterized by a protein backbone rich in hydroxyproline (Hyp). The HRGPs superfamily can be divided into three main subfamilies based on the varying degrees of *O*-glycosylation: Arabinogalactan proteins (AGPs), extensins (EXTs), and proline-rich proteins (PRPs) [4,5,6]. The protein backbones of AGPs are rich in hydroxyproline/proline (Hyp/Pro), alanine (Ala), serine (Ser), and threonine (Thr), and these amino acids are regularly arranged as Ala–Pro, Ser–Pro, and Thr–Pro, which were introduced as arabinogalactan (AG) glycomodules [7,8,9]. The carbohydrate side chains of AGPs are attached to Hyp and enriched in arabinose and galactose [10]. Based on the variable protein backbones [6], AGPs can be classified into classical AGPs, chimeric AGPs, and AGP-EXT hybrids. The chimeric AGPs can be further categorized into three subclasses based on different conserved domains: Fasciclin-like AGPs (FLAs) [11,12,13], phytocyanin-like AGPs (PAGs) [14,15], and xylogen-like AGPs (XYLPs) [16,17]. As one subclass of the chimeric AGPs, FLAs consist of both fasciclin domains and AGP regions. In most plant species, FLAs contain one or two fasciclin domains. The fasciclin domains contain two highly conserved motifs (H1 and H2) of about 10 amino acids long each and a conserved central YH motif [18]. Proteins with fasciclin domains were first identified in grasshoppers [19] and as adhesion factors were first identified in fruit flies [20]. Since then, more and more fasciclin domains have been identified in animal, yeast, bacteria and plant proteins [18]. The majority of plant fasciclin-like proteins are FLAs and the functions of FLAs are related to many important processes in development and stress responses, such as contributing to biophysical properties (e.g., swelling and interpolymer connectivity), affecting secondary cell wall formation and structure, acting in male gametophyte development, influencing organ formation, and sensing salt stress in roots [18].

To date, FLAs have been identified in several plants, including *Arabidopsis* (*Arabidopsis thaliana*) [21], rice (*Oryza sativa*) [12,22], wheat (*Triticum aestivum*) [22], poplar (*Populus trichocarpa*) [23,24], zinnia (*Zinnia elegans*) [25], cotton (*Gossypium raimondii*) [26], sea island cotton (*Gossypium barbadense*) [27], Chinese cabbage (*Brassica rapa*) [28], eucalyptus (*Eucalyptus grandis*) [13], and textile hemp (*Cannabis sativa*) [29]. The analysis of HRGPs from 1000 plant transcriptomes has provided new insights into the evolution of HRGPs across major evolutionary milestones and reveals the origin and diversity of Glycosylphosphatidylinositol (GPI)-anchored AGPs [3]. However, the evolutionary history of the FLA family in plants is little known. In a previous study, it was proposed that a conserved group of FLAs with a single fasciclin domain was specific to the evolution of flowering plant secondary cell wall formation and properties through phylogenetic analysis of >100 FLA mature proteins [30]. In this study, we identify 246 FLAs from 13 plant species belonging to algae, liverworts, mosses, lycophytes, gymnosperms, dicots, and monocots. Moreover, bioinformatics methods are adopted to reveal the evolutionary mechanisms of the FLA family. In order to understand the functions of the FLAs, the evolutionary history of FLAs is investigated in this study. It is found that the *FLA* genes are abundant in most investigated green plants, but only in one red alga. Additionally, our study shows that there is a reduction in the number of fasciclin domains in FLAs from algae to land plants, which indicates that the reduced number of fasciclin domains plays a crucial role in land plant evolution.

## 2. Results and Discussions

### 2.1. Identification of the FLA Family in Plants

FLAs contain both fasciclin domains and AGP regions [6]. We first used the HMM profile of fasciclin downloaded from Pfam (available online: http://pfam.xfam.org/family/PF02469) to identify the proteins with fasciclin domains from 13 plant species (*C. crispus, Chlamydomonas reinhardtii*, *Chara braunii*, *Marchantia polymorpha, Physcomitrella patens*, *Selaginella moellendorffii*, *Picea abies*, *Amborella trichopoda*, *Brachypodium distachyon*, *O. sativa*, *A. thaliana*, *E. grandis*, and *P. trichocarpa*) [31,32,33,34,35,36,37,38,39,40,41,42,43]. Then, the obtained proteins were examined by using Batch CD-search tool in the NCBI conserved domain database (available online: http://www.ncbi.nlm.nih.gov/Structure/bwrpsb/bwrpsb.cgi). After that, the AGP regions were identified from these fasciclin proteins by using Finding-AGP program [7]. The proteins that contained both AGP regions and fasciclin domains were identified as FLAs. A total of 235 *FLA* genes were identified by the HMMER-Finding-AGP program method. 

However, the number of *FLA* genes found in some plants was different from those described in former studies. In *A. thaliana*, *FLA20* (*AT5G40940*) and *FLA21* (*AT5G06920*) [21] were not identified, while a new putative *FLA* gene, *AT5G16920*, was identified. In *E. grandis*, *Eucgr.A01741* and *Eucgr.K02662* were missing [13], and *Eucgr.K00086* was a newly identified *FLA* gene. In *P. trichopoda*, 46 *FLA* genes were identified compared with the 50 *FLA* genes analyzed in a previous study [24]: *Potri.013G152200*, *Potri.T130300*, *Potri.001G440800*, *Potri.018G005100*, *Potri.008G127500*, *Potri.008G128200*, and *Potri.005G079500* were not identified, whereas *Potri.019G049600*, *Potri.T118500* and *Potri.012G006200* were new putative *FLA* genes identified in this study. In *O. sativa*, two *FLA* genes found in a previous study (*LOC_Os02g49420* and *LOC_Os02g26290*) [12] were not identified, while a putative new *FLA* gene (*LOC_Os12g13160*) was identified in our work. Among 13 *FLA* genes that were not identified by the HMMER-Finding-AGP program method, it was found that Potri.T130300, Potri.018G005100, LOC_Os02g49420, and LOC_Os02g26290 did not contain a fasciclin domain by using Batch CD-Search tool. Besides, because the AGP regions of Eucgr.K02662, Potri.008G127500, and Potri.008G128200 were found in the fasciclin domain, they were not identified as FLAs in this study. Then, the remaining six FLAs (AT5G40940, AT5G06920, Eucgr.A01741, Potri.013G152200, Potri.001G440800, and Potri.005G079500) were included in this study and also used as queries to perform BLAST searches to identify their homologous FLAs in other plant species: Phpat.003G041000 in *P. patens*, MA_89859g0010 and MA_10360g0010 in *P. abies*, scaffold00024.69 in *A. trichopoda*, and Eucgr.H00590.1 in *E. grandis*. As a result, 246 *FLA* genes were identified. 

The number of *FLA* genes ranged from 1 to 49 across the different plant species; in most species, the number of *FLA* genes was between 11 and 26. *C. crispus* had only one *FLA* gene, while *P. trichocarpa* contained the highest number of *FLA* genes (49), almost double the number of the second one, *O. sativa* (26). It was found that the number of *FLA* genes and genome size were uncorrelated. *P. abies*, for instance, which had the largest genome size (19,600 Mb) among these 13 plant species, had only 24 *FLA* genes compared with *P. trichocarpa* which had 49 *FLA* genes with a much smaller genome size (434.29 Mb) (Table 1). The number of *FLA* genes was also uncorrelated with the number of predicted genes in plant species. For example, *E. grandis* contained more genes (45,226) than *O. sativa*, while *O. sativa* had more *FLA* genes (26) than *E. grandis* (18) (Table 1). Overall, higher plants contained the highest number of *FLA* genes and the number of *FLA* genes increased from lower plants to higher plants. For example, the number of *FLA* genes was doubled from lycophytes to gymnosperm.

Moreover, the intron-exon structures of 246 *FLA* genes were retrieved from the OrcAE website (available online: https://bioinformatics.psb.ugent.be/orcae/overview/Chbra), Phytozome website (Version 12; available online: https://phytozome.jgi.doe.gov/pz/portal.html), and ConGenIE website (available online: http://congenie.org/) and were displayed by GSDS 2.0 (available online: http://gsds.cbi.pku.edu.cn/) [44]. Green algae *FLA* genes contained a large number of introns, while most land plants *FLA* genes contained one intron or even had no intron (Table S1). It seemed that introns in FLA genes were lost during plant evolution, especially from green algae to land plants.

### 2.2. Phylogenetic Analysis and Classification of FLAs

**Figure 1:**
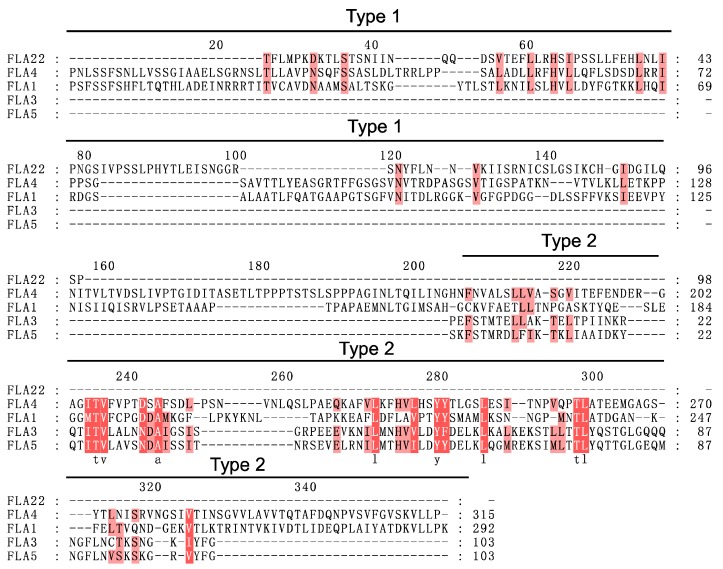
Multiple sequence alignment of representative FLA sequences. Fasciclin domains were divided into two types (Type 1 and Type 2). Residues with high similarity (80%, 60%) were highlighted in dark pink and light pink, respectively.

In order to understand the relationships between FLAs with different numbers of fasciclin domains, evolutionary analysis was performed based on multiple sequence alignments of FLAs. First, all the FLA protein sequences were filtered by BLAST+ [45] with a −5 expect (E) threshold. The sequences (CreFLA2, CreFLA3, CreFLA4, CreFLA5, CreFLA6, and CreFLA7 in *C. reinhardtii*, CbrFLA5, CbrFLA6, CbrFLA8, CbrFLA10, CbrFLA12, CbrFLA13, CbrFLA14, CbrFLA17, CbrFLA18, and CbrFLA21 in *C. braunii*) with low similarity to other plant species were removed, and classified into Group F (Table S1). Next, after removing sequences of signal peptides and GPI anchor addition signals, the filtered 230 FLA sequences were aligned by Clustal Omega 1.2.2, and the HMM profile of fasciclin domains was used as a guide [46,47]. Then, the fasciclin domains could be divided into two types (Type 1 and Type 2) based alignment results (Figure 1 and Figure S1). The FLA sequences with Type 1 and Type 2 fasciclin domains were further aligned, respectively (Figures S2 and S3). Interestingly, for some algae FLA sequences that contained more than two fasciclin domains, only one or two fasciclin domains had hits in other FLA sequences: The first and the fourth fasciclin domains in CreFLA11, the second fasciclin domain in CreFLA10. It was likely that the other fasciclin domains with low similarity to those in higher plants were lost in the course of evolution from algae to land plants.

**Figure 2:**
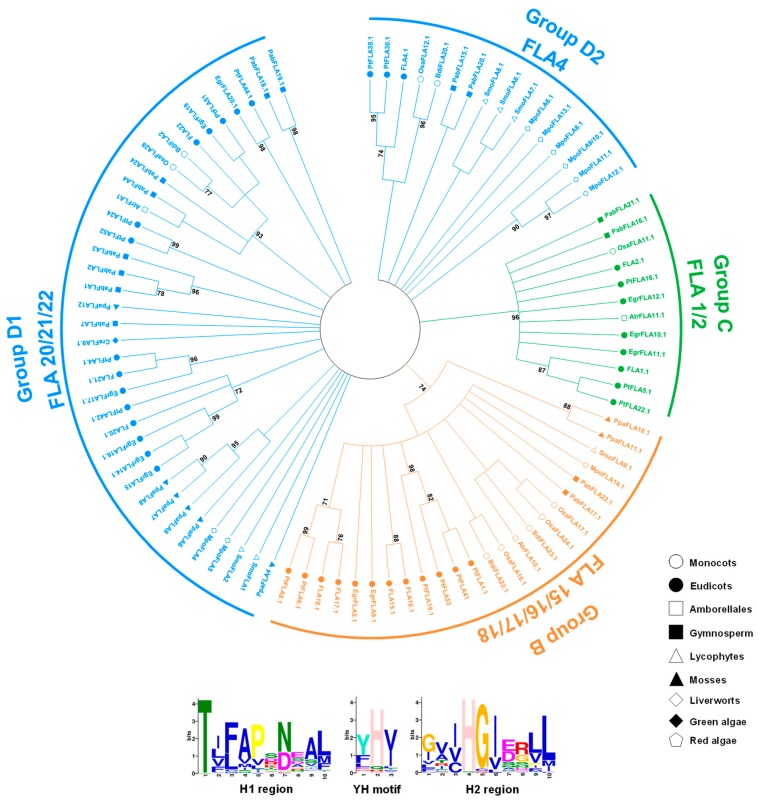
Phylogenetic relationships between Type 1 fasciclin domains in plant species. The amino acid sequences of fasciclin domains in FLAs were aligned by Clustal Omega 1.2.2 with the guide of HMM profile of fasciclin domains, and the phylogenetic trees were built by Mega 7 using the Maximum Likelihood (ML) method with 85% partial deletion. Bootstrap analyses with 1,000 replicates were performed for support estimation. The confidence values below 50% were cut off, and the confidence values higher than 70% are shown on nodes. The tree was divided into four major clades: Group B, Group C, Group D1, and Group D2. Plant species from different lineages are shown in different shape. FLAs from *A. thaliana* are indicated for each clade. The order of fasciclin domains was designated from the N-terminus to the C-teminus (e.g., FLA4.1, FLA4.2, and so on). The conserved motifs (H1, H2, and YH motifs) shown below the tree were found using the MEME web server.

**Figure 3:**
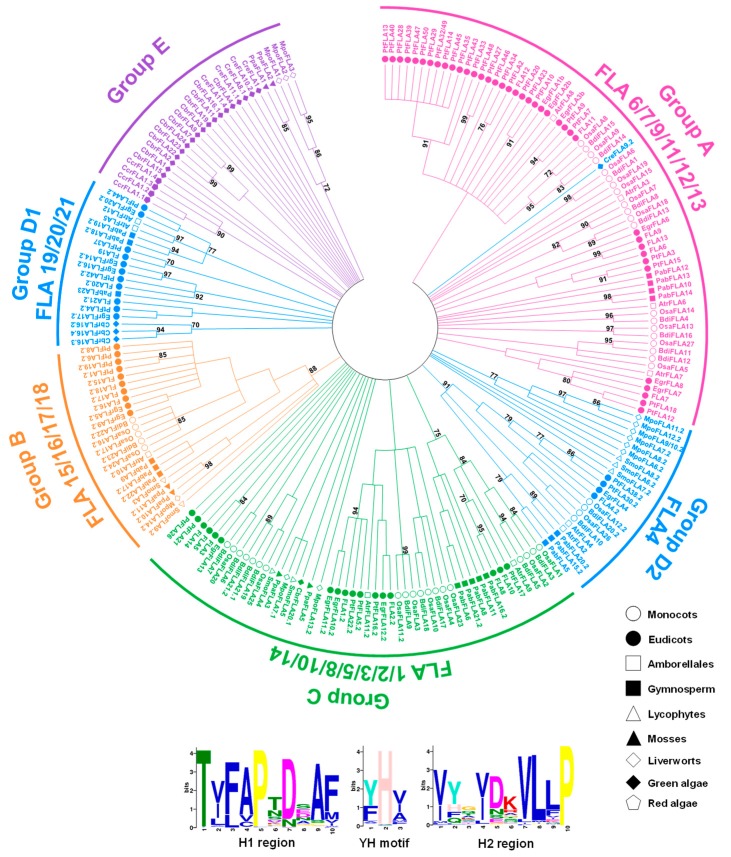
Phylogenetic relationships between Type 2 fasciclin domains in plant species. The amino acid sequences of fasciclin domains in FLAs were aligned by Clustal Omega 1.2.2 with the guide of HMM profile of fasciclin domains, and the phylogenetic trees were built by Mega 7 using the Maximum Likelihood (ML) method with 85% partial deletion. Bootstrap analyses with 1000 replicates were performed for support estimation. The confidence values below 50% were cut off, and the confidence values higher than 70% are shown on nodes. The tree was divided into six major clades: Group A, Group B, Group C, Group D1, Group D2, and Group E. Plant species from different lineages are shown in different shape. FLAs from *A. thaliana* are indicated for each clade. The domain closest to the N-terminus is indicated by .1 and the second by .2. The conserved motifs (H1, H2, and YH motifs) shown below the tree were found using the MEME web server.

The phylogenetic tree of filtered 230 FLA sequences could not be built because the identity of alignment was very low (<30%). Once the identity was above 30%, the accuracy of alignment was acceptable [48,49,50]. The accuracy of the FLA alignment results was tested by computing the overall mean distance with the P-distance method in Mega 7 [49,51]. As P-distance equals 1 minus the identity of amino acids, the identities of Type 1 and Type 2 fasciclin domains were 31.7% and 30.4%, respectively. The accuracy results of Type 1 and Type 2 were 0.683 and 0.696, respectively. These indicators made it suitable for building the phylogenetic trees. The Maximum Likelihood (ML) trees for each type were built using the best models: Le_Gascuel_2008 model [52] + Gamma distribution + evolutionarily invariable (LG + G + I) for Type 1, Le_Gascuel_2008 model + Gamma distribution (LG + G) for Type 2, with 85% partial deletion by Mega 7. Bootstrap analyses with 1000 replicates were performed for support estimation. Confidence values below 50% were cut off, and confidence values higher than 70% were shown on nodes (Figure 2 and Figure 3). Although the similarity between full-length sequences of FLAs are quite low, the fasciclin domains exhibited two highly conserved motifs (H1 and H2) and a conserved central YH motif [18]. MEME web server (available online: http://meme-suite.org/tools/meme) [53] was used to find the conserved motif (H1, H2, and YH motifs) of Type 1 and Type 2 sequences. The H1 and YH motif were similar between Type 1 and Type 2 sequences, while the H2 region was quite different. In Type 1 sequences, the H2 motif was characterized by [Gly/Ile/Val/Leu/Phe]–X–[Ile/Val/Cys]–His–Gly–[Ile/Val/Leu]–X–X– [Leu/Val/Pro/Ile]–[Leu/Met/Ile] sequence. In Type 2 sequences, the H2 motif was characterized by [Val/Ile/Met/Leu]–[Tyr/His/Phe/Gln]–X–[Val/Ile/Leu]–X–X–[Val/Leu]–Leu–[Leu/Phe/Val]–Pro sequence (X represents any amino acid) (Figure 2 and Figure 3). Interestingly, most FLAs with single fasciclin domain was of Type 2, while only a few FLAs with single fasciclin domain was of Type 1.

Based on the sequence similarity, phylogenetic analysis, and previous study [11], we have classified FLAs into seven groups: Group A (including FLA6, FLA7, FLA 9, FLA 11–13 from *A. thaliana*), Group B (FLA 15–18 from *A. thaliana*), Group C (including FLA 1–3, FLA 5, FLA8, FLA10, FLA14 from *A. thaliana*), Group D1 (including FLA 19–22 from *A. thaliana*), Group D2 (including FLA4 from *A. thaliana*), Group E, and Group F (Table S1). Group F sequences were all algae FLAs which were not included in building phylogenetic trees. The remaining algae FLAs were all in Group D1 and Group E, which meant that Group D1 and Group E might be traced back to the origin of the FLA family in plants. Moreover, *FLA3*, *5*, *14*, *20*, *21*, and *22* were specifically expressed in anthers at different stages of floral development [18,54,55]. *FLA3* was involved in microspore development, and its knock-down plants showed reduced female fertility [56]. There was a probability that Group C and Group D1 FLAs were mainly related to male gametophyte development. Group C and Group D1 FLAs were also related to the growth regulator. For instance, FLA1 and FLA2 might play an important role in root development [57,58]. Interestingly, in Group A, all FLAs were with single fasciclin domain. A previous study proposed that Group A FLAs were specific to the evolution of flowering plant secondary cell wall formation and properties [30]. For example, *FLA11*, *FLA12*, and *ZeFLA11* are highly expressed in vascular tissue and double mutants of *FLA11* and *12* showed defects in secondary cell wall thickening [25,30]. *EgrFLA1*, *2*, and *3* were also highly expressed in stems. EgrFLA2 was involved in altering fiber cellulose deposition in woody tissue, and EgrFLA3 influenced flexural strength [13]. In *Eucalyptus nitens*, EniFLA1, 2, and 3, which were closely related to FLA11 and 12, as well as highly similar to EgrFLA1 and 2, could affect stem biomechanics [30]. These Group A FLAs and their homologs in other plants (poplar, zinnia) were also involved in secondary cell wall biosynthesis [23,25]. In addition, FLA9 in Group A was also related to seed development. It had been shown that the stress-induced reductions of *FLA9* gene expression enhanced the abortion of fertilized ovaries [59].

In addition, the variable fasciclin number of FLAs had a tight relationship to the phylogenetic tree. All the FLAs with multiple fasciclin domains (>2) were in Group D1 and Group E. As these FLAs were only identified in algae, they might be the most original FLAs in the course of evolutionary history. In Group A, all the FLAs were with single fasciclin domain and belonged to seed plants. Group A FLAs were the latest FLAs generated in the course of evolutionary history. From Group E to Group A, the number of fasciclin domains reduced over the course of evolutionary history. Except for Group A FLAs, the structures of FLAs were quite diverse, especially for Group E FLAs, which included the most original FLAs. Moreover, Group E *FLA* genes contained more introns than other groups. The number of introns also reduced over the course of evolutionary history.

**Figure 4:**
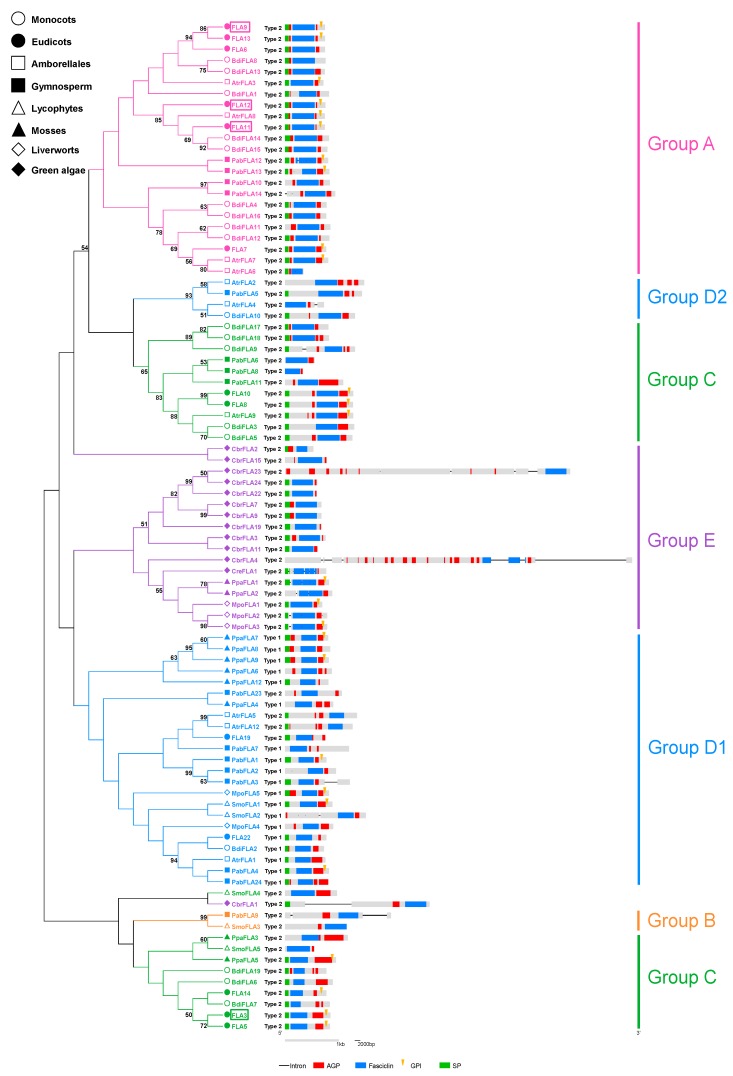
Phylogenetic relationships and structure display of FLAs with single fasciclin domain in nine plant species (*C. reinhardtii*, *C. crispus*, *M. polymorpha*, *P. patens*, *S. moellendorffii*, *P. abies*, *A. trichopoda*, *B. distachyon*, and *A. thaliana*). Plant species from different lineages are shown in different shapes. The phylogenetic trees were built by Mega 7 using the Maximum Likelihood (ML) method under LG+G model with 85% partial deletion. Bootstrap analyses with 1000 replicates were performed for support estimation, the confidence values higher than 50% are shown on nodes. The tree was divided into six groups according to the classifications based on two types fasciclin domains (Figure 2 and Figure 3): Group A, Group B, Group C, Group D1, Group D2 and Group E. The structure displays were generated by GSDS 2.0. Black lines represent introns, gray rectangles the CDS regions, red rectangles the AGP regions, blue rectangles the fasciclin domains, green rectangles signal peptides, and yellow wedges GPI-anchor modification sites. The framed FLAs denote functionally characterized FLAs (FLA3, FLA9, FLA11, and FLA12).

Moreover, to understand the relationship between FLAs with single fasciclin domain, a phylogenetic tree of FLAs with single fasciclin domain from nine plant species (*C. reinhardtii*, *C. crispus, M. polymorpha, P. patens*, *S. moellendorffii*, *P. abies*, *A. trichopoda*, *B. distachyon*, and *A. thaliana*) was built by the Maximum Likelihood (ML) method under the LG + G model with 85% partial deletion. Bootstrap analyses with 1000 replicates were performed for support estimation; confidence values higher than 50% were shown on nodes. The structure displays of these FLAs were generated by GSDS 2.0 (available online: http://gsds.cbi.pku.edu.cn/) [44] (Figure 4). The structure of Group A *FLA* genes was very similar. Except for *PabFLA12*, *PabFLA14*, and *AtrFLA6*, the remaining Group A *FLA* genes did not contain introns, and most of their fasciclin domains were flanked by two AGP regions. The structures of FLAs with single fasciclin domains in Group E were quite diverse. By contrast, the phylogenetic relationship of FLAs with single fasciclin domain was similar to the phylogenetic relationships of Type 2 (Figure 3). The main type of fasciclin domain in these FLAs was Type 2 fasciclin domain. Most of Group D1 FLAs contained Type 1 fasciclin domains. It is likely that the Type 1 fasciclin domain was lost mainly in FLAs with single fasciclin domain over the course of evolutionary history. Different from phylogenetic relationships of Type 1 and Type 2 fasciclin domains (Figure 2 and Figure 3), Group C appeared to be divergent (Figure 4). Some Group C FLAs were close to Group D2, while others were close to Group B. Moreover, the structure of these diverged Group C was different. The fasciclin domains of FLAs tailed with AGP regions belonged to Group C, which were close to Group B. For FLAs from Group C which was close to Group D2, their fasciclin domains were covered by two AGP regions.

### 2.3. Structural and Evolutionary Analysis of FLAs

**Figure 5:**
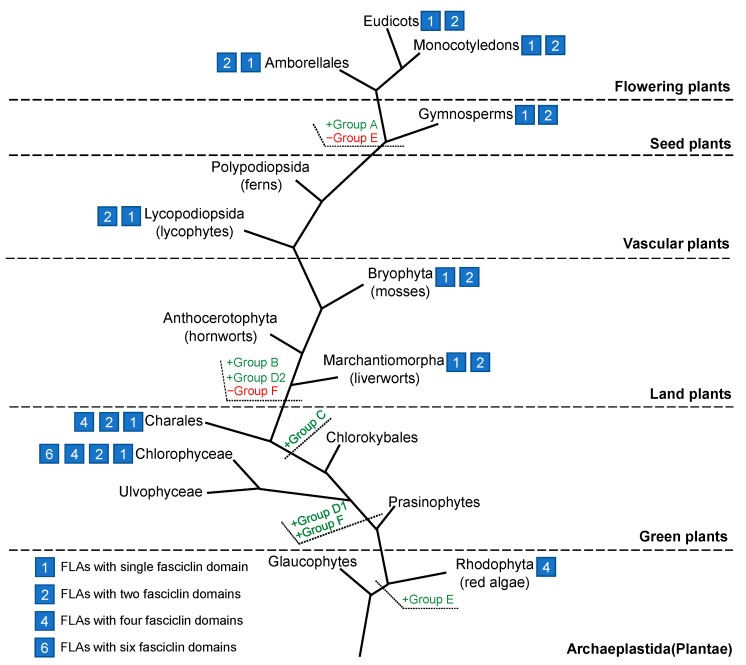
Evolutionary model of the FLA family in plants. The green letters display the appearance of different groups of FLAs. The red letters display the disappearance of Group E and Group F FLAs. The cubes display the number of fasciclin domains in FLAs.

The amino acid sequences of 246 FLAs identified in our work were shown in Figure S4. One hundred seventy-six of them contained a single fasciclin domain, and 66 of them contained two fasciclin domains. Only four FLAs with more than two fasciclin domains were found in algae, one in red algae and three in green algae. Moreover, FLAs with a single fasciclin domain, as well as with two domains first appeared in green algae (Figure 5). It was likely that divergence happened in green algae. From green algae to land plants, the number of fasciclin domains in FLAs was reduced. It had been proven that FLAs with a single fasciclin domain had conserved roles in secondary cell wall biology and properties [13]. Besides, there was an example of the functional roles of different fasciclin domains in one FLA protein. The C-proximal fasciclin domain of FLA4 was responsible for its genetic functions, while the N-proximal fasciclin domain was required for stabilization of plasma membrane localization [60,61]. It was likely that the number of fasciclin domains was related to the functions of FLAs.

FLAs were classified into seven groups based on the sequence similarity, phylogenetic analysis, and previous study [11]. Different from the previous study [11], Group D was divided into Group D1 and Group D2 because of their difference in phylogenetic analysis. Moreover, Group E and Group F present in non-seed plants are the groups newly proposed in this work. The evolutionary history of FLA family was shown in Figure 5. FLAs evolved very early during plant evolution. Group E first appeared in the plant kingdom, then Group F, Group D1, Group C, Group D2, Group B, Group A appeared successively. The Group E FLA from red algae was the most original FLA. Group F was largely dissimilar to the other groups and only existed in green algae. Group D1 and Group C evolved early during green plant evolution. The divergence of FLAs occurred in green algae; Group D1 and Group C remained, while Group F was lost after the separation between green algae and land plants. Group B and Group D2 evolved after plants conquered the land. Group A, the latest group appeared, evolved during seed plant evolution. By contrast, Group E, the earliest appeared group, was lost in seed plants.

### 2.4. Analysis of FLA Duplication Patterns during the Process of Evolution

**Figure 6:**
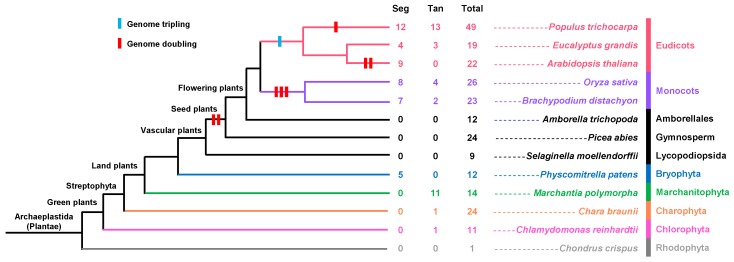
Duplication events of *FLA* genes in the plant kingdom. The phylogenetic tree on the left was built based on the Tree of Life Web project (available online: http://www.tolweb.org/Green_plants) and whole-genome duplication events in PGDD (available online: http://chibba.pgml.uga.edu/duplication/index/home). The number next to the tree is the number of *FLA* genes resulting from segmental duplication, tandem duplication, and total *FLA* genes in the species. Seg: Segmental duplication (pairs); Tan: Tandem duplication (pairs); Total: Total number of *FLA* genes in the species.

The evolution of genomes and genetic systems is mainly driven by gene duplications [62]. The three elementary gene expansion patterns are tandem duplication, segmental duplication, and transposition events [63,64]. In the plant kingdom, tandem duplication and segmental duplication are the main processes of gene family expansion compared with transposition events [65,66]. We investigated these two duplication events to understand the *FLA* genes’ expansion patterns in the plant kingdom. The paralogous genes that exist in the same chromosome within a 50 kb physical distance are examples of tandem duplication [65]. First, in order to find the chromosomal locations, the annotation information for the *FLA* genes was downloaded from OrcAE (available online: https://bioinformatics.psb.ugent.be/orcae/overview/Chbra), Phytozome (available online: https://phytozome.jgi.doe.gov/pz/portal.html) and ConGenIE (available online: http://congenie.org/). Then, the distances between *FLA* genes’ locations were compared in the same chromosome. The locus search tool on PGDD (available online: http://chibba.agtec.uga.edu/duplication/index/locus) and MCSCAN were used to find the segmental duplications (Table S2). The duplications in *FLA* genes were related to whole-genome duplication events (Figure 6). The higher plants exhibited more duplications than lower plants. *P. trichocarpa* had the highest number of duplicated *FLA* genes, which made it have more *FLA* genes than other plant species. Although most duplicated pairs shared the same structure type, some duplicated genes had different structure types. For example, in *C. reinhardtii*, *Cre16.g687742* containing two fasciclin domains and *Cre16.g687854* containing single fasciclin domain most probably result from tandem duplication. It seemed that some *FLA* genes with single fasciclin domain evolved from *FLA* genes with two fasciclin domains. FLAs with single fasciclin domain evolved from FLAs with multiple fasciclin domains, and the number of fasciclin domains was reduced in evolutionary history. 

In order to understand the evolution processes of the *FLA* gene family in the plant kingdom, duplicated gene pairs among *FLAs* were used to estimate the molecular evolutionary rates by calculating their Ka/Ks value (Table S2). The Ka/Ks values of all the duplicated gene pairs except the *Mapoly0075s0013.1*/*Mapoly0075s0013.2* gene pair were lower than 1. It was assumed that FLA duplicated gene pairs evolved under purifying selection, indicating that the functions of the *FLAs* gene family were crucial to plant development and functional mutations in *FLA* genes might have negative impacts on plants. The Ka/Ks ratio of *Mapoly0075s0013.1*/*Mapoly0075s0013.2* gene pair was 2.3512, showing that this gene pair underwent positive selection pressure during evolution. However, plants could not escape from their environment in order to adapt to changes, so positive selection, which could lead to beneficial functional changes, was also important during plant evolution [67]. The *Mapoly0075s0013.1*/*Mapoly0075s0013.2* gene pair, which was found to experience positive selection, might have improved the adaptation of the plant to new environments.

## 3. Materials and Methods

### 3.1. Bioinformatics Identification of FLAs

Multiple searches were carried out in order to identify FLA genes in 13 plant species (*C. crispus, C. reinhardtii, C. crispus, M. polymorpha, P. patens, S. moellendorffii, P. abies, A. trichopoda, B. distachyon, O. sativa, A. thaliana, E. grandis*, and *P. trichocarpa*) [31,32,33,34,35,36,37,38,39,40,41,42,43]. The predicted proteomes of *C. crispus* was downloaded from NCBI, that of *C. braunii* were from the OrcAE website (available online: https://bioinformatics.psb.ugent.be/orcae/overview/Chbra), that of P. abies were from the ConGenIE website (available online: http://congenie.org/), and that of other species from the Phytozome website (Version 12; available online: https://phytozome.jgi.doe.gov/pz/portal.html). Except for *P. abies* [35], the statistics of genome size overall number of predicted genes were from the NCBI Genome database (available online: https://www.ncbi.nlm.nih.gov/genome).

Then, the Hidden Markov Model (HMM) profile built for fasciclin domains was downloaded from Pfam (available online: http://pfam.xfam.org/family/PF02469) [68], and HMMER 3.0 [69] was used to search proteins with fasciclin domains from the selected plants. Then the presence of fasciclin domains corresponding to the obtained proteins was examined by the NCBI conserved domain database (available online: http://www.ncbi.nlm.nih.gov/cdd). Next, the Finding-AGP program [7] was used to identify AGP regions from proteins with fasciclin domains. Finally, proteins with both fasciclin domains and AGP regions were identified as FLAs. Also, the omitted FLA sequences that were identified in former studies (AT5G40940, AT5G06920, Eucgr.A01741, Potri.013G152200, Potri.001G440800, and Potri.005G079500) were used as queries to perform BLAST searches with a −3 expect (E) threshold to find FLAs that could not be identified by HMMER 3.0.

Moreover, most FLAs have a predicted signal peptide and GPI-anchor. Therefore, SignalP 4.1 Server (available online: http://www.cbs.dtu.dk/services/SignalP/) was used to predict signal peptides [70] and big-PI Plant Predictor (available online: http://mendel.imp.ac.at/gpi/plant_server.html) was used to predict GPI modification sites [71]. The intron of red algae *FLA* was detected by the GSDS website (available online: http://gsds.cbi.pku.edu.cn/) [44], and the intron of other *FLAs* were found from the OrcAE website (available online: https://bioinformatics.psb.ugent.be/orcae/overview/Chbra), the Phytozome website (Version 12; available online: https://phytozome.jgi.doe.gov/pz/portal.html), and the ConGenIE website (available online: http://congenie.org/). The amino acid sequences and the presence of AGP regions, signal peptides, fasciclin domains, and GPI-anchor signals are given in Table S1.

### 3.2. Multiple Sequence Alignment and Phylogenetic Analysis

All of the FLA protein sequences were searched against each other by BLAST+ with a −5 expect (E) threshold [45]. The sequences with low similarity were removed. Then, signal peptides and GPI modification sites were removed from filtered FLA sequences. These sequences were aligned by Clustal Omega 1.2.2 with HMM of the fasciclin domain as a guide in the alignment [46,47]. The fasciclin domains were designated as Type 1 and Type 2 and were also aligned by Clustal Omega 1.2.2 with the HMM of the fasciclin domain as a guide in the alignment [46,47]. GeneDoc [72] was used to display multiple sequence alignments.

The reliability of alignment results was tested by computing overall mean distance with the P-distance method by Mega 7 [49,51]. The alignments of Type 1, Type 2, and FLAs with a single fasciclin domain was then used to build phylogenetic trees with the Maximum Likelihood (ML) method. The best models for ML trees were found by Mega 7 [51,73]. Then, ML trees were built under the best model with 85% partial deletion by Mega 7. Bootstrap analyses with 1, 000 replicates were performed for support estimation [51,52].

### 3.3. Motif Prediction

In order to identify the conserved domains and motifs of Type 1 and Type 2 fasciclin domains, MEME web server (available online: http://meme-suite.org/tools/meme) [53] was used to identify the conserved motifs (H1 and H2 regions, YH motif). The following parameters were used when running the MEME: (1) The motif sites in sequences were distributed by 0 or 1 occurrence per sequence; (2) the maximum of motifs was set to be 10 for the H1 and H2 regions, and 3 for the YH motif; and (3) a 0-order model of sequences was used as the background model.

### 3.4. Gene Duplication and Molecular Evolution

The annotation information of the *FLA* genes on the phytozome website (available online: https://phytozome.jgi.doe.gov/pz/portal.html), the OrcAE website (available online: https://bioinformatics.psb.ugent.be/orcae/overview/Chbra), and the ConGenIE website (available online: http://congenie.org/) was used to find the chromosomal locations. The paralogous genes that exit in the same chromosome within a 50-kb physical distance was defined as tandem duplication [64]. The segmental duplications of 10 plants (*C. reinhardtii*, *P. patens*, *S. moellendorffii*, *P. abies*, *A. trichopoda*, *B. distachyon*, *O. sativa*, *A. thaliana*, *E. grandis*, and *P. trichocarpa*) were found by the PGDD locus search tool (available online: http://chibba.agtec.uga.edu/duplication/index/locus). Because *M. polymorpha and C. crispus* data were absent in PGDD, Multiple Collinearity Scan (MCSCAN) [74,75,76,77] was used to find the segmental duplications in *M. polymorpha*.

To calculate the molecular evolutionary rates between *FLAs* duplicated gene pairs, pairwise alignment was performed among these gene pairs by ClustalW (codons) in MEGA7 [51]. Then, the MYN (Modified YN) model in KaKs_Calculator 2.0 was used to estimate the nonsynonymous substitution rate (Ka), the synonymous substitution rate (Ks) and the Ka/Ks value of these duplicated gene pairs [78].

## 4. Conclusions

FLAs play an important role in plant development and adaption to the environment. Two hundred forty-six *FLA* genes in 13 plant species were identified in this study. It was found that FLAs first appeared in algae. Based on the sequence similarity and phylogenetic analysis, FLAs could be classified into seven groups: Group A, Group B, Group C, Group D1, Group D2, Group E, and Group F. Group E FLAs were the earliest to appear in evolutionary history and disappeared in seed plants, while Group A FLAs were the latest and only existed in seed plants. FLAs with multiple fasciclin domain (>2) were possibly the first FLA type to appear in Archaeplastida because they only existed in algae. FLAs with single fasciclin domain and with two fasciclin domains were dominant in green plants. The number of fasciclin domains in FLAs varied in green algae and was reduced to one or two in land plants. In addition, introns in *FLA* genes were lost during plant evolution, especially from green algae to land plants. Moreover, tandem and segmental duplications contributed to the expansion of the *FLA* gene family, and duplicated gene pairs in *FLAs* mainly evolved under purifying selection.

## Figures and Tables

**Table 1:** Information about genome size and fasciclin-like arabinogalactan protein (FLA) gene number in the plants of interest for this study.

Lineage	Organism	Genome Size (Mb)	No. of Predicted Genes	No. of *FLA* Genes	Reference
Red algae	*Chondrus crispus*	104.98	9843	1	This study
Green algae	*Chlamydomonas reinhardtii*	120.405	14,488	11	This study
	*Chara braunii*	1751.21	35,424	24	This study
Liverworts	*Marchantia polymorpha*	215.739	19,287	14	This study
Mosses	*Physcomitrella patens*	472.081	23,733	12	This study
Lycophytes	*Selaginella moellendorffii*	212.315	34,782	9	This study
Gymnosperm	*Picea abies*	19,600	28,354	24	This study
Amborellales	*Amborella trichopoda*	706.495	19,354	12	This study
Eudicots	*Arabidopsis thaliana*	119.148	38,093	22	Schultz et al. [21]
	*Eucalyptus grandis*	691.43	45,226	19	MacMillan et al. [13]
	*Populus trichocarpa*	434.29	37,197	49	Showalter et al. [24]
Monocots	*Brachypodium distachyon*	218.345	34,310	23	This study
	*Oryza sativa*	374.423	33,185	26	Ma and Zhao [12]

## Supplementary Materials

Supplementary materials can be found at https://www.mdpi.com/1422-0067/20/8/1945/s1.

## Abbreviations

FLA	Fasciclin-like arabinogalactan protein
AGP	Arabinogalactan protein
GPI	Glycosylphosphatidylinositol
PAST%	The percentage of Pro, Ala, Ser, and Thr residues in a protein amino-acid sequence
Ccr	*Chondrus crispus*
Cre	*Chlamydomonas reinhardtii*
Mpo	*Marchantia polymorpha*
Smo	*Selaginella moellendorffii*
Pab	*Picea abies*
Atr	*Amborella trichopoda*
Egr	*Eucalyptus grandis*
Pt	*Populus trichocarpa*
Bdi	*Brachypodium distachyon*
Os	*Oryza sativa*
Ka	Nonsynonymous substitution rate
Ks	Synonymous substitution rate
PGDD	Plant Genome Duplication Database
NCBI	National Center for Biotechnology Information
ConGenIE	Conifer Genome Integrative Explorer
HMM	Hidden Markov Model
BLASTP	Protein Basic Local Alignment Search Tool
MYN	Modified Yang-Nielsen Algorithm
MCSCAN	Multiple Collinearity Scan
MEME	Multiple Expectation maximization for Motif Elicitation
LG	Le_Gascuel_2008 model
G	Gamma distribution
I	Evolutionarily invariable
GSDS	Gene Structure Display Server
OrcAE	Online Resource for Community Annotation of Eukaryotes
