# Supplementary material for: Evolution Analysis of the Fasciclin-Like Arabinogalactan Proteins in Plants Shows Variable Fasciclin-AGP Domain Constitutions
Source: Int J Mol Sci. 2019 Apr 20;20(8):1945. doi: 10.3390/ijms20081945 (PMC6514703; doi:10.3390/ijms20081945)
Supplement: Supplementary file 1 [file ijms-20-01945-s001.zip › Supplementary files/Figure S1.pdf]

Figure S1. Multiple sequence alignment of FLA sequences. Fasciclin domains were divided into two types (Type 1 and Type 2). Residues with high similarity (80%, 60%) were highlighted in dark pink and light pink, respectively.

Type 1

```
                       *        20         *        40         *        60         *        80         *       100         *       120         *       140         *       160
AtrFLA10    : -----------------QTLEEAV-GKH--NITIFAPRNEALERDLDPE--FKRFLLEPGNLKSLQSLILFHVIPRRIPAKNWPESDAHSH-----DTLSSDRL-T---LT-----------------CKNRVRKVD--L------ASLIHPDS-IVRP----DGVIH : 107
AtrFLA11    : HEFSKFNDYLSR-TH----LASEINRRT--TITVLAVDNAAMDDILAKN----------YGIYEIKNILSLHILLDYFGAKKLHQITNGT----------------------ALAATLYQTTGSAPGTSGFVNITDEKGG----K-VSIGADDNGG-VL----DASYV : 119
AtrFLA5     : ------------------------------------------------------------------------------------------------------------------------------------------------------------------------ :   -
AtrFLA1     : --------------------------------TFLMPSDKDLANYN--F--------LP---GQLKELMLTHSIPNSMLFKHMLHFPTGT-L---LPSARHSRFLNVSNHGS-------------------YSFYLN--N------ARIVAPNV-CGST----NIRCH :  87
AtrFLA2     : ------------------------------------------------------------------------------------------------------------------------------------------------------------------------ :   -
AtrFLA7     : ------------------------------------------------------------------------------------------------------------------------------------------------------------------------ :   -
AtrFLA3     : ------------------------------------------------------------------------------------------------------------------------------------------------------------------------ :   -
AtrFLA8     : ------------------------------------------------------------------------------------------------------------------------------------------------------------------------ :   -
AtrFLA9     : ------------------------------------------------------------------------------------------------------------------------------------------------------------------------ :   -
AtrFLA4     : ------------------------------------------------------------------------------------------------------------------------------------------------------------------------ :   -
AtrFLA6     : ------------------------------------------------------------------------------------------------------------------------------------------------------------------------ :   -
AtrFLA12    : ------------------------------------------------------------------------------------------------------------------------------------------------------------------------ :   -
FLA16       : SHYTELAELVEK-ALLLQTLEEAV-GKH--NITIFAPRNDALERNLDPL--FKSFLLEPRNLKSLQSLLMFHILPKRITSPQWPSLSH---HHR---TLSNDHL-H---LT-----------------VDVNTLKVD--S------AEIIRPDD-VIRP----DGIIH : 122
FLA17       : SRYTELAELVEK-ALLLQTLEDAV-GRH--NITIFAPRNEALERDLDPE--FKRFLLEPGNLKSLQTLLMFHIIPNRVGSNQWPSEESGRVKHH---TLGNDQV-R---LS-----------------NGQGKKMVD--L------AEIIRPDD-LTRP----DGLIH : 125
FLA15       : SHYTELAELVEK-ALLLQTLEEAV-GQH--NITIFAPRNDALEKNLDPE--FKSFLLQPKNLKSLQSLLMFHILPKRITSPQFSSAVV---SHR---TLSNDHL-H---FT-----------------N----GKVN--S------AEITKPDD-LTRP----DGIIH : 118
FLA18       : SRYTELAELVEK-ALLLQTLEDAV-GRH--NITIFAPRNEALERDLDPD--FKRFLLQPGNLKSLQTLLLSHIIPKRVGSNQWPEENSGRVKHV---TLGHDQVLHLSKLK-----------------GTNGKRLVN--S------AVITRPDD-LTRP----DGLIH : 129
FLA1        : PSFSSFSHFLTQ-TH----LADEINRRR--TITVCAVDNAAMSALTSKG----------YTLSTLKNILSLHVLLDYFGTKKLHQIRDGS----------------------ALAATLFQATGAAPGTSGFVNITDLRGG----K-VGFGPDGG---DL----SSFFV : 117
FLA22       : --------------------------------TFLMPKDKTLSTSNIIN--------QQ---DSVTEFLLRHSIPSSLLFEHLNLIPNGS-I---VPSSLPHYTLEISNGGR-------------------SNYFLN--N------VKIISRNI-CSLG----SIKCH :  89
FLA20       : --------------------------------TLFAPSDQSFSKFGQP---------SL-------LDMKYQLSPTRLPGETLRNLPNGA-K---IPTLRSNYSLTVTNS-S----------------RFGGKTSIN--N------VVVQDSPV-FDDG----YVVIY :  86
FLA21       : --------------------------------TLFAIEDASFFNTSSL---------HP---LFLKQLLHYHTLPLMLSMDDLLKKPQGT-C---LPTLLHHKSVQISTV-N----------------QESRTAEVN--H------VRITHPDM-FLGD----SLVIH :  90
FLA19       : ------------------------------------------------------------------------------------------------------------------------------------------------------------------------ :   -
FLA4        : PNLSSFSNLLVS-SG----IAAELSGRN--SLTLLAVPNSQFSSASLDLTRRLPP-------SALADLLRFHVLLQFLSDSDLRRIPPSG----------------------SAVTTLYEASGRTFFGSGSVNVTRDPAS----GSVTIGSPAT-KN-------VTVL : 120
FLA10       : ------------------------------------------------------------------------------------------------------------------------------------------------------------------------ :   -
FLA8        : ------------------------------------------------------------------------------------------------------------------------------------------------------------------------ :   -
FLA11       : ------------------------------------------------------------------------------------------------------------------------------------------------------------------------ :   -
FLA12       : ------------------------------------------------------------------------------------------------------------------------------------------------------------------------ :   -
FLA7        : ------------------------------------------------------------------------------------------------------------------------------------------------------------------------ :   -
FLA9        : ------------------------------------------------------------------------------------------------------------------------------------------------------------------------ :   -
FLA6        : ------------------------------------------------------------------------------------------------------------------------------------------------------------------------ :   -
FLA13       : ------------------------------------------------------------------------------------------------------------------------------------------------------------------------ :   -
FLA14       : ------------------------------------------------------------------------------------------------------------------------------------------------------------------------ :   -
FLA3        : ------------------------------------------------------------------------------------------------------------------------------------------------------------------------ :   -
FLA5        : ------------------------------------------------------------------------------------------------------------------------------------------------------------------------ :   -
FLA2        : PDFSTFNHYLSA-TH----LADEINRRQ--TITVLAVDNSAMSSILSNG----------YSLYQIRNILSLHVLVDYFGTKKLHQITDGS----------------------TSTASMFQSTGSATGTSGYINITDIKGG----K-VAFGVQDDDS-KL----TAHYV : 119
CreFLA11.1  : ------------------------------------------------------------------------------------------------------------------------------------------------------------------------ :   -
CreFLA11.4  : ------------------------------------------------------------------------------------------------------------------------------------------------------------------------ :   -
CreFLA1     : ------------------------------------------------------------------------------------------------------------------------------------------------------------------------ :   -
CreFLA10.2  : ------------------------------------------------------------------------------------------------------------------------------------------------------------------------ :   -
CreFLA9     : PNLTTYLRALRA-GE----LMGRLNDVQAPPVTLFVADDSAWQVFAADAGTSVDALLTEAGGALVRHLLLYGLV----PTPLSPQQLAASPV---LNTSWAGQMVFVPQPFMGAVSSSAGTEARITGRVKVCESYAYR-V------AYVLMPAL-SL----------- : 137
CreFLA8.1   : ------------------------------------------------------------------------------------------------------------------------------------------------------------------------ :   -
EgrFLA9     : SHYTELAELVEK-ALLLQTLEETV-GRH--NITIFAPKNEALERDLDPE--FKSFLLEPRNLKSLQTLLMFHIVPSRIDSADWPEPAPSAAGHREELSLSSHRL-RLA-KR------------------ESGEREVD--R------ARVIHADA-VARP----DGVIH : 129
EgrFLA5     : SHYTELAELVEK-ALLLQSLEDAV-GRH--NLTIFAPRNEALERDLDPE--FRRFLLEPRNLRSLQTLLLSHIVPARLGSPQWPLPDSRPAR---HPTLGSDPV-HLS-VA-----------------PGSGLPAVD--S------AEVVRPDD-VVRP----DGVIH : 127
EgrFLA10    : PDFSTFNHYLSA-TH----LAAEINSRT--TITVLAVSNAAMNDLLAEH----------PTTTTLKNILSLHVLLDYFGSKKLHQLTDGS----------------------ALAATLFQSTGSAPGSSGFVNITDLKGG----K-VGFAPEEGGSSDA----GAYFV : 120
EgrFLA11    : PEFSTFNHYLTE-TH----LAAEINSRT--TITVMALDNAAMSRLLAPH----------PTIATLKNVLSLHVLLDYFGPKKLHQITNGT----------------------ALAATLFQATGSAPGSTGFVNITDLKGG----K-VGFAPEDNNG-RL----DTYFV : 119
EgrFLA16    : --------------------------------TVFSPADDSFARFGQP---------PL-------SLLQFHFSPHALSLDTLRSLPRGA-K---ISTMNANHSLMVTTSPS----------------DYQ--VEIN--G------VKIVGSPV-FNDG----NLVVF :  85
EgrFLA14    : --------------------------------TVFSPSDDSFAQFGQP---------PL-------SLLQFHFSPHALSLDTLRSLPHGA-K---ISTMNANHSLMVTTSPS----------------DYQ--VQIN--S------VKIIGSPV-FNDG----NLVIF :  85
EgrFLA15    : --------------------------------TVFSPSDDSFARFGQR---------PL-------SLLQFHFSPHALSLDTLRSLPHGA-K---ISTMNANHSLMVTTSPS----------------DYQ--VQIN--G------VKIVGSPV-FNDG----NLVVF :  85
EgrFLA19    : --------------------------------TFFMPDDGMLSRIV--L--------PT---NDVSHFVLRHSIPKPLLFDYLEHIPTGS-L---IPTSAAGYLVRIDNKGC-------------------RSYFLD--D------VRITKPNV-CVAGS---SIRCH :  88
EgrFLA4     : ------------------------------------------------------------------------------------------------------------------------------------------------------------------------ :   -
EgrFLA12    : PEFSTFNHYLTI-TH----LAAEINRRL--TITVLAVDNAAMNAILDKH----------LSVGTLKNVLSLHVLVDYFGTKKLHQISNGT----------------------TLTSTMFQATGAAAGTAGYVNITDLKGG----K-VGFGAEDNGS-DL----NAVFV : 119
EgrFLA8     : ------------------------------------------------------------------------------------------------------------------------------------------------------------------------ :   -
EgrFLA7     : ------------------------------------------------------------------------------------------------------------------------------------------------------------------------ :   -
EgrFLA13    : ------------------------------------------------------------------------------------------------------------------------------------------------------------------------ :   -
EgrFLA6     : ------------------------------------------------------------------------------------------------------------------------------------------------------------------------ :   -
EgrFLA1b    : ------------------------------------------------------------------------------------------------------------------------------------------------------------------------ :   -
EgrFLA3b    : ------------------------------------------------------------------------------------------------------------------------------------------------------------------------ :   -
EgrFLA2b    : ------------------------------------------------------------------------------------------------------------------------------------------------------------------------ :   -
EgrFLA17    : --------------------------------TIFVIKDLFMANL-SS---------SP---WLMEEVLRYHTSPLKLSVDDLLKMPRGS-C---LPTLLAKKNVTVTRS-D----------------AEQRLVEIN--G------AVITHPNV-FLGE----RLAIH :  89
EgrFLA20    : --------------------------------TLFAPSDSSLKSCAS---------------CSVPRILSEHIVPGLFSFGYIQKLAFGT-K---VETMSPGHCLTVTSATHR------------V--TNTTRIFID--G------VEISRPDM-FNNG----IVVVH :  90
OsaFLA17    : SRYTEMAELVEK-ALLLQALEDAV-AEH--NVTILAPRNEALERDIDPE--FKRFLLEPRNLNSLQSLLLFHVLPARLPSGSWPAA---AT----HPTLAGEDV-ELAAAD-----------------GGGGMRVA---H------AAVSRPDA-VVRT----DGVIH : 123
OsaFLA24    : SHYTELAELVEK-ALLLQTLEDAV-GKH--NVTIFAPRNEALERDLDPE--FKRFLLEPRNLKSLQSLLLFHVLPARLPSGSWSA----VS----HPTLSGEEV-ELAAAA-----------------NDGAMRV-A--H------AAVTRPDA-VLRP----DGVIH : 122
OsaFLA16    : SHYTELAELVEK-ALLLQTLEDAV-GKG--NVTIFAPRNEALERDLDPE--FRRFLLEPRNLRSLQRLLLFHVLPARLHASDSSSPDFPSS----HPTLSGERV-DLS---------------------ASPMRV-G--A------AAVTRPDA-VVRP----DGVIH : 122
OsaFLA4     : ------------------------------------------------------------------------------------------------------------------------------------------------------------------------ :   -
OsaFLA23    : ------------------------------------------------------------------------------------------------------------------------------------------------------------------------ :   -
OsaFLA12    : PSFADFARLLES-SP----VAGELAARS--SLTLLAVPNNNLPRSPSAF-AAASG-------ADIADVLRYHVLLEYLSPSDLARLPASG----------------------KLVTTLFQTTGRAPSDLGAVNLTVGGN----STVVVR-SPAP-FPGS----SATVL : 121
OsaFLA1     : ------------------------------------------------------------------------------------------------------------------------------------------------------------------------ :   -
OsaFLA5     : ------------------------------------------------------------------------------------------------------------------------------------------------------------------------ :   -
OsaFLA8     : ------------------------------------------------------------------------------------------------------------------------------------------------------------------------ :   -
OsaFLA27    : ------------------------------------------------------------------------------------------------------------------------------------------------------------------------ :   -
```

Type 1

```
OsaFLA26   : ------------------------------------------------------------------------------------------------------------------------------------------------------------------------ :   -
OsaFLA7    : ------------------------------------------------------------------------------------------------------------------------------------------------------------------------ :   -
OsaFLA14   : ------------------------------------------------------------------------------------------------------------------------------------------------------------------------ :   -
OsaFLA2    : ------------------------------------------------------------------------------------------------------------------------------------------------------------------------ :   -
OsaFLA11   : PEFSKFNEMLSK-TR----LAYDINRRQ--TITVLAVDNSAMASL--DH----------FTLPTIRHILSLHILVDYYGSKKLHALSHGA----------------------TASSSMFQATGSAPGTTGYVNITSHKGG----K-IDFISEDADESAK----PSRYV : 118
OsaFLA9    : ------------------------------------------------------------------------------------------------------------------------------------------------------------------------ :   -
OsaFLA3    : ------------------------------------------------------------------------------------------------------------------------------------------------------------------------ :   -
OsaFLA18   : ------------------------------------------------------------------------------------------------------------------------------------------------------------------------ :   -
OsaFLA13   : ------------------------------------------------------------------------------------------------------------------------------------------------------------------------ :   -
OsaFLA15   : ------------------------------------------------------------------------------------------------------------------------------------------------------------------------ :   -
OsaFLA19   : ------------------------------------------------------------------------------------------------------------------------------------------------------------------------ :   -
OsaFLA10   : ------------------------------------------------------------------------------------------------------------------------------------------------------------------------ :   -
OsaFLA6    : ------------------------------------------------------------------------------------------------------------------------------------------------------------------------ :   -
OsaFLA25   : ------------------------------------------------------------------------------------------------------------------------------------------------------------------------ :   -
OsaFLA20   : ------------------------------------------------------------------------------------------------------------------------------------------------------------------------ :   -
OsaFLA28   : --------------------------------TFLMPNDRMLSTAT--I--------PE---SQVMEFLSRHSIPAPLMFDDLIKLPNAT-I---VPTAHSSQTITITNVKH-------------------QKIYFN--N------IELISPDV-CRVGD---LFRCH :  88
PabFLA22   : SRYTELAELVEK-ASLLQPLEEVV-GNN--NITILAPQNAHLEKNLDPE--FKRFMHEPGNLNSLQRLLQSHIIPRRITAEEWSNNAT-------HEALSKDGL---QFSV-----------------KENNIKAVD--L------AVLTRPNA-VVRP----DGVIH : 122
PabFLA9    : ------------------------------------------------------------------------------------------------------------------------------------------------------------------------ :   -
PabFLA1    : --------------------------------TFFVPSDDALSRLPVQ----------A---EFTSSILQYHIIPQQLSSYDLQRLDVGM-R---LPTLLANQTIVVTSNTQ-------------------PNYTVD--D------TLVTHPDI-FYDG----TFAVH :  87
PabFLA2    : --------------------------------TFFVPSDDALSRLPVQ----------T---EFTSSILQYHIIPQQLSSYDLQRLDVGM-R---LPTLLANQTIVVTSNRQ-------------------PNYTVD--D------TLVTHPDI-FYDG----TFAVH :  87
PabFLA3    : --------------------------------TFFVPSDDALSRLPVQ----------T---EFTSSILQYHIIPQQLSSYALQRFDVGM-R---LPTLLANQTILVTSNRQ-------------------PNYTVD--D------TLVTHPDI-FYDD----TFAVH :  87
PabFLA4    : --------------------------------TFFMPSDQALAEVNL-Q--------VT---SYISELVLRHSVHQPLKFNHLLHFPTGT-L---IPSSFPNYMLKVSNNGR-------------------NDFYLN--Y------VHIVHPNV-CRASP---SISCH :  89
PabFLA17   : SHYTELAELVEK-ALLLQTLEEVV-GSS--NVTIFAPQNDILERKLDPE--FKRFLHEPGNLKALQKLLMFHVVPRRIVAEEWRNG-T-------HQTLAKDRL---KLSM-----------------KGDKKT-ID--L------ASVAHPNA-VVRP----DGVIH : 120
PabFLA15   : PEFETFNKLLTD-TN----VAKDINNRT--SLTILALPNSILENYMARFGGNLQV-------MEIESVLQYHVLLEYMDWDRLHQMTNRG----------------------TLITTLYQTTGRAPKDSGSVNITCEKNG---EIRIRAPA-PY-GGSF----NATVT : 123
PabFLA5    : ------------------------------------------------------------------------------------------------------------------------------------------------------------------------ :   -
PabFLA20   : PEFTTFNNLLTA-TN----VAGEINNRS--SLTILALSNAQLEPFISSFGGNLPT-------QEVSDVLRYHVLLEYLDWERLKMMPAAG----------------------TLVTTLYQTTGRAPNNTGAVNITTGGGN---NGQVRIGMPVP-GAAY----NATID : 124
PabFLA21   : PEYSKFNHDLSQ-TK----LADEINSRQ--TITVLALSNSEMASLSG------------LDLASLKRVLSLHVVLDFFDAKKLHEITNGT----------------------TLSTTLYQTTGNAPGNTGFINITDLKAG----K-VGFGPAAHGA-KL----DATYV : 117
PabFLA16   : -DYSSFNSYLTQ-TK----LADEINSRT--TITVLALNNGAMSVLTSKR----------LSLSVMKKVLSLHVLLDYFDGEKLHQISNGT----------------------MLSTTLYQTSGNALGKSGFLNITDLKGG----K-VGFGAASPGA-KL----DSMFV : 118
PabFLA10   : ------------------------------------------------------------------------------------------------------------------------------------------------------------------------ :   -
PabFLA6    : ------------------------------------------------------------------------------------------------------------------------------------------------------------------------ :   -
PabFLA11   : ------------------------------------------------------------------------------------------------------------------------------------------------------------------------ :   -
PabFLA18   : --FTSLGMHLQ--SS----Q-DLLV-GLKGPLTLFAPTEEAFRYFNSP---------NSSSNSSSDYLLPYHIAKGLFTYTQLQMLPDGT-K---LDTIASEGSLLVTFNSRGDGQDLIYSSAQTL--VDYRPVYVN--G------VLVSHPDL-LNDG----LLTIH : 130
PabFLA7    : -----FANALQM-VD----I-THLF-SSGGSYTFFAPPDEALEALGSA---------SKS-WQEFMDAVRYHIVNVQLSYYNLKHLPDGA-R---LGTLLRSRSLTFTTFAPE---------------SENETLMVN--N------VSVSFPDL-------------- : 105
PabFLA19   : ---------------------NLLV-GRAGPLTIFAPTEEAFRNFNSS---------NSSSDLSSDYLLSHHIAKGLFTYAHLVMQPDGK-K---LDTIASVGSIVVTFNSHGDLS----SSTQLR--AEYRPVYLN--G------VLLSHPDL-LSDG----LLTIH : 114
PabFLA12   : ------------------------------------------------------------------------------------------------------------------------------------------------------------------------ :   -
PabFLA13   : ------------------------------------------------------------------------------------------------------------------------------------------------------------------------ :   -
PabFLA8    : ------------------------------------------------------------------------------------------------------------------------------------------------------------------------ :   -
PabFLA14   : ------------------------------------------------------------------------------------------------------------------------------------------------------------------------ :   -
PabFLA23   : ------------------------------------------------------------------------------------------------------------------------------------------------------------------------ :   -
PabFLA24   : --------------------------------TFLMPSDEKLAHAA----------------ASKVDLLMHHTIPRPLTFHDLLHFPTGS-F---IPSFLPDYMYSVANDGK-------------------NNFSLN--N------VKIVTPDL-CNGSP---AIQCH :  85
PpaFLA10   : SQYSEMVLYLDK-ADMLEELEREVLRQK--AITLFAPKNSHLEQLD-AD--LGRFLMRPGHEEYLRTVLRYHVIPGRVEGADFQNRTV--------ETLSKGDVVGL---R-----------------TYGLKRYVG--L------LRVFSPNS-IVRK----DGVIH : 121
PpaFLA11   : SQYSEMVLYLDK-ADMLEELEREVLRRG--AITVFAPKNSYLEQQVDAD--LWRFLMRPGHEAYLRTLLKHHVIPGRVEGADFQNRTV--------ETLASGNVVGL---R-----------------SHGLKRYVE--S------ARVFSPNS-IVRK----DGVIH : 122
PpaFLA6    : --------------------------------TLLAPNDNAFSNVLMN---------ST---TYLTTLLTYHGAAKVYSYEGLLNLPVGT-K---IPSTAANVVIVVTSNSK-------------------GAYKLD--D------SQIVDPDI-FVDN----TVAVH :  88
PpaFLA7    : --------------------------------TLFAPDDGAFSGLNLN---------SS---KLLMTTLDYHVATSVYNYNQLSTLPLNS-T---IKTSVPDVVILITSTGT-------------------SGLRLD--N------VAISDPDL-YVDS----QIAVH :  88
PpaFLA8    : --------------------------------TLFAPDDGAFSGLNMN---------SS---SLLMTTLDYHVATSVYNFNQLSNLPLNS-T---IKTSAPNVEIFVTSTGT-------------------DGLRLD--N------VAISDPDL-YADG----QMAVQ :  88
PpaFLA9    : --------------------------------TFFAPDDGAFSGLNMN---------NS---MLFMNTLHYHVATAVYSYQQLSYLPLNS-T---IQTAAPNVVMYITSTGE-------------------DGLMLD--N------VVISDPDL-YLDD----KVAVH :  88
PpaFLA1    : ------------------------------------------------------------------------------------------------------------------------------------------------------------------------ :   -
PpaFLA2    : ------------------------------------------------------------------------------------------------------------------------------------------------------------------------ :   -
PpaFLA3    : ------------------------------------------------------------------------------------------------------------------------------------------------------------------------ :   -
PpaFLA4    : AGLTSSADAIQA-AV----ISGSIT-LPSSNFTIFAPVNQAVDSILPS----------N---PDLSKVLNYHLATTDLPHKALLLLPIGQ-R---IPTLLPDNTLLVTDNNL-------------------ANYSIN--N------EQILYFDL-------------- : 104
PpaFLA5    : ------------------------------------------------------------------------------------------------------------------------------------------------------------------------ :   -
PpaFLA12   : --------------------------------TIFAPNDGALSDFHKRK--------TQ---EHLENLVRFHIITTPLPFSNLLRMEAGS-R---LKTAVSNFTILVTNTTK-------------------DAYQVD--D------ATIIDPDL-YTGA----TIAVH :  89
PtFLA1     : SHYTELAELVEK-ALLLQTLEDAV-GKH--NITIFAPKNEALERDLDPE--FKRFLLEPGNLKSLQTLLLYHIVPNRINPSHNSSLQH-------HSTLCRDRV---KLSS-----------------QESGEKLID--S------AKIIQVNA-VERP----DGVIH : 122
PtFLA19    : SHYTELAELVEK-ALLLQTLEDAV-GKH--NITIFAPRNEALERDLDPE--FKRFLLEPGNLKSLQTLLLYHIVPNRINLSHNSSLHH-------HSTLCRDRI---KLGS-----------------Q-SGEKLID--S------AKIIQVNA-VERP----DGVIH : 121
PtFLA6     : SHYTELAELVEK-ALLLQTLEEAV-GKH--NITIFAPRNEALERQLDPE--FKRFLLEPGNLKSLQTLVLFHIIPQRVGSNDWPGHKSNPSRHT---TLCNDHL---HLIT-----------------KNSGKKVVG--S------ADVTRPDD-VTRP----DGVIH : 126
PtFLA8     : SHYTELAELVEK-ALLLQTLEEAV-GKH--NITIFAPKNEALERQLDPE--FKRFLLEPGNLKSLQTLLLFHIIPQRVGSNDWPGHKSNPTRHT---TLCNDHL---HLIT-----------------KNSGKKLVG--A------AVLTRPDD-VTRP----DGVIH : 126
PtFLA5     : PSFSTFNHYLTL-TH----LAGEINSRN--TITVCAVDNAAMSELLSKH----------PSIATIKNILSLHVLLDYFGTKKLHQIREGT----------------------ALAATMFQATGSAPGSTGFVNITDVKGG----K-VAFGPEDNEG-NL----DVFYV : 119
PtFLA22    : PSLSTFNHYLTL-TH----LAGEINRRT--TITVCAVDNAAMSEILSKK----------PSISTIKNILSLHVLLDYFGTKKLHQIRDGT----------------------ALAATMFQATGSAPGSTGFVNITDVKGG----K-VAFGPKDNGG-NL----DVFYV : 119
PtFLA42    : --------------------------------TIFAPLDIAFARLGQL---------SV-------LDLQYHISPVRLSGYYLDSLPFGT-R---IPTLLPNHSLIVTTSLS----------------YFDGKLSIN--G------ISIEESAL-VDFG----SLIIF :  87
PtFLA51    : --------------------------------TFLMPKDRLLSKIR--M--------HQ---NAVSDFLLHHSIPSPLLFDHLRHIPPGS-L---IPSSDPDYMLNISNEGR-------------------KSFFLN--N------VKISSPDL-CTAGS---SIRCH :  88
PtFLA52    : --------------------------------TFFIPSDNSLSPTT------------T---SADPDIFPYHIVPQRLSFADLQQFKTFS-R---LPTLLFDKSILITNNSA-------------------SNFTLD--G------SRLTHPDI-YTNA----AITVH :  85
PtFLA37    : ------------------------------------------------------------------------------------------------------------------------------------------------------------------------ :   -
PtFLA17    : ------------------------------------------------------------------------------------------------------------------------------------------------------------------------ :   -
PtFLA53    : SHYTELAELVEK-ALLLQTLEDVV-GKH--NITIFAPKNEALERDLDPE--FKRFLLEPGNLKSLQTLLLYHIVPNRINPSHNSSLQH-------HRTLCRDRV---KLSS-----------------QESGEKLID--S------AKIIQVNA-VERP----DGVIH : 122
PtFLA41    : SHYTELAELVEK-ALLLQTLEDAV-GKH--NITIFAPKNEALERDLDPE--FQRFLLEPGNLKSLQTLLLYHIVPNRINPSHNSSLQH-------NSTLCRDRV---KLSS-----------------QESGEKLID--S------AKIIQVNA-VERP----DGVIH : 122
PtFLA38    : PDFSSFTSLITSIPS----LTSDLSDRS--ALTLLVIPNSYL-SSSLDLTRRLSP-------SALADLLRYHILLQYLSSSDLHQVPPSG----------------------ALITTLFQTTGRASSNSGSVNITRNPVT----NAITISSPSP-FSSS----NATVL : 123
PtFLA30    : PDFSSFTSLLASTPS----ITSDLSNRS--ALTLLVIPNSYL-SSSLDFTRRLSP-------SSLTDLLRYHILLQYLSSSDLHQLPPAG----------------------ALITTLFQTTGRASSNFGSVNITRNPVT----NAITISSPSP-FSSS----NATVL : 123
PtFLA20    : ------------------------------------------------------------------------------------------------------------------------------------------------------------------------ :   -
PtFLA23    : ------------------------------------------------------------------------------------------------------------------------------------------------------------------------ :   -
PtFLA18    : ------------------------------------------------------------------------------------------------------------------------------------------------------------------------ :   -
PtFLA10    : ------------------------------------------------------------------------------------------------------------------------------------------------------------------------ :   -
```

Type 1

```
PtFLA16     : PQFSTFNHYLTV-TH----LAAEINRRQ--TITVLALDNAAMSSLISKQ----------LSVYTLRNVLSLHVLVDYFGTRKLHQITNGT----------------------ELTATMFQATGSAPGASGYVNITDLNGG---K-VAFGAEDNDG-KL----NAVYV : 119
PtFLA34     : ----------------------------------------------------------------------------------------------------------------------------------------------------------------------- :   -
PtFLA9      : ----------------------------------------------------------------------------------------------------------------------------------------------------------------------- :   -
PtFLA12     : ----------------------------------------------------------------------------------------------------------------------------------------------------------------------- :   -
PtFLA7      : ----------------------------------------------------------------------------------------------------------------------------------------------------------------------- :   -
PtFLA2      : ----------------------------------------------------------------------------------------------------------------------------------------------------------------------- :   -
PtFLA13     : ----------------------------------------------------------------------------------------------------------------------------------------------------------------------- :   -
PtFLA47     : ----------------------------------------------------------------------------------------------------------------------------------------------------------------------- :   -
PtFLA40     : ----------------------------------------------------------------------------------------------------------------------------------------------------------------------- :   -
PtFLA28     : ----------------------------------------------------------------------------------------------------------------------------------------------------------------------- :   -
PtFLA39     : ----------------------------------------------------------------------------------------------------------------------------------------------------------------------- :   -
PtFLA50     : ----------------------------------------------------------------------------------------------------------------------------------------------------------------------- :   -
PtFLA29     : ----------------------------------------------------------------------------------------------------------------------------------------------------------------------- :   -
PtFLA32/49  : ----------------------------------------------------------------------------------------------------------------------------------------------------------------------- :   -
PtFLA45     : ----------------------------------------------------------------------------------------------------------------------------------------------------------------------- :   -
PtFLA35     : ----------------------------------------------------------------------------------------------------------------------------------------------------------------------- :   -
PtFLA46     : ----------------------------------------------------------------------------------------------------------------------------------------------------------------------- :   -
PtFLA3      : ----------------------------------------------------------------------------------------------------------------------------------------------------------------------- :   -
PtFLA15     : ----------------------------------------------------------------------------------------------------------------------------------------------------------------------- :   -
PtFLA14     : ----------------------------------------------------------------------------------------------------------------------------------------------------------------------- :   -
PtFLA48     : ----------------------------------------------------------------------------------------------------------------------------------------------------------------------- :   -
PtFLA27     : ----------------------------------------------------------------------------------------------------------------------------------------------------------------------- :   -
PtFLA43     : ----------------------------------------------------------------------------------------------------------------------------------------------------------------------- :   -
PtFLA33     : ----------------------------------------------------------------------------------------------------------------------------------------------------------------------- :   -
PtFLA21     : ----------------------------------------------------------------------------------------------------------------------------------------------------------------------- :   -
PtFLA26     : ----------------------------------------------------------------------------------------------------------------------------------------------------------------------- :   -
PtFLA4      : --------------------------------TIFAIKDSSLVNT-SL---------PP---WFLKNLLQYHTSPLKLSMEDVFKKPQGS-C---FPTLVDRKKLAVTKI-D---------------AKERLAEIN--H------VLVSHPDM-VLER----RITIH :  89
PtFLA44     : --------------------------------TLFAPSDSSLRTCFS---------------CSIPDLLHEHIVPGLFSIDYLRKLAFGT-K---IETLSPGRCITVTSTSLKNDS-------AT--PSTVKVFIG--G------VEITHPDL-FNNG----VLIIH :  94
PtFLA24     : --------------------------------TFFIPSDNSLSPTT------------T---SADPDIFPYHIVPQRLSFADLQQFKTFS-R---LPTLLFDKSILITNNSA------------------SNFTLD--G------SRLTHPDI-YTNA----AITVH :  85
SmoFLA1     : --------------------------------TLMIPTNEAIAAFVN-L--------NQ---SEIPSLLLYHAVTGVLPYDVLSEFPVGQ-R---LPTLLFGNQLVVTDNSA------------------AGYRLN--T------ARIVRPNM-CGNSTS--VVTCH :  90
SmoFLA2     : --------------------------------TLLIPSNNAIAGVDANL--------SQ---EDIINTLQYHVLTFPTSFEALSRNDVGA-E---LPTMLQGEMITVTSNSP------------------GNFTLN--E------VNITHPDV-CSSTR---FIACH :  90
SmoFLA3     : ----------------------------------------------------------------------------------------------------------------------------------------------------------------------- :   -
SmoFLA6     : DHLSTFNRYLSE-TG----VIKEVDSRS--SITLFIPPNAVLDPVIASVG----A----NNLLVIADILRYHCVLQYLEIKEIRSMTNAT--------------------GIVLATLYQTTGKPTGEYGFVNVSVSHGG------LIHLGVPG-AASN-----ATVV : 120
SmoFLA7     : PDFSTFSKLLTD-TG----VISEINQRS--SLTLLVPHNSALDAVLNRSSPSSTP----LPLPVVADVLRYHCLLQYLDVPQIKSMTNEL--------------------GMLVTSLYQTTGRAAGQNGFVNVSVSSDG------QIHAGLPF-AATS----LATVR : 125
SmoFLA8     : -NFSTFSRLLSM-TN----VTAEINSRS--SLTILVPDNSILDSYVGA---NLEG----MHVWAVADFCRYHVLLQYLDTQEIMQMTNQS--------------------GLLITTLYQTTGRAEGIDGFVNMTFGNDI------QFGLPPPH-ASGP----VATVL : 121
SmoFLA9     : ---TEMMELVEQ-AGMLEALELAA-DRH--NLTIFAPRDEFLELHFDAD--FRRFLLLPGNVRFLQELVMFHVLPIRITASQWRSGRF--------QSLSGSRD-EVLLHW----------------NKKQRLAVD--R------STVDWPDM-IVRS----DGVVH : 120
SmoFLA4     : ----------------------------------------------------------------------------------------------------------------------------------------------------------------------- :   -
SmoFLA5     : ----------------------------------------------------------------------------------------------------------------------------------------------------------------------- :   -
BdiFLA23    : SHYTELSELVEK-ALLLQTLEDAV-GRH--NVTIFAPRNEALERDLDPE--FKRFLLEPRNLKSLQSLLLFHVLPSRHPAASWPA---SAA----HPTLSGEDL-ELAAG-----------------SNGSMRV-A--H------ASVTRPDA-VLRP----DGVIH : 122
BdiFLA22    : SRYTELSELVEK-ALLLQQLEDAV-GRG--NVTIFAPRNEALERDLDPE--FRAFLLQPRNLRSLQRLLLFHVLPSRLSSSQWRSA----A----SLTLSGEHL-ELSLSD----------------SSTTAMLAG--S------AAVTHPDA-VVRP----DGVIH : 123
BdiFLA9     : ----------------------------------------------------------------------------------------------------------------------------------------------------------------------- :   -
BdiFLA1     : ----------------------------------------------------------------------------------------------------------------------------------------------------------------------- :   -
BdiFLA2     : --------------------------------TFLMPSDRLLSTAS--V--------PG---NQVLDLLLRHSVPAVLMFADLNRLPNGT-V---VPTRHTNQMVTITKREH------------------RQLYFN--N------IELTSPDI-CRGGD---SFRCH :  88
BdiFLA20    : PNFSDFLRLLTS-SP----VAGELTGRS--SLTLLAVPNANLPRSPSAF-AAASG-------ADLADVLRYHVLLEYLSPSDLARLPASG---------------------KLVTTLFQTTGRAPSDLGAVNLTSSAAGGHSSTVVVHSPAPS-FPGS----NATVL : 126
BdiFLA10    : ----------------------------------------------------------------------------------------------------------------------------------------------------------------------- :   -
BdiFLA11    : ----------------------------------------------------------------------------------------------------------------------------------------------------------------------- :   -
BdiFLA3     : ----------------------------------------------------------------------------------------------------------------------------------------------------------------------- :   -
BdiFLA12    : ----------------------------------------------------------------------------------------------------------------------------------------------------------------------- :   -
BdiFLA8     : ----------------------------------------------------------------------------------------------------------------------------------------------------------------------- :   -
BdiFLA13    : ----------------------------------------------------------------------------------------------------------------------------------------------------------------------- :   -
BdiFLA4     : ----------------------------------------------------------------------------------------------------------------------------------------------------------------------- :   -
BdiFLA14    : ----------------------------------------------------------------------------------------------------------------------------------------------------------------------- :   -
BdiFLA15    : ----------------------------------------------------------------------------------------------------------------------------------------------------------------------- :   -
BdiFLA16    : ----------------------------------------------------------------------------------------------------------------------------------------------------------------------- :   -
BdiFLA17    : ----------------------------------------------------------------------------------------------------------------------------------------------------------------------- :   -
BdiFLA21.1  : ----------------------------------------------------------------------------------------------------------------------------------------------------------------------- :   -
BdiFLA21.2  : ----------------------------------------------------------------------------------------------------------------------------------------------------------------------- :   -
BdiFLA5     : ----------------------------------------------------------------------------------------------------------------------------------------------------------------------- :   -
BdiFLA18    : ----------------------------------------------------------------------------------------------------------------------------------------------------------------------- :   -
BdiFLA6     : ----------------------------------------------------------------------------------------------------------------------------------------------------------------------- :   -
BdiFLA19    : ----------------------------------------------------------------------------------------------------------------------------------------------------------------------- :   -
BdiFLA7     : ----------------------------------------------------------------------------------------------------------------------------------------------------------------------- :   -
MpoFLA6     : PDFSIFNQLLTD-TR----VAGEINRRQ--GVTVLAPSNEAMTDFRSAN-PALEG-------DRVANLLRYHVLLDFLTIQNLQRLDIAN--------------------YTRVGTLFRTTGPANADDASVNLYNTPSN------IFIGSSAV-DSSQ----NSTVI : 121
MpoFLA1     : ----------------------------------------------------------------------------------------------------------------------------------------------------------------------- :   -
MpoFLA14    : SQYSEWMELVEQ-AAMLHPLEELVGSGR--KLTIFAPQNHQLQ----PK--IKSFLLKPSNAIALKRVVQYHVLSERVTASEWPTASF--------TTLASEAV-WL---S----------------TQGPILMAG--D------VAVAAPDA-IIRP----DGVVH : 117
MpoFLA4     : --------------------------------TLFVPTNEALSSLSM-----------V---TPVLNILLYHAATPQYTFDQLVTLPVGT-R---LQSFLANESVLITNNAR------------------NNFQVD--N------VRIVLANV-CASNTTDLQISCH :  90
MpoFLA7     : PRFSVLSDLLVS-SG----VEKEINSRT--SITLLAPADAVLTAFQASV-PNADT-------VKIADLLRYHVLLQYFDMTELKGLGTVN--------------------YSSVTTLLQTTGRANEQDGFVNIYNTATQ------ILIGPAAS-ASAS----NATVV : 121
MpoFLA2     : ----------------------------------------------------------------------------------------------------------------------------------------------------------------------- :   -
MpoFLA3     : ----------------------------------------------------------------------------------------------------------------------------------------------------------------------- :   -
MpoFLA5     : --------------------------------TWLLPNDEAFSGTSY-----------P---KNVTKFIDYHVIRQLLPYSRLSTLSVGT-R---LPTFLGSETVVVTSNLR------------------FNYSLD--N------AMIVVPDL-YSDS----TVAVH :  86
MpoFLA8     : PRFSVFREMLSS-HG----VAAEINGRK--SVTLLAPANNVMRAFRTSN-NKTDA-------IKITDLLRFHVLLTYFDMAELRALKTTN--------------------YTSVTTLLQTTGRASGNNGFVNIYNLKSR------VKLGPSVP-DSSS----NVTVL : 121
```

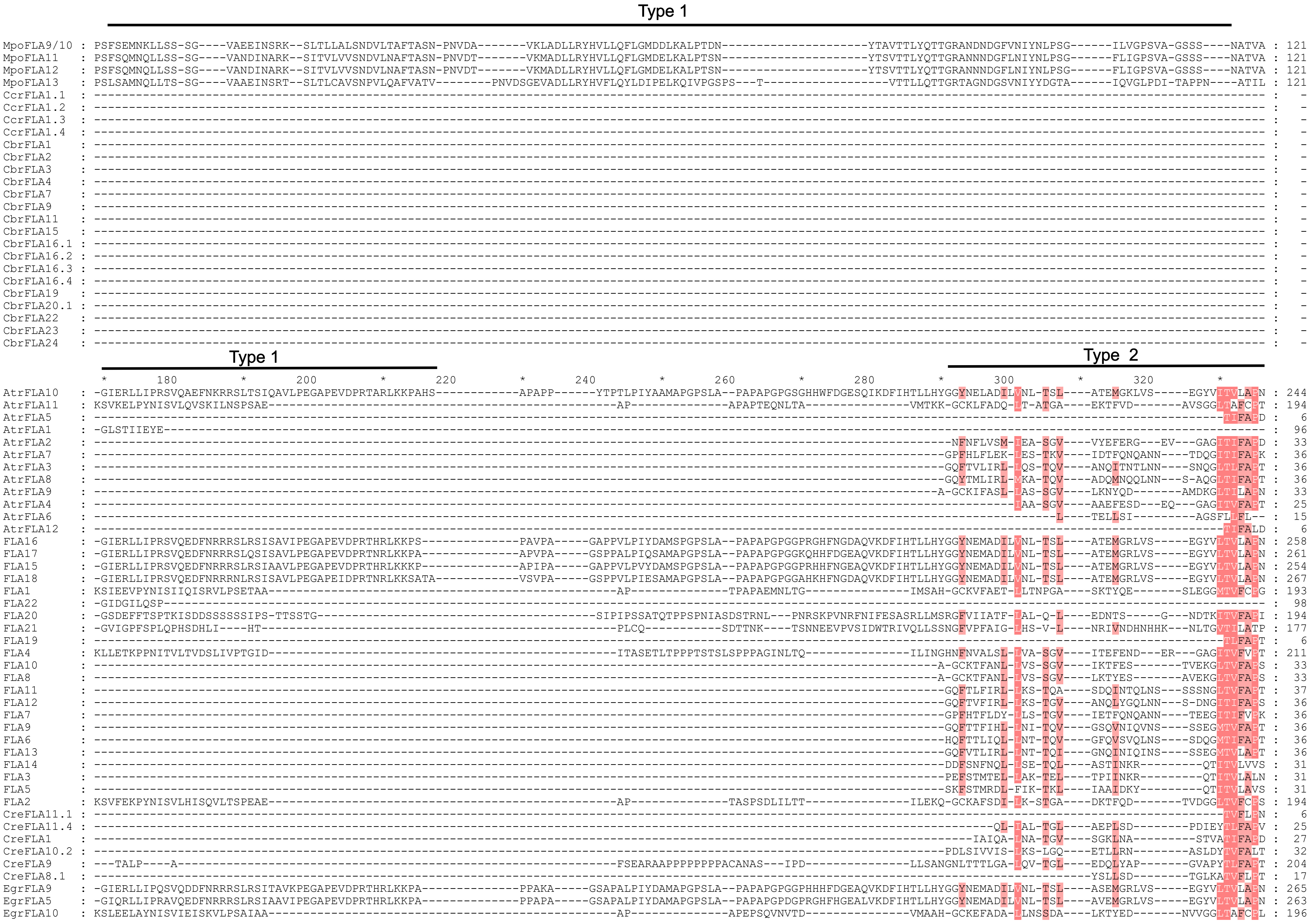
Type 1
Type 1
Type 2

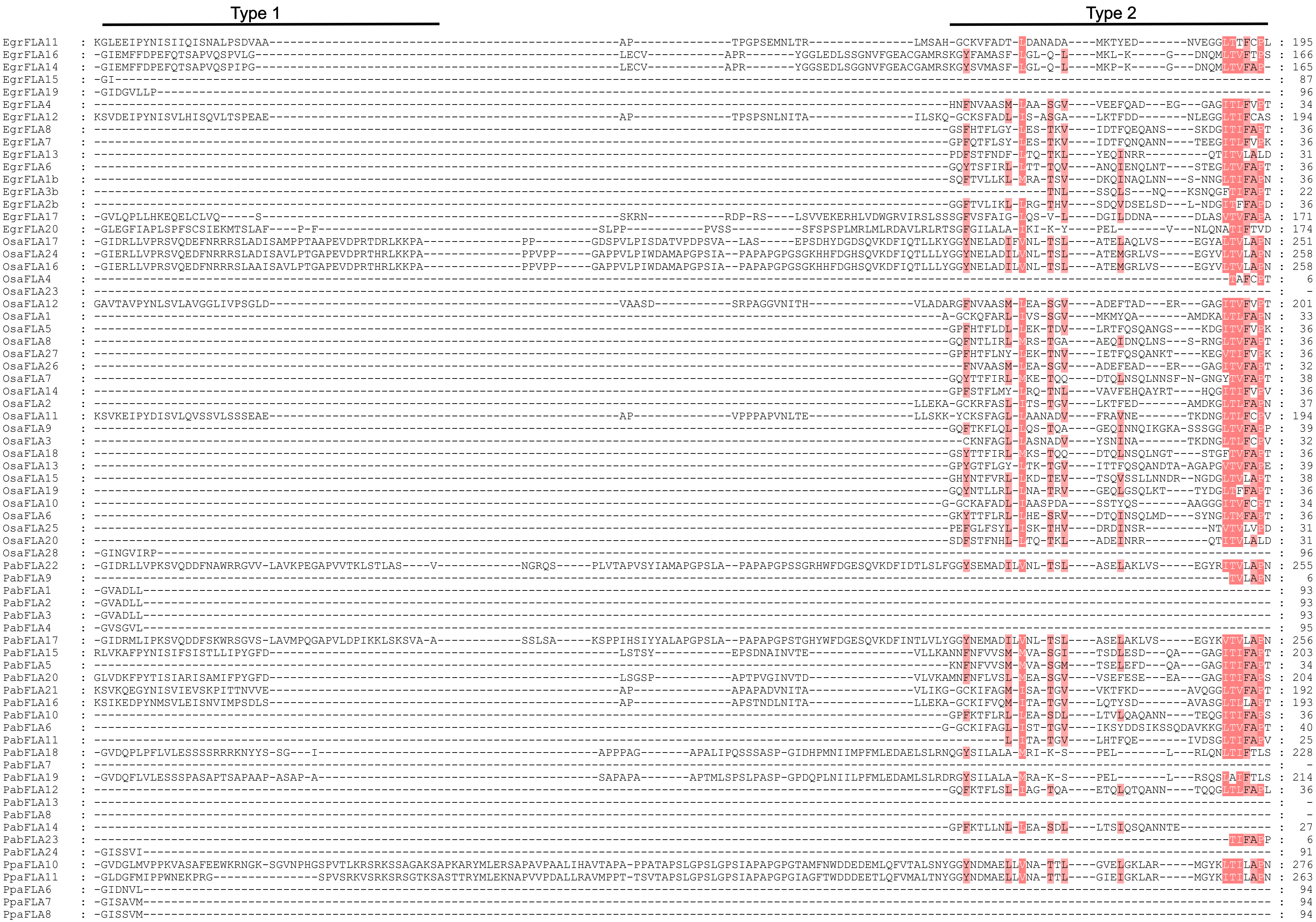
Type 1
Type 2

```
                   Type 1                                                                                                  Type 2
PpaFLA9    : -GISMVM----------------------------------------------------------------------------------------------------------------------------------------------------------------- :  94
PpaFLA1    : --------------------------------------------------------------------------------------------------------------------------PSLSILADV-IEA-SGL----AEQLSM---------TDNVTVLAPD :  31
PpaFLA2    : --------------------------------------------------------------------------------------------------------------------------PRLQQLAAV-IEA-AGL----ADTVER---------LVNVTVIAPD :  31
PpaFLA3    : --------------------------------------------------------------------------------------------------------------------------PEYSTFKSL-MES-TAV----LSEVQTR---------SSLTILCPP :  31
PpaFLA4    : ------------------------------------------------------------------------------------------------------------------------------------------------------------------------ :   -
PpaFLA5    : --------------------------------------------------------------------------------------------------------------------------PQFSMQKDA-LIS-SGV----ADALKGM---------NTLTLLLIN :  31
PpaFLA12   : -GINAV------------------------------------------------------------------------------------------------------------------------------------------------------------------ :  94
PtFLA1     : -GIERLLIPRSVQQDFNNRRSLQSISAVKPEGAPEVDPRTHRLKKPA--------------PPAKP-----GSAPVLPIYDAMAPGPSLA--PAPAPGPGGPHHHFNGEKQVKDFIETLLLYGGYNEMADILVNL-TSL----ATEMGRLVS-----EGYVLTVLAPN : 258
PtFLA19    : -GIERLLIPRSVQQDFNNRRSLQSISAVKPEGAPEVDPRTQRLKKPA--------------PPAKP-----GSAPVLPIYDAMAPGPSLA--PAPAPGPGGPHHHFNGERQVKDFIETLLLYGGYNEMADILVNL-TSL----ATEMGRLVS-----EGYVLTVLAPN : 257
PtFLA6     : -GIERLLVPQSVQEDFNRRRNLRSISAVLPEGAPEVDPRTHRLKKPE--------------PPVRA-----GSPPVLPIYDAMAPGPSLA--PAPAPGPGGPHHHFDGESQVKDFIQTLLLYGGYNEMADILVNL-TSL----ATEMGRLVS-----EGYVLTVLAPN : 262
PtFLA8     : -GIERLLVPQSVQEDFNRRRNLRSISAVLPEGAPEVDPRTHRLKKPE--------------PPVRA-----GSPPVLPVYDAMSPGPSLA--PAPAPGPGGPHHHFDGESQVKDFIQTLVHYGGYNEMADILVNL-TSL----ATEMGRLVS-----EGYVLTVLAPN : 262
PtFLA5     : KSLEEIPYNISVIQISKVLPSDVAA--------------------------------------------------AP--------------TPEPSAMNITD---------------IMSAH-GCKVFADT-LIANPEA----SKTYQD-------SVDGGLTVFCPL : 195
PtFLA22    : KSVEEIPYNISVIQISKLLPSDVAA--------------------------------------------------AP--------------TPEPSAMNITD---------------IMSAH-GCKVFADT-LIANPDA----SKTYQD-------TIDGGLTVFCPL : 195
PtFLA42    : -GMSEFFNSSLEISPNLTPAPAP----------------------------------------------------SPSP-----------VTSLGNT---SQNESTGLDVDFFGQASHLLMPRGYSIMGTF-LDA-Q-L----FGI-K---------NQTRLTIFAPV : 171
PtFLA51    : -GIDGVLL---------------------------------------------------------------------------------------------------------------------------------------------------------------- :  95
PtFLA52    : -CIDNLL----------------------------------------------------------------------------------------------------------------------------------------------------------------- :  91
PtFLA37    : ------------------------------------------------------------------------------------------------------------------------------------------------------------------TLFCPP :   6
PtFLA17    : -------------------------------------------------------------------------------------------------------------------------A-GCKTFASL-LQT-SGV----IKTYQS-------AADKGLTIFAPN :  33
PtFLA53    : -GIERLLIP--------------------------------------------------------------------------------------------------------------------------------------------------------------- : 130
PtFLA41    : -GIKRLLIP--------------------------------------------------------------------------------------------------------------------------------------------------------------- : 130
PtFLA38    : SLIMTLPYNVSIISVNSLLVPYGFD--------------------------------------------------LMASE-----------TRPTLGLNITK---------------ALLDGHSFLVAASL-LSA-SGV----VQEFEAD---EG---GAGITLFVPT : 203
PtFLA30    : SLIKTLPYNVSIISVNSLLVPYGFD--------------------------------------------------LMASE-----------TRPTLGLNITK---------------ALLDGRSFFVAASL-LSA-SGV----VEEFEAD---EG---GAGITLFVPT : 203
PtFLA20    : --------------------------------------------------------------------------------------------------------------------------GHFKTFIRL-LKS-TQL----DSNLNSQLGN----TNNGLTIFAPS :  36
PtFLA23    : --------------------------------------------------------------------------------------------------------------------------GQFSVFIRL-LKA-TQE----DVTLNGQLNN----TNNAITIFAPS :  36
PtFLA18    : --------------------------------------------------------------------------------------------------------------------------GPFHNFLNY-LES-TKV----IDTFQNQANN----TDEGITIFVPK :  36
PtFLA10    : --------------------------------------------------------------------------------------------------------------------------GQFSVFIRL-LKA-TQE----DVTLNGQLNN----TNNAITIFAPS :  36
PtFLA16    : KSLEEIPYNISILQISQPLNSAEAE--------------------------------------------------AP--------------TAAPT-LNVTA---------------ILSNQ-GCKAFSDL-LI-ASGA----HTTFEE-------NVDGGLTVFCPT : 193
PtFLA34    : --------------------------------------------------------------------------------------------------------------------------GHFTVFVRL-MQA-TTE----DTELNKELNK----TNNGITIFAPS :  36
PtFLA9     : --------------------------------------------------------------------------------------------------------------------------GQFTTLIRL-LKS-TQE----ADQINTQLNN---S-NQGLTVFAPT :  36
PtFLA12    : --------------------------------------------------------------------------------------------------------------------------GPFHTFLSY-LES-TKV----VDTFQNQANN----TDEGITIFVPK :  36
PtFLA7     : --------------------------------------------------------------------------------------------------------------------------GQFTTLIRL-MKS-TQE----ADQINTQLNN---S-NQGLTVFAPP :  36
PtFLA2     : --------------------------------------------------------------------------------------------------------------------------GHFTVFARL-MQA-TTE----DTELNKELNT----TNNGITILAPT :  36
PtFLA13    : --------------------------------------------------------------------------------------------------------------------------GHFTIFIRL-LRS-TQE----ENHLFSALND----SSTGLTIFAPT :  36
PtFLA47    : --------------------------------------------------------------------------------------------------------------------------GHFTIFIRL-LRS-TQE----ENHLFSALND----SSSGVTIFAPT :  36
PtFLA40    : --------------------------------------------------------------------------------------------------------------------------GHFTIFIRL-LRS-TQE----ENHLFSALND----SSSGVTIFAPT :  36
PtFLA28    : --------------------------------------------------------------------------------------------------------------------------GHFTIFIRL-LRS-TQE----ENHLFSALND----SSSGVTIFAPT :  36
PtFLA39    : --------------------------------------------------------------------------------------------------------------------------GHFTIFIRL-LRS-TQE----ENHLFSALND----SNTGITIFAPT :  36
PtFLA50    : --------------------------------------------------------------------------------------------------------------------------GHFTIFIRL-LRS-TQE----ENHLFSALND----SSTGLTIFAPT :  36
PtFLA29    : --------------------------------------------------------------------------------------------------------------------------GHFTIFIRL-LRS-TQE----ENHLFSALND----SSTGLTIFAPT :  36
PtFLA32/49 : --------------------------------------------------------------------------------------------------------------------------GHFTIFIRL-LRS-TQE----ESHLFSALND----SSTGLTIFAPT :  36
PtFLA45    : --------------------------------------------------------------------------------------------------------------------------GHFTIFIRL-LRS-TQE----ENHLFSALND----SSTGLTIFAPT :  36
PtFLA35    : --------------------------------------------------------------------------------------------------------------------------GHFTIFIRL-LRS-IQE----ENHLFSALND----SSTGLTIFAPT :  36
PtFLA46    : --------------------------------------------------------------------------------------------------------------------------GRFLSFVRL-MKA-THV----DTQLFSQLNS----STDGITMFAPN :  36
PtFLA3     : --------------------------------------------------------------------------------------------------------------------------GQFATLIRL-LNN-TQT----LNQIENQLNS----SSEGMTIFAPT :  36
PtFLA15    : --------------------------------------------------------------------------------------------------------------------------GQFVTFISL-LNK-TQT----FNQIENQINS----SSEGMTIFAPT :  36
PtFLA14    : ------------------------------------------------------------------------------------------------------------------------------------------------NHLFSALND----SSTGLTIFAPT :  20
PtFLA48    : --------------------------------------------------------------------------------------------------------------------------GHFAFFTRL-IKS-TQE----DIQLFSQLND----SRDGVTVFAPT :  36
PtFLA27    : --------------------------------------------------------------------------------------------------------------------------GGFAVFIRL-IKS-TQE----DIQVFSQLND----SRDGVTIFAPT :  36
PtFLA43    : --------------------------------------------------------------------------------------------------------------------------GHFTIFIRL-LRS-TQE----ENHLFSALND----SSPGLTIFAPT :  36
PtFLA33    : --------------------------------------------------------------------------------------------------------------------------GHFTIFIRL-LRS-TQD----ENRLFSALND----SSTGLTIFAPT :  36
PtFLA21    : --------------------------------------------------------------------------------------------------------------------------PDFSTFSSY-LTQ-TQL----AGEINSR---------QTITVLVVE :  31
PtFLA26    : --------------------------------------------------------------------------------------------------------------------------PEFANFNDL-LSQ-SGL----AQEMNSR---------QTITVLVLD :  31
PtFLA4     : -GVLAPFSSLRSKDVYFGWESIQA---------------------------------------------------PICD-----------ANS-SL----VSDANGPRIILEWTRIIHLLSSHRFVSFAIG-LNS-V-L----DRILADHK------NLSSVTIFAPP : 176
PtFLA44    : -GIQGYIAPLSPFSCDFERLSSLSF----P-F----------------------------------------QEGV-----------TPHVTSTTHQQGIGTLVQPAIMRLMLRDAMLRLRSNGFTILSLA-MRV-K-Y----PEL-------T---NLVNMTVFALD : 188
PtFLA24    : -CIDNLL----------------------------------------------------------------------------------------------------------------------------------------------------------------- :  91
SmoFLA1    : -GIDRVLNP--------------------------------------------------------------------------------------------------------------------------------------------------------------- :  98
SmoFLA2    : -GIDRVL----------------------------------------------------------------------------------------------------------------------------------------------------------------- :  96
SmoFLA3    : --------------------------------------------------------------------------------------------------------------------------GGYNEMAELLVNL-TSF----AWDMAKLVN-----EGHRLTLLAPN :  36
SmoFLA6    : KNVTQHPYDMSYMELDHPLMPMDLG--------------------------------------------------IDQGA-------------APSVNNLTA---------------ALKAKGNFNTFLGL-LAS-TGV----DKALRS---------MSAVTLLAPD : 195
SmoFLA7    : TNITQFPYNISYMQIDQALVPEDLG--------------------------------------------------RPQAA-------------ASSILNLTN---------------TLQRSGKFTTFLNL-MQS-TGF----TAALAT---------LPAFSLFVPT : 200
SmoFLA8    : NNFVRYPYNMSFMMISKPLMPVRLA--------------------------------------------------QMNRP-------------D-HFPSISN---------------ALFKGNIYKTFQKL-MQD-TGT----LAAAEDI---EHQPFTSGVTVFAPT : 201
SmoFLA9    : -RIDGLLVPKSVQDAYAAARISSS--AVLPQAAPSLSGESFGNKAEAK----------------------------LFHVTFPVIDDLIG--SAPAPAPSPSRFSADGHGRVQDFIAALVSIGGYGEIADLLVNL-TSL----SVEIANLVN-----EGHALTVLAPG : 245
SmoFLA4    : --------------------------------------------------------------------------------------------------------------------------PNFKRFNTL-LTT-SGV----VDQVNPM---------LTATLFIPE :  31
SmoFLA5    : --------------------------------------------------------------------------------------------------------------------------PDYTNLNML-LLQ-TGV----ANEINQR---------SSLTILAAE :  31
BdiFLA23   : -GIERLLVPRSVQEDFNRRRSLAAISAVLPTGAPEVDPRTHRLKKPA--------------PPVLP-----GAPPVLPVWDAMAPGPSIA--PAPAPGPNSGKLHFDGHSQVKDFIQTLVLYGGYNELADILVNL-TSL----ATEMGRLVS-----EGYVLTVLAPN : 258
BdiFLA22   : -GIEKLLIPRSVQEDFNRRRSLAAISAVLPTGAPEVDPRTHRLKKPA--------------PPVLP-----GAPPVLPVWDAMAPGPSIA--PAPAPGPNSGKLHFDGHSQVKDFIQTLVLYGGYNELADILVNL-TSL----ATEMGRLVS-----EGYVLTVLAPN : 259
BdiFLA9    : ------------------------------------------------------------------------------------------------------------------------------------------------------------------TLFCPV :   6
BdiFLA1    : ------------------------------------------------------------------------------------------------------------------------------------------------------------------TILAPT :   6
BdiFLA2    : -GINGVLRP--------------------------------------------------------------------------------------------------------------------------------------------------------------- :  96
BdiFLA20   : GAVTREPYNISVLAVGGLIVPSGFD--------------------------------------------------IAASG-----------SESRPAVNITR---------------VLADARGFNVAASM-LEA-SGV----ADDFEAD---ER---GAGITVFVPT : 206
BdiFLA10   : ---------------------------------------------------------------------------------------------------------------------------GFNVAASM-LEA-SGV----AEEFEDD---ER---GAGITVFVPT :  33
```

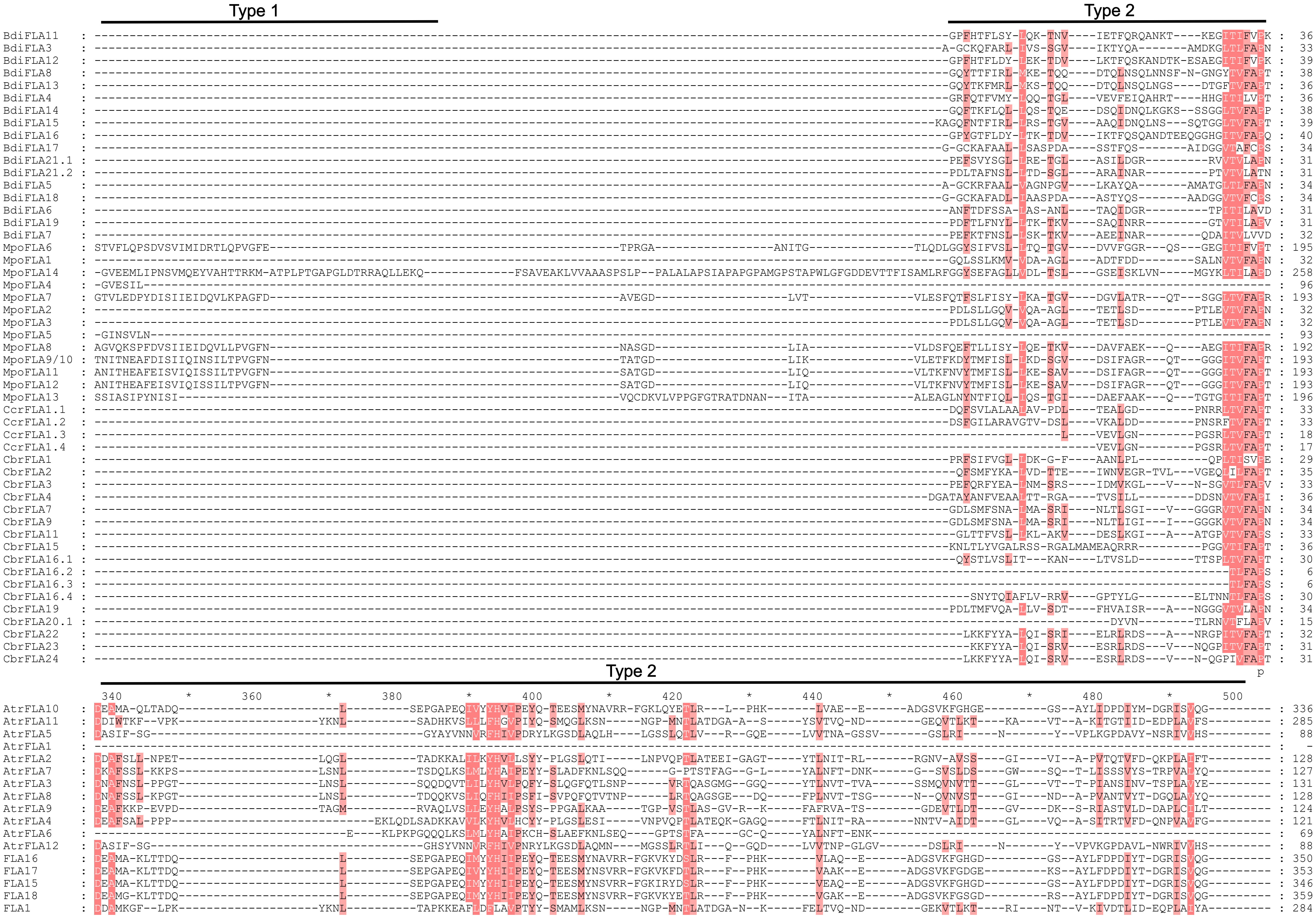
Type 1
Type 2
Type 2

Type 2

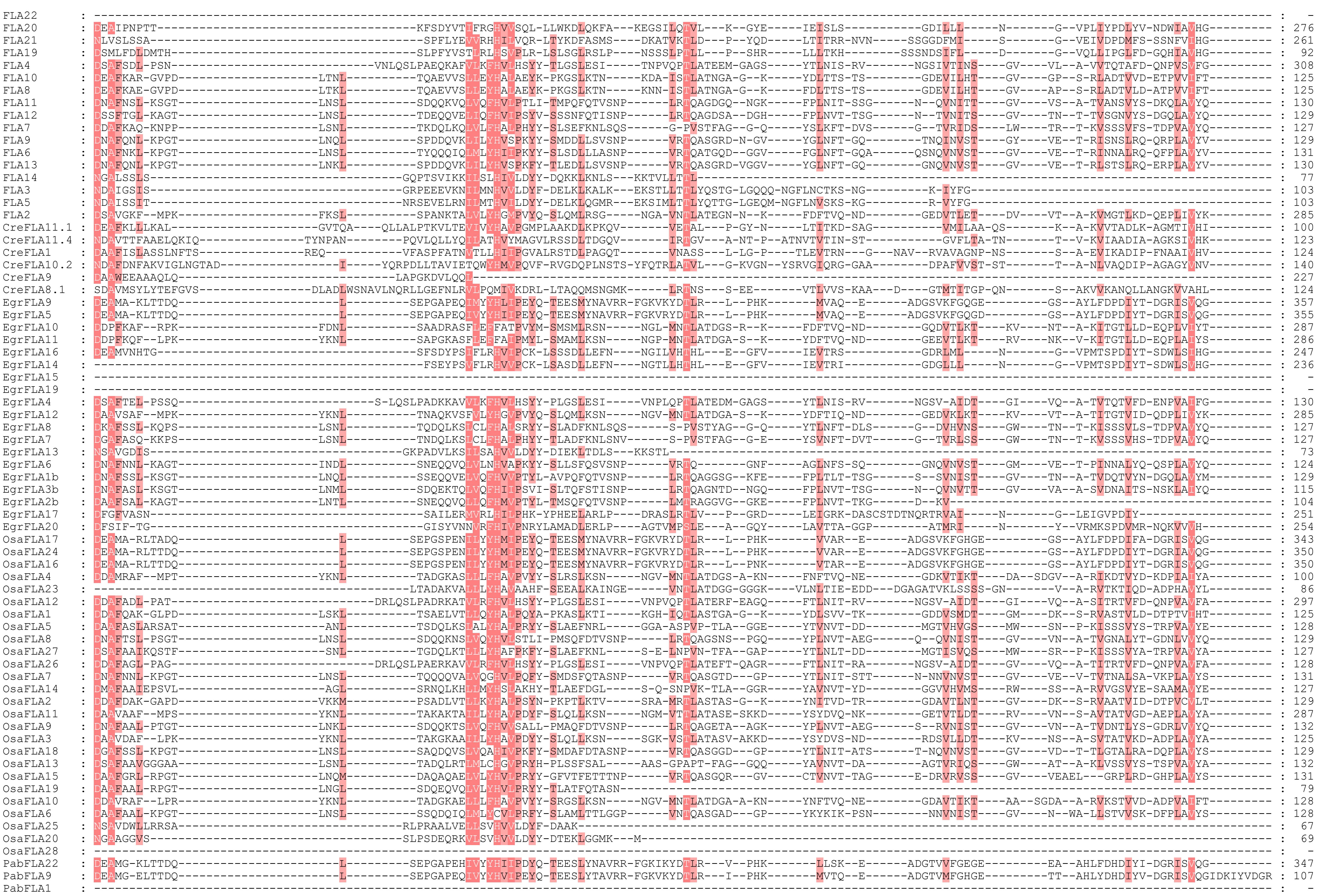
FLA22 : -
FLA20 : 276
FLA21 : 261
FLA19 : 92
FLA4 : 308
FLA10 : 125
FLA8 : 125
FLA11 : 130
FLA12 : 129
FLA7 : 127
FLA9 : 129
FLA6 : 131
FLA13 : 130
FLA14 : 77
FLA3 : 103
FLA5 : 103
FLA2 : 285
CreFLA11.1 : 100
CreFLA11.4 : 123
CreFLA1 : 124
CreFLA10.2 : 140
CreFLA9 : 227
CreFLA8.1 : 124
EgrFLA9 : 357
EgrFLA5 : 355
EgrFLA10 : 287
EgrFLA11 : 286
EgrFLA16 : 247
EgrFLA14 : 236
EgrFLA15 : -
EgrFLA19 : -
EgrFLA4 : 130
EgrFLA12 : 285
EgrFLA8 : 127
EgrFLA7 : 127
EgrFLA13 : 73
EgrFLA6 : 124
EgrFLA1b : 129
EgrFLA3b : 115
EgrFLA2b : 104
EgrFLA17 : 251
EgrFLA20 : 254
OsaFLA17 : 343
OsaFLA24 : 350
OsaFLA16 : 350
OsaFLA4 : 100
OsaFLA23 : 86
OsaFLA12 : 297
OsaFLA1 : 125
OsaFLA5 : 128
OsaFLA8 : 129
OsaFLA27 : 127
OsaFLA26 : 128
OsaFLA7 : 131
OsaFLA14 : 127
OsaFLA2 : 129
OsaFLA11 : 287
OsaFLA9 : 132
OsaFLA3 : 125
OsaFLA18 : 129
OsaFLA13 : 132
OsaFLA15 : 131
OsaFLA19 : 79
OsaFLA10 : 128
OsaFLA6 : 128
OsaFLA25 : 67
OsaFLA20 : 69
OsaFLA28 : -
PabFLA22 : 347
PabFLA9 : 107
PabFLA1 : -

Type 2

```
PabFLA2    : ------------------------------------------------------------------------------------------------------------------------------------------------------------------------ :   -
PabFLA3    : ------------------------------------------------------------------------------------------------------------------------------------------------------------------------ :   -
PabFLA4    : ------------------------------------------------------------------------------------------------------------------------------------------------------------------------ :   -
PabFLA17   : DEAMG-ELTTEQ-----------------------L---------SEPGAPEHIMYYHIIPEYQ-TEESLYN------------------------------------------------------------------------------------------------ : 294
PabFLA15   : DDAFSAL-PPDT--------------------LQGL----------TAENKAVVLKYHVLHSYY-PLGSLDSI-----VNPLQPTLATESM-GAGT-----YTLNIT-RV-------NGSV-AVDT----GI----VQ--A-SITQTVFD-QKPLAIFA--------- : 298
PabFLA5    : DDSFSAL-PPHT--------------------LQGLTARNKALQGLTARNKAVVLKYHVLHSYY-PLGSLESI-----VNPVQPTLATESM-GAET-----YTLNIT-RV-------NGSV-AVDT----GI----VQ--A-SITQTVFD-QKPLAVFA--------- : 139
PabFLA20   : DDAFSAL-NPET--------------------LQGL----------TAEKKAVILKYHVLLSYY-PLGSLQTI-----VNPAQPTLATQAM-GAGS-----YTLNIT-RM-------DGMV-AVNT----GI----VQ--A--------------------------- : 283
PabFLA21   : DASFTGV-TSKL--------------------LSKL----------SSEDKVSLLEFHALPMYS-PLGYLKTS-----NGP-ISTMATTGA-G--K-----YVMTAS-SS--------GDTVMLNT----RI----SK--A-TISDTLLD-DQPLAIFT--------- : 284
PabFLA16   : DGAFSGT-VMLK--------------------LKKL----------SSAQEVSLLEYHAVPAYN-PVGTLKTT-----IAP-ISTLATNGA-S--K-----YALSVS-SA--------GDTVILNT----GL----SK--S-TISSTILD-DQPVVLYT--------- : 285
PabFLA10   : DTAFSSL-PKKL--------------------LANL----------TADEIKEVLLAHCISKFY-TLTDFQDFSNP------ANTMATGSN--GGK-----YNLNIT-GDG------G--TVTVSS----GY----VT--T-PIISTVHV-TDPLALYT--------- : 129
PabFLA6    : DGAFTDV-IKKL--------------------LKKL----------SSEDKVSLLKFHAVPSYT-PLGTLKNT-----NGP-VTTMAS-------N-----YAVTVS-SS--------GNTVTLNT----SL----SK--A-TISSTLLD-NLPLAIFT--------- : 128
PabFLA11   : DGAFDSV-MT-E--------------------IKKL----------SRAQQVSILEYHARPVYS-PLGTLKTT-----DGDVVTTMATNGA-E--R-----YSLTVG-VN--------GDNVILGT----GV----NS--A-VITSTLLD-DQPLVVFS--------- : 117
PabFLA18   : DDSVFLKG---------------------------------------GQDFTNTVRYHVVPNRRLLLADLVRLP----AGTRLHTLL---H--GQS-----LLVTDN-------------KPFSI-----N-------H--VGVKIPDAFT-NRWIAVHE--------- : 307
PabFLA7    : ------------------------------------------------------------------------------------------------------------------------------------------------------------------------ :   -
PabFLA19   : DDSVFLKD---------------------------------------GQDFVDNVTYHVVPNRRLLLADLMHLP----AGTVLHTLL---D--GQS-----LVVTNS------------KPFSV-----N-------H--VGVKIPDAFT-NRWIAV----------- : 291
PabFLA12   : DGAFSSL-RPQY--------------------KAML----------SKLTDQ-----------------APTLSNP------LSTM--------GS-----YKFNVS-AFG--------AQVNVST----GL----VN--A-PLTSSIFS-QAPVAVYE--------- : 107
PabFLA13   : ----------------------------------------------AKLDKIALLQYHALPSYY-TFGQFQTVSNP------VRTMASGNG---GP-----FGVNVT-AVG------N--SVNIST----GL----VN--T-PVNSAVYS-QNPVAVYQ--------- :  77
PabFLA8    : --------VKNE--------------------LKKL----------SSEDKESLLEFHALPIYS-PRTTLKFS-----KRP-IATMASTGP-G--K-----YDIKVS-SA--------GDTVILNT----GV----DK--A-TITSTLLD-DVPMVILT--------- :  85
PabFLA14   : -QERSVVGPL----------------------HSQV----------LPDEKKEVLLAHCIPKFY-TLTDFQNF-----SNP-ANTMATGSN--GGK-----YNLNIT-AM--------SGAVTVSS----GY----VS--T-AITGSVRV-TDPVAVYT--------- : 118
PabFLA23   : DLALLPVVG--------------------------------------SPALPHHVLCHVAAQR-LSYGDLVNKT----SSDRIPSLA---L--GHP-----IVVTTG-PLTN--QGTGKYVVLN-----G-------R--ARVVSPDLYG-DANLIVHG--------- :  94
PabFLA24   : ------------------------------------------------------------------------------------------------------------------------------------------------------------------------ :   -
PpaFLA10   : DQAMQ-LLTTEQ-----------------------L---------NQAM--EPLLYYHFLSEYQ-TEESMYNAVKR-LGKQSYSTLR---H--PHK-------VVAS--E------SDGTVKFGDGD---------DA--AHIFDHDIYV-DGHISIQGINR------ : 369
PpaFLA11   : DQAMQ-QLTIEQ-----------------------L---------DMPM--EPLLYYHFLSEYQ-TDESMYNAVKR-LGKQSYSTLR---H--PHK-------VIAS--E------SDGTVKFGDGD---------GA--AHICDHDIYV-EGHISVQGINR------ : 356
PpaFLA6    : ------------------------------------------------------------------------------------------------------------------------------------------------------------------------ :   -
PpaFLA7    : ------------------------------------------------------------------------------------------------------------------------------------------------------------------------ :   -
PpaFLA8    : ------------------------------------------------------------------------------------------------------------------------------------------------------------------------ :   -
PpaFLA9    : ------------------------------------------------------------------------------------------------------------------------------------------------------------------------ :   -
PpaFLA1    : NNAFNGTGGLLSILA---------------ANNLTLE----QVTAPGSNRAASILLYHIVPSPA-TAADLTDGQT-------LTTAL---G--K-A-----YELTVD-KTA---TPT--VVVSFIGA-GS-------N--A-TVITADLR-VCNSVVHI--------- : 134
PpaFLA2    : NNAFNGTNGLLSLLR---------------QNNLNLT----DVTAPGQNRAASILLYHIIAGPA-RAADLKNNQV-------LTTFL---G--P-N-----YTLRVN-KQT---APT--LIVSFVGT-GS-------N--A-TVTAADVP-VCSSIVHV--------- : 134
PpaFLA3    : NSVLDPFIAARR--------------------------------SFSTQMLADVIRYHVLLQYL-DSAELRTQT---NNGGMFTTLYQTTG-RANEL-DGFVNITVT-PS----------NAVA-------------------------------------------- : 106
PpaFLA4    : ------------------------------------------------------------------------------------------------------------------------------------------------------------------------ :   -
PpaFLA5    : NGAFGGYLGGHS--------------------------------TYTPQMVSDVLKYHILLSYF-DTETIKTVST--MNDGVVTTLYQSTG-RANGM-DGFVNITVS-PT---------DGVVTI------------------------------------------- : 109
PpaFLA12   : ------------------------------------------------------------------------------------------------------------------------------------------------------------------------ :   -
PtFLA1     : DEAMA-KLTTDQ-----------------------L---------SEPGAPEQIIYYHVIPEYQ-TEESMYNAVRR-FGKISYDTLR---L--PHK-------VLAE--E------ADGSVKFGHTE---------NS--AYLFDPDIYT-DGRISVQG--------- : 350
PtFLA19    : DEAMA-KLTTDQ-----------------------L---------SEPGAPEQIIYYHVIPEYQ-TEESMYNAVRR-FGKISYDTLR---L--PHK-------VLAQ--E------ADGSVKFGHAE---------NS--AYLFDPDIYT-DGRISVQG--------- : 349
PtFLA6     : DEAMA-KLTTDQ-----------------------L---------SEPGAPEQIIYYHIIPEYQ-TEESMYNAVRR-FGKIGYDTLR---L--PHK-------VAAQ--E------ADGSVKFGSGD---------GS--AYLFDPDIYT-DGRISVQG--------- : 354
PtFLA8     : DEAMA-KLTTDQ-----------------------L---------SEPGAPEQIIYYHIIPEYQ-TEESMYNAVRR-FGKIGYDTLR---L--PHK-------VVAQ--E------ADGSVKFGSGD---------GS--AYLFDPDIYT-DGRISVQG--------- : 354
PtFLA5     : DDPFKAF--FPK--------------------FKNL----------TASGKVSFLEFFGVPIYQ-SLAMLKSN-----NGI-MNTLATDGE-K--K-----FDFTVQ-ND--------GEDVTLKT----RS----IT--A-KIVGTLID-EQPLAIYT--------- : 286
PtFLA22    : DDPFKAF--LPK--------------------FKNL----------TASGKESLLQFFGVPVYQ-SLAMLKSN-----NGI-MNTLATNGD-K--K-----FDFTVQ-ND--------GEDVTLKT----RG----TT--A-KIVGTLID-EQPLAIYS--------- : 286
PtFLA42    : DQAMDAYAK-------------------------------------NVSDYSSIFRKHVVPGL-FPRQDLEGFN----DGTSLPTFS---G--GFM-----INLTKS-----------GDVLVL-----N-------G--VPVIFPDMYQ-SDWLIIHG--------- : 252
PtFLA51    : ------------------------------------------------------------------------------------------------------------------------------------------------------------------------ :   -
PtFLA52    : ------------------------------------------------------------------------------------------------------------------------------------------------------------------------ :   -
PtFLA37    : DSLLFSVDLAS-----------------------------------TAPHYTKSLFLHVSPSR-LSTSDLRNLTAA-SGGTYIDSLV---P--NHR-----LLITNS-LAQL--NGTVDGSILV-----N-------R--VRVSVPDLFL-GSDIAVHG--------- : 100
PtFLA17    : DEAFKAA-GVPD--------------------LSKL----------TNAEIVSLLQYHATATYS-PFGSLKTS-----KDP-ISTLASNGA-G--K-----FDLTVT-SA--------GDSVTLHT----GI----GP--S-RVAETVLD-STPLVIFT--------- : 125
PtFLA53    : ------------------------------------------------------------------------------------------------------------------------------------------------------------------------ :   -
PtFLA41    : ------------------------------------------------------------------------------------------------------------------------------------------------------------------------ :   -
PtFLA38    : DSAFSDL-SATA----------------------------ISLQSLPADKKADVLKFHVLHSYY-PLGSLESI-----VNPVQPTLATEDM-GAGS-----FTLNIS-RV-------NGSV-AIDS----GI----VQ--A-SVTQTVFD-QNPVVIFG--------- : 300
PtFLA30    : DSAFSDL-SETD----------------------------VSLQSLPADKKADVLKFHVLHSYY-PLGSLESI-----VNPVQPTLATEDM-GAGS-----FTLNIS-RA-------NGSV-AIDS----GI----VL--A-SVTQTVFD-QNPVAIFG--------- : 300
PtFLA20    : DSAFSAL-KTGT--------------------LRTL----------TDQEKVELMQFHIVPMFI-SSSQFDTVSSP------LKTHA-GSG---AR-----FQLNVT-ASG------N--SLNIST----GL----TN--T-TISDTVYT-DTHLAIYQ--------- : 127
PtFLA23    : DNAFSSL-KSGT--------------------LNSL----------NDQEKAELVQFHIIPQYL-SSSQFQTVSNP------LTTQA-GSG---GR-----LELNVT-TTG------N--SVNITT----GL----TN--T-SVSGTIYT-DNQLAVYQ--------- : 127
PtFLA18    : DDAFKNL-KKAS--------------------LSNL----------TQDQLKQLILFHALPHYY-SLSDFKNLSQV------S-PVSTFAG--AGG-----YALNFT-DTS------G--TVHLDS----GW----SK--T-KVSSSVHS-TDPVAIYQ--------- : 128
PtFLA10    : DNAFSSL-KSGT--------------------LNSL----------SDQEKAELVQFHIIPQFL-SSSQFQTVSNP------LTTQA-GSG---GR-----LELNVT-TTG------N--SVNITT----GL----TN--T-SVSGTIYT-DNQLAVYQ--------- : 127
PtFLA16    : DPVINGF--MPK--------------------YKNL----------TAPQKVSLLLYHGIPIYQ-SLQMLKTS-----NGI-MNTLATNGA-N--K-----YDFTVQ-ND--------GEVVTLET----KV----TT--A-TITGTVKD-EEPLVVYK--------- : 284
PtFLA34    : DSAFSNL-KAGF--------------------LNAL----------SDEDKTELVKFHVLPALI-SSSQFQTVSNP------VRTQA-GTG---PR-----VTLNVT-TTG------N--FVNITT----GL----TN--T-SISGTVYT-DSQFAIYQ--------- : 127
PtFLA9     : DNSFANL-KAGT--------------------LNSL----------SDQQKVQLVQFHILPNFL-SMSNFQTVSNP------LRTQAGNSA--DGE-----FPLNVT-TSG------N--QVNITT----GV----NT--A-TVANTIYT-DGQLVVYQ--------- : 129
PtFLA12    : DDAFKNL-KKPS--------------------LSNL----------TQDQVKQLILFHALPHYY-ALADFKNLSQV------S-PVSTFAG--AGG-----YALNFT-DVS------G--TVHLDS----GW----SK--T-KVSSSVHS-TDPVAVYQ--------- : 128
PtFLA7     : DNAFTNL-KAGT--------------------LNSL----------SDQQKVQLVQFHIIPNFF-SMSSFQTVSNP------LRTQAGNSA--DGE-----FPLNVT-TSG------N--QVNITT----GV----NT--A-TVANTIFT-DGQLVVYQ--------- : 129
PtFLA2     : DNAFSSL-KAGF--------------------LNSL----------SDEDKTELVKFHVLPAFI-STSQFQTVSNP------VRTQA-GTG---PR-----VTLNVT-TTG------N--FVNISS----GL----TN--T-SISGTVYT-DSQLAIYQ--------- : 127
PtFLA13    : DSAFSEL-KSGT--------------------LNTL----------SDGDKSELVKFHVVPTFL-STSQFQTVSNP------LGTWA-GTG---SR-----LPLNVT-SYP------N--SVNITT----GL----TN--T-SLSGTVYT-DNQLAIYK--------- : 127
PtFLA47    : DSAFSEL-KSGT--------------------LNTL----------SDGDKSELVKFHVVPTFL-STSQFQTVSNP------LGTWA-GTG---SR-----LPLNVT-SYP------N--SVNITT----GL----TN--T-SLSGTVYT-DNQLAIYK--------- : 127
PtFLA40    : DSAFSEL-KSGT--------------------LNTL----------SDGDKSELVKFHVVPTFL-STSQFQTVSNP------LGTWA-GTG---SR-----LPLNVT-SYP------N--SVNITT----GL----TN--T-SLSGTVYT-DNQLAIYK--------- : 127
PtFLA28    : DSAFSEL-KSGT--------------------LNTL----------SDGDKSELVKFHVVPTFL-STSQFQTVSNP------LGTWA-GTG---NR-----LPLNVT-SYP------N--SVNITT----GL----TN--T-SLSGTVYT-DNQLAIYK--------- : 127
PtFLA39    : DSAFSEL-KSGT--------------------LNTL----------SDGDKSELVKFHVVPTFL-STSQFQTVSNP------LGTWA-GTG---SR-----LPLNVT-SYP------N--SVNITT----GL----TN--T-SLSGTVYT-DNQLAIYK--------- : 127
PtFLA50    : DSAFSEL-KSGT--------------------LNTL----------SDGDKSELVKFHVVPTFL-STSQFQTVSNP------LGTWA-GTG---SR-----LPLNVT-SYP------N--SVNITT----GL----TN--T-SLSGTVYT-DNQLAIYK--------- : 127
PtFLA29    : DSAFSEL-KSGT--------------------LNTL----------SDGDKSELVKFHVVPTFL-STSQFQTVSNP------LGTWA-GTG---SR-----LPLNVT-SYP------N--SVNITT----GL----TN--T-SLSGTVYT-DNQLAIYK--------- : 127
PtFLA32/49 : DSAFSEL-KSGT--------------------LNTL----------RDGDKSELVKFHVVPTFL-STSQFQTVSNP------LGTWA-GTG---SR-----LPLNVT-SYP------N--SVNITT----GL----TN--T-SLSGTVYT-DNQLAIYK--------- : 127
PtFLA45    : DSAFSEL-KSGT--------------------LNTL----------SDGDKSELVKFHVIPTFL-STSQFQTVSNP------LGTWA-GTG---SR-----LPLNVT-SYP------N--SVNITT----GL----TN--T-SLSGTVYT-DNQLAIYK--------- : 127
PtFLA35    : DSAFSEL-KSGT--------------------LNTL----------SDGDKSELVKFHVIPTFL-STSQFQTVSNP------LGTWA-GTG---SR-----LPLNVT-SYP------N--SVNITT----GL----TN--T-SLSGTVYT-DNQLAIYK--------- : 127
PtFLA46    : DNAFSSL-VAGA--------------------VGSL----------NDREKLEFVQFHILPRFL-SISDFQTLSNP------VKTLA-GSD---RK-----FPLTIT-TSD------N--SVTVSS----GL----TK--T-SISNTIYT-DKQVAIYE--------- : 127
PtFLA3     : DNAFNNL-KAGA--------------------LNGL----------NQQEQVQLLQYHTLPKFY-TMSNLLLVSNP------VPTQASGQD---GV-----WGLNFT-GQ--------SNQVNVST----GL----VE--V-QINNALRQ-DSPLAVYP--------- : 128
PtFLA15    : DNAFSNL-KSGA--------------------LNGL----------SQQQQVQLLQYHMLPKFY-SLSNLLLVSNP------VPTQASGQE---GV-----WGLNFT-GQ--------SNQVNVST----GL----VE--V-QVNNALRQ-DFPLAVYP--------- : 128
```

Type 2

```
PtFLA14     : DSAFSEL-KSGT--------------------LNTL----------SDGDKSELVKFHVIPTFL-STSQLQTVSNP------LGTWA-RTG---SR-----LPLNVT-SYP------N--SVNITT----GL----TN--T-SLSGTVYT-DNQLAIYK--------- : 111
PtFLA48     : DGAFSAIIKSGV--------------------LNSL----------TDHQKIELVQFHIIPRIL-TTANFQTVSNP------ITTLA-GSG---NR-----FALNVI-TTE------N--MVNVTT----GL----TN--T-SVSAIVYT-DSQLAIYQ--------- : 128
PtFLA27     : DGAFSAIIKSGV--------------------LNSL----------SDHQKIELVQFHIIPRIL-TTANFQTVSNP------ITTLA-GSG---SR-----FALNVI-TTE------N--MVNVTS----GL----TN--T-SVSAIVYT-DSQLAIYQ--------- : 128
PtFLA43     : DSAFSEL-KSGT--------------------LNTL----------SDGDKSQLVKFHVVPTFL-STSQFQTVVG--------------------------YHLTSQ-SYT------N--SVNITT----GL----TN--T-SLSGTVYT-DNQLAIYK--------- : 116
PtFLA33     : DSAFSEL-KSGT--------------------LNTL----------SDGDKSELVKFHVVPNSY-LLPSSR-----------------------------------------------------------------------PLSGTVYT-DNQLAIYK--------- :  91
PtFLA21     : NGNMSPLS------------------------------------GKPNGEIKNVLSGHVILDYY-DVAKLQKLQ---NKTAMLTTL---------------------------------------------------------------------------------- :  77
PtFLA26     : NGSIDGLS------------------------------------GRPLDIAKRILSAHVILDYY-DQIKLSKLQ---KASTI-------------------------------------------------------------------------------------- :  73
PtFLA4      : ELEFVASS---------------------------------------SPMLEKIVRLHILPQR-ATYIELAALP----DKQRLRTLL---P--DED-----LKITKG--------VGVTQGLAI-----N-------G--VEIAAPEIFS-SKEFIVHG--------- : 258
PtFLA44     : DVSIF-SG---------------------------------------SHGYISSVRFHIVPNHYLSTADLERLP----VGATLPTLE---R--GQA-----LVVTSA-GGLT--GFNTAVPMRI-----N-------Y--VRVKVPDVMR-NLKIVVHA--------- : 275
PtFLA24     : ------------------------------------------------------------------------------------------------------------------------------------------------------------------------ :   -
SmoFLA1     : ------------------------------------------------------------------------------------------------------------------------------------------------------------------------ :   -
SmoFLA2     : ------------------------------------------------------------------------------------------------------------------------------------------------------------------------ :   -
SmoFLA3     : DHAMD-HLTTEQ-----------------------L---------NAPGGLEAILMYHVLTEYQ-TEESLYNAVRR-FEKVKFLTLW---Q--PHT-------IHAK--E------TDGTVQFGEGE---------SG--ALLYDHDIFT-DGHISIQGISKVL---- : 133
SmoFLA6     : DDAFKAL-PPNA--------------------LAEL----------SQAQKIALLQFHAIATYF-TMGSLRSV-----A-TPLPTVASSRI--GG------FELNVS-TAG-------GKGVSFVT----GL----NR--A-DVTDTELD-TPPVAVYA--------- : 288
SmoFLA7     : DEAFQGL-PNGT--------------------MAAL----------SPSQASSLMAYHTLPAYT-SSGSLQRQ-----N-SLVQTVASNGD--NQK-----FLIQVA-PSG-----GNSGGVSLST----GV----DT--A-DVVSTIYD-QPPTAAYS--------- : 296
SmoFLA8     : DSAFQNL-PSGS--------------------LAAL----------TQSQRQLLVRYHLLPSFF-TFGSLRTL-----K-APLTTLATSN----RN-----FEVNAS-GE------GPSGGLAIAT----GV----ST--A-NVIATLLE-DDPVGVYA--------- : 294
SmoFLA9     : DRAVA-RLAAEH-----------------------L------------GAIESILAYHIVAEYQ-TEESLYTL----------------------------------------------------------------------------------------------- : 281
SmoFLA4     : NNTLLNDFIYDM-----------------------------GK-HPSEEKLADLVRYHISDFYI-ESEFLAVRR---NFSSPIKTLFRERP-YEETLHFRWLQLNVD-NH---------GVATLSRPPHQSP----PL--ATILRNVVQE-PFSITVYA--------- : 138
SmoFLA5     : DSVLDPILDQLA------------------------------Q-SVTGQQIADILRYHVLLEYE-GINDLRSLP---NKSKLFTTLFQTTG-RASNN-AGFVNITDD-PN---------GGVSVGWPSSSTV----FS--SEILGTVKEL-PFNVSVVD--------- : 136
BdiFLA23    : DEAMA-RLTTDQ-----------------------L---------SEPGSPENILYYHMIPEYQ-TEESMYNAVRR-FGKVRYDTLR---L--PHK-------VVAR--E------ADGSVKFGQGE---------GS--AYLFDPDIYT-DGRISVQG--------- : 350
BdiFLA22    : DEAMA-RLTTDQ-----------------------L---------SEPGSPENILYYHMVPEYQ-TEESMYNAVRR-FGTVRYDTLR---L--PQK-------VTAR--E------ADGSVKFGHGE---------GS--AYLFDPDIYT-DGRISVQG--------- : 351
BdiFLA9     : DAAVASF--MPK--------------------YKNL----------TAKAKTAILLYHAVPDYY-SLQLLTSN-----SGK-VSTLATSSV-AKKD-----YSFDVS-KD--------GETAALDT----KV----IT--A-SVTATVKD-DDPLAVYA--------- :  99
BdiFLA1     : DAAFEDL-PSGT--------------------MNGL----------SSQDQIQMMLYCVLPRFY-SLSMLGTLNGK------VSTQASGSD---GP-----YEYKIK-PSG--------SNVNVST----GV----KGNNM-LLSTIVSK-EFPLAVYS--------- : 100
BdiFLA2     : ------------------------------------------------------------------------------------------------------------------------------------------------------------------------ :   -
BdiFLA20    : DDAFAAG-GLPD--------------------A------AGRLQSLPADRKAVVLRFHVLHSYY-PLGSLESI-----VNPVQPTLATECT-EAGR-----FTLNIT-RS-------NGSV-AIDT----GV----VQ--A-SITRTVFD-QNPVAVFA--------- : 305
BdiFLA10    : DDAFASL-PAG---------------------P------GDRLQSLPAERKAVVLRYHVLHSYY-PLGSLESI-----VNPLQPTLATEFSSQAGR-----FTLNIT-RA-------NGSV-AIDT----GV----VQ--A-TITRTVFD-QNPVAVFA--------- : 132
BdiFLA11    : DSAFAALKKSTF---------------------SNL----------TSDQLKMLLMYHALPEFY-SLAQFRNL-----S-V-LNPVN-TFA--GAP-----YTLNLT-DD--------MGTISVKS----MW----SK--P-TISSSVYA-TDPVAIYS--------- : 127
BdiFLA3     : DDAFQAK-GLPD--------------------LSKL----------TSANLVTLLEYHALPQYA-PKASLKTM-----KGG-IPTLASTGS-G--K-----YDLSVV-TK--------GDDVSMAT----GM----DK--S-RVASTVLD-DTPVAVHT--------- : 125
BdiFLA12    : DSAFSSLRATTF---------------------ANL----------TGEELKSLVLYHALPKYY-SLAEFNKL-----S-S-LNPVP-TFA--GSQ-----YTLNLT-DN--------MGSIRVKS----MW----SN--P-KISSSVYS-TRPVAVYE--------- : 130
BdiFLA8     : DNAFNNL-KPGT--------------------LNSL----------TQQQQVALVQGHVLPQFY-SMESFQTASNP------VRTQASGQD---GP-----FTLNIT-ATA------N-NQVNVSS----GV----SE--V-TVNNALSD-KKPLAVYS--------- : 131
BdiFLA13    : DNAFDSL-KAGT--------------------LNSL----------SQQEQVSLVQAHIVPAFF-SMESFETASNP------VRTQASGAD---GP-----YTVNVT-ATS------N-GQVNVST----GL----VS--T-MVGTALRK-EKPLAVYS--------- : 129
BdiFLA4     : DRAFAAIEPSVL---------------------SGL----------KKHQVKSLMMYHALARHY-ALKEFDAL-----S-R-VSPVT-TFA--GGL-----YTVNVT-YD--------AGAIRVVS----SW----AD--A-KVVRPVYE-MPPMAVYE--------- : 127
BdiFLA14    : DNAFSAL-KSGT--------------------LNAL----------SDAQKTSLVQFHVVSQLI-PMAQFDTASNP------LRTQAGETR--PGK-----YPLNVT-ADG------Q--QVNIST----GV----VN--A-SVSGTVYT-GDRLVVYQ--------- : 131
BdiFLA15    : DNAFTSL-ASGT--------------------LNSL----------SDSQKNSLVQFHVLSTAV-PMSQFDTVSNP------LRTQAGSSS--PGE-----YPLNVT-ATG------Q--QVNIST----GV----VN--A-TVDNTLFT-GDQLVVYQ--------- : 132
BdiFLA16    : DSAFAAVDSAAL---------------------SNL----------TADRLRSLMLHHAAPKYY-PLSVFSAL-----A----ASST-PVS--MFA-----YSVNVT-DK--------AGKTGVVS----GW----AA--A-KLVSSVYS-TRPVAVYA--------- : 129
BdiFLA17    : DGAVKSF--LPR--------------------YKNL----------TAAGKAALLLSHAVPVYY-TRRALKSN-----NGV-MNTLATDGG-A-GN-----FNLTVQ-NV--------GDQVSVKT----AG-KGKGA--A-RVESTVYD-KEPVAVYG--------- : 129
BdiFLA21.1  : NTDIPKVIH-----------------------------------TLPRPLLADLLALHVIPDYL-DPEKLDALRR--GRTG-----------DGS------------------------------------------------------------------------- :  77
BdiFLA21.2  : NTALADSLRGLR--------------------------------HLPEPALVDLLALHVVLDYL-DPEKLDALRR--GRTG-----------GGSIV-T--------------------------------------------------------------------- :  83
BdiFLA5     : DDAFVAKAGTPD--------------------VGKM----------EKDDLVRLLMYHAVPAYE-PKPSLKLVKA--GARP-LRTLASTAA-G--E-----YNVTVV-AR--------GDDVSLDT----GV----RK--S-RVAATVLD-EVPLCVLT--------- : 130
BdiFLA18    : DAAVRAF--MPR--------------------YKNL----------TADGKASLLLFHAVPVYY-SPGSLKSN-----NGV-MNTLATDGA-S-KN-----FNFTLQ-NE--------GEAVAIKT----GASPGGGV--A-RVEATVMD-RDPVAVYR--------- : 130
BdiFLA6     : NAAVAQLKA--R--------------------------------RLAPESLAHVLSLHVLLDYF-DDARIRHL----------------------------------------------------------------------------------------------- :  69
BdiFLA19    : NADVDWLLRRSS--------------------------------RLPRAALVELLAVHVILDYI-DAAKL-------------------------------------------------------------------------------------------------- :  68
BdiFLA7     : DKAAGAIT------------------------------------ALPADTQKKVLAVHVILDYY-------------------------------------------------------------------------------------------------------- :  60
MpoFLA6     : DEAFNKL-PGQW--------------------FEAF----------ELKEQKLLLEYHALTRYN-SLDALWRY-----RDKQVPTVSSTVQ--EGP-----DAFNLL-VTA-----NKGLV-TIHSS-TPDK----PV--A-TLQSTLFD-ASPLVLYS--------- : 294
MpoFLA1     : NAAIQGLV---NVLN---------------ASGLTLA----NVTGN-NNKAASIVLYHVATLVA-TSTQLVNDQS-------LPTLY---G-GY-N-----LTVDRN---A-----T--NVEIDGGA-DT-------Q--A-TVVTPDVR-VCGSVVHI--------- : 128
MpoFLA14    : DKAWSGALTEEH-----------------------L---------SSQTALEDILHYHIITEYQ-TEESLYSTLRR-MGKTHFSTLR---V--PHK-------LAAH--E------VDGQVVFGEGD---------DA--AGVFDHDIFA-DGRLSVQGIDRVMIP-- : 358
MpoFLA4     : ------------------------------------------------------------------------------------------------------------------------------------------------------------------------ :   -
MpoFLA7     : DSAFNAL-KSGS--------------------IQTL----------SPGKQKLLMQYHALTQYY-TMTNFLSM-----VDAPVQTVSSTVG--GGY-----L-MNIT-SK-------ASTV-TIKT----GV----GN--A-TVGEVLYN-ANPVTMYA--------- : 286
MpoFLA2     : NAAFEELL---QVLN---------------SSGLTLD----DVTAPESNKAASILLYHVVPVAA-LSTQLSDMQV-------LPTLL-----GK-N-----LTVSLM---G-----G--MVNIIA-Q-ES-------N--A-TVVTADVT-VCGSVVHI--------- : 127
MpoFLA3     : NAAFEELL---QVLN---------------SSGLTLD----DVTAPESNKAASILLYHVVPVAA-LSTQLSDMQV-------LPTLL-----GK-N-----LTVSLM---G-----G--MVNIIA-Q-ES-------N--A-TVVTADVT-VCGSVVHI--------- : 127
MpoFLA5     : ------------------------------------------------------------------------------------------------------------------------------------------------------------------------ :   -
MpoFLA8     : DSAFNSL-ATGS--------------------IQTL----------SLSEQKLLLEYHALDGYE-SLDSLETM-----LNKPTKTLASADA--AGY-----I-LNVS-ATP-----GQSTV-RLRT----GV----SV--A-TVLEIIYD-ANPVTMYA--------- : 287
MpoFLA9/10  : DSAFNGL-TSAS--------------------LQAL----------SVTDLKLLMQYHALTSYQ-PLDILQNM-----VDSPVSTIASTIA--SGY-----L-LNVS-SA-------VMTV-TLHT----GV----ND--A-KIIETLYD-ARPVTMFG--------- : 286
MpoFLA11    : DSAFNSL-ASGS--------------------IQGL----------SVSDLKLLMQYHALTSYQ-PLSVLQNM-----VNSPVSTIASTIA--SGY-----L-LNVS-SA-------AKTV-TLHT----GV----SV--A-TIQETLYD-ATPTTMFG--------- : 286
MpoFLA12    : DSAFNSL-ASGS--------------------IQGL----------SVSDLKLLMQYHALTSYQ-PLSVLQNM-----VNSPVSTIASTIA--SGY-----L-LNVS-SA-------AKTV-TLHT----GV----SV--A-TIQETLYD-ATPTTMFG--------- : 286
MpoFLA13    : DAAFAALPAGAL------------------------------A-ALTPDQAKQVLRAHAIVTYY-PLGTLSTM----------------------------------------------------------------------------------------------- : 237
CcrFLA1.1   : NAAFAALTNKLSPGAGVDAADPEGILFALMGALPGP-----LPGRSPGEVLTTILSYHLLAFAA-PFQELENDGT-------AETVQ---G--T-L-----LRFRDG------------RVVDRDPS-RR-------N--P-RPDPRNVF-TQNGWVHV--------- : 144
CcrFLA1.2   : DEAFTALANQLVPGADLNPADKDAVVEALVSAIAPLA----DVEAAANSTIESILGYHALPFAA-PFQELEDMSS-------AETVQ---G--D-A-----LRFADG------------LVIDADES-RE-------N--P-VADPRNFF-PQNGWVHV--------- : 145
CcrFLA1.3   : DEAFTNLANTLVPDANLEASDKDAVVEALVTAIAPLA----DVEAAGGATIESILLYHALGMAA-PLGKLEEMKT-------AKTLQ---G--G-V-----LSFNSG------------SVTDDDPS-RE-------D--A-VVDPRNIF-TQNGWVHV--------- : 130
CcrFLA1.4   : DEAFTNLANTLAPDANLEASDKDAVVEALVTAIAPLA----DVEAAGGATIESILLYHTLGEAS-PIQELEKSDT-------LTTLQ---G--D-S-----ISVADG------------KVTDGDDS-RD-------D--A-AISTPNIF-LQNGWVHV--------- : 129
CbrFLA1     : NAAFARLGPGQL-----------------------------DTIKADEALLYEFLGYHFADGFY-PKADLLAAI---AKSTSGTVSVPTQG-------GSYVSFSSV-GD---------V-IKVDGS--TSV----TE--A---DFLVDS-PNKAVAHV--------- : 125
CbrFLA2     : NAAFSTALSE--E------------------VITCL-----QKEPGQA-TLASFLKY--------------------------------------------------------------------------------------------------------------- :  66
CbrFLA3     : NGAFDALGAV---------------------YTECM-----LKRPGMDDLLPLIVRFHVAT-GNFVNAQLMTMSS-------VQSFL---G-LP-------IPLTH-AAAG--------GLVLD-----G-------Q--ATVTGPDVKI-AVNATVHT--------- : 123
CbrFLA4     : NSAFDKLGSSL---------------------VDCLLSDS------SSGLLDIILLNHIFEGGH-PSKSLTD-GQK------LLTVA---N-TT-------AEIAV---N----E----TGAF-----IE-------G--AKIVEPDLFL-TGESAVHG--------- : 123
CbrFLA7     : NGAFAMLSPE---------------------VKQCL-----DTQSGKIDVLTQIMLFHLAVGGNYTAAELKTISR-------LTAAS---G-MP-------IDLMT-LGDG--------TIMVE-----G-------T--ARVVVPDAIT-GVNATVHI--------- : 125
CbrFLA9     : NGAFAMLSPE---------------------LKQCL-----DTQSGKIDVLTQIMLFHLAVGGNYTVAELKTISR-------LTAAS---G-MP-------IDLMT-LGDG--------TIMVE-----G-------T--ARVVVPDAIT-GVNATVHI--------- : 125
CbrFLA11    : EAAFAVLSSAYPA------------------QLACV-----TTGQGIDNYLSQILKYHILPNGNFTASKLTKLTQ-------VSTLI---G-LP-------IQLEYTLPSG--------PLKLD-----M-------T--AEVVQADAIF-AGNGTVHV--------- : 128
CbrFLA15    : DAAVSDQIQRLG---------------------GCLA----AIPDVADLVMTSSLLYSAVPGHL-PMAEIRSRLNQSNGSLRLPTLY---P-PN-E-----VAVSSA-TSR---YPSTKMGVFVDGA-QI-------F--P-SGGFDVVT-APDVVVQM--------- : 143
CbrFLA16.1  : NDVIEALNVGD---------------------TKFLTDPK------NVELLKRLVFYHFVNSKI-LAVNLTA-GRS------LRSLE---G-EN-------LIVEV---E----D----GTTF-----VQ-------G--AGITAADDII-REDGVVHG--------- : 117
CbrFLA16.2  : NQGMSSSVE-M----------------------AQLLTNA--------TARNDILLYHLLAGRY-TFSWLKEHPGV------YDNFT---G-FE-------I--VV--SGG---G----TEVFVGNA-AS-------L--GQIVTPDLYA-NPKIVVHG--------- :  94
CbrFLA16.3  : NEAITNFMLAY----------------------AALLSDN--------AKLDEVLKFHVLNGSY-SALFLRTNPGD------YGG--------N-------F--SVV-VQ----G----DSLRVGYS-LA-------L--GSVVAPDLYA-TSSITIHG--------- :  91
```

```
                                                                                                     Type 2
CbrFLA16.4 : NEAITNFMVAN----------------------AALLGDR--------AKLDEVLKFHVLNGSY-TASFLRKNPGD------YGG--------N-------F--SVD-VQW---D----NSLMVGYS-SA-------R--GLVVAPDLYA-TSKITIHG--------- : 116
CbrFLA19   : NTAFSVFDPA---------------------VLDCL-----YSKPASLNVLSQITKYHILV-GNHNATELIAMQT-------VHADS---G-LP-------LHFNL-TSRG--------RVRISDDD-GG-------E--AFMTRPDFRL-LINSTIHV--------- : 128
CbrFLA20.1 : NSAWDFLDPAM---------------------KDCLMAGD--------KLVDVVVKSHTLIGKL-TAEMISNVPR-------VENLY---R-FW-------L--NVT-TDD---I----TNLT---S-VN-------N--VEIIRPDVYM-FPTKVVHG--------- : 102
CbrFLA22   : DDSITKLDPE---------------------LWTCV-----TTGQGPLDILSQITLYHFVTDGNFTAAEVATKRH-------LTSAS---G-MP-------LGVKV-V-NG--------NVMLE-----D-------Y--APITEPNALR-TSNATVHL--------- : 122
CbrFLA23   : DDSFMKLDPE---------------------LWRCA-----TTGQGPLDVLSQIMLYHFVTSGNFTAAEVATKTQ-------LTSAS---G-MP-------IDVKD-V-NG--------NVVLE-----G-------Y--ASITGPDALK-SPNATVHL--------- : 121
CbrFLA24   : DDSFMKLDPE---------------------LWRCA-----TTGQGPLDVLSQIMLYHFVTSGNFTAAEVATKTQ-------LTSAS---G-MP-------IDVKD-V-NG--------NVVLE-----G-------Y--AAITGPDALT-SPNATVHL--------- : 121
              [a]Type 2                                                 h
                  *
AtrFLA10   : -----IDKVIFPP : 344
AtrFLA11   : -----IDKVIQPK : 293
AtrFLA5    : -----IF------ :  90
AtrFLA1    : ------------- :   -
AtrFLA2    : -----VPKVILP- : 135
AtrFLA7    : -----VDSVILP- : 134
AtrFLA3    : -----VDKVILP- : 138
AtrFLA8    : -----VDKVILP- : 135
AtrFLA9    : -----VDNVILP- : 131
AtrFLA4    : -----ISHVILP- : 128
AtrFLA6    : ------------- :   -
AtrFLA12   : -----I------- :  89
FLA16      : -----IDGVIFPK : 358
FLA17      : -----IDGVIFP- : 360
FLA15      : -----IDGVIFPE : 354
FLA18      : -----IDGVIFP- : 366
FLA1       : -----TDKVILPK : 292
FLA22      : ------------- :   -
FLA20      : -----FNQMI--- : 281
FLA21      : -----ISHTLEIP : 269
FLA19      : -----IADLIPL- :  99
FLA4       : -----VSKVILP- : 315
FLA10      : -----VDNVILP- : 132
FLA8       : -----VDNVILP- : 132
FLA11      : -----VDQVILP- : 137
FLA12      : -----VDKVILP- : 136
FLA7       : -----VNRVILP- : 134
FLA9       : -----VDMVILP- : 136
FLA6       : -----VDSVILP- : 138
FLA13      : -----VDMVILP- : 137
FLA14      : ------------- :   -
FLA3       : ------------- :   -
FLA5       : ------------- :   -
FLA2       : -----IDKVILPR : 293
CreFLA11.1 : -----VDTVIVP- : 107
CreFLA11.4 : -----IDGVLV-- : 129
CreFLA1    : -----INKVILP- : 131
CreFLA10.2 : -----VDRVI--- : 145
CreFLA9    : ------------- :   -
CreFLA8.1  : -----VDKVILP- : 131
EgrFLA9    : -----IDGVIFPP : 365
EgrFLA5    : -----IDGVIFP- : 362
EgrFLA10   : -----IDKVILP- : 294
EgrFLA11   : -----IDKVIMPK : 294
EgrFLA16   : -----VLDVI--- : 252
EgrFLA14   : -----ILDVI--- : 241
EgrFLA15   : ------------- :   -
EgrFLA19   : ------------- :   -
EgrFLA4    : -----VSRVILP- : 137
EgrFLA12   : -----IDKVIQP- : 292
EgrFLA8    : -----VDHVILP- : 134
EgrFLA7    : -----IDKVILP- : 134
EgrFLA13   : ------------- :   -
EgrFLA6    : -----VDKVILP- : 131
EgrFLA1b   : -----VDKVILP- : 136
EgrFLA3b   : -----VDQVILP- : 122
EgrFLA2b   : ------------- :   -
EgrFLA17   : ------------- :   -
EgrFLA20   : ------------- :   -
OsaFLA17   : -----IDAVIFPP : 351
OsaFLA24   : -----IDAVIFPP : 358
OsaFLA16   : -----IDAVIFPP : 358
```

Type 2

```
OsaFLA4     : -----VDTVLEP- : 107
OsaFLA23    : -----IDAVLMPL :  94
OsaFLA12    : -----VSKVLLP- : 304
OsaFLA1     : -----VDSVLLP- : 132
OsaFLA5     : -----VDRVLLP- : 135
OsaFLA8     : -----VDKVLLP- : 136
OsaFLA27    : -----LNKVLLP- : 134
OsaFLA26    : -----VSKVLLP- : 135
OsaFLA7     : -----VDKVLLP- : 138
OsaFLA14    : -----LDTVLLP- : 134
OsaFLA2     : -----VDNLLMP- : 136
OsaFLA11    : -----VTKFLKP- : 294
OsaFLA9     : -----VDKVLLP- : 139
OsaFLA3     : -----ISKFLQP- : 132
OsaFLA18    : -----VDKVLLP- : 136
OsaFLA13    : -----LNRVLLP- : 139
OsaFLA15    : -----LDAVLLP- : 138
OsaFLA19    : ------------- :   -
OsaFLA10    : -----VDAVIEP- : 135
OsaFLA6     : -----VDKVPLP- : 135
OsaFLA25    : ------------- :   -
OsaFLA20    : ------------- :   -
OsaFLA28    : ------------- :   -
PabFLA22    : -----IDKVLFP- : 354
PabFLA9     : ISVQGIDKVLFPP : 120
PabFLA1     : ------------- :   -
PabFLA2     : ------------- :   -
PabFLA3     : ------------- :   -
PabFLA4     : ------------- :   -
PabFLA17    : ------------- :   -
PabFLA15    : -----VPRVLLP- : 305
PabFLA5     : -----VPKVLLP- : 146
PabFLA20    : ------------- :   -
PabFLA21    : -----VDKLLKP- : 291
PabFLA16    : -----ISGVLLP- : 292
PabFLA10    : -----VGKVLLP- : 136
PabFLA6     : -----VDKVLEP- : 135
PabFLA11    : -----IDKVLKP- : 124
PabFLA18    : -----IVRPFL-- : 313
PabFLA7     : ------------- :   -
PabFLA19    : ------------- :   -
PabFLA12    : -----VNKVLLP- : 114
PabFLA13    : -----VDKVLLP- :  84
PabFLA8     : -----VDKVLKP- :  92
PabFLA14    : -----MGKVLLP- : 125
PabFLA23    : -----IDRVL--- :  99
PabFLA24    : ------------- :   -
PpaFLA10    : --------VLTPP : 374
PpaFLA11    : --------VLSPP : 361
PpaFLA6     : ------------- :   -
PpaFLA7     : ------------- :   -
PpaFLA8     : ------------- :   -
PpaFLA9     : ------------- :   -
PpaFLA1     : -----VNTVLLP- : 141
PpaFLA2     : -----VNNVLLP- : 141
PpaFLA3     : ------------- :   -
PpaFLA4     : ------------- :   -
PpaFLA5     : ------------- :   -
PpaFLA12    : ------------- :   -
PtFLA1      : -----IDGVLFPL : 358
PtFLA19     : -----IDGVLFPL : 357
PtFLA6      : -----IDGVLFPE : 362
PtFLA8      : -----IDGVLFPE : 362
PtFLA5      : -----IDKVLLP- : 293
PtFLA22     : -----IDKVLLP- : 293
PtFLA42     : -----LNQLLTPP : 260
PtFLA51     : ------------- :   -
PtFLA52     : ------------- :   -
PtFLA37     : -----LDGIL--- : 105
PtFLA17     : -----VDNVLLP- : 132
PtFLA53     : ------------- :   -
```

```
                   Type 2
PtFLA41     : ------------- :   -
PtFLA38     : -----VSKVLLP- : 307
PtFLA30     : -----VSKVLLP- : 307
PtFLA20     : -----VDKVLLP- : 134
PtFLA23     : -----VDKVLLP- : 134
PtFLA18     : -----VDKVLLP- : 135
PtFLA10     : -----VDKVLLP- : 134
PtFLA16     : -----INKVLLP- : 291
PtFLA34     : -----IDKVLFP- : 134
PtFLA9      : -----VDQVLLP- : 136
PtFLA12     : -----VDKVLLP- : 135
PtFLA7      : -----VDQVLLP- : 136
PtFLA2      : -----LDKVLFP- : 134
PtFLA13     : -----IEKVLLP- : 134
PtFLA47     : -----IEKVLLP- : 134
PtFLA40     : -----IEKVLLP- : 134
PtFLA28     : -----IEKVLLP- : 134
PtFLA39     : -----IEKVLLP- : 134
PtFLA50     : -----IEKVLLP- : 134
PtFLA29     : -----IEKVLLP- : 134
PtFLA32/49  : -----IEKVLLP- : 134
PtFLA45     : -----IEKVLLP- : 134
PtFLA35     : -----IEKVLLP- : 134
PtFLA46     : -----VDKVLVP- : 134
PtFLA3      : -----VDKVLLP- : 135
PtFLA15     : -----VDKVLLP- : 135
PtFLA14     : -----IEKVLLP- : 118
PtFLA48     : -----VDKVLLP- : 135
PtFLA27     : -----VDKVLLP- : 135
PtFLA43     : -----IEKVLLP- : 123
PtFLA33     : -----IEKVLLP- :  98
PtFLA21     : ------------- :   -
PtFLA26     : ------------- :   -
PtFLA4      : -----I------- : 259
PtFLA44     : -----V------- : 276
PtFLA24     : ------------- :   -
SmoFLA1     : ------------- :   -
SmoFLA2     : ------------- :   -
SmoFLA3     : ------------- :   -
SmoFLA6     : -----VNRVLLP- : 295
SmoFLA7     : -----VNRVLLP- : 303
SmoFLA8     : -----LDAVLLP- : 301
SmoFLA9     : ------------- :   -
SmoFLA4     : -----IDRVLEP- : 145
SmoFLA5     : -----VSRVLVP- : 143
BdiFLA23    : -----IDAVLLPE : 358
BdiFLA22    : -----IDAVLFPP : 359
BdiFLA9     : -----VSKFLQPK : 107
BdiFLA1     : -----VDKVPLP- : 107
BdiFLA2     : ------------- :   -
BdiFLA20    : -----VSKVLLP- : 312
BdiFLA10    : -----VSKVLLP- : 139
BdiFLA11    : -----LNKVLLP- : 134
BdiFLA3     : -----VDSVLLP- : 132
BdiFLA12    : -----VDKVLLP- : 137
BdiFLA8     : -----VDKVLLP- : 138
BdiFLA13    : -----VDKVLLP- : 136
BdiFLA4     : -----IDRVLLP- : 134
BdiFLA14    : -----VDKVLLP- : 138
BdiFLA15    : -----VNQVLLP- : 139
BdiFLA16    : -----LDRVLLP- : 136
BdiFLA17    : -----VDAVLEP- : 136
BdiFLA21.1  : ------------- :   -
BdiFLA21.2  : ------------- :   -
BdiFLA5     : -----VDSLLMP- : 137
BdiFLA18    : -----LDAVVEP- : 137
BdiFLA6     : ------------- :   -
BdiFLA19    : ------------- :   -
BdiFLA7     : ------------- :   -
MpoFLA6     : -----IDEVLLP- : 301
MpoFLA1     : -----VNAVLLP- : 135
```

Type 2

```
MpoFLA14    : ------------- :   -
MpoFLA4     : ------------- :   -
MpoFLA7     : -----IDKVLLP- : 293
MpoFLA2     : -----INHVLIP- : 134
MpoFLA3     : -----INHVLIP- : 134
MpoFLA5     : ------------- :   -
MpoFLA8     : -----IDEVLLP- : 294
MpoFLA9/10  : -----IDAVLLP- : 293
MpoFLA11    : -----IDQVLLP- : 293
MpoFLA12    : -----IDQVLLP- : 293
MpoFLA13    : ------------- :   -
CcrFLA1.1   : -----IDTVLLP- : 151
CcrFLA1.2   : -----IDLVLLP- : 152
CcrFLA1.3   : -----IDSVLLP- : 137
CcrFLA1.4   : -----VDSVLLP- : 136
CbrFLA1     : -----IASVLL-- : 131
CbrFLA2     : ------------- :   -
CbrFLA3     : -----IDTVMVP- : 130
CbrFLA4     : -----IDQIIM-- : 129
CbrFLA7     : -----INDIIFP- : 132
CbrFLA9     : -----INDIIFP- : 132
CbrFLA11    : -----IDSVLIP- : 135
CbrFLA15    : -----VDTLLIP- : 150
CbrFLA16.1  : -----IDSILFP- : 124
CbrFLA16.2  : -----INKVLLPP : 102
CbrFLA16.3  : -----VDTVLLP- :  98
CbrFLA16.4  : -----VDRVLLP- : 123
CbrFLA19    : -----IDTVMIP- : 135
CbrFLA20.1  : -----FSRIMLP- : 109
CbrFLA22    : -----IGELLVP- : 129
CbrFLA23    : -----IGELLVPE : 129
CbrFLA24    : -----IGELLVP- : 128
```
