# Supplementary material for: Evolution Analysis of the Fasciclin-Like Arabinogalactan Proteins in Plants Shows Variable Fasciclin-AGP Domain Constitutions
Source: Int J Mol Sci. 2019 Apr 20;20(8):1945. doi: 10.3390/ijms20081945 (PMC6514703; doi:10.3390/ijms20081945)
Supplement: Supplementary file 1 [file ijms-20-01945-s001.zip › Supplementary files/Figure S2.pdf]

Figure S2. Multiple sequence alignment of Type 1 fasciclin domain sequences. Rresidues with high similarity (100%, 80%, 60%) were highlighted in red, dark pink and light pink, respectively.

```
                      *        20         *        40         *        60         *        80         *       100
AtrFLA10.1 : -----------------QTLEEAVGK-HNITIFAPRNEALERDLDPEFK--RFLLEPGNLKSLQSLILFHVIPRRIPAKNWPESD--AHSHD---TLS----S :  74
AtrFLA11.1 : HEFSKFNDYLSR-THLASEINR----RTTITVLAVDNAAMDDILAK----------NYGIYEIKNILSLHILLDYFGAKKLHQITNGT-ALA----------- :  76
AtrFLA1    : ------------------------------TFLMPSDKDLANYN--FLP-----------GQLKELMLTHSIPNSMLFKHMLHFPTGT-LLP---SAR----- :  51
FLA16.1    : SHYTELAELVEK-ALLLQTLEEAVGK-HNITIFAPRNDALERNLDPLFK--SFLLEPRNLKSLQSLLMFHILPKRITSPQWPSLS---HHHR---TLS----N :  89
FLA17.1    : SRYTELAELVEK-ALLLQTLEDAVGR-HNITIFAPRNEALERDLDPEFK--RFLLEPGNLKSLQTLLMFHIIPNRVGSNQWPSEESGRVKHH---TLG----N :  92
FLA15.1    : SHYTELAELVEK-ALLLQTLEEAVGQ-HNITIFAPRNDALEKNLDPEFK--SFLLQPKNLKSLQSLLMFHILPKRITSPQFSSAV---VSHR---TLS----N :  89
FLA18.1    : SRYTELAELVEK-ALLLQTLEDAVGR-HNITIFAPRNEALERDLDPDFK--RFLLQPGNLKSLQTLLLSHIIPKRVGSNQWPEENSGRVKHV---TLG----H :  92
FLA1.1     : PSFSSFSHFLTQ-THLADEINR----RRTITVCAVDNAAMSALTSK----------GYTLSTLKNILSLHVLLDYFGTKKLHQIR------------------ :  70
FLA22      : ------------------------------TFLMPKDKTLSTSNIINQQ-----------DSVTEFLLRHSIPSSLLFEHLNLIPNGS-IVP---SSL----- :  53
FLA20.1    : ------------------------------TLFAPSDQSFSKFGQPS----------------LLDMKYQLSPTRLPGETLRNLPNGA-KIP---TLR----- :  48
FLA21.1    : ------------------------------TLFAIEDASFFNTSS-LHP-----------LFLKQLLHYHTLPLMLSMDDLLKKPQGT-CLP---TLL----- :  52
FLA4.1     : PNLSSFSNLLVS-SGIAAELSG----RNSLTLLAVPNSQFSSASLDLTR-------RLPPSALADLLRFHVLLQFLSDSDLRRIPPSG-SAVTTLYEASGRTF :  90
FLA2.1     : PDFSTFNHYLSA-THLADEINR----RQTITVLAVDNSAMSSILSN----------GYSLYQIRNILSLHVLVDYFGTKKLHQITDGS-TSTASMFQSTGSAT :  87
CreFLA9.1  : PNLTTYLRALRA-GELMGRLNDV-Q-APPVTLFVADDSAWQVFAADAGTSVDALLTEAGGALVRHLLLYGLVPTPLSPQQLAASPVLNTSWAGQMVFVPQPFM : 100
EgrFLA9.1  : SHYTELAELVEK-ALLLQTLEETVGR-HNITIFAPKNEALERDLDPEFK--SFLLEPRNLKSLQTLLMFHIVPSRIDSADWPEPAPSAAGHREELSLS----S :  95
EgrFLA5.1  : SHYTELAELVEK-ALLLQSLEDAVGR-HNLTIFAPRNEALERDLDPEFR--RFLLEPRNLRSLQTLLLSHIVPARLGSPQWPLPDSRPARHP---TLG----S :  92
EgrFLA10.1 : PDFSTFNHYLSA-THLAAEINS----RTTITVLAVSNAAMNDLLAE----------HPTTTTLKNILSLHVLLDYFGSKKLHQLT------------------ :  70
EgrFLA11.1 : PEFSTFNHYLTE-THLAAEINS----RTTITVMALDNAAMSRLLAP----------HPTIATLKNVLSLHVLLDYFGPKKLHQITNGT-ALAATLFQATGSAP :  87
EgrFLA16.1 : ------------------------------TVFSPADDSFARFGQPP----------------LSLLQFHFSPHALSLDTLRSLPRGA-KIS---TMN----- :  48
EgrFLA14.1 : ------------------------------TVFSPSDDSFAQFGQPP----------------LSLLQFHFSPHALSLDTLRSLPHGA-KIS---TMN----- :  48
EgrFLA15   : ------------------------------TVFSPSDDSFARFGQRP----------------LSLLQFHFSPHALSLDTLRSLPHGA-KIS---TMN----- :  48
EgrFLA19   : ------------------------------TFFMPDDGMLSRIV--LPT-----------NDVSHFVLRHSIPKPLLFDYLEHIPTGS-LIP---TSA----- :  51
EgrFLA12.1 : PEFSTFNHYLTI-THLAAEINR----RLTITVLAVDNAAMNAILDK----------HLSVGTLKNVLSLHVLVDYFGTKKLHQISNGT-TLTS---------- :  77
EgrFLA17.1 : ------------------------------TIFVIKDLFMANL-S-SSP-----------WLMEEVLRYHTSPLKLSVDDLLKMPRGS-CLP---TLL----- :  51
EgrFLA20.1 : ------------------------------TLFAPSDSSLKSCASCS---------------VPRILSEHIVPGLFSFGYIQKLAFGT-KVE---TMS----- :  49
OsaFLA17.1 : SRYTEMAELVEK-ALLLQALEDAVAE-HNVTILAPRNEALERDIDPEFK--RFLLEPRNLNSLQSLLLFHVLPARLPSGSWPA----AATHP---TLA----G :  88
OsaFLA24.1 : SHYTELAELVEK-ALLLQTLEDAVGK-HNVTIFAPRNEALERDLDPEFK--RFLLEPRNLKSLQSLLLFHVLPARLPSGSWSA-----VSHP---TLS----G :  87
OsaFLA16.1 : SHYTELAELVEK-ALLLQTLEDAVGK-GNVTIFAPRNEALERDLDPEFR--RFLLEPRNLRSLQRLLLFHVLPARLHASDSSSPDF-PSSHP---TLS----G :  91
OsaFLA12.1 : PSFADFARLLES-SPVAGELAA----RSSLTLLAVPNNNLPRSPSAFA--------AASGADIADVLRYHVLLEYLSPSDLARLPASG-KLVTTLFQTTGRAP :  89
OsaFLA11.1 : PEFSKFNEMLSK-TRLAYDINR----RQTITVLAVDNSAMASLDH------------FTLPTIRHILSLHILVDYYGSKKLHALSHGA-T------------- :  72
OsaFLA28   : ------------------------------TFLMPNDRMLSTAT--IPE-----------SQVMEFLSRHSIPAPLMFDDLIKLPNAT-IVP---TAH----- :  51
PabFLA22.1 : SRYTELAELVEK-ASLLQPLEEVVGN-NNITILAPQNAHLEKNLDPEFK--RFMHEPGNLNSLQRLLQSHIIPRRITAEEWSNNAT----HE---ALS----K :  88
PabFLA1    : ------------------------------TFFVPSDDALSRLPVQ--A-----------EFTSSILQYHIIPQQLSSYDLQRLDVGM-RLP---TLL----- :  51
PabFLA2    : ------------------------------TFFVPSDDALSRLPVQ--T-----------EFTSSILQYHIIPQQLSSYDLQRLDVGM-RLP---TLL----- :  51
PabFLA3    : ------------------------------TFFVPSDDALSRLPVQ--T-----------EFTSSILQYHIIPQQLSSYALQRFDVGM-RLP---TLL----- :  51
PabFLA4    : ------------------------------TFFMPSDQALAEVNL-QVT-----------SYISELVLRHSVHQPLKFNHLLHFPTGT-LIP---SSF----- :  52
PabFLA17.1 : SHYTELAELVEK-ALLLQTLEEVVGS-SNVTIFAPQNDILERKLDPEFK--RFLHEPGNLKALQKLLMFHVVPRRIVAEEWRNG-T----HQ---TLA----K :  87
PabFLA15.1 : PEFETFNKLLTD-TNVAKDINN----RTSLTILALPNSILENYMARFGG-------NLQVMEIESVLQYHVLLEYMDWDRLHQMTNRG-TL------------ :  78
PabFLA20.1 : PEFTTFNNLLTA-TNVAGEINN----RSSLTILALSNAQLEPFISSFGG-------NLPTQEVSDVLRYHVLLEYLDWERLKMMPAAG-TLVTTL-------- :  82
PabFLA21.1 : PEYSKFNHDLSQ-TKLADEINS----RQTITVLALSNSEMASLSG------------LDLASLKRVLSLHVVLDFFDAKKLHEITNGT-TLS----------- :  74
PabFLA16.1 : -DYSSFNSYLTQ-TKLADEINS----RTTITVLALNNGAMSVLTSK----------RLSLSVMKKVLSLHVLLDYFDGEKLHQISNGT-MLST---------- :  76
PabFLA18.1 : --FTSLGMHLQ--SSQ-DLL---VGLKGPLTLFAPTEEAFRYFNSPNSS---------SNSSSDYLLPYHIAKGLFTYTQLQMLPDGT-KLD---TIA----- :  77
PabFLA7    : -----FANALQM-VDI-THL---FSSGGSYTFFAPPDEALEALGSASKS---------W-QEFMDAVRYHIVNVQLSYYNLKHLPDGA-RLG---TLL----- :  74
PabFLA19.1 : -----------------NLL---VGRAGPLTIFAPTEEAFRNFNSSNSS---------SDLSSDYLLSHHIAKGLFTYAHLVMQPDGK-KLD---TIA----- :  65
PabFLA24   : ------------------------------TFLMPSDEKLAHAA----------------ASKVDLLMHHTIPRPLTFHDLLHFPTGS-FIP---SFL----- :  48
PpaFLA10.1 : SQYSEMVLYLDK-ADMLEELEREVLRQKAITLFAPKNSHLEQLD-ADLG--RFLMRPGHEEYLRTVLRYHVIPGRVEGADFQNRTV-----E---TLS----K :  87
PpaFLA11.1 : SQYSEMVLYLDK-ADMLEELEREVLRRGAITVFAPKNSYLEQQVDADLW--RFLMRPGHEAYLRTLLKHHVIPGRVEGADFQNRTV-----E---TLA----S :  88
PpaFLA6    : ------------------------------TLLAPNDNAFSNVLMN-ST-----------TYLTTLLTYHGAAKVYSYEGLLNLPVGT-KIP---STA----- :  52
PpaFLA7    : ------------------------------TLFAPDDGAFSGLNLN-SS-----------KLLMTTLDYHVATSVYNYNQLSTLPLNS-TIK---TSV----- :  52
PpaFLA8    : ------------------------------TLFAPDDGAFSGLNMN-SS-----------SLLMTTLDYHVATSVYNFNQLSNLPLNS-TIK---TSA----- :  52
PpaFLA9    : ------------------------------TFFAPDDGAFSGLNMN-NS-----------MLFMNTLHYHVATAVYSYQQLSYLPLNS-TIQ---TAA----- :  52
PpaFLA4    : AGLTSSADAIQA-AVISGSI---TLPSSNFTIFAPVNQAVDSILPS--N-----------PDLSKVLNYHLATTDLPHKALLLLPIGQ-RIP---TLL----- :  77
PpaFLA12   : ------------------------------TIFAPNDGALSDFHKRKTQ-----------EHLENLVRFHIITTPLPFSNLLRMEAGS-RLK---TAV----- :  53
PtFLA1.1   : SHYTELAELVEK-ALLLQTLEDAVGK-HNITIFAPKNEALERDLDPEFK--RFLLEPGNLKSLQTLLLYHIVPNRINPSHNSSLQHHS-------TLC----R :  88
PtFLA19.1  : SHYTELAELVEK-ALLLQTLEDAVGK-HNITIFAPRNEALERDLDPEFK--RFLLEPGNLKSLQTLLLYHIVPNRINLSHNSSLHHHS-------TLC----R :  88
PtFLA6.1   : SHYTELAELVEK-ALLLQTLEEAVGK-HNITIFAPRNEALERQLDPEFK--RFLLEPGNLKSLQTLVLFHIIPQRVGSNDWPGHKSNPSRHT---TLC----N :  92
PtFLA8.1   : SHYTELAELVEK-ALLLQTLEEAVGK-HNITIFAPKNEALERQLDPEFK--RFLLEPGNLKSLQTLLLFHIIPQRVGSNDWPGHKSNPTRHT---TLC----N :  92
PtFLA5.1   : PSFSTFNHYLTL-THLAGEINS----RNTITVCAVDNAAMSELLSK----------HPSIATIKNILSLHVLLDYFGTKKLHQIREGT-ALA----------- :  76
PtFLA22.1  : PSLSTFNHYLTL-THLAGEINR----RTTITVCAVDNAAMSEILSK----------KPSISTIKNILSLHVLLDYFGTKKLHQIRDGT-ALA----------- :  76
PtFLA42.1  : ------------------------------TIFAPLDIAFARLGQLS----------------VLDLQYHISPVRLSGYYLDSLPFGT-RIP---TLL----- :  48
PtFLA51    : ------------------------------TFLMPKDRLLSKIR--MHQ-----------NAVSDFLLHHSIPSPLLFDHLRHIPPGS-LIP---SSD----- :  51
PtFLA52    : ------------------------------TFFIPSDNSLSPTT----T-----------SADPDIFPYHIVPQRLSFADLQQFKTFS-RLP---TLL----- :  49
PtFLA53    : SHYTELAELVEK-ALLLQTLEDVVGK-HNITIFAPKNEALERDLDPEFK--RFLLEPGNLKSLQTLLLYHIVPNRINPSHNSSLQHHR-------TLC----R :  88
PtFLA41    : SHYTELAELVEK-ALLLQTLEDAVGK-HNITIFAPKNEALERDLDPEFQ--RFLLEPGNLKSLQTLLLYHIVPNRINPSHNSSLQHNS-------TLC----R :  88
PtFLA38.1  : PDFSSFTSLITSIPSLTSDLSD----RSALTLLVIPNSYLSSS-LDLTR-------RLSPSALADLLRYHILLQYLSSSDLHQVPPSG-ALITTLFQTTGRAS :  90
PtFLA30.1  : PDFSSFTSLLASTPSITSDLSN----RSALTLLVIPNSYLSSS-LDFTR-------RLSPSSLTDLLRYHILLQYLSSSDLHQLPPAG-ALITTLFQTTGRAS :  90
PtFLA16.1  : PQFSTFNHYLTV-THLAAEINR----RQTITVLALDNAAMSSLISK----------QLSVYTLRNVLSLHVLVDYFGTRKLHQITNGT-ELTATMFQATGSAP :  87
PtFLA4.1   : ------------------------------TIFAIKDSSLVNT-S-LPP-----------WFLKNLLQYHTSPLKLSMEDVFKKPQGS-CFP---TLV----- :  51
PtFLA44.1  : ------------------------------TLFAPSDSSLRTCFSCS---------------IPDLLHEHIVPGLFSIDYLRKLAFGT-KIE---TLS----- :  49
PtFLA24    : ------------------------------TFFIPSDNSLSPTT----T-----------SADPDIFPYHIVPQRLSFADLQQFKTFS-RLP---TLL----- :  49
SmoFLA1    : ------------------------------TLMIPTNEAIAAFVN-LNQ-----------SEIPSLLLYHAVTGVLPYDVLSEFPVGQ-RLP---TLL----- :  52
SmoFLA2    : ------------------------------TLLIPSNNAIAGVDANLSQ-----------EDIINTLQYHVLTFPTSFEALSRNDVGA-ELP---TML----- :  53
SmoFLA6.1  : DHLSTFNRYLSE-TGVIKEVDS----RSSITLFIPPNAVLDPVIASVGA--------NNLLVIADILRYHCVLQYLEIKEIRSMTNAT--------------- :  75
SmoFLA7.1  : PDFSTFSKLLTD-TGVISEINQ----RSSLTLLVPHNSALDAVLNRSSPSS----TPLPLPVVADVLRYHCLLQYLDVPQIKSMTN----------------- :  77
SmoFLA8.1  : -NFSTFSRLLSM-TNVTAEINS----RSSLTILVPDNSILDSYVGANLE-------GMHVWAVADFCRYHVLLQYLDTQEIMQMTNQS-GL------------ :  77
SmoFLA9.1  : ---TEMMELVEQ-AGMLEALELAADR-HNLTIFAPRDEFLELHFDADFR--RFLLLPGNVRFLQELVMFHVLPIRITASQWRSGRF-----Q---SLS----G :  84
BdiFLA23.1 : SHYTELSELVEK-ALLLQTLEDAVGR-HNVTIFAPRNEALERDLDPEFK--RFLLEPRNLKSLQSLLLFHVLPSRHPAASWPA----SAAHP---TLS----G :  88
BdiFLA22.1 : SRYTELSELVEK-ALLLQQLEDAVGR-GNVTIFAPRNEALERDLDPEFR--AFLLQPRNLRSLQRLLLFHVLPSRLSSSQWRS-----AASL---TLS----G :  87
BdiFLA2    : ------------------------------TFLMPSDRLLSTAS--VPG-----------NQVLDLLLRHSVPAVLMFADLNRLPNGT-VVP---TRH----- :  51
BdiFLA20.1 : PNFSDFLRLLTS-SPVAGELTG----RSSLTLLAVPNANLPRSPSAFA--------AASGADLADVLRYHVLLEYLSPSDLARLPASG-KLVTTL-------- :  81
MpoFLA6.1  : PDFSIFNQLLTD-TRVAGEINR----RQGVTVLAPSNEAMTDFRSA-NP-------ALEGDRVANLLRYHVLLDFLTIQNLQRLDIANYTRVGTLFRTTGPAN :  90
MpoFLA14.1 : SQYSEWMELVEQ-AAMLHPLEELVGSGRKLTIFAPQNHQLQ----PKIK--SFLLKPSNAIALKRVVQYHVLSERVTASEWPTASF-----T---TLA----S :  84
MpoFLA4    : ------------------------------TLFVPTNEALSSLSM---V-----------TPVLNILLYHAATPQYTFDQLVTLPVGT-RLQ---SFL----- :  50
MpoFLA5    : ------------------------------TWLLPNDEAFSGTSY---P-----------KNVTKFIDYHVIRQLLPYSRLSTLSVGT-RLP---TFL----- :  50
MpoFLA8.1  : PRFSVFREMLSS-HGVAAEING----RKSVTLLAPANNVMRAFRTS-NN-------KTDAIKITDLLRFHVLLTYFDMAELRALKTTNYTSVTTLLQTTGRAS :  90
MpoFLA9/10 : PSFSEMNKLLSS-SGVAEEINS----RKSLTLLALSNDVLTAFTAS-NP-------NVDAVKLADLLRYHVLLQFLGMDDLKALPTDNYTAVTTLYQTTGRAN :  90
MpoFLA11.1 : PSFSQMNQLLSS-SGVANDINA----RKSITVLVVSNDVLNAFTAS-NP-------NVDTVKMADLLRYHVLLQFLGMDELKALPTSNYTSVTTLYQTTGRAN :  90
MpoFLA12.1 : PSFSQMNQLLSS-SGVANDINA----RKSITVLVVSNDVLNAFTAS-NP-------NVDTVKMADLLRYHVLLQFLGMDELKALPTSNYTSVTTLYQTTGRAN :  90
MpoFLA13.1 : PSLSAMNQLLTS-SGVAAEINS----RTSLTLCAVSNPVLQAFVAT-VP-------NVDSGEVADLLRYHVFLQYLDIPELKQIVPGSPSTVTTLLQTTGRTA :  90
                                            T                                      h
```

```
                   *       120         *       140         *       160         *
AtrFLA10.1 : DRL-T---LTCK-----------------NRVRKVD--------LASLIHPDSIVRP----DGVIHGIERLLIP : 115
AtrFLA11.1 : -------------------------------------------------------------------------- :   -
AtrFLA1    : -HSRFLNVSNHG-----------------SYSFYL--------NNARIVAPNVCGST----NIRCHGLSTIIEY :  95
FLA16.1    : DHL-H---LTVD-----------------VNTLKVD--------SAEIIRPDDVIRP----DGIIHGIERLLIP : 130
FLA17.1    : DQV-R---LSNG-----------------QGKKMVD--------LAEIIRPDDLTRP----DGLIHGIERLLIP : 133
FLA15.1    : DHL-H---FTN---------------------GKVN--------SAEITKPDDLTRP----DGIIHGIERLLIP : 126
FLA18.1    : DQVLHLSKLKGT-----------------NGKRLVN--------SAVITRPDDLTRP----DGLIHGIERLLIP : 137
FLA1.1     : -------------------------------------------------------------------------- :   -
FLA22      : -PHYTLEISNGG-----------------RSNYFL--------NNVKIISRNICSLG----SIKCHGIDGILQS :  97
FLA20.1    : -SNYSLTVTNSS---------------RFGGKTSI--------NNVVVQDSPVFDDG----YVVIYGSDE---- :  90
FLA21.1    : -HHKSVQISTVN---------------QESRTAEV--------NHVRITHPDMFLGD----SLVIHGVIGPFSP :  98
FLA4.1     : FGSGSVNV------------------------------------------------------------------ :  98
FLA2.1     : GTSGYINITDIK-----------------GGKVAFGVQDDDSKLTAHYVKSVFEKPY----NISVLHISQVLTS : 140
CreFLA9.1  : GAVSSSAGTE-----------------------------------ARIT--GRVKVC----ESYAYRVAYVLMP : 133
EgrFLA9.1  : HRL---RLAKRE-----------------SGEREVD--------RARVIHADAVARP----DGVIHGIERLLIP : 137
EgrFLA5.1  : DPV-HLS-VAPG-----------------SGLPAVD--------SAEVVRPDDVVRP----DGVIHGIQRLLIP : 135
EgrFLA10.1 : -------------------------------------------------------------------------- :   -
EgrFLA11.1 : GSTGFVNITD---------------------------------------------------------------- :  97
EgrFLA16.1 : -ANHSLMVTTSP----------------SDYQVEI--------NGVKIVGSPVFNDG----NLVVFGI------ :  87
EgrFLA14.1 : -ANHSLMVTTSP----------------SDYQVQI--------NSVKIIGSPVFNDG----NLVIFGI------ :  87
EgrFLA15   : -ANHSLMVTTSP----------------SDYQVQI--------NGVKIVGSPVFNDG----NLVVFGI------ :  87
EgrFLA19   : -AGYLVRIDNKG-----------------CRSYFL--------DDVRITKPNVCVAGS---SIRCHGIDGVLLP :  96
EgrFLA12.1 : -------------------------------------------------------------------------- :   -
EgrFLA17.1 : -AKKNVTVTRSD---------------AEQRLVEI--------NGAVITHPNVFLGE----RLAIHGVLQPLL- :  96
EgrFLA20.1 : -PGHCLTVTSATHR------------VTNTTRIFI--------DGVEISRPDMFNNG----IVVVHGLEGFIAP :  98
OsaFLA17.1 : EDV-ELAAADGG-----------------GGMRVA---------HAAVSRPDAVVRT----DGVIHGIDRLLVP : 131
OsaFLA24.1 : EEV-ELAAAAND-----------------GAMRVA---------HAAVTRPDAVLRP----DGVIHGIERLLVP : 130
OsaFLA16.1 : ERV-DLSA---------------------SPMRVG---------AAAVTRPDAVVRP----DGVIHGIERLLVP : 130
OsaFLA12.1 : SDLGAVNLTVGG-----------------NST-VVVRS------------------------------------ : 109
OsaFLA11.1 : -------------------------------------------------------------------------- :   -
OsaFLA28   : -SSQTITITNVK-----------------HQKIYF--------NNIELISPDVCRVGD---LFRCHGINGVIRP :  96
PabFLA22.1 : DGL---QFSVKE-----------------NNIKAVD--------LAVLTRPNAVVRP----DGVIHGIDRLLVP : 130
PabFLA1    : -ANQTIVVTSNT-----------------QPNYTV--------DDTLVTHPDIFYDG----TFAVHGVADLL-- :  93
PabFLA2    : -ANQTIVVTSNR-----------------QPNYTV--------DDTLVTHPDIFYDG----TFAVHGVADLL-- :  93
PabFLA3    : -ANQTILVTSNR-----------------QPNYTV--------DDTLVTHPDIFYDD----TFAVHGVADLL-- :  93
PabFLA4    : -PNYMLKVSNNG-----------------RNDFYL--------NYVHIVHPNVCRASP---SISCHGVSGVL-- :  95
PabFLA17.1 : DRL---KLSMKG-----------------DKKTI-D--------LASVAHPNAVVRP----DGVIHGIDRMLIP : 128
PabFLA15.1 : -------------------------------------------------------------------------- :   -
PabFLA20.1 : -------------------------------------------------------------------------- :   -
PabFLA21.1 : -------------------------------------------------------------------------- :   -
PabFLA16.1 : -------------------------------------------------------------------------- :   -
PabFLA18.1 : -SEGSLLVTFNSRGDGQDLIYSSAQTLVDYRPVYV--------NGVLVSHPDLLNDG----LLTIHGVDQPL-- : 136
PabFLA7    : -RSRSLTFTTFAPE-------------SENETLMV--------NNVSVSFPDL--------------------- : 105
PabFLA19.1 : -SVGSIVVTFNSHGDLS----SSTQLRAEYRPVYL--------NGVLLSHPDLLSDG----LLTIHGVDQFLV- : 121
PabFLA24   : -PDYMYSVANDG-----------------KNNFSL--------NNVKIVTPDLCNGSP---AIQCHGISSVI-- :  91
PpaFLA10.1 : GDVVG---LRTY-----------------GLKRYVG--------LLRVFSPNSIVRK----DGIVHGVDGLMVP : 129
PpaFLA11.1 : GNVVG---LRSH-----------------GLKRYVE--------SARVFSPNSIVRK----DGIVHGLDGFMIP : 130
PpaFLA6    : -ANVVIVVTSNS-----------------KGAYKL--------DDSQIVDPDIFVDN----TVAVHGIDNVL-- :  94
PpaFLA7    : -PDVVILITSTG-----------------TSGLRL--------DNVAISDPDLYVDS----QIAVHGISAVM-- :  94
PpaFLA8    : -PNVEIFVTSTG-----------------TDGLRL--------DNVAISDPDLYADG----QMAVQGISSVM-- :  94
PpaFLA9    : -PNVVMYITSTG-----------------EDGLML--------DNVVISDPDLYLDD----KVAVHGISMVM-- :  94
PpaFLA4    : -PDNTLLVTDNN-----------------LANYSI--------NNEQILYFDL--------------------- : 104
PpaFLA12   : -SNFTILVTNTT-----------------KDAYQV--------DDATIIDPDLYTGA----TIAVHGINAV--- :  94
PtFLA1.1   : DRV---KLSSQE-----------------SGEKLID--------SAKIIQVNAVERP----DGVIHGIERLLIP : 130
PtFLA19.1  : DRI---KLGSQ------------------SGEKLID--------SAKIIQVNAVERP----DGVIHGIERLLIP : 129
PtFLA6.1   : DHL---HLITKN-----------------SGKKVVG--------SADVTRPDDVTRP----DGVIHGIERLLVP : 134
PtFLA8.1   : DHL---HLITKN-----------------SGKKLVG--------AAVLTRPDDVTRP----DGVIHGIERLLVP : 134
PtFLA5.1   : -------------------------------------------------------------------------- :   -
PtFLA22.1  : -------------------------------------------------------------------------- :   -
PtFLA42.1  : -PNHSLIVTTSLS--------------YFDGKLSI--------NGISIEESALVDFG----SLIIFGMSE---- :  91
PtFLA51    : -PDYMLNISNEG-----------------RKSFFL--------NNVKISSPDLCTAGS---SIRCHGIDGVLL- :  95
PtFLA52    : -FDKSILITNNS-----------------ASNFTL--------DGSRLTHPDIYTNA----AITVHCIDNLL-- :  91
PtFLA53    : DRV---KLSSQE-----------------SGEKLID--------SAKIIQVNAVERP----DGVIHGIERLLIP : 130
PtFLA41    : DRV---KLSSQE-----------------SGEKLID--------SAKIIQVNAVERP----DGVIHGIKRLLIP : 130
PtFLA38.1  : SNSGSVNITRNP-----------------VTN-A---------------------------------------- : 106
PtFLA30.1  : SNFGSVNITRNP-----------------VTN-A---------------------------------------- : 106
PtFLA16.1  : GASGYVNITDLN-----------------GGKVA---------------------------------------- : 104
PtFLA4.1   : -DRKKLAVTKID---------------AKERLAEI--------NHVLVSHPDMVLER----RITIHGV------ :  91
PtFLA44.1  : -PGRCITVTSTSLKNDS--------ATPSTVKVFI--------GGVEITHPDLFNNG----VLIIHGIQGYIAP : 102
PtFLA24    : -FDKSILITNNS-----------------ASNFTL--------DGSRLTHPDIYTNA----AITVHCIDNLL-- :  91
SmoFLA1    : -FGNQLVVTDNS-----------------AAGYRL--------NTARIVRPNMCGNSTS--VVTCHGIDRVLNP :  98
SmoFLA2    : -QGEMITVTSNS-----------------PGNFTL--------NEVNITHPDVCSSTR---FIACHGIDRVL-- :  96
SmoFLA6.1  : -------------------------------------------------------------------------- :   -
SmoFLA7.1  : -------------------------------------------------------------------------- :   -
SmoFLA8.1  : -------------------------------------------------------------------------- :   -
SmoFLA9.1  : SRD-EVLLHWNK-----------------KQRLAVD--------RSTVDWPDMIVRS----DGVVHRIDGLLVP : 128
BdiFLA23.1 : EDL-ELAAG-SN-----------------GSMRVA---------HASVTRPDAVLRP----DGVIHGIERLLVP : 130
BdiFLA22.1 : EHL-ELSLSDSS-----------------TTAMLAG--------SAAVTHPDAVVRP----DGVIHGIEKLLIP : 131
BdiFLA2    : -TNQMVTITKRE-----------------HRQLYF--------NNIELTSPDICRGGD---SFRCHGINGVLRP :  96
BdiFLA20.1 : -------------------------------------------------------------------------- :   -
MpoFLA6.1  : ADDASVNLYNTP-----------------SNI-FIGSSAVDSSQNSTVISTVFLQPS----DVSVIMIDRTLQP : 142
MpoFLA14.1 : EAV-W---LSTQ-----------------GPILMAG--------DVAVAAPDAIIRP----DGVVHGVEEMLIP : 125
MpoFLA4    : -ANESVLITNNA-----------------RNNFQV--------DNVRIVLANVCASNTTDLQISCHGVESIL-- :  96
MpoFLA5    : -GSETVVVTSNL-----------------RFNYSL--------DNAMIVVPDLYSDS----TVAVHGINSVLN- :  93
MpoFLA8.1  : GNNGFVNIYNLK-----------------SRV-KLGPSVPDSSSNVTVLAGVQKSPF----DVSIIEIDQVLLP : 142
MpoFLA9/10 : DNDGFVNIYN---------------------------------------------------------------- : 100
MpoFLA11.1 : NNDGFLNIYN---------------------------------------------------------------- : 100
MpoFLA12.1 : NNDGFLNIYN---------------------------------------------------------------- : 100
MpoFLA13.1 : GNDGSVNI------------------------------------------------------------------ :  98
```
