# Supplementary material for: Evolution Analysis of the Fasciclin-Like Arabinogalactan Proteins in Plants Shows Variable Fasciclin-AGP Domain Constitutions
Source: Int J Mol Sci. 2019 Apr 20;20(8):1945. doi: 10.3390/ijms20081945 (PMC6514703; doi:10.3390/ijms20081945)
Supplement: Supplementary file 1 [file ijms-20-01945-s001.zip › Supplementary files/Figure S3.pdf]

Figure S3. Multiple sequence alignment of Type 2 fasciclin domain sequences. Residues with high similarity (80%, 60%) were highlighted in dark pink and light pink, respectively.

```
                       *        20         *        40         *        60         *        80         *       100         *       120         *       140         *       160
AtrFLA10.2 : ---------------------------------------TVLAPNDEAMAQLT--ADQ---------------------------------LSEPGAPEQIVYYHVIPEYQ-TEESMYNAVR------R-FG-KLQYETLRL------PHKLVAEEA--D------GS :  71
AtrFLA11.2 : ---------------------------------------TAFCPTDIWTKFV--PKYK--------------------------------NLSADHKVSLLLFHGVPIYQ-SMQGLKSNN------GP-MN-TLATDGAA-----S--YSVTVQND---G-----EQ :  71
AtrFLA5    : ---------------------------------------TIFAPDDASIFSG---------------------------------------GYAYVNNVRFHIVPDRYLKGSDLAQLH---L-GSS-LQ-TLVRGQEL---------VVTNAGS--S----V--- :  63
AtrFLA2    : -NFNFLVSMIEAS-----GVVYEFE---RGE---VGAGITIFAPDDDAFSLL-NPETLQ---------------GL-----------------TADKKALILKYHVLLSYY-PLGSLQTILN------P-VQPTLATEEI-GA--GTYTLNITRLR----G-----N- : 102
AtrFLA7    : GPFHLFLEKLEST-----KVIDTFQNQANNT----DQGITIFAPKDKAFSSL-KKPS-----------------------------------LSNLTSDQLKSLMLYHAIPEYY-SLADFKNLS-------Q-QGPTS---TFAGG---LYALNFTDN-----K-----GS : 101
AtrFLA3    : GQFTVLIRLLQST-----QVANQITNTLNN----SNQGLTIFAPTDNAFNSL-PPGT-----------------------------------LNSLSQQDQVTLILYHVLPQFY-SLQGFQTLSN------P-VR-TQASGMG--G--GQYTLNVTTVAS---S-----MQ : 105
AtrFLA8    : GQYTMLIRLMKAT-----QVADQMNQQLNN---S-AQGLTIFAPTDNAFSSL-KPGT-----------------------------------LNSLTDQQKVSLIQFHIIPSFI-SVPQFQTVTN------P-LR-TQAGSGE-----DQFPLNVTTSGN----------Q : 102
AtrFLA9    : AGCKIFASLLAS-S----GVLKNYQD---A----MDKGLTILAPNDEAFKKPE-VPDTA-----------------------------------GMRVAQLVSLLEYHALPSYS-PPGALKAAT------GP-VS-TLAS-GVR-----K--FAFRVATS---G-----DE :  98
AtrFLA4    : ---------IAAS-----GVAAEFE---SDE---QGAGITVFAPTEEAFSAL-PPP------------------------EK-------LQDLSADKKAVVLKYHVLHCYY-PLGSLESIVN------P-VQPTLATEQK-GA--GQFTLNITRAN----N-----T- :  95
AtrFLA6    : -----------------LTELLSI---------AGSFL------------------------------------------LFLEK----LPKPGQQQLKSLMLYHAIPKCH-SLAEFKNLSE----QGP-TS-TFA---GCQ--------YALNF-TE--NK------- :  69
AtrFLA12   : ---------------------------------------TIFALDDASIFSG---------------------------------------GHSYVNNVRFHIVPNRYLKGSDLAQMN---M-GSS-LR-TLIQGQDL---------VVTNPGL--G----V--- :  63
FLA16.2    : ---------------------------------------TVLAPNDEAMAKLT--TDQ---------------------------------LSEPGAPEQIMYYHIIPEYQ-TEESMYNAVR------R-FG-KVKYDSLRF------PHKVLAQEA--D------GS :  71
FLA17.2    : ---------------------------------------TVLAPNDEAMAKLT--TDQ---------------------------------LSEPGAPEQIVYYHIIPEYQ-TEESMYNSVR------R-FG-KVKFDTLRF------PHKVAAKEA--D------GS :  71
FLA15.2    : ---------------------------------------TVLAPNDEAMAKLT--TDQ---------------------------------LSEPGAPEQIMYYHIIPEYQ-TEESMYNSVR------R-FG-KIRYDSLRF------PHKVEAQEA--D------GS :  71
FLA18.2    : ---------------------------------------TVLAPNDEAMGKLT--TDQ---------------------------------LSEPGAPEQIMYYHIIPEYQ-TEESMYNSVR------R-FG-KVKYETLRF------PHKVGAKEA--D------GS :  71
FLA1.2     : ---------------------------------------TVFCPGDDAMKGFL--PKYK--------------------------------NLTAPKKEAFLDFLAVPTYY-SMAMLKSNN------GP-MN-TLATDGAN-----K--FELTVQND---G-----EK :  71
FLA20.2    : ---------------------------------------TVFAPIDEAIPNP----TT-----------------------------------KFSDYVTIFRGHVVSQL-LLWKDLQKFA--KE-GSI-LQ-TVLKGYEI---------EISLSG----------- :  63
FLA21.2    : ------------------------------------------------------------------------------------------------FLYEVVRHHILVQR-LTYKDFASMS---D-KAT-VK-TLDPYQDL---------TITRRNV--N-------S :  47
FLA19      : ---------------------------------------TLFAPTDSMLFDLD--MTH-----------------------------------SLPFYVSTLRLHSVPLR-LSLSGLRSLP---N-SSS-LP-TLLPSHRL---------LLTKH-----------SS :  65
FLA4.2     : HNFNVALSLLVAS-----GVITEFE---NDE---RGAGITVFVPTDSAFSDL-PSNV------------------------N--------LQSLPAEQKAFVLKFHVLHSYY-TLGSLESITN------P-VQPTLATEEM-GA--GSYTLNISRVN----G-----SI : 105
FLA10      : AGCKTFANLLVS-S----GVIKTFES---T----VEKGLTVFAPSDEAFKARG-VPDLT---------------------------------NLTQAEVVSLLEYHALAEYK-PKGSLKTNK------DA-IS-TLATNGAG-----K--YDLTTSTS---G-----DE :  99
FLA8       : AGCKTFANLLVS-S----GVLKTYES---A----VEKGLTVFAPSDEAFKAEG-VPDLT---------------------------------KLTQAEVVSLLEYHALAEYK-PKGSLKTNK------NN-IS-TLATNGAG-----K--FDLTTSTS---G-----DE :  99
FLA11      : GQFTLFIRLLKST-----QASDQINTQLNS---SSSNGLTVFAPTDNAFNSL-KSGT-----------------------------------LNSLSDQQKVQLVQFHVLPTLI-TMPQFQTVSN------P-LR-TQAGDGQ--N--GKFPLNITSSGN----------Q : 104
FLA12      : GQFTVFIRLLKST-----GVANQLYGQLNN---S-DNGITIFAPSDSSFTGL-KAGT-----------------------------------LNSLTDEQQVELIQFHVIPSYV-SSSNFQTISN------P-LR-TQAGDSA--D--GHFPLNVTTSGN----------T : 103
FLA7       : GPFHTFLDYLLST-----GVIETFQNQANNT----EEGITIFVPKDDAFKAQ-KNPP-----------------------------------LSNLTKDQLKQLVLFHALPHYY-SLSEFKNLS-------Q-SGPVS---TFAGG---QYSLKFTDV-----S-----GT : 101
FLA9       : GQFTTFIHLLNIT-----QVGSQVNIQVNS----SSEGMTVFAPTDNAFQNL-KPGT-----------------------------------LNQLSPDDQVKLILYHVSPKYY-SMDDLLSVSN------P-VR-TQASGRD-N---GVYGLNFTGQ-----T-----NQ : 103
FLA6       : HQFTTLIQLLNTT-----QVGFQVSVQLNS----SDQGMTIFAPTDNAFNKL-KPGT-----------------------------------LNSLTYQQQIQLMLYHIIPKYY-SLSDLLLASN------P-VR-TQATGQD--G--GVFGLNFTGQAQ---S-----NQ : 105
FLA13      : GQFVTLIRLLNTT-----QIGNQINIQINS----SSEGMTVLAPTDNAFQNL-KPGT-----------------------------------LNKLSPDDQVKLILYHVSPKFY-TLEDLLSVSN------P-VR-TQASGRD-VG--GVYGLNFTGQ-----G-----NQ : 104
FLA14      : DDFSNFNQLLSET-----QLASTINKR---------QTITVLVVSNGALSSLS-------------------------------------GQPTSVIKKILSLHIVLDYY-DQKKLKNLSKK---TVL-LT-TL------------------------------------- :  77
FLA3       : PEFSTMTELLAKT-----ELTPIINKR---------QTITVLALNNDAIGSIS-------------------------------------GRPEEEVKNILMNHVVLDYF-DELKLKALKEK---STL-LT-TLYQSTGLGQQQNGF-LNCTK-SN----------GK :  99
FLA5       : SKFSTMRDLFIKT-----KLIAAIDKY---------QTITVLAVSNDAISSIT-------------------------------------NRSEVELRNILMTHVILDYY-DELKLQGMREK---SIM-LT-TLYQTTGLGEQMNGF-LNVSK-SK----------GR :  99
FLA2.2     : ---------------------------------------TVFCPSDSAVGKFM--PKFK----------------------------------SLSPANKTALVLYHGMPVYQ-SLQMLRSGN------GA-VN-TLATEGNN-----K--FDFTVQND---G-----ED :  71
CreFLA11.1 : ---------------------------------------TVFLPNDEAFKLLL--KAL------------------------GVTQAQ----LLALPTKVLTEVIVYHAVPGMPLAAKDLKPKQV--------VE-TALP--GYN--------LTITK-DS--AG------V :  75
CreFLA11.4 : -------QLIALT-----GLAEPLSDP--------DIEYTLFAPVNDAVTTFA--AELQKIQTY---------------NPAN---------PQVLQLLYQILATHVYMAGVLRSSDLTDGQV--------IR-TGVA--NTPA--TN--VTVTI-NS--T-------G :  98
CreFLA1    : ----IAIQALNAT-----GVSGKLNA---------STVATIFAPDDAAFISLA--SSLNFTSRE-------------QV------------FASPFATNVTLLHITPGVALRSTDLPAGQTVNASSLL----------GPT-------LEVTR-NG--NA---V-RV :  98
CreFLA10.2 : PDLSIVVISLK-SL----GQETLLRNA--------SLDYTVFALTNDAFDNFA--KVIGLNGTADIYQRPD-------------------LLTAVIETQWYHMVPQVF-RVGDQPLNSTSYF-QTR-LA-TVLGKVGN--------YSRVGIQRGGAA-----DP : 114
CreFLA9.2  : GNLTTTLGALQVT-----GLEDQLYAP--------GVAPYTLFAPTDAAWEEAA--AQLQLAPGK---------------DV-------------LQQ-----L-------------------------------------------------------------------- :  56
CreFLA8.1  : -------------------YSLLSDT---------GLKATVFLPTSDAVMSYL--YTEFG----------------VSDLADLWSNAVLNQRLLGEFNLRVLPQMIVKDR-LLTAQQMSNG------MK-LR-TNSSEEVT--------LVVSKAA---D------GT :  96
EgrFLA9.2  : ---------------------------------------TVLAPNDEAMAKLT--TDQ---------------------------------LSEPGAPEQIMYYHLIPEYQ-TEESMYNAVR------R-FG-KVKYDTLRL------PHKMVAQEA--D------GS :  71
EgrFLA5.2  : ---------------------------------------TVLAPNDEAMAKLT--TDQ---------------------------------LSEPGAPEQIVYYHIIPEYQ-TEESMYNAVR------R-FG-KVRYDTLRL------PHKMVAQEA--D------GS :  71
EgrFLA10.2 : ---------------------------------------TAFCPLDDPFKAFR--PKFD--------------------------------NLSAADRASFLEFFATPVYM-SMSMLRSNN------GL-MN-TLATDGSR-----K--FDFTVQND---G-----QD :  71
EgrFLA11.2 : ---------------------------------------TTFCPLDDPFKQFL--PKYK--------------------------------NLSAPGKASFLEFFAIPMYL-SMAMLKSNN------GP-MN-TLATDGAS-----K--YDFTVQND---G-----EE :  71
EgrFLA16.2 : ----------------------LKGD---------NQMLTVFTPSDEAMVNH----TG-----------------------------------SFSDYPSIFLRHVIPCK-LSSSDLLEFN---N-GIL-VH-THLEGFVI---------EVTRSG----------- :  70
EgrFLA14.2 : KGYSVMAS---FLG----LQLMKPKGD--------NQMLTVFAPFS--------------------------------------------------EYPSVFLRHVVPCK-LSASDLLEFN---N-GTL-LH-THLEGFVI---------EVTRIG----------- :  75
EgrFLA4    : HNFNVAASMLAAS-----GVVEEFQ---ADE---GGAGITLFVPTDSAFTEL-PSSQ-----------------------S--------LQSLPADKKAVVLKFHVLHSYY-PLGSLESIVN------P-LQPTLATEDM-GA--GSYTLNISRVN----G-----S- : 104
EgrFLA12.2 : -GCKSFADLISAS-----GALKTFDD---N----LEGGLTIFCASDAAVSAFM--PKYK--------------------------------NLTNAQKVSFVLYHGVPVYQ-SLQMLKSNN------GV-MN-TLATDGAS-----K--YDFTIQND---G-----ED :  97
EgrFLA8    : GSFHTFLGYLEST-----KVIDTFQEQANSS----KDGITIFAPTDKAFSSL-KQPS-----------------------------------LSNLTQDQLKSLCLFHALSRYY-SLADFKNLS-------Q-SSPVS---TYAGG---QYTLNFTDL-----S-----GD : 101
EgrFLA7    : GPFQTFLSYLEST-----KVIDTFQNQANNT----EEGITLFVPKDGAFASQ-KKPS-----------------------------------LSNLTNDQLKSLCLFHALPHYY-TLADFKNLS-------N-VSPVS---TFAGG---EYSVNFTDV-----T-----GT : 101
EgrFLA13   : PDFSTFNDFLTQT-----KLYEQINRR---------QTITVLALDNSAVGDIS-------------------------------------GKPADVLKSILSAHVVLDYY-DIEKLTDLSKK---STL-------------------------------------- :  73
EgrFLA6    : GQYTSFIRLLTTT-----QVANQIENQLNT----STEGLTVFAPTDNAFNNL-KAGT-----------------------------------INDLSNEQQVQLVLNHVAPKYY-SLLSFQSVSN------P-VR-TQ--------G--NFAGLNFSSQ-----G-----NQ :  98
EgrFLA1b   : SQFTVLLKLMRAT-----SVDKQINAQLNN---S-NNGLTIFAPNDNAFSSL-KSGT-----------------------------------LNQLSSEQQVELVQFHVVPTYL-AVPQFQTVSN------P-LR-TQAGGSG--K--FEFPLTLTTSGS----------S : 103
EgrFLA3b   : ------------T-----NLSSQLS---NQ---KSNQGFTIFAPTDNAFASL-KSGT-----------------------------------LNMLSDQEKTQLVQFHIIPSVI-SLTQFSTISN------P-LR-TQAGNTD--N--GQFPLNVTTSGN----------Q :  89
EgrFLA2b   : GGFTVLIKLLRGT-----HVSDQVDSELSD---L-NDGITFFAPDDAAFSAL-KAGT-----------------------------------LNTLSNEQQVQLLQFHMVPTYL-TMSQFQTVSN------P-LM-TRAGGVG--G--KEFPLNVTTKGD----------K : 103
EgrFLA17.2 : ---------------------------------------TVFAPADFGFVAS-----------------------------------------NSAILERMVRLHILPHK-YPHEELARLP---D-RAS-LR-TLVPGRDL---------EIGRKDA--SC--STDTN :  68
EgrFLA20.2 : -------------------------------------------------------------------------------------------------VNNVRFHIVPNRYLAMADLERLP---A-GTV-MP-SLEAGQYL---------AVTTAGG--P-------- :  45
OsaFLA17.2 : ----------------------------------------VLAPNDEAMARLT--ADQ---------------------------------LSEPGSPENILYYHMIPEYQ-TEESMYNAVR------R-FG-KVRYDTLRL------PHKVVAREA--D------GS :  70
OsaFLA24.2 : ---------------------------------------TVLAPNDEAMARLT--TDQ---------------------------------LSEPGSPENILYYHMIPEYQ-TEESMYNAVR------R-FG-KVRYDTLRL------PHKVVAREA--D------GS :  71
OsaFLA16.2 : ---------------------------------------TVLAPNDEAMARLT--TDQ---------------------------------LSEPGSPENILYYHMIPEYQ-TEESMYNAVR------R-FG-KVRYDTLRL------PNKVTAREA--D------GS :  71
OsaFLA4    : ---------------------------------------TAFCPTDDAMRAFM--PTYK--------------------------------NLTADGKASLLLFHAVPVYY-SLRSLKSNN------GV-MN-TLATDGSA-K---N--FNFTVQNE---G-----DK :  72
OsaFLA23   : ----------------------------------------------------------------------------------------------LTADAKVALLLYHAVAAHF-SEEALKAIN------GE-VN-TLATDGGGGG--KV--LNLTIEED-DDG-----AG :  57
OsaFLA12.2 : -GFNVAASMLEAS-----GVADEFT---ADE---RGAGITVFVPTDDAFADL-PAT-----------------------DR-------LQSLPADRKATVLRFHVLHSYY-PLGSLESIVN------P-VQPTLATERF-EA--GQFTLNITRVN----G-----S- : 103
OsaFLA1    : AGCKQFARLIVS-S----GVMKMYQA---A----MDKALTLFAPNDDAFQAKG-LPDLS---------------------------------KLTSAELVTLLQYHALPQYA-PKASLKTIK------GH-IQ-TLASTGAG-----K--YDLSVVTK---G-----DD :  99
OsaFLA5    : GPFHTFLDLLEKT-----DVLRTFQSQANGS----KDGITVFVPKDAAFASL-ARSA-----------------------------------TANLTSDQLKSLALYHALPRYY-SLAEFNRLGG------A-ASPVP---TLAGG---EYTVNVTDD-----M-----GT : 102
OsaFLA8    : GQFNTLIRLMRST-----GAAEQIDNQLNS---S-RNGLTVFAPTDNAFTSL-PSGT-----------------------------------LNSLSDQQKNSLVQYHVLSTLI-PMSQFDTVSN------P-LR-TQAGSNS--P--GQYPLNVTAEGQ----------Q : 103
OsaFLA27   : GPFHTFLNYLEKT-----NVIETFQSQANKT----KEGVTIFVPKDSAFAAI-KQST-----------------------------------FSNLTGDQLKTLLLYHAFPKFY-SLAEFKNLS-------E-LNPVN---TFAGA---PYTLNLTDD-----M-----GT : 101
OsaFLA26   : --FNVAASMLEAS-----GVADEFE---ADE---RGAGITVFAPTDDAFAGL-PAG-----------------------DR-------LQSLPAERKAVVLRFHVLHSYY-PLGSLESIVN------P-VQPTLATEFT-QA--GRFTLNITRAN----G-----S- : 102
OsaFLA7    : GQYTTFIRLMKET-----QQDTQLNSQLNNSF-N-GNGYTVFAPTDNAFNNL-KPGT-----------------------------------LNSLTQQQQVALVQGHVLPQFY-SMDSFQTASN------P-VR-TQASGTD-----GPYTLNITSTTN---------NN : 105
OsaFLA14   : GPFSTFLMYLRQT-----NLVAVFEHQAYRT----HQGITIFVPVDMAFAAI-EPSV-----------------------------------LAGLSRNQLKHLLMYHSLAKHY-TLAEFDGLS-------Q-SNPVK---TLAGG---RYAVNVTYD-----G-----GV : 101
OsaFLA2    : AGCKRFASLITS-T----GVLKTFED---A----MDKGLTLFAPNDDAFDAKG-APDVK---------------------------------KMPSADLVTLLKYHALPSYN-PKPTLKTVS------RA-MR-TLASTASG-----K--YNITVDTR---G-----DA :  99
```

```
OsaFLA11.2 : --CKSFAGLLAANA----DVFRAVNE---T----KDNGLTLFCPVDAAVAAFM--PSYK---------------------------------NLTAKAKTAILLYHAVPDYF-SLQLLKSNN------GM-VT-TLATASESKK---D--YSYDVQNK---G-----ET :  99
OsaFLA9    : GQFTKFLQLLQST-----QAGEQINNQIKGKA-SSSGGLTVFAPPDNAFAAL-PTGT---------------------------------LNKLSDQQKTSLVQFHVVSALL-PMAQFDTVSN------P-LR-TQAGETA--A--GKYPLNVTAEGS----------R : 106
OsaFLA3    : --CKNFAGLLASNA----DVYSNINA---T----KDNGLTLFCPVDAAVDAFL--PKYK---------------------------------NLTAKGKAAILLYHAVPDYY-SLQLLKSNS------GK-VS-TLATASVAKK---D--YSYDVSND---R-----DS :  99
OsaFLA18   : GSYTTFIRLMKST-----QQDTQLNSQLNGT----STGFTVFAPTDGAFSSL-KPGT---------------------------------LNSLSAQDQVSLVQAHIVPKFY-SMDAFDTASN------P-VR-TQASGGD-----GPYTLNITATST---------NQ : 103
OsaFLA13   : GPYGTFLGYLTKT-----GVITTFQSQANDTA-AGAPGVTVFAPEDSAFAAV-GGGAA--------------------------------LSNLTADQLRTLMLCHGVPRYH-PLSSFSALA-------A-SGPAP---TFAGG--QQYAVNVTDA-----A-----GT : 106
OsaFLA15   : GHYNTFVRLLKDT-----EVTSQVSSLLNNDR--NGDGLTVLAPTDAAFGRL-RPGT---------------------------------LNQMDAQAQAELVLYHVLPRYY-GFVTFETTTN------P-VR-TQASGQR-----GVCTVNVTTAGE---------DR : 105
OsaFLA19   : GQYNTLLRLLNAT-----RVGEQLGSQLKT----TYDGLTFFAPTDAAFAAL-RPGT---------------------------------LNGLSDQEQVQLVLYHVLPRYY-TLATFQTASN---------------------------------------------- :  79
OsaFLA10   : GGCKAFADLIAASP----DASSTYQS---A----AGGGITVFCPTDDAVRAFL--PRYK---------------------------------NLTADGKAELLLFHAVPVYY-SRGSLKSNN------GV-MN-TLATDGAA-K---N--YNFTVQNE---G-----DA : 100
OsaFLA6    : GKYTTFLRLLHES-----RVDTQINSQLMD----SYNGLTMFAPTDAAFAAL-KPGT---------------------------------LNSLSSQDQIQLMLYCVLPRFY-SLAMLTTLGG------P-VN-TQASGAD-----GPYKYKIKPSN----------NN : 102
OsaFLA25   : PEFGLFSYLISKT-----HVDRDINSR---------NTVTVLVPDNSAVDWLL--RRS--------------------------------A-RLPRAALVELLSVHVVLDYF-DAAKI--------------------------------------------------- :  68
OsaFLA20   : SDFSTFNHLLTQT-----KLADEINRR---------QTITVLALDNGAAGGVS---------------------------------------SLPSDEQRKVLSVHVVLDYY-DTEKLGGMKM---------------------------------------------- :  69
PabFLA22.2 : ---------------------------------------TVLAPNDEAMGKLT--TDQ----------------------------------LSEPGAPEHIVYYHIIPDYQ-TEESLYNAVR------R-FG-KIKYDTLRV------PHKLLSKEA--D------GT :  71
PabFLA9    : ---------------------------------------TVLAPNDEAMGELT--TDQ----------------------------------LSEPGAPEQIVYYHVIPEYQ-TEESLYTAVR------R-FG-KVKYDTLRI------PHKMVTQEA--D------GT :  71
PabFLA17.2 : GGYNEMADILVNLT----SLASELAKLVSE-----GYKVTVLAPNDEAMGELT--TEQ----------------------------------LSEPGAPEHIMYYHIIPEYQ-TEESLYN------------------------------------------------- :  74
PabFLA15.2 : -NFNFVVSMMVAS-----GITSDLE---SDQ---AGAGITIFAPTDDAFSAL-PPDTLQ---------------GL------------------TAENKAVVLKYHVLHSYY-PLGSLDSIVN------P-LQPTLATESM-GA--GTYTLNITRVN----G-----S- : 102
PabFLA5    : KNFNFVVSMMVAS-----GMTSELE---FDQ---AGAGITIFAPTDDSFSAL-PPHTLQ---------------GLTARNKA--------LQGLTARNKAVVLKYHVLHSYY-PLGSLESIVN------P-VQPTLATESM-GA--ETYTLNITRVN----G-----S- : 113
PabFLA20.2 : -NFNFLVSLMEAS-----GVVSEFE---SEE---AGAGITIFAPSDDAFSAL-NPETLQ---------------GL------------------TAEKKAVILKYHVLLSYY-PLGSLQTIVN------P-AQPTLATQAM-GA--GSYTLNITRMD----G-----M- : 102
PabFLA21.2 : GGCKIFAGMISA-T----GVVKTFKD---A----VQGGLTVFAPTDASFTGVT-SKLLS---------------------------------KLSSEDKVSLLEFHALPMYS-PLGYLKTSN------GP-IS-TMATTGAG-----K--YVMTASSS---G-----DT :  99
PabFLA16.2 : AGCKIFVQMITA-T----GVLQTYSD---A----VASGLTLLAPTDGAFSGTV-MLKLK---------------------------------KLSSAQEVSLLEYHAVPAYN-PVGTLKTTI------AP-IS-TLATNGAS-----K--YALSVSSA---G-----DT :  99
PabFLA10   : GPFKTFLRLLEAS-----DLLTVLQAQANN----TEQGITIFAPSDTAFSSL-PKKL---------------------------------LANLTADEIKEVLLAHCISKFY-TLTDFQDFSN------P-AN-TMATGSN--G--GKYNLNITGDGG----------T : 103
PabFLA6    : GGCKIFAGLISTT-----GVIKSYDDSIKSSQDAVKKGLTVFAPTDGAFTDV-IKKL---------------------------------LKKLSSEDKVSLLKFHAVPSYT-PLGTLKNTNG------P-VT-TMASN---------YAVTVSSSGN----------T : 102
PabFLA11   : --------LITA-T----GVLHTFQE---I----VDSGLTIFAPVDGAFDSVM-T-EIK---------------------------------KLSRAQQVSILEYHARPVYS-PLGTLKTTD------GDVVT-TMATNGAE-----R--YSLTVGVN---G-----DN :  91
PabFLA18.2 : -----------------------------------------------------------------------------------------------------VRYHVVPNRRLLLADLVRLP---A-GTR-LH-TLLHGQSL---------LVTDN-------------- :  39
PabFLA19.2 : ---------------------------------------------------D------------------------------------------GQDFVDNVTYHVVPNRRLLLADLMHLP---A-GTV-LH-TLLDGQSL---------VVTNS------------- :  47
PabFLA12   : GQFKTFLSLIAGT-----QAETQLQTQANN----TQQGLTLFAPLDGAFSSL-RPQY---------------------------------KAMLSKLTDQ----------------APTLSN------P-LS-TM----------GSYKFNVSAFG----------AQ :  81
PabFLA13   : --------------------------------------------------------------------------------------------AKL--DKIALLQYHALPSYY-TFGQFQTVSN------P-VR-TMASGNG-----GPFGVNVTAVGN----------S :  51
PabFLA8    : ----------------------------------------------------V-KNELK---------------------------------KLSSEDKESLLEFHALPIYS-PRTTLKFSK------RP-IA-TMASTGPG-----K--YDIKVSSA---G-----DT :  59
PabFLA14   : GPFKTLLNLLEAS-----DLLTSIQSQANN---------TEQE-----RSVV-GP-L---------------------------------HSQVLPDEKKEVLLAHCIPKFY-TLTDFQNFSN------P-AN-TMATGSN--G--GKYNLNITAMSG----------A :  92
PabFLA23   : ---------------------------------------TIFAPPDLALLPVV--G--------------------------------------SPALPHHVLCHVAAQR-LSYGDLVNKT---S-SDR-IP-SLALGHPI---------VVTTGPL--T----NQGT :  67
PpaFLA10.2 : ---------------------------------------TILAPNDQAMQLLT--TEQ----------------------------------LNQA--MEPLLYYHFLSEYQ-TEESMYNAVK------R-LG-KQSYSTLRH------PHKVVASES--D------GT :  69
PpaFLA11.2 : ---------------------------------------TILAPNDQAMQQLT--IEQ----------------------------------LDMP--MEPLLYYHFLSEYQ-TDESMYNAVK------R-LG-KQSYSTLRH------PHKVIASES--D------GT :  69
PpaFLA1    : PSLSILADVIEAS-----GLAEQLSM---------TDNVTVLAPDNNAFNGTG--GLLSILAAN----------------NLTLEQ----VTAPGSNRAASILLYHIVPSPA-TAADLTDGQT--------LT-TALG---KA-------YELTV-DK--TATPTV-VV : 109
PpaFLA2    : PRLQQLAAVIEAA-----GLADTVER---------LVNVTVIAPDNNAFNGTN--GLLSLLRQN----------------NLNLTD----VTAPGQNRAASILLYHIIAGPA-RAADLKNNQV--------LT-TFLG---PN-------YTLRV-NK--QTAPTL-IV : 109
PpaFLA3    : PEYSTFKSLMEST-----AVLSEVQTR---------SSLTILCPPNSVLDPFI--AAR--------------------------R-------SFSTQMLADVIRYHVLLQYL-DSAELRTQTNN---GGM-FT-TLYQTTGRANELDGF-VNITVTPS---------NA : 104
PpaFLA5    : PQFSMQKDALISS-----GVADALKGM---------NTLTLLLINNGAFGGYL--GGH--------------------------S-------TYTPQMVSDVLKYHILLSYF-DTETIKTVSTMN--DGV-VT-TLYQSTGRANGMDGF-VNITVSPT---------DG : 105
PtFLA1.2   : ---------------------------------------TVLAPNDEAMAKLT--TDQ----------------------------------LSEPGAPEQIIYYHVIPEYQ-TEESMYNAVR------R-FG-KISYDTLRL------PHKVLAEEA--D------GS :  71
PtFLA19.2  : ---------------------------------------TVLAPNDEAMAKLT--TDQ----------------------------------LSEPGAPEQIIYYHVIPEYQ-TEESMYNAVR------R-FG-KISYDTLRL------PHKVLAQEA--D------GS :  71
PtFLA6.2   : ---------------------------------------TVLAPNDEAMAKLT--TDQ----------------------------------LSEPGAPEQIIYYHIIPEYQ-TEESMYNAVR------R-FG-KIGYDTLRL------PHKVAAQEA--D------GS :  71
PtFLA8.2   : ---------------------------------------TVLAPNDEAMAKLT--TDQ----------------------------------LSEPGAPEQIIYYHIIPEYQ-TEESMYNAVR------R-FG-KIGYDTLRL------PHKVVAQEA--D------GS :  71
PtFLA5.2   : ---------------------------------------TVFCPLDDPFKAFF--PKFK---------------------------------NLTASGKVSFLEFFGVPIYQ-SLAMLKSNN------GI-MN-TLATDGEK-----K--FDFTVQND---G-----ED :  71
PtFLA22.2  : ---------------------------------------TVFCPLDDPFKAFL--PKFK---------------------------------NLTASGKESLLQFFGVPVYQ-SLAMLKSNN------GI-MN-TLATNGDK-----K--FDFTVQND---G-----ED :  71
PtFLA42.2  : ---------------------------------------TIFAPVDQAMDAY----AK------------------------------------NVSDYSSIFRKHVVPGL-FPRQDLEGFN---D-GTS-LP-TFSGGFMI---------NLTKSG------------ :  62
PtFLA37    : ---------------------------------------TLFCPPDSLLFSVD--LAS------------------------------------TAPHYTKSLFLHVSPSR-LSTSDLRNLTAASG-GTY-ID-SLVPNHRL---------LITNSLA--Q----LNGT :  73
PtFLA17    : AGCKTFASLLQTS-----GVIKTYQ-------SAADKGLTIFAPNDEAFKAA-GVPD---------------------------------LSKLTNAEIVSLLQYHATATYS-PFGSLKTSKD------P-IS-TLASNGA-----GKFDLTVTSAGD----------S :  99
PtFLA38.2  : HSFLVAASLLSAS-----GVVQEFE---ADE---GGAGITLFVPTDSAFSDL-SATA------------------------IS-------LQSLPADKKADVLKFHVLHSYY-PLGSLESIVN------P-VQPTLATEDM-GA--GSFTLNISRVN----G-----S- : 105
PtFLA30.2  : --FFVAASLLSAS-----GVVEEFE---ADE---GGAGITLFVPTDSAFSDL-SETD------------------------VS-------LQSLPADKKADVLKFHVLHSYY-PLGSLESIVN------P-VQPTLATEDM-GA--GSFTLNISRAN----G-----S- : 103
PtFLA20    : GHFKTFIRLLKST-----QLDSNLNSQLGN----TNNGLTIFAPSDSAFSAL-KTGT---------------------------------LRTLTDQEKVELMQFHIVPMFI-SSSQFDTVSS------P-LK-THA-GSG-----ARFQLNVTASGN----------S : 101
PtFLA23    : GQFSVFIRLLKAT-----QEDVTLNGQLNN----TNNAITIFAPSDNAFSSL-KSGT---------------------------------LNSLNDQEKAELVQFHIIPQYL-SSSQFQTVSN------P-LT-TQA-GSG-----GRLELNVTTTGN----------S : 101
PtFLA18    : GPFHNFLNYLEST-----KVIDTFQNQANNT----DEGITIFVPKDDAFKNL-KKAS---------------------------------LSNLTQDQLKQLILFHALPHYY-SLSDFKNLS-------Q-VSPVS---TFAGA--GGYALNFTDT-----S-----GT : 102
PtFLA10    : GQFSVFIRLLKAT-----QEDVTLNGQLNN----TNNAITIFAPSDNAFSSL-KSGT---------------------------------LNSLSDQEKAELVQFHIIPQFL-SSSQFQTVSN------P-LT-TQA-GSG-----GRLELNVTTTGN----------S : 101
PtFLA16.2  : -GCKAFSDLLIAS-----GAHTTFEE---N----VDGGLTVFCPTDPVINGFM--PKYK---------------------------------NLTAPQKVSLLLYHGIPIYQ-SLQMLKTSN------GI-MN-TLATNGAN-----K--YDFTVQND---G-----EV :  97
PtFLA34    : GHFTVFVRLMQAT-----TEDTELNKELNK----TNNGITIFAPSDSAFSNL-KAGF---------------------------------LNALSDEDKTELVKFHVLPALI-SSSQFQTVSN------P-VR-TQA-GTG-----PRVTLNVTTTGN----------F : 101
PtFLA9     : GQFTTLIRLLKST-----QEADQINTQLNN---S-NQGLTVFAPTDNSFANL-KAGT---------------------------------LNSLSDQQKVQLVQFHILPNFL-SMSNFQTVSN------P-LR-TQAGNSA--D--GEFPLNVTTSGN----------Q : 103
PtFLA12    : GPFHTFLSYLEST-----KVVDTFQNQANNT----DEGITIFVPKDDAFKNL-KKPS---------------------------------LSNLTQDQVKQLILFHALPHYY-ALADFKNLS-------Q-VSPVS---TFAGA--GGYALNFTDV-----S-----GT : 102
PtFLA7     : GQFTTLIRLMKST-----QEADQINTQLNN---S-NQGLTVFAPPDNAFTNL-KAGT---------------------------------LNSLSDQQKVQLVQFHIIPNFF-SMSSFQTVSN------P-LR-TQAGNSA--D--GEFPLNVTTSGN----------Q : 103
PtFLA2     : GHFTVFARLMQAT-----TEDTELNKELNT----TNNGITILAPTDNAFSSL-KAGF---------------------------------LNSLSDEDKTELVKFHVLPAFI-STSQFQTVSN------P-VR-TQA-GTG-----PRVTLNVTTTGN----------F : 101
PtFLA13    : GHFTIFIRLLRST-----QEENHLFSALND----SSTGLTIFAPTDSAFSEL-KSGT---------------------------------LNTLSDGDKSELVKFHVVPTFL-STSQFQTVSN------P-LG-TWA-GTG-----SRLPLNVTSYPN----------S : 101
PtFLA47    : GHFTIFIRLLRST-----QEENHLFSALND----SSSGVTIFAPTDSAFSEL-KSGT---------------------------------LNTLSDGDKSELVKFHVVPTFL-STSQFQTVSN------P-LG-TWA-GTG-----SRLPLNVTSYPN----------S : 101
PtFLA40    : GHFTIFIRLLRST-----QEENHLFSALND----SSSGVTIFAPTDSAFSEL-KSGT---------------------------------LNTLSDGDKSELVKFHVVPTFL-STSQFQTVSN------P-LG-TWA-GTG-----SRLPLNVTSYPN----------S : 101
PtFLA28    : GHFTIFIRLLRST-----QEENHLFSALND----SSSGVTIFAPTDSAFSEL-KSGT---------------------------------LNTLSDGDKSELVKFHVVPTFL-STSQFQTVSN------P-LG-TWA-GTG-----NRLPLNVTSYPN----------S : 101
PtFLA39    : GHFTIFIRLLRST-----QEENHLFSALND----SNTGITIFAPTDSAFSEL-KSGT---------------------------------LNTLSDGDKSELVKFHVVPTFL-STSQFQTVSN------P-LG-TWA-GTG-----SRLPLNVTSYPN----------S : 101
PtFLA50    : GHFTIFIRLLRST-----QEENHLFSALND----SSTGLTIFAPTDSAFSEL-KSGT---------------------------------LNTLSDGDKSELVKFHVVPTFL-STSQFQTVSN------P-LG-TWA-GTG-----SRLPLNVTSYPN----------S : 101
PtFLA29    : GHFTIFIRLLRST-----QEENHLFSALND----SSTGLTIFAPTDSAFSEL-KSGT---------------------------------LNTLSDGDKSELVKFHVVPTFL-STSQFQTVSN------P-LG-TWA-GTG-----SRLPLNVTSYPN----------S : 101
PtFLA32/49 : GHFTIFIRLLRST-----QEESHLFSALND----SSTGLTIFAPTDSAFSEL-KSGT---------------------------------LNTLRDGDKSELVKFHVVPTFL-STSQFQTVSN------P-LG-TWA-GTG-----SRLPLNVTSYPN----------S : 101
PtFLA45    : GHFTIFIRLLRST-----QEENHLFSALND----SSTGLTIFAPTDSAFSEL-KSGT---------------------------------LNTLSDGDKSELVKFHVIPTFL-STSQFQTVSN------P-LG-TWA-GTG-----SRLPLNVTSYPN----------S : 101
PtFLA35    : GHFTIFIRLLRSI-----QEENHLFSALND----SSTGLTIFAPTDSAFSEL-KSGT---------------------------------LNTLSDGDKSELVKFHVIPTFL-STSQFQTVSN------P-LG-TWA-GTG-----SRLPLNVTSYPN----------S : 101
PtFLA46    : GRFLSFVRLMKAT-----HVDTQLFSQLNS----STDGITMFAPNDNAFSSL-VAGA---------------------------------VGSLNDREKLEFVQFHILPRFL-SISDFQTLSN------P-VK-TLA-GSD-----RKFPLTITTSDN----------S : 101
PtFLA3     : GQFATLIRLLNNT-----QTLNQIENQLNS----SSEGMTIFAPTDNAFNNL-KAGA---------------------------------LNGLNQQEQVQLLQYHTLPKFY-TMSNLLLVSN------P-VP-TQASGQD-----GVWGLNFTGQ-----S-----NQ : 102
PtFLA15    : GQFVTFISLLNKT-----QTFNQIENQINS----SSEGMTIFAPTDNAFSNL-KSGA---------------------------------LNGLSQQQQVQLLQYHMLPKFY-SLSNLLLVSN------P-VP-TQASGQE-----GVWGLNFTGQ-----S-----NQ : 102
PtFLA14    : ---------------------NHLFSALND----SSTGLTIFAPTDSAFSEL-KSGT---------------------------------LNTLSDGDKSELVKFHVIPTFL-STSQLQTVSN------P-LG-TWA-RTG-----SRLPLNVTSYPN----------S :  85
PtFLA48    : GHFAFFTRLIKST-----QEDIQLFSQLND----SRDGVTVFAPTDGAFSAIIKSGV---------------------------------LNSLTDHQKIELVQFHIIPRIL-TTANFQTVSN------P-IT-TLA-GSG-----NRFALNVITTEN----------M : 102
```

```
PtFLA27     : GGFAVFIRLIKST-----QEDIQVFSQLND----SRDGVTIFAPTDGAFSAIIKSGV--------------------------------LNSLSDHQKIELVQFHIIPRIL-TTANFQTVSN------P-IT-TLA-GSG-----SRFALNVITTEN---------M : 102
PtFLA43     : GHFTIFIRLLRST-----QEENHLFSALND----SSPGLTIFAPTDSAFSEL-KSGT--------------------------------LNTLSDGDKSQLVKFHVVPTFL-STSQFQTVVG-------------------------YHLTSQSYTN---------S :  90
PtFLA33     : GHFTIFIRLLRST-----QDENRLFSALND----SSTGLTIFAPTDSAFSEL-KSGT--------------------------------LNTLSDGDKSELVKFHVVPNSY-LLPSSR---------------------------------------------------- :  75
PtFLA21     : PDFSTFSSYLTQT-----QLAGEINSR---------QTITVLVVENGNMSPLS----------------------------------GKPNGEIKNVLSGHVILDYY-DVAKLQKLQNK---TAM-LT-TL---------------------------------- :  77
PtFLA26     : PEFANFNDLLSQS-----GLAQEMNSR---------QTITVLVLDNGSIDGLS----------------------------------GRPLDIAKRTLSAHVILDYY-DQIKLSKLQKA---STI--------------------------------------- :  73
PtFLA4.2    : ----------------------------------------TIFAPPELEFVAS-------------------------------------SSPMLEKLVRLHILPQR-ATYIELAALP---D-KQR-LR-TLLPDEDL---------KITKG-----------VG :  61
PtFLA44.2   : ------------------------------------------------------------------------------------------------VRFHIVPNHYLSTADLERLP---V-GAT-LP-TLERGQAL---------VVTSAGG--L----TGFN :  46
SmoFLA3     : GGYNEMAELLVNLT----SFAWDMAKLVNE-----GHRLTILAPNDHAMDHLT--TEQ------------------------------LNAPGGLEATIMYHVITEYQ-TEESLYNAVR------R-FE-KVKFLTLWQ------PHTIHAKET--D------GT : 101
SmoFLA6.2   : GNFNTFLGLLAST-----GVDKALR---------SMSAVTILAPDDDAFKAL-PPNA-------------------------------LAELSQAQKIALLQFHAIATYF-TMGSLRSVAT------P-LP-TVASSRI-----GGFELNVSTAGG---------KG :  98
SmoFLA7.2   : GKFTTFLNLMQS-T----GFTAALA---------TLPAFSIFVPTDEAFQGLP-NGTMA-----------------------------ALSPSQASSLMAYHTLPAYT-SSGSLQRQN------SL-VQ-TVASNGDNQ----K--FLIQVAPSGGNS-----GG : 101
SmoFLA8.2   : --YKTFQKLMQDT-----GTLAAAEDIEHQP---FTSGVTVFAPTDSAFQNL-PSGSL------------------------------AALTQSQRQLLVRYHLIPSFF-TFGSLRTLKA------P-LT-TLATS----N--RNFEVNASGEGP---S-----GG : 102
SmoFLA9.2   : -----------------------LVNE--------GHALTVLAPGDRAVARLA--AE-------------------------------HLGAIESLLAYHIVAEYQ-TEESLYTL----------------------------------------------------- :  50
SmoFLA4     : PNFKRFNTLLTTS-----GVVDQVNPM---------LTATIFIPENTLLNDF--IYDM-------------------------G-----K-HPSEEKLADLVRYHISDFYI-ESEFLAVRRNF---SSP-IK-TLFRERPYEETLHFRWLQLNVDNH---------GV : 107
SmoFLA5     : PDYTNLNMLLLQT-----GVANEINQR---------SSLTILAAEDSVLDPIL--DQL-----------------------A-----Q-SVTGQQIADILRYHVLLEYE-GINDLRSLPNK---SKL-FT-TLFQTTGRASNNAGF-VNITDDPN---------GG : 105
BdiFLA23.2  : ----------------------------------------TVLAPNDEAMARLT--TDQ------------------------------LSEPGSPENILYHMIPEYQ-TEESMYNAVR------R-FG-KVRYDTLRL------PHKVVAREA--D------GS :  71
BdiFLA22.2  : ----------------------------------------TVLAPNDEAMARLT--TDQ------------------------------LSEPGSPENILYHMVPEYQ-TEESMYNAVR------R-FG-TVRYDTLRL------PQKVTAREA--D------GS :  71
BdiFLA9     : ----------------------------------------TIFCPVDAAVASFM--PKYK------------------------------NLTAKAKTAILYHAVPDYY-SLQLLTSNS------GK-VS-TLATSSVAKK---D--YSFDVSKD---G-----ET :  73
BdiFLA1     : ----------------------------------------TILAPTDAAFEDL-PSGT--------------------------------MNGLSSQDQIQMLYCVIPRFY-SLSMLGTLNG------K-VS-TQASGSD-----GPYEYKIKPSG----------SN :  72
BdiFLA20.2  : -GFNVAASMLEAS-----GVADDFE---ADE---RGAGITVFVPTDDAFAAG-GLPDA-------------------AGR-------LQSLPADRKAVVLRYHVLHSYY-PLGSLESIVN------P-VQPTLATECT-EA--GRFTLNITRSN----G-----S- : 106
BdiFLA10    : -GFNVAASMLEAS-----GVAEEFE---DDE---RGAGITVFVPTDDAFASL-PAG-P-------------------GDR-------LQSLPAERKAVVLRYHVLHSYY-PLGSLESIVN------P-LQPTLATEFSSQA--GRFTLNITRAN----G-----S- : 106
BdiFLA11    : GPFHTFLSYLQKT-----NVIETFQRQANKT----KEGITIFVPKDSAFAAL-KKST--------------------------------FSNLTSDQLKMLMYHALPEFY-SLAQFRNLS-------V-LNPVN--TFAGA---PYTLNLTDD-----M-----GT : 101
BdiFLA3     : AGCKQFARLIVS-S----GVIKTYQA---A----MDKGLTIFAPNDDAFQAKG-LPDLS-------------------------------LKTSANLVTLLEYHALPQYA-PKASLKTMK------GG-IP-TLASTGSG-----K--YDLSVVTK---G-----DD :  99
BdiFLA12    : GPFHTFLDYLEKT-----DVLKTFQSKANDTK-ESAEGITIFVPKDSAFSSL-RATT--------------------------------FANLTGEELKSLVLYHALPKYY-SLAEFNKLS-------S-LNPVP---TFAGS---QYTLNLTDN-----M-----GS : 104
BdiFLA8     : GQYTTFIRLMKET-----QQDTQLNSQLNNSF-N-GNGYTVFAPTDNAFNNL-KPGT--------------------------------LNSLTQQQQVALVQGHVLPQFY-SMESFQTASN------P-VR-TQASGQD-----GPFTLNITATAN---------NQ : 105
BdiFLA13    : GQYTKFMRLMKST-----QQDTQLNSQLNGS--DTGFTVFAPTDNAFDSL-KAGT--------------------------------LNSLSQQEQVSLVQAHIVPAFF-SMESFETASN------P-VR-TQASGAD-----GPYTVNVTATSN--------GQ : 103
BdiFLA4     : GRFQTFVMYLQQT-----GLVEVFEIQAHRT----HHGITILVPTDRAFAAI-EPSV--------------------------------LSGLKKHQVKSLMMYHALARHY-ALKEFDALS-------R-VSPVT---TFAGG---LYTVNVTYD-----A-----GA : 101
BdiFLA14    : GQFTKFLQLLQST-----QEDSQIDNQLKGKS--SSGGLTVFAPPDNAFSAL-KSGT--------------------------------LNALSDAQKTSLVQFHVVSQLI-PMAQFDTASN------P-LR-TQAGETR--P--GKYPLNVTADGQ----------Q : 105
BdiFLA15    : GQFNTFIRLLQNS-----GVAAQIDNQLNS--SQTGGLTVFAPTDNAFTSL-ASGT--------------------------------LNSLSDNQQKNSLVQFHVLSTAV-PMSQFDTVSN------P-LR-TQAGSSS--P--GEYPLNVTATGQ----------Q : 104
BdiFLA16    : GPYGTFLDYLTKT-----DVIKTFQSQANDTEEQGGHGITVFAPQDSAFAAV-DSAAL-------------------------------SNLTADRLRSLMLHHAAPKYY-PLSVFSALA--------ASS---TPVSM--FAYSVNVTDK-----A-----GK : 103
BdiFLA17    : GGCKAFAALLSASP----DASSTFQS---A----IDGGVTAFCPSDGAVKSFL--PRYK------------------------------NLTAAGKAALLSHAVPVYY-TRRALKSNN------GV-MN-TLATDGGA-G---N--FNLTVQNV---G-----DQ : 100
BdiFLA21.1  : PEFSVYSGLLRET-----GLASILDGR---------RVVTVLAPNNTDIPKVI--H---------------------------------TLPRPLLADLLALHVIPDYL-DPEKLDALRRG---RTG-DG-S----------------------------------- :  77
BdiFLA21.2  : PDLTAFNSLLTDS-----GLARAINAR---------PTVTVLATNNTALADSL--RGL----------------------------R-HLPEPALVDLLALHVVLDYL-DPEKLDALRRG---RTG-GG-SIVT------------------------------ :  83
BdiFLA5     : AGCKRFAALVAGNP----GVLKAYQA---A----MATGLTIFAPNDDAFVAKAGTPDVG-------------------------------KMEKDDLVRLLMYHAVPAYE-PKPSLKLVKA---GARP-LR-TLASTAAG-----E--YNVTVVAR---G-----DD : 104
BdiFLA18    : GGCKAFAALLAASP----DAASTYQS---A----ADGGVTVFCPSDAAVRAFM--PRYK------------------------------NLTAAGKAALLLFHAVPVYY-PSGSLKSNN------GV-MN-TLATDGAS-K---N--FNFTLQNE---G-----EA : 100
BdiFLA6     : ANFTDFSSALASA-----NLTAQIDGR---------TPITILAVDNAAVAQLK--A--------------------------------R-RLAPESLAHVLSLHVLLDYF-DDARIRHL----------------------------------------------- :  69
BdiFLA19    : PDFTLFNYLLTKT-----KVSAQINRR---------GTVTILAPVNADVDWLL--RRS-------------------------------S-RLPRAALVELLAVHVILDYI-DAAKL------------------------------------------------- :  68
BdiFLA7     : PEFKTFNSLLSKT-----KVAEEINAR---------QDAITVLVVDNKAGAIT------------------------------------ALPADTQKKVLAVHVILDYY----------------------------------------------------------- :  60
MpoFLA6.2   : GGYSIFVSLLTQ-T----GVDVVFGGR--Q----SGEGITIFVPTDEAFNKLP-GQWFE----------------------------AFELKEQKLLLEYHALTRYN-SLDALWRYR------DKQVP-TVSSTVQEGP--DA--FNLLVTAN---K-----GL : 104
MpoFLA1     : GQLSSLKMVVDAA-----GLADTFDDS---------ALNVTVFAPNNAAIQGLV--NVLNAS------------------GLTLAN----VTGN-NNKAASTVLYHVATLVA-TSTQLVNDQS--------LP-TLYG--GYN--------LTVDR-N-----A--TN-VE : 103
MpoFLA14.2  : GGYSEFAGLLVDLT----SLGSEISKLVNM-----GYKLTILAPDDDAMKWSGAL--TEEH------------------------------LSSQTALEDILHYHIITEYQ-TEESLYSTLR------R-MG-KTHFSTLRV------PHKLAAHEV--D------GQ : 102
MpoFLA7.1   : PRFSVLSDLLVSS-----GVEKEINSR---------TSITILAPAAVLTAFQ--ASV----------------------P-------NADTVKIADLLRYHVLLQYF-DMTELKGLGTVN--YSS-VT-TLLQTTGRANEQDGF-VNIYNTAT---------QI : 105
MpoFLA7.2   : --FSLFISLLKAT-----GVDGVLA---TRQ---TSGGLTVFAPRESAFNAL-KSGSI-------------------------------QTLSPGKQKLLMQYHALTQYY-TMNTFLSMVD------A-PVQTVSSTV---G--GGYLMNITSK-----A-----ST :  99
MpoFLA2     : PDLSLLGQVVQAA-----GLTETLSDP---------TLEVTVFAPNNAAFEELL-----QVLNSS---------------GLTLDD----VTAPESNKAASILLYHVVPVAA-LSTQLSDMQV--------LP-TLL---GKN--------LTVSL-M-----G--GM-VN : 103
MpoFLA3     : PDLSLLGQVVQAA-----GLTETLSDP---------TLEVTVFAPNNAAFEELL-----QVLNSS---------------GLTLDD----VTAPESNKAASILLYHVVPVAA-LSTQLSDMQV--------LP-TLL---GKN--------LTVSL-M-----G--GM-VN : 103
MpoFLA8.2   : -EFTLLISYLQET-----KVDAVFA---EKQ----AEGITIFAPRDSAFNSL-ATGSI--------------------------------QTLSLSEQKLLEYHALDGYE-SLDSLETMLN------K-PTKTLASAD---A--AGYILNVSATPG----Q-----ST : 101
MpoFLA9/10  : KDYTMFISLLKES-----GVDSIFA---GRQ---TGGGITVFAPTDSAFNGL-TSASL-------------------------------QALSVTDLKLLMQYHALTSYQ-PLDILQNMVD------S-PVSTIASTI---A--SGYLLNVSSA-----V-----MT : 101
MpoFLA11.2  : --YTMFISLLKES-----AVDSIFA---GRQ---TGGGITVFAPTDSAFNSL-ASGSI-------------------------------QGLSVSDLKLLMQYHALTSYQ-PLSVLQNMVN------S-PVSTIASTI---A--SGYLLNVSSA-----A-----KT :  99
MpoFLA12.2  : --YTMFISLLKES-----AVDSIFA---GRQ---TGGGITVFAPTDSAFNSL-ASGSI-------------------------------QGLSVSDLKLLMQYHALTSYQ-PLSVLQNMVN------S-PVSTIASTI---A--SGYLLNVSSA-----A-----KT :  99
MpoFLA13.2  : -NYNTFIQLIQST-----GIDAEFA---AKQ---TGTGITIFAPTDAAFAAL-PAGAL-------------------------------AALTPDQAKQVLRAHAIVTYY-PLGTLSTM----------------------------------------------- :  74
CcrFLA1.1   : DQFSVLALAALAVP----DLTEALGDP---------NRRLTVFAPTNAAFAALT--NKLSPGAGVDAADPEGILFALMGALPGP-L----PGRSPGEVLTTILSYHLIAFAA-PFQELENDGT--A------E-TVQGTLLR---------FRDGRVV---D-------- : 120
CcrFLA1.2   : DSFGILARAVGTVD----SLVKALDDP---------NSRFTVFAPTDEAFTALA--NQLVPGADLNPADVVEALVSAIAPLAD----VEAANSTIESILGYHALPFAA-PFQELEDMSS--A------E-TVQGDALR---------FADGLVI---D-------- : 121
CcrFLA1.3   : ------------------LVEVLGNP---------GSRLTVFAPTDEAFTNLA--NTLVPDANLEASDKDAVVEALVTAIAPLAD----VEAAGGATIESILLYHALGMAA-PLGKLEEMKT--A------K-TLQGGVLS---------FNSGSVT---D-------- : 106
CcrFLA1.4   : --------------------VEVLGNP---------GSRLTVFAPTDEAFTNLA--NTLAPDANLEASDKDAVVEALVTAIAPLAD----VEAAGGATIESILLYHTLGEAS-PIQELEKSDT--L------T-TLQGDSIS---------VADGKVT---D-------- : 105
CbrFLA1     : PRFSIFVGLLDKG------FAANLPL----------QPLTISVPENAAFSTLA--PGQL------------------------D-----TIKADEALLYEFIGYHGFADGFY-PKADLLAAIAK---STS-GT-VSVPT-------QGGSYVSFSSVG---------DV :  99
CbrFLA2     : -QFSMFYKALVDT-----TEIWNVEGRTVL----VGEQLILFAPTNAAFSTAL--SE-----EV-------------------ITCLQK----EP-GQA-TLASFLKI---------------------------------------------------------- :  66
CbrFLA3     : PEFQRFYEALNMS-----RSIDMVKGL--V----N-SGVTIFAPVNGAFDALG--AV-----Y-------------------TECMLK----RP-GMDDLLPLLVRFHVAT-GNFVNAQLMTMSS--------VQ-SFLG---LP--------IPLTH-AA---AG-----GL : 100
CbrFLA4     : TAYANFVEAALTTR----GATVSILLD---------DSNVTVFAPINSAFDKLG--SSL----------------------VDCLLSDS------SSGLLDIILNHIFEGG-HPSKSLTD-G------QK-LL-TVANTTAE---------IAV--N-----E------TG :  97
CbrFLA7     : GDLSMFSNALMAS-----RINLTLSGI--V----GGGRVTVFAPNNGAFAMLS--PE-------V-------------------KQCLDT----QS-GKIDVLTQMLLFHLAVGGNYTAAELKTISR--------LT-AASG---MP--------IDLMT-LG--DG-----TI : 102
CbrFLA9     : GDLSMFSNALMAS-----RINLTLIGI--I----GGGKVTVFAPNNGAFAMLS--PE-------L-------------------KQCLDT----QS-GKIDVLTQMLLFHLAVGGNYTVAELKTISR--------LT-AASG---MP--------IDLMT-LG--DG-----TI : 102
CbrFLA11    : -GLTTFVSLLKLA-----KVDESLKGI--A----ATGPVTVFAPSEAAFAVLS--SAYP---AQ-------------------LACVTT----GQ-GIDNYLSQILKYHILPNGNFTASKLTKLTQ--------VS-TLIG---LP--------IQLEYTLP--SG-----PL : 105
CbrFLA15    : KNLTLYVGALRSSRGALMAMEAQRRR----------PGGVTVFAPTDAAVSDQI--QRL------------------------GGCLAA----IPDVADLVMTSSLLYSAVPGHL-PMAEIRSRLNQSNGSLR-LP-TLYP--PNE--------VAVSS-AT--SRYPSTKMG : 117
CbrFLA16.1  : -QYSTLVSLIT--K----ANLTVSLDT---------TSPLTVFAPTNDVIEALN--VGD-------------------------TKFLTDPK------NVELLKRLVFYHFVNSK-ILAVNLTA-G------RS-LR-SLEGENLI---------VEV--E----D------GT :  94
CbrFLA16.2  : ----------------------------------------TIFAPSNQGMSSSV--E-M----------------------AQLLTNA---------TARNDILLYHLIAGR-YTFSWLKEHP------GV-YD-NFTGFEI---------VV-SGG---G------TE :  67
CbrFLA16.3  : ----------------------------------------TIFAPSNEAITNFM--LAY----------------------AALLSDN---------AKLDEVLKFHVLNGS-YSALFLRTNP------GD-YG-G----NF---------SVVQ----G------DS :  64
CbrFLA16.4  : ---SNYTQIAFLVR----RVGPTYLGE---------LTNNTIFAPSNEAITNFM--VAN----------------------AALLGDR---------AKLDEVLKFHVLNGS-YTASFLRKNP------GD-YG-G----NF---------SVDVQW---D------NS :  89
CbrFLA19    : PDLTMFVQALLVS-----DTFHVAISR--A----NGGGVTVLAPNNTAFSVFD--PA------V-------------------LDCLYS----KP-ASLNVLSQITKYHIIV-GNHNATELIAMQT--------VH-ADSG---LP--------LHFNL-TS--RG-----RV : 101
CbrFLA20.1  : -----------------------DYVNT--------LRNVTFLAPVNSAWDFLD--PAM----------------------KDCLMAGD-------KLVDVVVKSHTLIGK-LTAEMISNVP-----R--VE-NLYRFWL---------NVTTDD----I------TN :  78
CbrFLA22    : --LKKFYYALQIS-----RIELRLRDS--A----NRGPITVFAPTDSITKLD--PE------L--------------------WTCVTT----GQ-GPLDILSQITLYHFVTDGNFTAAEVATKRH--------LT-SASG---MP--------LGVKV-V---NG-----NV :  99
CbrFLA23    : ---KKFYYALQIS-----RVESRLRDS--V----NQGPITVFAPTDSFMKLD--PE------L--------------------WRCATT----GQ-GPLDVLSQIMLYHFVTSGNFTAAEVATKTQ--------LT-SASG---MP--------IDVKD-V---NG-----NV :  98
CbrFLA24    : --LKKFYYALQIS-----RVESRLRDS--V----N-QGPITVFAPTDSFMKLD--PE------L--------------------WRCATT----GQ-GPLDVLSQIMLYHFVTSGNFTAAEVATKTQ--------LT-SASG---MP--------IDVKD-V---NG-----NV :  98
                                                           p   a                                                         h
```

```
               *       180         *       200         *       220
AtrFLA10.2 : VKFGHG----EGSAYLIDPDIYM-DG--------------RISVQGIDKVLFP : 105
AtrFLA11.2 : VTLKTKA----VTAKITGTI-ID-ED--------------PLAVFSIDKVLQP : 104
AtrFLA5    : --GS----LRINYVPLKGPDAVY-NS--------------RIVVHSIF----- :  90
AtrFLA2    : VAVSSGI--VI--APVTQTV-FD-QK--------------PLAIFTVPKVLLP : 135
AtrFLA7    : VSLDSGW--SQ--TLISSSV-YS-TR--------------PVALYQVDSVLLP : 134
AtrFLA3    : VNVTTGL--VT--TPIANSI-NV-TS--------------PLAVYEVDKVLLP : 138
AtrFLA8    : VNVSTGI--ND--APVANTV-YT-DG--------------QLAVYQVDKVLLP : 135
AtrFLA9    : VTLDTGV----DKSRIASTV-LD-DA--------------PLCILTVDNVLLP : 131
AtrFLA4    : VAIDTGL--VQ--ASITRTV-FD-QN--------------PVAVFGISHVLLP : 128
AtrFLA6    : ----------------------------------------------------- :   -
AtrFLA12   : --DS----LRINYVPVKGPDAVL-NW--------------RIVVHSI------ :  89
FLA16.2    : VKFGHG----DGSAYLFDPDIYT-DG--------------RISVQGIDGVLFP : 105
FLA17.2    : VKFGDG----EKSAYLFDPDIYT-DG--------------RISVQGIDGVLFP : 105
FLA15.2    : VKFGHG----DGSAYLFDPDIYT-DG--------------RISVQGIDGVLFP : 105
FLA18.2    : VKFGSG----DRSAYLFDPDIYT-DG--------------RISVQGIDGVLFP : 105
FLA1.2     : VTLKTRI----NTVKIVDTL-ID-EQ--------------PLAIYATDKVLLP : 104
FLA20.2    : --DI----LLLNGVPLIYPDLYV-ND--------------WIAVHGFNQMI-- :  93
FLA21.2    : SGGD----FMISGVEIVDPDMFS-SS--------------NFVIHGISHTLEI :  81
FLA19      : SNDS----IFLDGVQLLIPGLFD-GQ--------------HIAVHGLADLLPL :  99
FLA4.2     : VTINSGV--VL--AVVTQTA-FD-QN--------------PVSVFGVSKVLLP : 138
FLA10      : VILHTGV----GPSRLADTV-VD-ET--------------PVVIFTVDNVLLP : 132
FLA8       : VILHTGV----APSRLADTV-LD-AT--------------PVVIFTVDNVLLP : 132
FLA11      : VNITTGV--VS--ATVANSV-YS-DK--------------QLAVYQVDQVLLP : 137
FLA12      : VNITSGV--TN--TTVSGNV-YS-DG--------------QLAVYQVDKVLLP : 136
FLA7       : VRIDSLW--TR--TKVSSSV-FS-TD--------------PVAVYQVNRVLLP : 134
FLA9       : INVSTGY--VE--TRISNSL-RQ-QR--------------PLAVYVVDMVLLP : 136
FLA6       : VNVSTGV--VE--TRINNAL-RQ-QF--------------PLAVYVVDSVLLP : 138
FLA13      : VNVSTGV--VE--TRLSTSL-RQ-ER--------------PLAVYVVDMVLLP : 137
FLA14      : ----------------------------------------------------- :   -
FLA3       : IYFG------------------------------------------------- : 103
FLA5       : VYFG------------------------------------------------- : 103
FLA2.2     : VTLETDV----VTAKVMGTL-KD-QE--------------PLIVYKIDKVLLP : 104
CreFLA11.1 : MILA-A----QSKAKVVTAD-LK-AG--------------MTIVHIVDTVLVP : 107
CreFLA11.4 : VFLT-A----TNTVKVIAAD-IA-AG--------------KSIVHKLDGVLV- : 129
CreFLA1    : AVAGNP----NSSAEVIKAD-IP-FN--------------AAIVHVINKVLLP : 131
CreFLA10.2 : AF----VVSTSTTANLVAQD-IP-AG--------------AGYVNVVDRVL-- : 145
CreFLA9.2  : ----------------------------------------------------- :   -
CreFLA8.1  : MTIT----GPQNSAKVVKANQLLANG--------------KVVAHLVDKVLLP : 131
EgrFLA9.2  : VKFGQG----EGSAYLFDPDIYT-DG--------------RISVQGIDGVLFP : 105
EgrFLA5.2  : VKFGQG----DGSAYLFDPDIYT-DG--------------RISVQGIDGVLFP : 105
EgrFLA10.2 : VTLKTKV----NTAKITGTL-LD-EQ--------------PLVIYTLDKVLLP : 104
EgrFLA11.2 : VTLKTRV----NKVKITGTL-LD-EQ--------------PLAIYSIDKVLMP : 104
EgrFLA16.2 : --DR----LMLNGVPMTSPDIYT-SD--------------WLSIHGVLDVI-- : 100
EgrFLA14.2 : --DG----LLLNGVPMTSPDIYT-SD--------------WLSVHGILDVI-- : 105
EgrFLA4    : VAIDTGI--VQ--ATVTQTV-FD-EN--------------PVAIFGVSRVLLP : 137
EgrFLA12.2 : VKLKTKV----VTATITGTV-ID-QD--------------PLIVYKIDKVLQP : 130
EgrFLA8    : VHVNSGW--TN--TKISSSV-LS-TD--------------PVAVYQVDHVLLP : 134
EgrFLA7    : VRLSSGW--TN--TKVSSSV-HS-TD--------------PVAVYQIDKVLLP : 134
EgrFLA13   : ----------------------------------------------------- :   -
EgrFLA6    : VNVSTGM--VE--TPINNAL-YQ-QS--------------PLAVYQVDKVLLP : 131
EgrFLA1b   : VNISTGV--TN--ATVDQTV-YN-DG--------------QLAVYMVDKVLLP : 136
EgrFLA3b   : VNVTTGV--VA--ASVDNAI-TS-NS--------------KLAIYQVDQVLLP : 122
EgrFLA2b   : V---------------------------------------------------- : 104
EgrFLA17.2 : QRTR----VAINGLEIGVPDIY------------------------------- :  86
EgrFLA20.2 : --AT----MRINYVRMKSPDVMR-NQ---------------KVVVH------- :  69
OsaFLA17.2 : VKFGHG----EGSAYLFDPDIFA-DG--------------RISVQGIDAVLFP : 104
OsaFLA24.2 : VKFGHG----EGSAYLFDPDIYT-DG--------------RIAVQGIDAVLFP : 105
OsaFLA16.2 : VKFGHG----EGSAYLFDPDIYT-DG--------------RISVQGIDAVLFP : 105
OsaFLA4    : VTIKTDA--SDGVARIKDTV-YD-KD--------------PIAIYAVDTVLEP : 107
OsaFLA23   : ATVKLS-SSSGNVARVTKTI-QD-AD--------------PHAVYLIDAVLMP :  93
OsaFLA12.2 : VAIDTGI--VQ--ASITRTV-FD-QN--------------PVAVFAVSKVLLP : 136
OsaFLA1    : VSMDTGM----DKSRVASTV-LD-DT--------------PTVIHTVDSVLLP : 132
OsaFLA5    : VHVGSMW--SN--PKISSSV-YS-TR--------------PVAVYEVDRVLLP : 135
OsaFLA8    : VNISTGV--VN--ATVGNAL-YT-GD--------------NLVVYQVDKVLLP : 136
OsaFLA27   : ISVQSMW--SR--PKISSSV-YA-TR--------------PVAVYALNKVLLP : 134
OsaFLA26   : VAIDTGV--VQ--ATITRTV-FD-QN--------------PVAVFAVSKVLLP : 135
OsaFLA7    : VNVSTGV--VE--VTVTNAL-SA-VK--------------PLAVYSVDKVLLP : 138
OsaFLA14   : VHVMSRW--SS--ARVVGSV-YE-SA--------------AMAVYELDTVLLP : 134
OsaFLA2    : VTLNTGV----DKSRVAATV-ID-DT--------------PVCVLTVDNLLMP : 132
```

```
OsaFLA11.2 : VTLDTRV----VNSAVTATV-GD-AE--------------PLAVYAVTKFLKP : 132
OsaFLA9    : VNISTGV--VN--ATVDNTL-YS-GD--------------RLVVYQVDKVLLP : 139
OsaFLA3    : VLLDTKV----NSASVTATV-KD-AD--------------PLAVYAISKFLQP : 132
OsaFLA18   : VNVSTGV--VD--TTLGTAL-RA-DQ--------------PLAVYSVDKVLLP : 136
OsaFLA13   : VRIQSGW--AT--AKLVSSV-YS-TS--------------PVAVYALNRVLLP : 139
OsaFLA15   : VRVSSGV--VEAEL--GRPL-RD-GH--------------PLAVYSLDAVLLP : 138
OsaFLA19   : ----------------------------------------------------- :   -
OsaFLA10   : VTIKTAA--SGDAARVKSTV-VD-AD--------------PVAIFTVDAVIEP : 135
OsaFLA6    : VNISTGV--N--WALLSTVV-SK-DF--------------PLAVYSVDKVPLP : 135
OsaFLA25   : ----------------------------------------------------- :   -
OsaFLA20   : ----------------------------------------------------- :   -
PabFLA22.2 : VVFGEG----EEAAHLFDHDIYI-DG--------------RISVQGIDKVLFP : 105
PabFLA9    : VMFGHG----ETTAHLYDHDIYV-DGRISVQGIDKIYVDGRISVQGIDKVLFP : 119
PabFLA17.2 : ----------------------------------------------------- :   -
PabFLA15.2 : VAVDTGI--VQ--ASITQTV-FD-QK--------------PLAIFAVPRVLLP : 135
PabFLA5    : VAVDTGI--VQ--ASITQTV-FD-QK--------------PLAVFAVPKVLLP : 146
PabFLA20.2 : VAVNTGI--VQ--A--------------------------------------- : 112
PabFLA21.2 : VMLNTRI----SKATISDTL-LD-DQ--------------PLAIFTVDKLLKP : 132
PabFLA16.2 : VILNTGL----SKSTISSTI-LD-DQ--------------PVVLYTISGVLLP : 132
PabFLA10   : VTVSSGY--VT--TPIISTV-HV-TD--------------PLALYTVGKVLLP : 136
PabFLA6    : VTLNTSL--SK--ATISSTL-LD-NL--------------PLAIFTVDKVLEP : 135
PabFLA11   : VILGTGV----NSAVITSTL-LD-DQ--------------PLVVFSIDKVLKP : 124
PabFLA18.2 : --KP----FSINHVGVKIPDAFT-NR--------------WIAVHEIVRPFL- :  70
PabFLA19.2 : --KP----FSVNHVGVKIPDAFT-NR--------------WIAV--------- :  70
PabFLA12   : VNVSTGL--VN--APLTSSI-FS-QA--------------PVAVYEVNKVLLP : 114
PabFLA13   : VNISTGL--VN--TPVNSAV-YS-QN--------------PVAVYQVDKVLLP :  84
PabFLA8    : VILNTGV----DKATITSTL-LD-DV--------------PMVILTVDKVLKP :  92
PabFLA14   : VTVSSGY--VS--TAITGSV-RV-TD--------------PVAVYTMGKVLLP : 125
PabFLA23   : GKYV----VLNGRARVVSPDLYG-DA--------------NLIVHGIDRVL-- :  99
PpaFLA10.2 : VKFGDG----DDAAHIFDHDIYV-DG--------------HISIQGINRVLTP : 103
PpaFLA11.2 : VKFGDG----DGAAHICDHDIYV-EG--------------HISVQGINRVLSP : 103
PpaFLA1    : SFIG-A----GSNATVITAD-LR-VC--------------NSVVHIVNTVLLP : 141
PpaFLA2    : SFVG-T----GSNATVTAAD-VP-VC--------------SSIVHVVNNVLLP : 141
PpaFLA3    : VA--------------------------------------------------- : 106
PpaFLA5    : VVTI------------------------------------------------- : 109
PtFLA1.2   : VKFGHT----ENSAYLFDPDIYT-DG--------------RISVQGIDGVLFP : 105
PtFLA19.2  : VKFGHA----ENSAYLFDPDIYT-DG--------------RISVQGIDGVLFP : 105
PtFLA6.2   : VKFGSG----DGSAYLFDPDIYT-DG--------------RISVQGIDGVLFP : 105
PtFLA8.2   : VKFGSG----DGSAYLFDPDIYT-DG--------------RISVQGIDGVLFP : 105
PtFLA5.2   : VTLKTRS----ITAKIVGTL-ID-EQ--------------PLAIYTIDKVLLP : 104
PtFLA22.2  : VTLKTRG----TTAKIVGTL-ID-EQ--------------PLAIYSIDKVLLP : 104
PtFLA42.2  : --DV----LVLNGVPVIFPDMYQ-SD--------------WLIIHGLNQLLTP :  94
PtFLA37    : VDGS----ILVNRVRVSVPDLFL-GS--------------DIAVHGLDGIL-- : 105
PtFLA17    : VTLHTGI--GP--SRVAETV-LD-ST--------------PLVIFTVDNVLLP : 132
PtFLA38.2  : VAIDSGI--VQ--ASVTQTV-FD-QN--------------PVVIFGVSKVLLP : 138
PtFLA30.2  : VAIDSGI--VL--ASVTQTV-FD-QN--------------PVAIFGVSKVLLP : 136
PtFLA20    : LNISTGL--TN--TTISDTV-YT-DT--------------HLAIYQVDKVLLP : 134
PtFLA23    : VNITTGL--TN--TSVSGTI-YT-DN--------------QLAVYQVDKVLLP : 134
PtFLA18    : VHLDSGW--SK--TKVSSSV-HS-TD--------------PVAIYQVDKVLLP : 135
PtFLA10    : VNITTGL--TN--TSVSGTI-YT-DN--------------QLAVYQVDKVLLP : 134
PtFLA16.2  : VTLETKV----TTATITGTV-KD-EE--------------PLVVYKINKVLLP : 130
PtFLA34    : VNITTGL--TN--TSISGTV-YT-DS--------------QFAIYQIDKVLFP : 134
PtFLA9     : VNITTGV--NT--ATVANTI-YT-DG--------------QLVVYQVDQVLLP : 136
PtFLA12    : VHLDSGW--SK--TKVSSSV-HS-TD--------------PVAVYQVDKVLLP : 135
PtFLA7     : VNITTGV--NT--ATVANTI-FT-DG--------------QLVVYQVDQVLLP : 136
PtFLA2     : VNISSGL--TN--TSISGTV-YT-DS--------------QLAIYQLDKVLFP : 134
PtFLA13    : VNITTGL--TN--TSLSGTV-YT-DN--------------QLAIYKIEKVLLP : 134
PtFLA47    : VNITTGL--TN--TSLSGTV-YT-DN--------------QLAIYKIEKVLLP : 134
PtFLA40    : VNITTGL--TN--TSLSGTV-YT-DN--------------QLAIYKIEKVLLP : 134
PtFLA28    : VNITTGL--TN--TSLSGTV-YT-DN--------------QLAIYKIEKVLLP : 134
PtFLA39    : VNITTGL--TN--TSLSGTV-YT-DN--------------QLAIYKIEKVLLP : 134
PtFLA50    : VNITTGL--TN--TSLSGTV-YT-DN--------------QLAIYKIEKVLLP : 134
PtFLA29    : VNITTGL--TN--TSLSGTV-YT-DN--------------QLAIYKIEKVLLP : 134
PtFLA32/49 : VNITTGL--TN--TSLSGTV-YT-DN--------------QLAIYKIEKVLLP : 134
PtFLA45    : VNITTGL--TN--TSLSGTV-YT-DN--------------QLAIYKIEKVLLP : 134
PtFLA35    : VNITTGL--TN--TSLSGTV-YT-DN--------------QLAIYKIEKVLLP : 134
PtFLA46    : VTVSSGL--TK--TSISNTI-YT-DK--------------QVAIYEVDKVLVP : 134
PtFLA3     : VNVSTGL--VE--VQINNAL-RQ-DS--------------PLAVYPVDKVLLP : 135
PtFLA15    : VNVSTGL--VE--VQVNNAL-RQ-DF--------------PLAVYPVDKVLLP : 135
PtFLA14    : VNITTGL--TN--TSLSGTV-YT-DN--------------QLAIYKIEKVLLP : 118
PtFLA48    : VNVTTGL--TN--TSVSAIV-YT-DS--------------QLAIYQVDKVLLP : 135
```

```
PtFLA27     : VNVTSGL--TN--TSVSAIV-YT-DS--------------QLAIYQVDKVLLP : 135
PtFLA43     : VNITTGL--TN--TSLSGTV-YT-DN--------------QLAIYKIEKVLLP : 123
PtFLA33     : --------------PLSGTV-YT-DN--------------QLAIYKIEKVLLP :  98
PtFLA21     : ----------------------------------------------------- :   -
PtFLA26     : ----------------------------------------------------- :   -
PtFLA4.2    : VTQG----LAINGVEIAAPEIFS-SK--------------EFIVHGI------ :  89
PtFLA44.2   : TAVP----MRINYVRVKVPDVMR-NL--------------KIVVHAV------ :  74
SmoFLA3     : VQFGEG----ESGALLYDHDIFT-DG--------------HISIQGISKVL-- : 133
SmoFLA6.2   : VSFVTGL--NR--ADVTDTE-LD-TP--------------PVAVYAVNRVLLP : 131
SmoFLA7.2   : VSLSTGV----DTADVVSTI-YD-QP--------------PTAAYSVNRVLLP : 134
SmoFLA8.2   : LAIATGV--ST--ANVIATL-LE-DD--------------PVGVYALDAVLLP : 135
SmoFLA9.2   : ----------------------------------------------------- :   -
SmoFLA4     : ATLSRPPHQSPPLATILRNVVQE-PF--------------SITVYAIDRVLEP : 145
SmoFLA5     : VSVGWPSSSTVFSSEILGTVKEL-PF--------------NVSVVDVSRVLVP : 143
BdiFLA23.2  : VKFGQG----EGSAYLFDPDIYT-DG--------------RISVQGIDAVLLP : 105
BdiFLA22.2  : VKFGHG----EGSAYLFDPDIYT-DG--------------RISVQGIDAVLFP : 105
BdiFLA9     : AALDTKV----ITASVTATV-KD-DD--------------PLAVYAVSKFLQP : 106
BdiFLA1     : VNVSTGV--KGNNMLLSTIV-SK-EF--------------PLAVYSVDKVPLP : 107
BdiFLA20.2  : VAIDTGV--VQ--ASITRTV-FD-QN--------------PVAVFAVSKVLLP : 139
BdiFLA10    : VAIDTGV--VQ--ATITRTV-FD-QN--------------PVAVFAVSKVLLP : 139
BdiFLA11    : ISVKSMW--SK--PTISSSV-YA-TD--------------PVAIYSLNKVLLP : 134
BdiFLA3     : VSMATGM----DKSRVASTV-LD-DT--------------PVAVHTVDSVLLP : 132
BdiFLA12    : IRVKSMW--SN--PKISSSV-YS-TR--------------PVAVYEVDKVLLP : 137
BdiFLA8     : VNVSSGV--SE--VTVNNAL-SD-KK--------------PLAVYSVDKVLLP : 138
BdiFLA13    : VNVSTGL--VS--TMVGTAL-RK-EK--------------PLAVYSVDKVLLP : 136
BdiFLA4     : IRVVSSW--AD--AKVVRPV-YE-MP--------------PMAVYEIDRVLLP : 134
BdiFLA14    : VNISTGV--VN--ASVSGTV-YT-GD--------------RLVVYQVDKVLLP : 138
BdiFLA15    : VNISTGV--VN--ATVDNTL-FT-GD--------------QLVVYQVNQVLLP : 137
BdiFLA16    : TGVVSGW--AA--AKLVSSV-YS-TR--------------PVAVYALDRVLLP : 136
BdiFLA17    : VSVKTAG-KGKGAARVESTV-YD-KE--------------PVAVYGVDAVLEP : 136
BdiFLA21.1  : ----------------------------------------------------- :   -
BdiFLA21.2  : ----------------------------------------------------- :   -
BdiFLA5     : VSLDTGV----RKSRVAATV-LD-EV--------------PLCVLTVDSLLMP : 137
BdiFLA18    : VAIKTGASPGGGVARVEATV-MD-RD--------------PVAVYRLDAVVEP : 137
BdiFLA6     : ----------------------------------------------------- :   -
BdiFLA19    : ----------------------------------------------------- :   -
BdiFLA7     : ----------------------------------------------------- :   -
MpoFLA6.2   : VTIHSST-PDKPVATLQSTL-FD-AS--------------PLVLYSIDEVLLP : 140
MpoFLA1     : IDGG-A----DTQATVVTPD-VR-VC--------------GSVVHIVNAVLLP : 135
MpoFLA14.2  : VVFGEG----DDAAGVFDHDIFA-DG--------------RLSVQGIDRVMIP : 136
MpoFLA7.1   : L-IGPAASASASNATVVGTVLED-PY--------------DISIIEIDQVLKP : 142
MpoFLA7.2   : VTIKTGV--GN--ATVGEVL-YN-AN--------------PVTMYAIDKVLLP : 132
MpoFLA2     : IIA--Q----ESNATVVTAD-VT-VC--------------GSVVHIINHVLIP : 134
MpoFLA3     : IIA--Q----ESNATVVTAD-VT-VC--------------GSVVHIINHVLIP : 134
MpoFLA8.2   : VRLRTGV--SV--ATVLEII-YD-AN--------------PVTMYAIDEVLLP : 134
MpoFLA9/10  : VTLHTGV--ND--AKIIETL-YD-AR--------------PVTMFGIDAVLLP : 134
MpoFLA11.2  : VTLHTGV--SV--ATIQETL-YD-AT--------------PTTMFGIDQVLLP : 132
MpoFLA12.2  : VTLHTGV--SV--ATIQETL-YD-AT--------------PTTMFGIDQVLLP : 132
MpoFLA13.2  : ----------------------------------------------------- :   -
CcrFLA1.1   : -R-----DPSRRNPRPDPRN-VF-TQ--------------NGWVHVIDTVLLP : 151
CcrFLA1.2   : -A-----DESRENPVADPRN-FF-PQ--------------NGWVHVIDLVLLP : 152
CcrFLA1.3   : -D-----DPSREDAVVDPRN-IF-TQ--------------NGWVHVIDSVLLP : 137
CcrFLA1.4   : -G-----DDSRDDAAISTPN-IF-LQ--------------NGWVHVVDSVLLP : 136
CbrFLA1     : IKVDGST--SVTEA---DFLVDS-PN--------------KAVAHVIASVLL- : 131
CbrFLA2     : ----------------------------------------------------- :   -
CbrFLA3     : VLD--------GQATVTGPDVKI-AV--------------NATVHTIDTVMVP : 130
CbrFLA4     : AF--------IEGAKIVEPDLFL-TG--------------ESAVHGIDQIIM- : 126
CbrFLA7     : MVE--------GTARVVVPDAIT-GV--------------NATVHIINDIIFP : 132
CbrFLA9     : MVE--------GTARVVVPDAIT-GV--------------NATVHIINDIIFP : 132
CbrFLA11    : KLD--------MTAEVVQADAIF-AG--------------NGTVHVIDSVLIP : 135
CbrFLA15    : VFVDGA----QIFPSGGFDV-VT-AP--------------DVVVQMVDTLLIP : 150
CbrFLA16.1  : TF--------VQGAGITAADDII-RE--------------DGVVHGIDSILFP : 124
CbrFLA16.2  : VFVG----NAASLGQIVTPDLYA-NP--------------KIVVHGINKVLLP : 101
CbrFLA16.3  : LRVG----YSLALGSVVAPDLYA-TS--------------SITIHGVDTVLLP :  98
CbrFLA16.4  : LMVG----YSSARGLVVAPDLYA-TS--------------KITIHGVDRVLLP : 123
CbrFLA19    : RISDDD----GGEAFMTRPDFRL-LI--------------NSTIHVIDTVMIP : 135
CbrFLA20.1  : LT-------SVNNVEIIRPDVYM-FP--------------TKVVHGFSRIMLP : 109
CbrFLA22    : MLE--------DYAPITEPNALR-TS--------------NATVHLIGELLVP : 129
CbrFLA23    : VLE--------GYASITGPDALK-SP--------------NATVHLIGELLVP : 128
CbrFLA24    : VLE--------GYAAITGPDALT-SP--------------NATVHLIGELLVP : 128
```
