# Supplementary material for: Evolution Analysis of the Fasciclin-Like Arabinogalactan Proteins in Plants Shows Variable Fasciclin-AGP Domain Constitutions
Source: Int J Mol Sci. 2019 Apr 20;20(8):1945. doi: 10.3390/ijms20081945 (PMC6514703; doi:10.3390/ijms20081945)
Supplement: Supplementary file 1 [file ijms-20-01945-s001.zip › Supplementary files/Figure S4.pdf]

Figure S4. FLA protein sequences in 13 plant species. Signal peptide is highlighted in green. The fasciclin domain is denoted by blue. Yellow highlighted sequences are AGP regions, and the red sequences in AGP regions indicate putative AG glycomodules (Ala–Pro, Ser–Pro, Thr–Pro, Gly–Pro, Val–Pro and Pro–Ala). Red highlighted sequences are the GPI-modification sites.

Red algae

Chondrus crispus

> CHC_T00002202001

MTLRPIIILLLALLAAAAAQDGPLPPSAADDVPDAVVPDPRPTILGLLASDPDQFSVLALAALAVPDLTEALGDPNRRLTVFAPTNAAFAALTNKLSPGAGVDAADPEGILFALMGALPGPLPGRSPGEVLTTILSYHLLAFAAPFQELENDGTAETVQGTLLRFRDGRVVDRDPSRRNPRPDPRNVFTQNGWVHVIDTVLLPFNLETALAPLTPDGPDPIPIEEAEDVPDAILLDPRPTILGFVESRPDSFGILARAVGTVDSLVKALDDPNSRFTVFAPTDEAFTALANQLVPGADLNPADKDAVVEALVSAIAPLADVEAAANSTIESILGYHALPFAAPFQELEDMSSAETVQGDALRFADGLVIDADESRENPVADPRNFFPQNGWVHVIDLVLLPFDLDAVLGPSEPEMPEEGQPIDISEAEDVPDATIPDPRPTILGLVQSDPEQFGILGRAVGTVDSLVEVLGNPGSRLTVFAPTDEAFTNLANTLVPDANLEASDKDAVVEALVTAIAPLADVEAAGGATIESILLYHALGMAAPLGKLEEMKTAKTLQGGVLSFNSGSVTDDDPSREDAVVDPRNIFTQNGWVHVIDSVLLPFDLGAVVNPSEPEMPEEGQPIDISEAEDVPDATIPDPRPTILGLVQSDPEQFGILGRAVGTVDSLVEVLGNPGSRLTVFAPTDEAFTNLANTLAPDANLEASDKDAVVEALVTAIAPLADVEAAGGATIESILLYHTLGEASPIQELEKSDTLTTLQGDSISVADGKVTDGDDSRDDAAISTPNIFLQNGWVHVVDSVLLPFNLGNLASAASPSISPEAEASPNISPEAEASPDEDDDDDGVCFPESATIRLADGSSVPMHLLEAGTEIHVGREGKTSRIFAFTHKNRVRRTLFYALTTASGHSVTLTGSHYLYANGKLTAAGAVQIGDMLDTRAGNSQVVRVGRVHGKGLFAPHTLHGDLEVDGVVVSGYSRALNPTIAHGLLAPVRLLTKYAGVVEPLGSLFYDGADRIASYLPKGSRRYE

Green algae

Chlamydomonas reinhardtii

>Cre09.g393450

MGHIGLLLLALALLVVAPGALSQSSPYDYVSSRTDMTFMKKCIDNLLPKNKQPWTATQNKALTMFLVTDEGFRTTFSGLNIRTTLGTDPERFCTVVTKKMNNTRNAIFKYHVLAANLGARTRAQLPGNCPVGPDGLPGCPYNTQAGGKNLLDVITVAEADDVVGTDKTYLIGGVDANYADYVKFPNGTNDYDRAVKGRILHTINALLVPTRYVPIPKSPSPSPAPKPAPKPAPTPSPASSPGPVISSSPSPPPPPSPPPPSPPPPSPPPPSPPPPSPPPPNPPPPNPPPPPGIASPPPPPSPAPSPNPPSPRPPPSPYAGFKSIYDFLSQRVADLSRVKASVDAADLFLQLSDPSLAWTCFFPDDDSADWNQLWAGVYGEGRNILQQTQYVNQCWDSVRAGALTNPKCPNATREYVGVMQNVYLQSCSPSAPLVSTDVWANRANLSTVAPYSGLTTWRMGPNPANASTTLWVTTVFDPFPMPNSGLLLPNVLNAQGVVAIRKRDNLVANSMVQVISSFLGDYAAAPPPPPPPSPAPPSPAPPPQAFATISTALLGQNALSVTRQVITLYNLTQRVNALADTTCYFPSNQAWALFAANGNDQGAFISVAANGTFTPLSRHRRSLLQSQPLPQHVTAIRFCALGFCATADLTTAGASGAAAQARRSLAMGPNPFNGSRFADTLTWNVTLEPADSYNRAIMRNVILQSCYNPPSAAGSQNAGANAVRRLVDTDEFAWETALGWGSSTGIVVQKTPYTGPCGNNVTVSGVATSGALTNLPNVDLEALGYCTQCAQSDPATGLYAGCAFTVYSIATGGASGFDPNPTNTSASVITGLGYSRARSPVAYWVPHDIVVGTYDAPKGYIQLLDRLVLSPRLPPPPPSPLPPSPPPAPFPPPPFTGLRPLLEGNSNFNDCAAKLFAATNYYPYVEQFDTSGWTLFIPTDDGCQLGLQTYDSTTYSGTLAQNIATAIANGYARSLVKNMFVVNAYLSSGAINNFTSAVTDQGLFGNSAIGTNTLIFRKTGAISLFQRYPGSSGAFPGGFPQMTADVLIADIPVQRNPNALGGVGLAGYVHMTSMLLTPYLVAQSPPPSPAPPLPPPSPPLPPPPTAFPNGLQDYLNANPELSILNTLLSCTNSTTTIQNLLNQGAQYTLLAPTDTAFSNYLNSAGIGGNWRDVLCNTTNGRAAQTADLLNAHFIAGVYFGVNITANNNTAANLYTYAFNASYKIQILNLNGLYTVRQQMPITSQTFVSGKYDNAINGTAAVAHMITAVLALPVPFPPPPPPPPPPGNGPAGAAAVSVYVGLRNEPEAALFVNLVDKFNLTLPSGVSTPATAYLNTPDTVATVLVPS

VNAVNAFLGRVSFAGTPMSYADCIAPSGTPQQVAAKLAVCGGIVQFAVLPGTQFLPSFTAADYTLNPPIVA
DGTFKYILIGNNTGAGSSATKIAYRLDTSTNPIVTTSLAFQTTTSPPCSGLAVTSKGPIPAGVPNFLTKQQGMI
YVISQVLFPQEIYNSAIAGSGATYAGCPP

>Cre09.g394200

MGRLSVALAVAVALLAVLPGGVLSQSSPYDYISSRSDMTFMKKCIDNLLPKNKQPWTATSNKALTMFLVTD
DGFRTTFSGLNIRTTLGTDPERFCTVVTKKMNNTRNAIFKYHVLAANLGARTRAQLPGNCPVGPDGLPGC
PYNTQAGGKNLLDVITVAEADDVVGTDKTYLIGGVDANYADYIKFPNGTNDYDRAVKGRILHSINALLVPT
RYVPIPRSPSPSPAPKPSPKPAPTPSPASSPGPVISSSPSPPPPPSPPPPSPPPPSPPPPAPPPPSPPPPNPPPP
SPPPPPGVASPSPPPSPAPSPNPPSPAPPPSPYAGFASIYDFVSQRVADLSRMKTAIDNVKLTARLSDPALA
WTCFFPEDTSISWGLNWPGTARNYAEQGAFVASCIANPAFAPTCNTSVIEYTGVMTNVILQQCTPAALITT
SNWVNSTTNYTTVAPFGGLTIFRSSNLANATFWVASKFDPNPQPISGVLAANNVTSTGLIVIRKRDNFVG
QSVVQVVTSFTGNYAAAPPPPPPPSPVPPSPAPPPIAYGSITSAINGVAGLQITRQLISIFNLTAKVNALAGTT
CYFPSNQAWALFGNNVNDLGAFVNVTANGTAVPLSSRRLLSAMEDGDNLQFCVPSHGFCAMASTVPLR
RMLINIPNRFNATQFLDLLAWNVTVEPSGVASMPVFKAQILANTILSGCYNPPTAPGSQNVGNAVRKLVD
TDNFAWETALGWSVDVGIVVQKTPYTGTCGNNVTVTGTATSGAAINLPNVDLENLGYCTQCTTYDNDTM
LYSGCSFTVYSISTGGASGFDTVNGVQQITTLGYSRGRSPVAYWVPHDIVVGTYDNPTGYVQLIDRIILSPRL
PPPPPSPLPPSPPPAPFPPPPFTGLRPLLEGNSNFNDCAAKLFAATNYYPYVEQFDTSGWTLFIPTDDGCQA
ALTAYDPATYNGNLTQNVNTAISNGYARTLVKNMLVVNAYLSSGSINNLTTAVTDQGLYGNTTVGTNTLTFL
KTSVISVSQQYPGFPKMTSNITIADIPVQRNPNALGGVGLAGYVQMTTMLFTPPLAFQAQSPPPSPAPPL
PPPSPPLPPPPVAFPNGFQDYLNANPELSILNALLSCTNSTTTIQNLLNQGAQYTLLAPTDTAFSSFFSSNNI
GGNWRDVLCNTTNGQNTKTAQLLAVHVIAGANFAVNITANNNTAANLYNYAFSAGFKIQLLNLGGVYTV
RQQMPITSQTFVAGKYDNAINGTAAVAHMITAVLALPVPFPPPPPPPPPPGNGPAGAAAVSVYVGLRNEP
EAALFVNLVDKFNVTLPSGVSTPATAYLNTPDTVATVLVPSVNAMNGFFSRIAFSGTPMSYADCIAPTGGA
NVVAAKTAVCAAIVQFAVLPGAQFLPSFIPATYNVSGAATVGVSDAASMYAALGNVTGYGATTASKLYYKL
NTTPPVNAATLAFQTTTNPPCVGIASNIKGPIPAGVPNFLSKLQGMIYVVSQVLFPKEAYDIAVNAGATFSG
CP

>Cre06.g278170

MKQGGLCAYLALLLLAGVSASYADMVVTKGSGHRPPPRRVSPSRKPPTAPTTYKSILALAAARKDLSIANA
VLKKTAGPDFGPNAPPATVFLPNDEAFKLLLKALGVTQAQLLALPTKVLTEVIVYHAVPGMPLAAKDLKPK
QVVETALPGYNLTITKDSAGVMILAAQSKAKVVTADLKAGMTIVHIVDTVLVPFDVLNPPVPPGAFDTPGG
ALTELGLEYLVGAIEAAGLGGVLNRTDVIGTLFAPTNDAIIRTQTALANNSLGVNLMYPQVVQMILGDHVH
LGEKLMAADVAMRSTIPVGWAPADYATPDAHILTVTFNPAGPSVTLSGFPGNSAKVIKADVLVGTRQELVV
HVVDGVLITTQTARALGLIQANGTTAPVPTPAPTPAPSPSSSGTGTVPVSTVPNLGAAIATTPDLSQLLSKAL
AAGLGNQVATANSPLTMFAPNNAAWAAFESMLNAKDPRLTAVANQIIALIISDHVHVGDSYNANALTNG
QIIPIGMDPTKYMDANATTVRVTVNATGVFLAHQVSGAKVLRTILAGPTVVNIIDSVLLSRASAAALGLIEM
PSASPTPTPTPVPTPSPVAGDTILGVISADISLAGIGQLIALTGLAEPLSDPDIEYTLFAPVNDAVTTFAAELQK
IQTYNPANPQVLQLLYQILATHVYMAGVLRSSDLTDGQVIRTGVANTPATNVTVTINSTGVFLTATNTVKVIA
ADIAAGKSIVHKLDGVLVSPEVARLIVELSAGGGASPAPSPSGTPSSANYSSVLAALSSNTDLIGFNGLLGITG
ALQQLSSPNLVATFFAPTNEAIEQAGDLLASLGTNPVFTPQIQQIVNELLGSHIHLGEALTYADLNMDGKKIP
VGLNPAAYTAADNTVLTVIANSTGVYLQSTNIVMVSTTTLTAGKSIIHVIDGVIVSPKLLAMLPPPGSSPAPT
PASTPGEEPAQSPSPSPSAGPASGTFSDVRQALTSTPDLFGFNGLLTGNAVVYAQTNNPNLVATIFAPSNAA
LENSEAITSLAAGAPDTTQILLRYNELLGSHIHIGEALTYADLNMDGKKIPVGLNPATYTAADNTVLTVIANST
GVYLQSGNIVMVSTTTITAGKSIIHIIDDVIVSTKLLSLLSGPSATPSPSPSPEPADPTYDDLPSALRGQGFNHL
ADLYDAAVNSAVGPILQSLLSAPITLFAPSDTSIDEYLSSQSTTFEAFLADSKTNPINIAGLVLPHIHIGHAYTLD

EFTVGQVITTSTTGLEMFGAQLTVQEHDAKVVFSTSVGPVTVIGGIIRAGTGIIIPIDKVLKPDV
> Cre16.g679445
MATLRRAALLLVALFAAVSVVNAQEAAQKRNPANTAASTGSGPAVDIAIQALNATGVSGKLNASTVATIFA
PDDAAFISLASSLNFTSREQVFASPFATNVTLLHIIPGVALRSTDLPAGQTVNASSLLGPTLEVTRNGNAVRV
AVAGNPNSSAEVIKADIPFNAAIVHVINKVLLPPSTTAPAAAASPSPVTAAPSPIILAPVPGGTANETTVGGG
GNYTGGAYNTTNATTGGV
> Cre02.g095130
MTLASFALALLLGLSLAISATDAAGTAETVLVPAPCSAFTGANSLDELARACPELSTFAKLLATADNYGKWM
LDCGKRCGTLQKLPRTVFLPVNVAWQKYFTATGTTLAGLTSKKSRKALTALMRYHVLAQVVTSSRVPLAVD
AVGSSAAGSSNNGSSNNGSTIGSVAVSSSGGEEVETLQGGSITLARRPNTSTIRVEGSRNQAVVLHADISL
GTPVVHLVSDVLLLEADPPPPPELPSPMPSPSPPPPSPWATPAPSPAYASPTPTPTPTPSPASGTGGTSPKPL
SRPGTPITGSTIFNLTWDRNVDMDLIVVASWPETDTTDTMTMFYGYTGPEALLGGGAMSASTSGGSGAL
TGFYSRTAGSWEALAWPNPLKPAPAVYTICSFFYNLEWAPGTVINADSPVTPAFSVITGADQNAGAGRGT
AGSSSSSSSSSSSSSSSSSSSSNGSGSNSGEVLVGTLSRPLWKPGEFSFTLLPRNCSPDCAWYLGQVCVDGA
GKVRVVSGTERC
>Cre17.g713150
MCRIRTDITRPSAFLAALAGLSCVLCVFAQAPCSNLTSARTIEALTKRCPTDLSSFGTILAKIPKYAQWLLDCG
TGACDNAQRTLLMPTDMALDTYLKAKGTTLTKMLAKTGGGRKALQAVLRCHVLQPSIALTSVNITAAAAP
LSYKSLQGPSLTFAESSSGGVTVECGANSVAEVVHADITTGKSVVHLIDTVLQCQAAPSPPPPPSPSPPSLPT
PNSSPSPPSPETPPPPSSPPPSPPPSSPPPPEPSLPDPPSLEPPSLNPPSPDPPSLPPTPPSAPLTPPSPPSPPL
PPSPAPSPPDPPSPPAPPSPPLQPSPPNPPPPPPWYSLGGLDSYPFCAGEVCSRPQLCTAERKCMCGTGPG
CGGNTACWATPDMAGWPTPPAASCVVQERICSTWDGNPRLCLPDFFCYKDIYCMSRGQLRIMLESWTS
VGDLDMWVTNPLNQTVWWNSDGWNADETRDPKWLPYGFEDNGYAVLDTQSDQYGPENVYYIDKN
WQDGTPAAPLPSGNFYVCARVYIQDLDPSVWLKFPVIVTTIAESAKYGINMRTVVTLRDANFEPSTPTLES
DCTASSPSLVMTIPIP
>Cre17.g745097
MGKGACWIAVLVLLAATGGRSQDPPSPEPSPAPPSPEPSPLPPSPTPPSPAPPSPTPPSPAPPLPAVTYNSV
YDYIVKRRTTGLSFTYQLMQLAATLAPEQVAAINNSSLAVTAFLLSDTAFDAFARKWGYASTAALQVAGFGS
PAIQARTQQLLNYIYVNQAFRLADLPNNASNPVDNTTTTYFNTQLTETGLASYFSRIGVRRNTNDSRPLAA
QVVGSTTAAGLLEVDVPAGAAYINLLDRVPQFYWDTPYDVLKADPEFSTLVSQLDNVPTLAGVKASLQRA
NLSQTFLAPTNAAIAKFAATYNTSLDDPLLAHTLYSYLRLNSTKEVVSTDNITTVPSSLGDTVTFSKSGQAPIV
KSFQPNGTWANVTVPNIPTGFQVRSFVHKIDAVVVPLPYTAPQPGAAAAAVTFASIWDYVTSRPDLSIVVI
SLKSLGQETLLRNASLDYTVFALTNDAFDNFAKVIGLNGTADIYQRPDLLTAVIETQWYHMVPQVFRVGDQ
PLNSTSYFQTRLATVLGKVGNYSRVGIQRGGAADPAFVVSTSTTANLVAQDIPAGAGYVNVVDRVLQYYYD
TMYDAIKGEPDMATLATQLDASPQLAPIKAVLSTPNITRTFLAPTDTAVARLSSVYGLSMNDTSYGANIYGY
HQLTDARNLDSNYPGSSSAMSSLGCCAVNFTVTRNNGTTASSYLRDGTRATVVSANIPVGFGPVAFVHKI
DQVLLPVYTSAWHYVSQQPDHRALSALLALEGSVQGELRAPSPPLSLLAPTDAAVTKAAAAYGLTNLTQLL
TTTALQKAVLANHALSGSYPLERLNGTLDTRAGAGYLNASRSGSAITITAARSQAVVIQASSNVSILYGRAYV
HTVDTVLLPKEVPAPGDGGGAGSTAPSLLALLLAAVVGRATTWLLA
>Cre03.g175300
MPPQGRRFDPVWKRATFFCMLPVRGRALARHAGSSDTSIGSISTSGSSSGTSSSTSSSSSSLDDDIPCDGT
SLVSALMMQPNLTTYLRALRAGELMGRLNDVQAPPVTLFVADDSAWQVFAADAGTSVDALLTEAGGAL
VRHLLLYGLVPTPLSPQQLAASPVLNTSWAGQMVFVPQPFMGAVSSSAGTEARITGRVKCESYAYRVAY
VLMPALSLTALPAFSEARAAPPPPPPPPACANASIPDLLSANGNLTTTLGALQVTGLEDQLYAPGVAPYTLFA

PTDAAWEEAAAQLQLAPGKDVLQQLNRTVLRSLLLAHLLPGNLPGAQLRSQIYTTAAGTAASPIFLSTAGLE
AAPPPPAGGRGNEPDPGIALQTRDADAVVVVADVGAVGCTASVHAIDAVLLPDAATLATLVVAAKPGDR
QQQATRKLLMAAGV
>Cre16.g687742
MAARRLFVTILVAVLGLGVRGQSSDTLPYISLQAYLNGEPALSMLLQLAIPSYSLLSDTGLKATVFLPTSDAV
MSYLYTEFGVSDLADLWSNAVLNQRLLGEFNLRVLPQMIVKDRLLTAQQMSNGMKLRTNSSEEVTLVVSK
AADGTMTITGPQNSAKVVKANQLLANGKVVAHLVDKVLLPPDIVETPPLLEDSPPPPLRSPRPSPRPSATS
PRPSPASSPSPSPSPSPSPSPSPSPSPEKRKKSPPPAGRPSPSPRPSPSPSPSPSPSPSPSPSPSPSPYPSPKKK
PSKSPSPSPSPSPSPSPSPSPSPSPSPRPSRPSPSPSPSPSPAKSPSPSPRPSPKPSPKPSPKPAPKPSPSPSPSP
SPSPEEVPEFGSFPNLQAALSAIPQLSLLSLLYDTAVQDSLVRIPLDAIMLSPFTLWLPTNQAFEAWFADPES
PWHWQNDLGQAVGANPRLLLTLLSPHALAGNQLLLSDPNDLHDGSTFTTGLKVLGRPPPGAQFVVRRD
GDTVALEGTRGANTGVVINVLRLQAALVGKAGVVHVVDRVIVADNSTPASHRRQRRRLGHQEPQQEQ
LHAR
>Cre03.g166250
MGTQLRPSRRAGSSPAATAGGSSSSHGRARLSPRPGFLPGGRLQLLAVVGTLLVFGAFSAMGQFSAAPAA
APAPAPAPPPAAAAPPPAAAPAAKPAPPPPAGTAAATPGALADASDGGAAGAAGAPAAGDDEDNPFAPP
PLCQGKTPAKVVIDRVVAYPAPGASPLVVLRNVGGQTANLTGWRLTDSDTRSVPAAANLVFGADPTNCGG
VGNFTIDPSRILALTPKNDSTNPCGFPFSLSFRDEVNLFDPSGKLTATAKWANADMGSALHLQPDGSYQSL
PEMEDVLAVLRAAGSHKILLQALEVTGMDKLLVAASAPDYTLPEKPKPKESTDVAPQFPWWFGFSVDRSK
LPPPPPPPPPPPPPAGIPAAGPFTILAPTDDAFETLLRQLGGGGRRLPLDLFFQLPELPDILQYHVVPGLYTT
EYMFNNTPIFTARGVEVIPFNDPCMTEGVVMLHDNCVDKPTPDNYTCEEQVKFDKCYFPFMISALAAQ
WQGGFCQRTCQRCSCAAGTGVQCASLTEPDMLASNGVAHSISRVLFPPPAFSKADYVNMTTSINITKDTP
GDAMVPGMASGGAAAGPTAAAAGAGAPGGAASAFMAAAANAAAKAAPAAAPAAAPAAAPAEPAPA
AAAAPAEPAPAAAAAAAPAEPVPAAADPAVSATKK
>Cre16.g687854
MNIRGRNWIGAILCLVVVCGAPSSAYRLAANRGLSWNSLAAGASVEAQAASELLERRIAQEGTTNSKLPP
GSFDFPPEGAPPTEHEPQAPPSPRGLQPSPANSSAAPDSSSPNPAPQPSPGEPGTLPAAGAPPPGSSPWV
MPAPSYPPPPYGGESPEPCIGGSYGYGGYAVAYGSYGYGGYGCHTPPSPPPYGGYYGSTPPPPYGGYGGYG
GYGSYPPSPEVQPSPEPSPSSSPSPEGNPSPSPDENAPSPPPERWTTIWRYLEDVVVPDLWPFLQAAMDA
RQKAYLQDDQARFTLFVPLYEDMLKDELILSIFANPADPRNAMRLNRLMAYHQVLDVTIYPQDLQDNMTL
TNMLGEQLHITQKLTEDTNEFYVDGVNIARWQVGGRGIVYWLNGVIHEPVAPSPPVAGPNRFGTLGEAL
TSLPQCSIMSALFAGVSADPAYGPLVNALLANPFTLLVPTDTAIATFLAGMGLGPSDLLTMAPEAVAAIVAP
HLVVTGDAFLPAARLTPGAAVVTGLPPVLPSATLVVQAAAEPGALPVLAYEDGGAPPLHVLVADVFVRAEG
DPTEAVVHVVDAVVTAPAGGAGRRRRNRRALSSH
Chara braunii
>CHBRA165g00180
MAAAESCSAMMLVATLLLALYACELAPSVNARTGSHMRPLDSLHFFRRELKTHRERMERVLVEVTEPPPGGPA
RTITVALDGSGDYKLPSEAVASLGTKSSAPVTIYIKDGVYRDRFNVVKRRWNVTMIGNPEDPSRVVIVDNRHAG
KMGADGDDIGTFQSATATFGGYHIIVQGISFINDFPRGKPGEIGNQAVAMRASGWFIIFANCIFRGFQDTLYAHK
GVQLYKNCYIEGTIDFIFGYATAQYEDCHIHTLADPFTISAEKRQLPGARTSGFLFLRGIITGVSGKAYLGRAWGAA
SLTWFVETQMEAFIQPAGWNNFNDPTREKTADYGEYNCSGPGSDRSGRVSWSRALTLEEVGPFLGGDRQWI
DGNSWVRLPSPLPPTPDGREDAATTPLPISSGSPPPPPPPPPPASVVPPTPPSVEPPVLPSGFATAGLEPAAASG
NATGVIAALRADPRFSIFVGLLDKGFAANLPLQPLTISVPENAAFARLGPGQLDTIKADEALLYEFLGYHFADGFYP
KADLLAAIAKSTSGTVSVPTQGGSYVSFSSVGDVIKVDGSTSVTEADFLVDSPNKAVAHVIASVLLSQDVTDSLAS

SSSPPPHPV*
>CHBRA1815g00010
MKTAMKLVALVALTFLMSASPRASMAKAAFMNAPPSAAAPSTAAASGAAPSAAMVKTGKDAKVFEILKNEM
QFSMFYKALVDTTEIWNVEGRTVLVGEQLILFAPTNAAFSTALSEEVITCLQKEPGQATLASFLKYLQVVVPNATV
PQVKTPKQLLKVTKDDFLWTVGGKGIA
>CHBRA83g00340
MARPADSTRLALSSFLLFCLAVWTAVSGRLVAAQYPYPGAAASTTPPPYYYYPPPPPPASASYYYYAPPPPPNPFA
EQLMAELQAEPEFQRFYEALNMSRSIDMVKGLVNSGVTLFAPVNGAFDALGAVYTECMLKRPGMDDLLPLIV
RFHVATGNFVNAQLMTMSSVQSFLGLPIPLTHAAAGGLVLDGQATVTGPDVKIAVNATVHTIDTVMVPYTARR
ILELLCYNAPPSSPEEANQVAMWRMTGGG*
>CHBRA1065g00030
MKLQGGGKERGGKGGAEEGAGEEEWEELERSQSGDMAREEEREKEGEEERGEDQEKEEREEEQERSGSGIM
AGSFSRCLAEVERAGEEEEECEKWEKERSEECEKEWGRSGEGVGKERGRSGSGAMAREEEWEKEWEKEWEN
EGEEERGQEQKKQLEKEETEKDQEGEREKEREQEREEEREEWERGYGEILITFFGGVGGAGEGAGGGVGEGVG
EGLQGETGSPCFLSMRCRVCADAIRRCPLLYMEGGAEVEGRRIARQRDLVMWVRKRQDEAAKTESGAKLTRG
GLGRNTAAAALTDGQVAAQRTAASPLSPSASDEDAIDISISSSISSSSLSPQQKGKEEVLAAQDDEEELKATKRGL
VRPRLDHDTASLPPPLLTPSPFASGRQPTIDASGAAVAAPAIEGRDNSSAFSEEKGRPPPLTPEVTPQEGGNEAG
PLMPQPPVDELDGSDSSFSKLQRQSTVESQADEGEATTPASPPHLSYPTLAGHHAGNTQISVPSILDSTDIAAIA
NTVWSAAESATFGGDSLDSSTFQEEGEAEKVQPSVGAPSPPLTADVPTSHAAPPFGLEGSNLQDGTVVGQLSQ
TPLPVLPSLVPGSGPAEGVDLQIPEIAEVSTETIPPLAVLPSDDVWTESEGISLDKGQPSESAEGRVVAEPPSKSPS
ASRKSLGWPSPSPSPSPSPSPATVVAARNRSSLPERGLQGQLLKLREIALGKLVPIRDSLQPFRSPSSSSSSSSSPSS
SSSSSSSSSSSSSSSKFEGVSAQPAPFDFDLDQDLNLDLQEPPPKLAFSPYKSTGDREVGEVGEVGSASPGSSPSP
GLSSTAGANLEDSELALRRSNSSNPGETHEQPMPVVAAQASLQPGVHLQDFPVDNNYVATARSVATAKALESL
GMSSTAGGGGAEASRPLPWEIIGADGAPAPQSRGIESVSIPYQPYMILPPPPPPPPPPPSASPSLESQLSPAPAIT
VVEKTRTWVSSLSPHSHPVDPLPSLSPSPSPVFRSVPPSRSPQYTNVLPPSSEPPTGFHIARPLPLVGVAKVALPPS
LSSREEEDSASSLSQPRQRHSSSPPPSTAFKSSRASSSPLPSPNSLPTTLSSGTPASLQKPLHVRNVNGSAGIAPSG
SPNSWSIESAAPPSVSHVSVAQATTVPLLSLLDGATAYANFVEAALTTRGATVSILLDDSNVTVFAPINSAFDKLGS
SLVDCLLSDSSSGLLDIILLNHIFEGGHPSKSLTDGQKLLTVANTTAEIAVNETGAFIEGAKIVEPDLFLTGESAVHGI
DQIIMTSKLQNEIQEMCGGPKLAPQSMASQKVSTSSPFPEDWRPSVSTEGVAIPSENLPKSFITSSVSRAEVSAL
TAGLWSVASLTIFSLLVSLDCSWTAGIL*
>CHBRA869g00060
MVKMMGTLSSLRMIAAILAAALISTVVAQPGRPSLPDLIDQSASRYNLSMYVEMLRSSSEALRTLEMQRSAGG
GLTVLAPTNEVVAACLHRLSSCLSGQEMEQVMTMNLKYCALPGRFEEADLRLSIQQSGGQIHLPALRGGSHQL
AVSEGPSKYASAQRGLFVDNAQVLPDGFHVVSTPHVIVHVIDKSVVAPDVRAMINKYCPTPAESAPSPSRVPG*
>CHBRA837g00140
MLFKRWRVSVKKPEGVTIFAPTDESLLAAPESVTQCLRQSPRSNEIASAFLLSHAIPGSLPIDAQTTKVSQSPNGK
FTGKFTGKFMQPTLAGGDSSAVVIRQAASRYPGANTGIFVDDAQILPDGSNVVTASGLVVHAIDGPILTPHVVQ
MLSEACPGAVPSAPPPLTPVDGSRSARTWGGGVTASLRYSDGATRREEERH*
>CHBRA06g01550
MAQSRLVRSVLLTMAAAAVFIALAGIVGAQTPAPASYVPPVYTPPPPPPPSPAALELLARIQGLGDLSMFSNAL
MASRINLTLSGIVGGGRVTVFAPNNGAFAMLSPEVKQCLDTQSGKIDVLTQIMLFHLAVGGNYTAAELKTISRLT
AASGMPIDLMTLGDGTIMVEGTARVVVPDAITGVNATVHIINDIIFPESILSTVMIECYNVPPSASLPVPSAGFF*
>CHBRA06g01510
MERDGENRLAALMLGLMCVVAIAHVGAQVVAPPPPPPAGTDIVSLICAQPGLTAFCSALNTSRVGLELLQNVA

VQRVTVFAANDAAFGSVRADIKACFDREPIDVLSQVAAFHIATGWNYTAAEMASVYVLTTLHGMPLMVGRNV
NGSPLIEGTASIVGPDAIHTVNATVHVIDDVLIPENLVRTIWEECFGVPASSNTPVPPASSI*
>CHBRA06g01470
MAQSRVVRSVLLTMAAAAVFIALAGIVGAQTPAPASYVPPVYTPPPPPPPPSPAALALLARIQGLGDLSMFSNAL
MASRINLTLIGIIGGGKVTVFAPNNGAFAMLSPELKQCLDTQSGKIDVLTQIMLFHLAVGGNYTVAELKTISRLTA
ASGMPIDLMTLGDGTIMVEGTARVVVPDAITGVNATVHIINDIIFPESILSTVMIQCYNVPPSASLPVPSAGFF*
>CHBRA06g01400
MAAHLAAVFAVLLVMSPVLVHGGPGHDLLEAVRQHPRLTMFYQALVTTEYSKELSTSVDDKPVTIFAPVDEAIQ
AATADAFPWDCLTNTIAGKAALKDTLKHHVLKGRFYTVYKLTQTNQVIPADGFPIEVQYDTEKIVITLDGYAHVIE
PEVILMENAIVHLIDAVLTTENIYADVLDACAEAPASAPSSLGFADGL*
>CHBRA06g01390
MAPLYVRSLAAVFTVMMLVSPFSVRAQLVPGGDLVTSMANYGLTTFVSLLKLAKVDESLKGIAATGPVTVFAPSE
AAFAVLSSAYPAQLACVTTGQGIDNYLSQILKYHILPNGNFTASKLTKLTQVSTLIGLPIQLEYTLPSGPLKLDMTAE
VVQADAIFAGNGTVHVIDSVLIPETILPALMDQCGGAAPSSVAPVPGL*
>CHBRA741g00090
MERGNGEKLLVALILGLVCLAGVAQVDAQGVVPPPPPPPPPPPPSTPVGTDIVSLICATPGLSAFCNALATSRVSLE
LLQNVAVQRVTVFAANDAAFGNVRANIKACFGKEPIDVLSQVVAFHIATGGNYTAAELGAVYTLTTLHGMPLM
MGRNVNGSPLIEGTASIVSPDVIHAVNATVHVIDDVLVPENLVRNIWEVCFGVPASSNTPVPPASTI*
>CHBRA741g00080
MERGDGGKRLAALILGLACLVGVARVDAQGVAPPPPPPPSTPVGTDIVSLICATPGLSAFCNALATSRVSLELLQ
NVAVQRVTVFAANDAAFGNVRADIKACFGKEPIDVLSQVVAFHIATGGNYTAAELGAVYTLTTLHGMPLMMG
RNVNGSPLIEGTASIVSPDVIHAVNATVHIIDDVLVPENLVRNIWEECFGVPASSNTPVPPASTI*
>CHBRA741g00070
MERGDGGKRFAAFILGLVCLVGVARVDAQGVAPPPPPPSSTPVGTDIVSLICATPGLSAFCNALATSRVSLELLQN
VAVQRVTVFAANDAAFGNVRADIKACFDKEPIDVLSQVVAFHIATGGNYTAAELGAVYTLTTLHGMPLMMGR
NVNGSPLIEGTASIVSPDVIHAVNATVHVIDDVLVPENLVRNIWEVCFGVPASSNTPVPPASTI*
>CHBRA617g00160
MSMAVGDPWSSGGGVSGGRKCGRSSMIGEVAVIVMRIMVMAVTALSVISAVMGQPSPSAPGHPGQVQPLP
DVIERSTQGKNLTLYVGALRSSRGALMAMEAQRRRPGGVTIFAPTDAAVSDQIQRLGGCLAAIPDVADLVMTSS
LLYSAVPGHLPMAEIRSRLNQSNGSLRLPTLYPPNEVAVSSATSRYPSTKMGVFVDGAQIFPSGGFDVVTAPDVV
VQMVDTLLIPRDTTELISEFCGEATISVPPPISPGTP*
>CHBRA129g00470
MSKGSKLRSVSSPSSSIFLFSSSSSFFFLSFLLCCSILAVVGETPSSSPSPSPRKSGNAVRGSSSAPASGKSPPRTRAA
SSSSSPPPPSPASPTQRREPSSSIMAALKEAPQYSTLVSLITKANLTVSLDTTSPLTVFAPTNDVIEALNVGDTKFLT
DPKNVELLKRLVFYHFVNSKILAVNLTAGRSLRSLEGENLIVEVEDGTTFVQGAGITAADDIIREDGVVHGIDSILF
PLSVENAMPPPGEFTDDTADPSDQSPSSSSSSSSSAWQEGAPSSDVMEDYSGPPDEKFLGSSSSPSSSAPSYSG
ELVAALKAHPDYSLIGQLLASAGPNNLAALDSLENDTLFAPSNQGMSSSVEMAQLLTNATARNDILLYHLLAGRY
TFSWLKEHPGVYDNFTGFEIVVSGGGTEVFVGNAASLGQIVTPDLYANPKIVVHGINKVLLPPEYAAVPPASASL
PLSLSAADEVVAVSSHSEAAAPSLAAASGSWILEEYPADDEGSLGSLAPSSLAEDSLAPMSDVGAPLLASSPSAV
DPSSSSMQMTAAASSDVADATAGFVSALARNSNYTQIAFLVGQVSPTDLGELTNNTLFAPSNEAITNFMLAYAA
LLSDNAKLDEVLKFHVLNGSYSALFLRTNPGDYGGNFSVVVQGDSLRVGYSLALGSVVAPDLYATSSITIHGVDTV
LLPPEYADIDVSDYDAAPSAPLASTFSTSEEDMLGSSSSPPSSSSMEMTAATDAPLESSSSSSSDGAGALSSGAAD
VFISALGRNSNYTQIAFLVRRVGPTYLGELTNNTLFAPSNEAITNFMVANAALLGDRAKLDEVLKFHVLNGSYTAS
FLRKNPGDYGGNFSVDVQWDNSLMVGYSSARGLVVAPDLYATSKITIHGVDRVLLPPKYANGSADGHVPVSAA

AGPTVPVPATNGASSAAGANGQTPLTNSSSLPLGKTAARPSESPENSAAVVSSLSLGAPLVGFMIHFLLFFLK*
>CHBRA513g00210
MAAAMSAAMAALFTLLLVVSPVLIHGDAGGDLMKAIKADQRLHHLHQALITTGFSKELIKAVKDKPVTIFAPLD
DAVDAATAAGWVSWDCLTSTKAGKAALKENLKHQVVNGSFYTVWKLAKTNQVIPADGFPIEVTFDKATHLVSL
DGYVHVAQPEAIIMKNAIVHLTDGVIATESNYANMLDVCGEAPPSAPPSAVFGSGL*
>CHBRA510g00060
MAFLRSSHKWKASVVVSVFLLVFCFLSSHCGLTSAQAQPFLSSPSPPVPTTSSRPAINVSTIDPSFFQYPATMSYLL
AKPELYRTFTRLLRIPKDKALPGSYAVQIVEAAKLNITLLVPVEAAWTALGNAVSCLESTGAYSLTDNILRNHIIGM
HNATEMALNPNSYPTAGDTPVSIVSVPTGITANGANVVVPDKFMTFGGVVLNLDRVILPLDFGAQWQAACPTI
PSVSPPPAVESVPAIAAPASGAAVPAPVGPELTPPVAANPVNQSVGVPSTDAGVPPTDVGASSAAVVDSNETQ
APGSGARDQSSSGSSLNKDGLFSFALELLVTAALVWTAYAS*
>CHBRA04g01220
MAGTTEALRLAIIAMLTCLLIWSSPGGCGAIASATAQGEDSQNVTEINAGVLLEGKLAEQPDLTMFVQALLVSDT
FHVAISRANGGGVTVLAPNNTAFSVFDPAVLDCLYSKPASLNVLSQITKYHILVGNHNATELIAMQTVHADSGLP
LHFNLTSRGRVRISDDDGGEAFMTRPDFRLLINSTIHVIDTVMIPPVIESVFATECFAYANSNDAPSSSDAPPSFHL
*
>CHBRA476g00010
MIMRSCQGKWRVTVLGGIDGGRDRYGGRDGYGGRERYGARETVFFFFLGFLATVLAMSVDIADAVPSARLLR
DEAIGGEEDWDVLGSASVRLVNGYLGRDTARESSSSGTLPFLHFDDVEYFLWGDHRGRLLEAVASSAVASGFHS
AAAFGSESNDGGGVGSQPSAPADPSLARTGGGERARMPPPSVPGGASVEQSAPATAHSSMPSHGGPVSQAP
ASGSHSAVSDGTISQGSLRPSASLSSQSPSLVEGWDVADGSVASSTNAVKSLASMSSALASSSGSIERADDTLGH
GSEATVEAHAASASSTSAGSSSSAPAVPEFLEVIQDDTDFSILNEANNEAAARLHRSLSVGLDYVNTLRNVTFLAP
VNSAWDFLDPAMKDCLMAGDKLVDVVVKSHTLIGKLTAEMISNVPRVENLYRFWLNVTTDDITNLTSVNNVEII
RPDVYMFPTKVVHGFSRIMLPSNWKSFCVGDAGTGSTSVTAPPPSSESPLASSHSPPPPLSSSAAPPSSSSQEG
VQESDASTSPSLSPSLPLSSPPSSSASVIPPPPSVPPVDQKFVGFIESETSLSNLTRGSEASAAAAESRAPVHPGAP
KSPIEKLSRLRNVTFLAPLDEAWHLLNPNITDCLTAGRYLLDWIIQSQTIRGKYAATAFTAGQVVYNLFGDPLHVS
KIAESNGILRTFVNGLELVKENMYQGSDRVIHGLSGLILPKKWQKYCTPTPPSPSTPGSQGGAQTPPFSSSPPPR
DEDESVSVCCARTPPQPDEGYSTVCVLPAPLFPPRPSSSSSAPSKPSISTPMSSSSKLPVPVSSSPTMRTEDKGSSS
TPSVSESEPPVSDKSPDNSPPIEASASEPCDVRRGRGCVPGAHTASNGSVLITSSPTEGGEEGTDNGTIVTGTSG
DTGEVDGAEDQGNNRMATFNVISGCSSVTQMIRTGCVAIPVLTSLLTTVLVFSLSLSLSDFIW*
>CHBRA312g00020
MDVEPPGRVERDGRAQVPGDYRLEEERVREMVRACFEEGILPTNIDPGEMEVSGREVKFTLNASLDEIKIRWL
KERTVMVIFRDGARFLPKKVKEDVVRAYEDVWIRDDVFEEEFKRGRIKVESPNAVSYVPRAQEITDWMLQKKS
DFIDLACTTYRTEFKPWLARAEIRDWRQLVDQNAFWVVAVGIPLDEMPSIHVHIEKAIGKVIKQHKPEADESDP
KLVNLRFDVDPACKSNMKDKFSIQTCQGDILEVKLVGPELEWCKRCRSFFYTEDNCRRPGRGAQGGINNNQPQ
ASTTGSRQPRPSYKGTRQAPGAASQPTVQPTITPLNVKQHPVIRMNPTFSPSGRQVQQHGVPLQGTGQGQA
SSVEAAWAAYFRSFPPPLGAGLQPGMGPMQYQMGMSPSVLNLHAFSLWVASVPLQEGTLGNEEAMQMQQ
LVSPAGMSLPVASGSRVRGKSPRKQRRVSEGDVDIQVDIQTKNSGSSTDSQAAPSTAGKRKSTRLRMNALSKR
QAVVESRAQLCVYAATYYQYILTTRRPGEDLTENLSEGSSHWDQYVVRLSPQGKLDMDRLITIEEAWEASKAVA
KAGILSHPLLLFRVGPSETMKTAMKLVALIVALSFFMSPRASMAKAFMSAAPSAAAPSAASGAVPSAAMAKTG
KDAKVFEILKNESHFSMFYKALVDTEIWVEGRTVLVGEQLILFAPTNAAFSTALSEEVMTCLQNEPGQATLASFLK
YLQVVVPSNATAPQVKTPKQLKVTKDGFLWTVGGKGIAVTKDVDGSIRLTRDDGVDSVTVQEARPTDPTTLIPL
EDAVVVDEDVGNTILSMCFGEEPSQSSTAAPSSGSLPAPELSSEDSDAASSESATHVDSFRRLWELLF*
>CHBRA246g00180

MAAAGSVTFTSLAFLLLLSPLFVSAQVDTSAGDDLVGAIINHCCLKKFYYALQISRIELRLRDSANRGPITVFAPTD
DSITKLDPELWTCVTTGQGPLDILSQITLYHFVTDGNFTAAEVATKRHLTSASGMPLGVKVVNGNVMLEDYAPIT
EPNALRTSNATVHLIGELLVPETIVSTIENLCFAGPASAPAPSK*
>CHBRA246g00140
MSQDRGKGPAPADPSTGPRQGHNTKAFKSPFNVDAKVYGKRDDPVWHFVARGRYLDGSTAAGRRLGRRCLC
RLCGRSICGGAKAAREHFSKIEKFRCEEATPAVWYAIGAKDMTKYPKEFVSAIESLQSLDSGHPRFQWPPLRYLE
DSVPPPVSTTAPSCASPLPGLAAPTDGVTAPLPAKAGPPPRPHPTVAGTSGARHEHVGRSLPANEPPSVREGGG
VAAFDEKSTRAFVDSRRWGRDAGSSAGLATVFTAARQSPAARATSAVAPAAPARRSSEHPWLPPPPSRAARDH
VVGHVGVMDRPDPTHVASDARHPAPPSRVGGQAVPGVPDEEVLPPLADSAPTPIRTTTATAPPVLSGSLDPLY
CMRDIAVAQSAAPASPGGLLDAGVLGGQTTTGRGRGTYRQQAVTSYYSDPLERAWHLHIMRFLVESGMPFNC
AKLESFKRMFTMIIPPGIPGAPTPKLPTYHMLRTTLLDKLDAEVEKCVRPVLDMSREHGCTIMTDGWTNIRGQT
LCNYLVGTETGVAYVATDVMRGKKDAHALATAWLNRVKSLDVNLSDITAFVTDSAGVNVAAMDVFQKDESIKH
IFWIPLVTRFFKRHHAREILKGHSKKTLLLLTQTRFGTNVIMMERLVDVRGELTKVIGQKRWRETVWSTNKIRKD
ATEVTACIGSPSWWEDLIALYNMLKPIMDMLRLVDSDTRLISKILRRYEVMIASCLSACKDIDRDQQDAILEVFAR
RRTMFRTPAHRAAMLDPEFRDPTLIDDDEVQQALVEALVQFGYPEGSPQHREVRRANGKFHTGEPPFDDVT
MRRAMDTFDHPASFLSSKSAKFPHTAVFAGRIIRIWVTGSPCERAWSRWSFIHSKSRNRLEVSRAEKLVWCHW
NLRLLDRPELHEDGTGERPDIFGKWLHYWHAVEDEVPVDHQLVRGSGAQVMVTEEDLTHARERLCAVSCMG
VERRVAKSDRRRRRSEGRGRAVAEGATRGDVRAGARTRRRRADDFVRKARVRWDEGDFLFDSTSSNDDDFFA
GGGGGGEGGGGGGGGSDSGEGGGGGGGGGEGRGVDGDVGHGGHTMTRMPAIVLKRLRRGGRRDDSIAS
RVRRRPRVHVATRTEAHVMQQDPVVMSEDDMGDHPASLAREESGAVVTRPRSPEPAVGTAKDTQLHRMPST
DVVMGEDGAPRAEVASAGVGLQDGEAPPPPTTGGGLEDGEVAHTPACAQDGLESLRYSREEVAHVLASCGYR
AEDIRDTLAGYPDRLQQGVGLHCPPDSLPRTDELLAIDAALAGLPEILILISLSLPDVAPPKAAPLDETHHDTGSDA
VGGDKRLLPSEVRSSPAGFHVGGPWTVEQGLADEVARNRHGGPRVAAQRSLGESLEAASTDHVAPGGRGSQ
DFSDGGSLVPARAAGQQAEPHLSPTAATEADQSRQMSAAADGQAGADGGDGVDGPGRGEKRRGGWSIIHD
DNSTAAEASETTGADDPNDSDYVPRFRMADGDDGGGRRVSAMAAAVSVATATLAILLLLAPFSVAAEVDTSAG
DALISAITNHCCLKKFYYALQISRVESRLRDSVNQGPITVFAPTDDSFMKLDPELWRCATTGQGPLDVLSQIMLY
HFVTSGNFTAAEVATKTQLTSASGMPIDVKDVNGNVVLEGYASITGPDALKSPNATVHLIGELLVPETIVSTIENV
CRPGPVSASLPSPGL*
>CHBRA246g00120
MAAAVSVATATLAILLLLAPFYVAAEVDTSAGDALISAITNHCCLKKFYYALQISRVESRLRDSVNQGPIVFAPTDD
SFMKLDPELWRCATTGQGPLDVLSQIMLYHFVTSGNFTAAEVATKTQLTSASGMPIDVKDVNGNVVLEGYAAIT
GPDALTSPNATVHLIGELLVPETIVSTIQNVCRPGPASASLPSPGL*

Liverworts

Marchantia polymorpha

>Mapoly0101s0015.1
MKVLSACLLTLLVLGIVQQSDAQACTTTIYKSLTGQLSSLKMVVDAAGLADTFDDSALNVTVFAPNNAAIQ
GLVNVLNASGLTLANVTGNNNKAASIVLYHVATLVATSTQLVNDQSLPTLYGGYNLTVDRNATNVEIDGGA
DTQATVVTPDVRVCGSVVHIVNAVLLPQELNDITNYVPPVPPAPGTPPTPPGTVSPPPPPPPGGAATRVG
SSVVMGGFAAIAVVLALV
>Mapoly0097s0014.1
MAALSGLALVLCLCLCLGAAPPGASPAFAAAAGASANADVVGNAGSGSVLQALLESQYSEWMELVEQAA
MLHPLEELVGSGRKLTIFAPQNHQLQPKIKSFLLKPSNAIALKRVVQYHVLSERVTASEWPTASFTTLASEAV
WLSTQGPILMAGDVAVAAPDAIIRPDGVVHGVEEMLIPNSVMQEYVAHTTRKMATPLPTGAPGLDTRR
AQLLEKQFSAVEAKLVVAAASPSLPPALALAPSIAPAPGPAMGPSTAPWLGFGDDEVTTFISAMLRFGGYS

EFAGLLVDLTSLGSEISKLVNMGYKLTILAPDDKAWSGALTEEHLSSQTALEDILHYHIITEYQTEESLYSTLRR
MGKTHFSTLRVPHKLAAHEVDGQVVFGEGDDAAGVFDHDIFADGRLSVQGIDRVMIPAELKATLPSDTT
VSPPAPKVVKAKARNLVEQTLQLPGSSSATVNAGPGSSANGATVCLALAAGAAVLVNLLAL
>Mapoly0095s0061.1
MGVRSPGVVVVRTMTIKRPSPKQFRAGRDGVQLGIYLVAFVLVGLVKIDAVEAQVVPSPSPLRAPAPSAIP
PPPPLTALQQSQAAVQALRANNFTTAAQLIQTYLPILRLNSTLFVPTNEALSSLSMVTPVLNILLYHAATPQY
TFDQLVTLPVGTRLQSFLANESVLITNNARNNFQVDNVRIVLANVCASNTTDLQISCHGVESILNSTLYGNS
APAPVSSSPVAAPVPAFTPAASPPVPFILPGPPAVPSGSPESSIPIAASPTESSPTGGASACRVRRILYIVFLGV
MTLSYNL
>Mapoly0093s0009.1
MGRGNAGLSLLTIVLLGLYVASTTAQTTPALNITSILAKYPRFSVLSDLLVSSGVEKEINSRTSITLLAPADAVLT
AFQASVPNADTVKIADLLRYHVLLQYFDMTELKGLGTVNYSSVTTLLQTTGRANEQDGFVNIYNTATQILIG
PAASASASNATVVGTVLEDPYDISIIEIDQVLKPAGFDAVEGDLVTVLESFQTFSLFISYLKATGVDGVLATRQ
TSGGLTVFAPRDSAFNALKSGSIQTLSPGKQKLLMQYHALTQYYTMTNFLSMVDAPVQTVSSTVGGGYL
MNITSKASTVTIKTGVGNATVGEVLYNANPVTMYAIDKVLLPTELFGTPDQLSPAEAPTPTPDAAPAPTPKA
SAPVPPAAETPTILLEPEVPPASTNPNAASVAGVSGALSAAVLIVSSIAAFL
>Mapoly0075s0013.2
MARSAIALLLVLVLASATQLNAQAACNTSIFDAVAATPDLSLLGQVVQAAGLTETLSDPTLEVTVFAPNNAA
FEELLQVLNSSGLTLDDVTAPESNKAASILLYHVVPVAALSTQLSDMQVLPTLLGKNLTVSLMGGMVNIIAQ
ESNATVVTADVTVCGSVVHIINHVLIPAALADIPNYMPTTPTTPTTPPAAMTPPAAGSPPGTPPMLASPPP
PDCKY
>Mapoly0075s0013.1
MARSAIALLLVLVLASATQLNAQAACNTSIFDAVAATPDLSLLGQVVQAAGLTETLSDPTLEVTVFAPNNAA
FEELLQVLNSSGLTLDDVTAPESNKAASILLYHVVPVAALSTQLSDMQVLPTLLGKNLTVSLMGGMVNIIAQ
ESNATVVTADVTVCGSVVHIINHVLIPAALADIPNYMPTTPTTPTTPPAAMTPPAAGSPPGTPPMLASPPP
PGPDNGAMKASSSVAMGVVAAALGIFVALA
>Mapoly0033s0033.1
MVGMASYRAAPGIYCALLLCLDFLMVSSQSPTVAPSPTTTPSAPVTAPPSPAALPPPAIVPAPAPAAKPQP
QVVDMIEALRDAGQFGAIAGLLDGLQMKNLTPMTTWLLPNDEAFSGTSYPKNVTKFIDYHVIRQLLPYSR
LSTLSVGTRLPTFLGSETVVVTSNLRFNYSLDNAMIVVPDLYSDSTVAVHGINSVLNDYLFNEGVSAPSPGE
SPSGVPTAGPPSPPSSSSSPTTPGESNPITLPAAAPTLSTSTSLPVSFGLILIVAMLIV
>Mapoly0027s0087.1
MGPRRAARSGLMALALLCCIVGTCSQSTHKFNITKMLSGHPRFSVFREMLSSHGVAAEINGRKSVTLLAP
ANNVMRAFRTSNNKTDAIKITDLLRFHVLLTYFDMAELRALKTTNYTSVTTLLQTTGRASGNNGFVNIYNL
KSRVKLGPSVPDSSSNVTVLAGVQKSPFDVSIIEIDQVLLPVGFNNASGDLIAVLDSFQEFTLLISYLQETKVD
AVFAEKQAEGITIFAPRDSAFNSLATGSIQTLSLSEQKLLLEYHALDGYESLDSLETMLNKPTKTLASADAAGY
ILNVSATPGQSTVRLRTGVSVATVLEIIYDANPVTMYAIDEVLLPLNLFTPAPLASPAPAPAPKKSAVKSPPAH
KVPATVTPDSASLAERSLLVTMAALISSTLAVIL
>Mapoly0027s0084.1
MASGRTALVASVLLSCIIVSSAFNVTTMLDKFPSFSEMNKLLSSSGVAEEINSRKSLTLLALSNDVLTAFTASN
PNVDAVKLADLLRYHVLLQFLGMDDLKALPTDNYTAVTTLYQTTGRANDNDGFVNIYNLPSGILVGPSVAG
SSSNATVATNITNEAFDISIIQINSILTPVGFNTATGDLIKVLETFKDYTMFISLLKDSGVDSIFAGRQTGGGITV
FAPTDSAFNGLTSASLQALSVTDLKLLMQYHALTSYQPLDILQNMVDSPVSTIASTIASGYLLNVSSAVMTV
TLHTGVNDAKIIETLYDARPVTMFGIDAVLLPAELFSKDALVPTPAEAPVPEASAPDAAPEAAPGPEKPAGS

PASVVKMSVALSAVAMIASSLVAAL
>Mapoly0027s0081.1
MASGRTALVASVLLSCIIVSSAFNVTTMLDKFPSFSEMNKLLSSSGVAEEINSRKSLTLLALSNDVLTAFTASN
PNVDAVKLADLLRYHVLLQFLGMDDLKALPTDNYTAVTTLYQTTGRANDNGFVNIYNLPSGILVGPSVAG
SSSNATVATNITNEAFDISIIQINSILTPVGFNTATGDLIKVLETFKDYTMFISLLKDSGVDSIFAGRQTGGGITV
FAPTDSAFNGLTSASLQALSVTDLKLLMQYHALTSYQPLDILQNMVDSPVSTIASTIASGYLLNVSSAVMTV
TLHTGVNDAKIIETLYDARPVTMFGIDAVLLPAELFSKDALVPTPAEAPVPEASAPDAAPEAAPGPEKPAGS
PASVVKMSVALSAVAMIASSLVAAL
>Mapoly0027s0080.1
MASGRTALVASVLLSCIVVSSAFNITTMLNQFPSFSQMNQLLSSSGVANDINARKSITVLVVSNDVLNAFTA
SNPNVDTVKMADLLRYHVLLQFLGMDELKALPTSNYTSVTTLYQTTGRANNNDGFLNIYNLPSGFLIGPSV
AGSSSNATVAANITHEAFEISVIQISSILTPVGFNSATGDLIQVLTKFNVYTMFISLLKESAVDSIFAGRQTGGG
ITVFAPTDSAFNSLASGSIQGLSVSDLKLLMQYHALTSYQPLSVLQNMVNSPVSTIASTIASGYLLNVSSAAK
TVTLHTGVSVATIQETLYDATPTTMFGIDQVLLPSELFSKAALVPSPAEAPVPEAAPGPKEAKAPGAAPDAA
PGPEKPAGSTASVVSMSVVVSAVALIASSCLAVL
>Mapoly0027s0079.1
MASGRTALVASVLLSCIVVSSAFNITTMLNQFPSFSQMNQLLSSSGVANDINARKSITVLVVSNDVLNAFTA
SNPNVDTVKMADLLRYHVLLQFLGMDELKALPTSNYTSVTTLYQTTGRANNNDGFLNIYNLPSGFLIGPSV
AGSSSNATVAANITHEAFEISVIQISSILTPVGFNSATGDLIQVLTKFNVYTMFISLLKESAVDSIFAGRQTGGG
ITVFAPTDSAFNSLASGSIQGLSVSDLKLLMQYHALTSYQPLSVLQNMVNSPVSTIASTIASGYLLNVSSAAK
TVTLHTGVSVATIQETLYDATPTTMFGIDQVLLPSELFSKAALVPSPAEAPVPEAAPGPKEAKAPGAAPDAA
PGPEKPAGSTASVVSMSVVLSAVALIASSCLAAL
>Mapoly0012s0106.1
MGRTRAGSVVAVLLLSWMAVASAFNITLMLNARPSLSAMNQLLTSSGVAAEINSRTSLTLCAVSNPVLQA
FVATVPNVDSGEVADLLRYHVFLQYLDIPELKQIVPGSPSTVTTLLQTTGRTAGNDGSVNIYYDGTAIQVGL
PDITAPPNATILSSIASIPYNISIVQCDKVLVPPGFGTRATDNANITAALEAGLNYNTFIQLIQSTGIDAEFAAK
QTGTGITIFAPTDAAFAALPAGALAALTPDQAKQVLRAHAIVTYYPLGTLSTMNKPNEPTLATTTGGPGTYT
VNVSTSTTGGVTLSTSVSSSRIGATLFDTNPTAIFSIDNVILPAEIFATAPVPSPAVAPAPLTPGLAPAPLSPVPA
PAPGTPVPAPAPAPLTPVPAPVPTPSSTPAPAPAPMTPTPAPVPAPDMASPPAPPEEIPIAETPAETPADTPS
SASSIDMSRVVLAAVTLFSGLMLL
>Mapoly0194s0008.1
MGRQKTVVPSLVGSLLLNFTVLTWAATFNITSIFDEFPDFSIFNQLLTDTRVAGEINRRQGVTVLAPSNEAM
TDFRSANPALEGDRVANLLRYHVLLDFLTIQNLQRLDIANYTRVGTLFRTTGPANADDASVNLYNTPSNIFI
GSSAVDSSQNSTVISTVFLQPSDVSVIMIDRTLQPVGFETPRGAANITGTLQDLGGYSIFVSLLTQTGVDVVF
GGRQSGEGITIFVPTDEAFNKLPGQWFEAFELKEQKLLLEYHALTRYNSLDALWRYRDKQVPTVSSTVQEG
PDAFNLLVTANKGLVTIHSSTPDKPVATLQSTLFDASPLVLYSIDEVLLPVELFGDVMAVPLTPPAPPPAPACA
AAPAPAPSPAPPPPVKPKPKRPVPPAPPSPPIASSAGADSPLGSLPLALLLTAVLRFSLRL
Mosses
Physcomitrella patens
>Phpat.016G034100
MGSRGMAAVGYLGEGGGGIAAAAMDWRWRKIGVLSMLMLVWCGGVEGFDAGVGSNKVIGLREVRL
GTGSVLQALLDSQYSEMVLYLDKADMLEELEREVLRQKAITLFAPKNSHLEQLDADLGRFLMRPGHEEYLR
TVLRYHVIPGRVEGADFQNRTVETLSKGDVVGLRTYGLKRYVGLLRVFSPNSIVRKDGIVHGVDGLMVPPK
VASAFEEWKRNGKSGVNPHGSPVTLKRSRKSSAGAKSAPKARYMLERSAPAVPAALIHAVTAPAPPATAPS

LGPSLGPSIAPAPGPGTAMFNWDDEDEMLQFVTALSNYGGYNDMAELLVNATTLGVELGKLARMGYKLT
ILAPNDQAMQLLTTEQLNQAMEPLLYYHFLSEYQTEESMYNAVKRLGKQSYSTLRHPHKVVASESDGTVK
FGDGDDAAHIFDHDIYVDGHISIQGINRVLTPPP
>Phpat.025G021800
MVVSWGLGVEGFDPLESKRVIGLREVRLGTGSVLQALLDSQYSEMVLYLDKADMLEELEREVLRRGAITVF
APKNSYLEQQVDADLWRFLMRPGHEAYLRTLLKHHVIPGRVEGADFQNRTVETLASGNVVGLRSHGLKR
YVESARVFSPNSIVRKDGIVHGLDGFMIPPWNEKPRGSPVSRKVSRKSRSGTKSASTTRYMLEKNAPVVP
DALLRAVMPPTTSVTAPSLGPSLGPSIAPAPGPGIAGFTWDDDEETLQFVMALTNYGGYNDMAELLVNAT
TLGIEIGKLARMGYKITILAPNDQAMQQLTIEQLDMPMEPLLYYHFLSEYQTDESMYNAVKRLGKQSYSTL
RHPHKVIASESDGTVKFGDGDGAAHICDHDIYVEGHISVQGINRVLSPPP
>Phpat.001G016800
MAGVNLFPSLRGAQLVFNTMVCFTLLLPLALGTVASESLVTAAASKVPAVPVPAPVRAPAPAPKILTLHEKV
VTSLRSAGHYGAIAGLLDSLPNNNIIKTGMTLLAPNDNAFSNVLMNSTTYLTTLLTYHGAAKVYSYEGLLNL
PVGTKIPSTAANVVIVVTSNSKGAYKLDDSQIVDPDIFVDNTVAVHGIDNVLNTAKYNKGVVAPEAAPAPV
DASPIAPARAGPPAPPGSIVNPGGSGGDATDGPSYSPDSTPQLINPNGASVVPKSSLLDVSLIALWLFLIPVL
AW
>Phpat.013G027300
MAGSHFFRPGANAWVLLASIVCCALLVSAELADDSPTKAAIAAAPAPLIGAPAPAPVAPPPKPLTLHEKVVA
ALRAAGHYGAISGLLDSLGEASSIIKEGVTLFAPDDGAFSGLNLNSSKLLMTTLDYHVATSVYNYNQLSTLPL
NSTIKTSVPDVVILITSTGTSGLRLDNVAISDPDLYVDSQIAVHGISAVMDTAKYNKGVVPPEAAPAPLATNP
SPAPGLITTPTTPRTASPAGTGNGAAHVLTNIVSTMLLVSAVSFLIALL
>Phpat.003G016800
MADSRCAWMILASTLVCGLLFPAVLAKDSPSLAAAIAAAPAPPVGAPAPAPVAPPPRPLTLHEKVVAALRA
AGHYGAISGLLDSVGATSNVIREGVTLFAPDDGAFSGLNMNSSSLLMTTLDYHVATSVYNFNQLSNLPLNS
TIKTSAPNVEIFVTSTGTDGLRLDNVAISDPDLYADGQMAVQGISSVMDTAKYNKGVVPPEAAPAPVAGD
PAPAPGLITTPTTPKTATPAGLTLAKRQGFVKYLNFTSTLFTQKGRDLAVYRNDANQGVINGSMKKLGD
>Phpat.026G042300
MSARLISLRAGVWLVVASTLFHTLVASAAAADDAPSLAEAILAAAAPSPFIGAPAPAPLAPPPKPLTLRDRV
VAAIRAAGHYGAISGLLDSLSETSVIKEGITFFAPDDGAFSGLNMNNSMLFMNTLHYHVATAVYSYQQLSY
LPLNSTIQTAAPNVVMYITSTGEDGLMLDNVVISDPDLYLDDKVAVHGISMVMDTAKYNKGVVPPEAAPA
AVAGAPSASPGLTTTPTTPGTTSPAAVGNDAAHVVSSAANAAILVAAVLFLSLLFQI
>Phpat.021G037400
MVAMASSAALRSYVLALLVVMAVVPHISAQQCNTNLLEAVRAQPSLSILADVIEASGLAEQLSMTDNVTV
LAPDNNAFNGTGGLLSILAANNLTLEQVTAPGSNRAASILLYHIVPSPATAADLTDGQTLTTALGKAYELTVD
KTATPTVVVSFIGAGSNATVITADLRVCNSVVHIVNTVLLPATTLTAIPIYNLEPSPSPVGSPGMSPAPTGNG
VVPGSPTAPTASVPGSPPESGGADSSFTAGSAVVALSAAVALAMIM
>Phpat.019G005200
MEFCLFRLVFAPQISNCSCFDLIQECDTDLSCALFPRLSRLTMALSSVSRCLVLVLVMLAVVPQAYAQNCNT
TLLAAVKADPRLQQLAAVIEAAGLADTVERLVNVTVIAPDNNAFNGTNGLLSLLRQNNLNLTDVTAPGQN
RAASILLYHIIAGPARAADLKNNQVLTTFLGPNYTLRVNKQTAPTLIVSFVGTGSNATVTAADVPVCSSIVHV
VNNVLLPATNLTAIPAYTAPGAAPGSPPGSMVPTPPGSQAPSGSGASSLSVGSMAFALSVAVAAVLMN
>Phpat.007G002900
MHRRRLFQSSSLRPPDCALLLQQCPPSCALSQQATRLTSFSTLPTSSESSLKSSLPSAHRFSVGDWRYQELT
MKTQAVALVGLLLLGTLSITTAQNISLVLARYPEYSTFKSLMESTAVLSEVQTRSSLTILCPPNSVLDPFIAARR

SFSTQMLADVIRYHVLLQYLDSAELRTQTNNGGMFTTLYQTTGRANELDGFVNITVTPSNAVAAGPSTAG
TPPQATILENITQFPYNYSFFRVDAVMTPLGLAAAQLAPTTPPTVAPVPAPLVAPVVAPVVAPVVAPVTAPA
SVPAVAPSAPVLAPVVPSPMAPPTASPEPSLVTPTPAPSTTPVVSPAASPPAPPTNGPSADETPAVAEPFTS
PINANSAVSVNIDFVAALASLFVAALLS
>Phpat.006G011100
MKCVGGTWHRGNTVDCRRLHLTHLRHAILCIKLIFYSYAATRATAQGLAQESVDSILQSLRAAGLTSSADAI
QAAVISGSITLPSSNFTIFAPVNQAVDSILPSNPDLSKVLNYHLATTDLPHKALLLLPIGQRIPTLLPDNTLLVT
DNNLANYSINNEQILYFDLCTVYTVSCHAVSGILNSTIWGQASLIGPATSPSAAPAGAPFIAPFAAPIGAPYTS
PAGAPYAPPPESALPPVIGIPPTVPGAPETPPGIIPPTVPGAPETPPGNIPRTAGSPPPPGQDRAAAATALLIS
VFFI
>Phpat.004G062900
MGSSKMLVGLLALIALAVEVTSQDCTTALNEFPQFSMQKDALISSGVADALKGMNTLTLLLINNGAFGGYL
GGHSTYTPQMVSDVLKYHILLSYFDTETIKTVSTMNDGVVTTLYQSTGRANGMDGFVNITVSPTDGVVTI
KPSIPGSTTQATVVSYVKAVPYNCSYIEISNVLEPIGLAAAQLAPTSPPSPAPVAVPAPATVAPTTAPISTPTTA
PASAPVESPTAVPASAPVDAPASAPSNAPVDSPVSSPPAPVEAPAETPVETPSETPVSTPPAASIEGPAMSP
QSPDSSAYSLRLNLATLLVSAFLGALFL
>Phpat.003G041000
MMAYQRGKKEVSALVLLLSCMSACTLAIALNEEDSRGIGAGKFNPNHAPVITGSSHLKSELHQDVVRVLRTVQT
YSAFAALLDNMTESVIRQGITIFAPNDGALSDFHKRKTQEHLENLVRFHIITTPLPFSNLLRMEAGSRLKTAVSNFT
ILVTNTTKDAYQVDDATIIDPDLYTGATIAVHGINAVFNTTKIGEGPLPENPKLGNEAPSETTPGGIVVSGSNSGSG
GKRSKYGSAFRIPINVEGVPFHDYLMAFVVMLLLQYFTVAI

Lycophytes

Selaginella moellendorffii

>15418660
MGGRGSSRVTLTALLFSALFLARLEAQVPIPGIGGGGGGGIPIGGGGIPIGSGGIPVGSLPTIPPAIAQSIIDTL
RTGGFSLAGWAIKALSNFIPAYSTLMIPTNEAIAAFVNLNQSEIPSLLLYHAVTGVLPYDVLSEFPVGQRLPTL
LFGNQLVVTDNSAAGYRLNTARIVRPNMCGNSTSVVTCHGIDRVLNPSFSLASANGPASGPSGAFAPANA
PGGMPLQRPPPAFVIPGPAIAPGAPPVTVNVAPAGANETGGAASSTARCGPPAPAACVALLLSALYCSFVSI
R
>15409766
MGVADAPSPGNVPVLGDMKNRSAAEMKISVLALRALTLVLLVIALALMVSNKQTQSIPIKLPGMASTIFLK
KTATFSQITGVQYYVGALSVAVAYMFFQMLAGLFTILTTGSIVGSKSRAWVTFILDQLIAYLMVSAATVVAEV
GYIARRGETKVGWNQVCSDFKHYCFIYGFSLVNAFLATIAFLPVVAVSAFHLFRMYGAQSAQSKKYGALHA
CMRRTGRSYKPLLSQLKDHHIPVHPSSRAERAMESRTLLVLLFVGVVTIVSSGLERAAAQDDTDDGILPSSD
VQPLVSNMIGQGFTVAAAVAQSLQTLIPIRSTLLIPSNNAIAGVDANLSQEDIINTLQYHVLTFPTSFEALSR
NDVGAELPTMLQGEMITVTSNSPGNFTLNEVNITHPDVCSSTRFIACHGIDRVLAYNSSLVTAAGPEASPP
FGAEQASPAPEALPPGTRSPNNTANPSNRKSNSTSGVSLRHSFTSGVAFIEVLAASWLLM
>15417822
SSSSGDSLPKISSKGSSSSSVLAALMESQYTELMELMGHAGMLEPLEKVVGKGSITIFAPRNAYMEQQVDS
EFKHFLLEPGNIKHLRQVLKHHIVPGKIMSEDWSNRTVRTLSTETVRIHSHRGINRVELSEVTSPNDINKKD
GVVHGVNGLLVPRSVQEAFSVKKLGSSANALLPQASPEFDPLGASLAEMSKEFQDFLEAAESPLPLEFAYAP
EVAPAPAPGPSSGKHRVYTWEDVDDFVSTLANFGGYNEMAELLVNLTSFAWDMAKLVNEGHRLTLLAPN
DHAMDHLTTEQLNAPGGLEAILMYHVLTEYQTEESLYNAVRRFEKVKFLTLWQPHTIHAKETDGTVQFGE
GESGALLYDHDIFTDGHISIQGISKVL

>15418372
NSTRVLAKFDHLSTFNRYLSETGVIKEVDSRSSITLFIPPNAVLDPVIASVGANNLLVIADILRYHCVLQYLEIKE
IRSMTNATGIVLATLYQTTGKPTGEYGFVNVSVSHGGLIHLGVPGAASNATVVKNVTQHPYDMSYMELDH
PLMPMDLGIDQGAAPSVNNLTAALKAKGNFNTFLGLLASTGVDKALRSMSAVTLLAPDDDAFKALPPNA
LAELSQAQKIALLQFHAIATYFTMGSLRSVATPLPTVASSRIGGFELNVSTAGGKGVSFVTGLNRADVTDTEL
DTPPVAVYAVNRVLLPPEIFALAPAGAPGPALSVAPSLAPELSPAPTPGPSSSPSPSPSPSPSPESAPSPSPVM
VPAASPPAPPSSETPSSSSSAPEEAA
>15419255
ILASTLAAHSTAQLTATTNPTSVLQNRPDFSTFSKLLTDTGVISEINQRSSLTLLVPHNSALDAVLNRSSPSSTP
LPLPVVADVLRYHCLLQYLDVPQIKSMTNELGMLVTSLYQTTGRAAGQNGFVNVSVSSDGQIHAGLPFAA
TSLATVRTNITQFPYNISYMQIDQALVPEDLGRPQAAASSILNLTNTLQRSGKFTTFLNLMQSTGFTAALATL
PAFSLFVPTDEAFQGLPNGTMAALSPSQASSLMAYHTLPAYTSSGSLQRQNSLVQTVASNGDNQKFLIQV
APSGGNSGGVSLSTGVDTADVVSTIYDQPPTAAYSVNRVLLPKEIFEHAAAPSPSPLVAPLPPPTQVPPSSSP
LPSPSPAPTEASFPPLPSSTPSPAP
>15417283
MSVEQQHRQASLALVCSLAAMAIIILPWASLCGAQNVTVVLENSAPVNTNFSTFSRLLSMTNVTAEINSRS
SLTILVPDNSILDSYVGANLEGMHVWAVADFCRYHVLLQYLDTQEIMQMTNQSGLLITTLYQTTGRAEGID
GFVNMTFGNDIQFGLPPPHASGPVATVLNNFVRYPYNMSFMMISKPLMPVRLAQMNRPDHFPSISNAL
FKGNIYKTFQKLMQDTGTLAAAEDIEHQPFTSGVTVFAPTDSAFQNLPSGSLAALTQSQRQLLVRYHLLPSF
FTFGSLRTLKAPLTTLATSNRNFEVNASGEGPSGGLAIATGVSTANVIATLLEDDPVGVYALDAVLLPPEIFPL
HSTPPASIAPSPAPPATVPPPKAVQAPAPAPKPAPPPSPSSSPSPPPSATSPPPVQSSSNSSSSSSSSSPAAE
NGTSAEAPVAAEGPLAERSDARKFGLGFTTRYWDLEHSLTPAHLSTNARVVLDHSKTVETSSEMPAVRGR
EEAGGARSAATGRIASGRLEGQLKVTREDEAGRGEDEANGDLRRARPWWDEASAAMPCYDFNRI
>15401851
AVAAILAIHNARSIGAAASVDHPPAPSSGAGNVALKSVLAALLESPATEMMELVEQAGMLEALELAADRH
NLTIFAPRDEFLELHFDADFRRFLLLPGNVRFLQELVMFHVLPIRITASQWRSGRFQSLSGSRDEVLLHWNK
KQRLAVDRSTVDWPDMIVRSDGVVHRIDGLLVPKSVQDAYAAARISSSAVLPQAAPSLSGESFGNKAEAK
LFHVTFPVIDDLIGSAPAPAPSPSRFSADGHGRVQDFIAALVSIGGYGEIADLLVNLTSLSVEIANLVNEGHAL
TVLAPGDRAVARLAAEHLGAIESILAYHIVAEYQTEESLYTLAKRHDRVVLSTLHEPPFNRLVAREIDGTVEFG
SGNDLASSNSSRSSTGLLMDPNIYTDGRISVQGINAVLIP
>15413899
MEQHKQAPLWALLLILASGLILTAVGLTKNLTLLLNNYPNFKRFNTLLTTSGVVDQVNPMLTATLFIPENNTL
LNDFIYDMGKHPSEEKLADLVRYHISDFYIESEFLAVRRNFSSPIKTLFRERPYEETLHFRWLQLNVDNHGVA
TLSRPPHQSPPLATILRNVVQEPFSITVYAIDRVLEPEGFLPPPPPPPPSPPPPSPPPPPPPPPSPPPPPPSPPP
PSPPPPPSPPPPSPPPPPSPPPPSPPPPPSPPPPSPPPPPSPPPPPPPSPPPPHPPPPPAFLPESEDGIPPPQS
LAMQRFHRSVLASTLVSLLAMLLLKF
>15404663
SINITQVLGLFPDYTNLNMLLLQTGVANEINQRSSLTILAAEDSVLDPILDQLAQSVTGQQIADILRYHVLLEY
EGINDLRSLPNKSKLFTTLFQTTGRASNNAGFVNITDDPNGGVSVGWPSSSTVFSSEILGTVKELPFNVSVV
DVSRVLVPYGLLPPSLMPLFLAPSPPPAESPSPSG
Gymnosperm
Picea abies
>MA_216501g0010
MGRFSPAVQAKSSQFFAFFVAFCIILSVQNVNSSPSSPPPAISSNSILVALLDSRYTELAELVEKASLLQPLEEV

VGNNNITILAPQNAHLEKNLDPEFKRFMHEPGNLNSLQRLLQSHIIPRRITAEEWSNNATHEALSKDGLQF
SVKENNIKAVDLAVLTRPNAVVRPDGVIHGIDRLLVPKSVQDDFNAWRRGVVLAVKPEGAPVVTKLSTLAS
VNGRQSPLVTAPVSYIAMAPGPSLAPAPAPGPSSGRHWFDGESQVKDFIDTLSLFGGYSEMADILVNLTSL
ASELAKLVSEGYRITVLAPNDEAMGKLTTDQLSEPGAPEHIVYYHIIPDYQTEESLYNAVRRFGKIKYDTLRVP
HKLLSKEADGTVVFGEGEEAAHLFDHDIYIDGRISVQGIDKVLFPTQQAGTSSHGNKGRKLEEQATAQRRG
RLIGSV
>MA_43595g0010
MRVATQLKYVSRTDDVAVDRVPAKPPVGRPSQGDGSTAKSLQNKHAGKQNSSTQVSAISSNSVLVALLDS
HYTELAELVEKALLLQPLEEVVGSSNITIFAPNNVFLEKNLDPEFKRFLHEPGNLRSLQRLLQYHVVPRRIVAE
EWGNETHHALNRDGLKFEMNGDGRKVDLGAVIHPNAVVRPDGVIHGIDRVLVPKSVQDDFNAWRSGV
VLAVKPEGSPVVDGPYQKKSVSGPGLKLYSSPPVHSIYYALAPGPSLAPAPAPGPSSGHHWFDGESQVKDF
IHTLMLYGGYNEMADILVNLTSLASEMAKLVSEGYRITVLAPNDEAMGELTTDQLSEPGAPEQIVYYHVIPE
YQTEESLYTAVRRFGKVKYDTLRIPHKMVTQEADGTVMFGHGETTAHLYDHDIYVDGRISVQGIDKXXIYV
DGRISVQGIDKVLFPPQEAASRHVKGRKLEEEQKEVEETKKPSKGRSLGALCNLLERFGRNSLLSGCQ
>MA_10051181g0010
MGNQRNAAEREKSHLPAVLWLYVVIWATSNIPGCAAATRNPAANFTARNDRMENVIETMIGAGDFGG
WANVLLAMDPAVLPTKATFFVPSDDALSRLPVQAEFTSSILQYHIIPQQLSSYDLQRLDVGMRLPTLLANQ
TIVVTSNTQPNYTVDDTLVTHPDIFYDGTFAVHGVADLLNFTRYGVDLDTPRPLGDAVPAPDSVEALTPVP
DGNSTTGSVLPRGSGSQSDAPCLCKEFPVALTAASVVFVIRFLRFPLR
>MA_153279g0010
MLTTGSRRHSFYLRFYASQRTNDRSALSSPPFTTNHHEESSLRFESWRAALIKKIAMGNQRNAAEREKSHL
QAVLWLYVVIWATSNIPGCAAATRNPAANFTARNDRMENVIETMIGAGDFGGWANVLLAMDPAVLPTK
ATFFVPSDDALSRLPVQTEFTSSILQYHIIPQQLSSYDLQRLDVGMRLPTLLANQTIVVTSNRQPNYTVDDTL
VTHPDIFYDGTFAVHGVADLLNFTRYGVDLDTPRPLGDAVPAPDSVEALTPVPDGNSTTGSVLPRGSGSHS
DAPCLCKEFPVALTAASVVFVIRFLRFPLR
>MA_10437029g0030
MGNQRNAAEREKSHLPAVLWLYVVIWATSNIPGCAAATRNPVANFTARNDRMENVIETMIGAGDFGG
WANVLLAMDPAVLPTKATFFVPSDDALSRLPVQTEFTSSILQYHIIPQQLSSYALQRFDVGMRLPTLLANQT
ILVTSNRQPNYTVDDTLVTHPDIFYDDTFAVHGVADLLDFTRYGVDLDTPRPLSDAAPASDTVETLTPVPDG
NSTTGSVLPCGSGSGRLSDAPCLCKEFPVAFTAASVIVEVQVGAGSVSAQWEPRFQRNLITILKHGRGVGGVL
QQTSICHHALNTRGDIQLHQFGTTGQAERGIADGCCGPE
>MA_99914g0010
MNFKMGFVAVSMLVAVLCLPATIDGQSDLHPTNKTSDLQVAMEEMKGSSYYGFVMLLQMLQGEQLPM
SQITFFMPSDQALAEVNLQVTSYISELVLRHSVHQPLKFNHLLHFPTGTLIPSSFPNYMLKVSNNGRNDFYL
NYVHIVHPNVCRASPSISCHGVSGVLANASTDPYPGSPAGALPAPSPSSQPPPPPPASPRNSNSSPPSQG
PSISNPPEQGPSIQNLPRSAVSPLSPISNGTGKNSASCMGLVYGSIFLLLTWSTATFLAIAQP
>MA_28601g0020
MDYQSPRSIIVAILLALFIAASNASALQKAGLPSNSSSSQVAINSNSVLVALLDSHYTELAELVEKALLLQTLEE
VVGSSNVTIFAPQNDILERKLDPEFKRFLHEPGNLKALQKLLMFHVVPRRIVAEEWRNGTHQTLAKDRLKL
SMKGDKKTIDLASVAHPNAVVRPDGVIHGIDRMLIPKSVQDDFSKWRSGVSLAVMPQGAPVLDPIKKLSK
SVAASSLSAKSPPIHSIYYALAPGPSLAPAPAPGPSTGHYWFDGESQVKDFINTLVLYGGYNEMADILVNLTS
LASELAKLVSEGYKVTVLAPNDEAMGELTTEQLSEPGAPEHIMYYHIIPEYQTEESLYNAVRRFGKMKYDTL
RIPHKITSQEADGTVLFGEGEQSAHLRQPRSSSASLEPRNI
>MA_137299g0010

MGQLDCCYFCCFLCLIWAVSAGSAASGFNITAFLWRYPEFETFNKLLTDTNVAKDINNRTSLTILALPNSILE
NYMARFGGNLQVMEIESVLQYHVLLEYMDWDRLHQMTNRGTLITTLYQTTGRAPKDSGSVNITCEKNG
EIRIRAPAPYGGSFNATVTRLVKAFPYNISIFSISTLLIPYGFDLSTSYEPSDNAINVTEVLLKANNFNFVVSMM
VASGITSDLESDQAGAGITIFAPTDDAFSALPPDTLQGLTAENKAVVLKYHVLHSYYPLGSLDSIVNPLQPTL
ATESMGAGTYTLNITRVNGSVAVDTGIVQASITQTVFDQKPLAIFAVPRVLLPREMFGKRSPVQSPNKSPS
PKVLVPSPAPQSSPLLAIPPTPPNVVPNPPSSYIPPSAAAPPSSSSRSFGEITGEPHSPSPSQHEKTSRAPTSLI
AGAFNLFSKNSLPNKIQVSDPLENKFILPSIKRA
>MA_16264g0020
MEQLGCRYFYCFWCLIWVVSAGSTAASGFNITTFLWRYQEFETFNNLLTATNVAKDINSGTSLTILALSNSIL
QNFIASFRGNLPVMEIQYVIQYHVLLEYLDWDRLRQSAIRDVYKRQLYQTTGRAPKNSGLMNITCEKNGKI
RIRAPAPYGGSFNATVTRLVKTFPYNISIFSISNMLIPYGFDLSASSEPNDSAINVTDVLLNAKNFNFVVSMM
VASGMTSELEFDQAGAGITIFAPTDDSFSALPPHTLQGLTARNKAXXXXXLQGLTARNKAVVLKYHVLHSYY
PLGSLESIVNPVQPTLATESMGAETYTLNITRVNGSVAVDTGIVQASITQTVFDQKPLAVFAVPKVLLPREM
FVKQSSVQSPKKSQSPQVPVPSPAPQSSPLLAIPPTPPDVVLNPPSSHIPPSAYTPPSSSSPSFGGPFGEPHL
LSRPQPDQTSKAMTTLITFQFTLAAQCIWIIYLGYPFFQRKFS
>MA_107g0010
MGGRGVGVGAGVGVYSYSYSGLLVAMVVCIGIAPSASGFNITTFFWKHPEFTTFNNLLTATNVAGEINNR
SSLTILALSNAQLEPFISSFGGNLPTQEVSDVLRYHVLLEYLDWERLKMMPAAGTLVTTLYQTTGRAPNNTG
AVNITTGGGNNGQVRIGMPVPGAAYNATIDGLVDKFPYTISIARISAMIFPYGFDLSGSPAPTPVGINVTDV
LVKAMNFNFLVSLMEASGVVSEFESEEAGAGITIFAPSDDAFSALNPETLQGLTAEKKAVILKYHVLLSYYPL
GSLQTIVNPAQPTLATQAMGAGSYTLNITRMDGMVAVNTGIVQAPLLPLRLLRPRFPFSPFPLHVPVFPVS
PSPSPVPVPVPVSPVSPLLSPVSVPVSPVSPLLSPVSAPVSPVSPLTSPLSSPVSGPVSPCPVKDSSVSPTPAH
SPPIAPAYSPLIAFSPSNIPVPPILAPIFPPSISSPPIPTTLGGPEGDTVSPSPSPFPLTTAKISSGAPGTPAAVTFG
RAVLGFACVVVINLLEAEV
>MA_10436490g0010
MKFLGLGFAFLLLLSFLCSANAFNITKILAKYPEYSKFNHDLSQTKLADEINSRQTITVLALSNSEMASLSGLD
LASLKRVLSLHVVLDFFDAKKLHEITNGTTLSTTLYQTTGNAPGNTGFINITDLKAGKVGFGPAAHGAKLDA
TYVKSVKQEGYNISVIEVSKPITTNVVEAPAPAPADVNITAVLIKGGCKIFAGMISATGVVKTFKDAVQGGLT
VFAPTDASFTGVTSKLLSKLSSEDKVSLLEFHALPMYSPLGYLKTSNGPISTMATTGAGKYVMTASSSGDTV
MLNTRISKATISDTLLDDQPLAIFTVDKLLKPKELFAVAPSPAPTPSPSESLAPAPAPEAESPEATPVSSPPAPA
GPAESPTEAPAASESNSTSFAPAAAGVCRGLLAFMLACMGVFLV
>MA_55418g0010
MLITIPVVVVGWRGERLGFENEDYSSFNSYLTQTKLADEINSRTTITVLALNNGAMSVLTSKRLSLSVMKKV
LSLHVLLDYFDGEKLHQISNGTMLSTTLYQTSGNALGKSGFLNITDLKGGKVGFGAASPGAKLDSMFVKSIK
EDPYNMSVLEISNVIMPSDLSAPAPSTNDLNITALLEKAGCKIFVQMITATGVLQTYSDAVASGLTLLAPTDG
AFSGTVMLKLKKLSSAQEVSLLEYHAVPAYNPVGTLKTTIAPISTLATNGASKYALSVSSAGDTVILNTGLSKS
TISSTILDDQPVVLYTISGVLLPMEIFGAAPAPAPASLSAPSSTPAPAPASLFAPSSNPALNPTPQTAISPSPSVS
APLLSPPAPPTAFPAEGPAVGGPTAGKSSASFAAPLNSNVGFVTLAFLARAYCALMFRL
>MA_7057334g0010
MADPVGATMKNCTAAIQIVACFIVMLGIVGGSHGHENAGMVLLAAELTPTLTPTPAPAPAPVFANLTALLS
LAGPFKTFLRLLEASDLLTVLQAQANNTEQGITIFAPSDTAFSSLPKKLLANLTADEIKEVLLAHCISKFYTLTDF
QDFSNPANTMATGSNGGKYNLNITGDGGTVTVSSGYVTTPIISTVHVTDPLALYTVGKVLLPEDIFGLPSPS
PAPAPSPEADSLSPSGTPASSSSSTNTTSATHSAASFLSAQQFIYLIVAIAAGALLLL
>MA_5116514g0010

VLIKGGCKIFAGLISTTGVIKSYDDSIKSSQDAVKKGLTVFAPTDGAFTDVIKKLLKKLSSEDKVSLLKFHAVPSY
TPLGTLKNTNGPVTTMASNYAVTVSSSGNTVTLNTSLSKATISSTLLDNLPLAIFTVDKVLEPKELFGTASPPS
KAPTPEPPTPEAHTPEASSPEAPAGSPADSKSSK

>MA_10433947g0020

MSGFLNITDLEGGVVAFEVPGSNTVAAKFVKSIYSDPYNISVVQISNLIVSPGLLTPSIPASSVNISAALANTG
HAIFARLITATGVLHTFQEIVDSGLTIFAPVDGAFDSVMTEIKKLSRAQQVSILEYHARPVYSPLGTLKTTDGD
VVTTMATNGAERYSLTVGVNGDNVILGTGVNSAVITSTLLDDQPLVVFSIDKVLKPSEIFAVAVPHHARSPA
PAPTPALGFPVPVSSPHLVPVSSAPTPVSSPTPTPVSSPPPVPVSSAPTPVSAPPPVPVSSTPTPVSSPPPAIS
SPSQTIFSPPAPPVESPLSLLAPSVAPSDEKAHSPISEKSSAFHVGIGSVASMLAAVGHVVLGGIF

>MA_44729g0010

MKINAMEYGRLIWSMITVMAIFCLSDANYADAMDESSIVHFQDRHDQEELVQILSNMGFTSLGMHLQS
SQDLLVGLKGPLTLFAPTEEAFRYFNSPNSSSNSSSDYLLPYHIAKGLFTYTQLQMLPDGTKLDTIASEGSLLV
TFNSRGDGQDLIYSSAQTLVDYRPVYVNGVLVSHPDLLNDGLLTIHGVDQPLPFLVLESSSSRRRKNYYSSGI
APPPAGAPALIPQSSSASPGIDHPMNIIMPFMLEDAELSLRNQGYSILALAMRIKSPELLRLQNLTIFTLSDD
SVFLKGGQDFTNTVRYHVVPNRRLLLADLVRLPAGTRLHTLLHGQSLLVTDNKPFSINHVGVKIPDAFTNR
WIAVHEIVRPFLISSHHHHQMQGISNGNRSSTDNKDHQGVMAGQELRK

>MA_8251628g0010

MTAQADGYQFTDEEEFMWVISVLRGWGYHHFANALQMVDITHLFSSGGSYTFFAPPDEALEALGSASKS
WQEFMDAVRYHIVNVQLSYYNLKHLPDGARLGTLLRSRSLTFTTFAPESENETLMVNNVSVSFPDLYYDRR
FAAHGLTAVLNPSPVDHVDVLSIRAFWIKDAAATASWDRVKGLHGKHFAPSKLLPVSSFSFKNTSVNLTTH
QEILTETGGHKEDSSTASVLPEPAPSPCDEVAANGSTKEKEAISSKKTHQEILTESGGHKEDSSTTPVLPAPAP
SPYDEVAANSSTKVKEAISSKKTHQEILTETGGHKENSSIAPVLPPPAPSPCDEVAANGSTKVKEAISSKKVSE
VIYSTTSSDSPAAALASPPRLKKFTLSLSVSLSFSSPS

>MA_10397539g0010

MEAVQCCAERLKINAMDYRRFSLTVITMLIFTLYGANYADATDELSSIVRFQDKQEEEELVQVFRNMGFPSL
AMLLHSRQNLLVGRAGPLTIFAPTEEAFRNFNSSNSSSDLSSDYLLSHHIAKGLFTYAHLVMQPDGKKLDTI
ASVGSIVVTFNSHGDLSSSTQLRAEYRPVYLNGVLLSHPDLLSDGLLTIHGVDQFLVLESSSPASAPTSAPAA
PASAPASAPAPAAPTMLSPSLPASPGPDQPLNIILPFMLEDAMLSLRDRGYSILALAMRAKSPELLRSQSLAI
FTLSDDSVFLKDGQDFVDNVTYHVVPNRRLLLADLMHLPAGTVLHTLLDGQSLVVTNSKPFSVNHVGVKI
PDAFTNRWIAVHEIGRPLVISSHPHQQHR

>MA_59950g0010

MESLKMALFNGLALVMMLLISAEAQGEDINIVNLATAPASAPFSSPPVSAPAPAPHYVNVSNVLEQAGQF
KTFLSLIAGTQAETQLQTQANNTQQGLTLFAPLDGAFSSLRPQYKAMLSKLTDQAPTLSNPLSTMGSYKFN
VSAFGAQVNVSTGLVNAPLTSSIFSQAPVAVYEVNKVLLPEEIFGLPIPSPAPSPTPVSAPTPALSPSAGVQSP
LSSSDHTNGAAHTWHLNAKDLAIQSGLAMFGFLFLLLNQQCV

>MA_5195g0010

MVLVCIMSFTILVFSSAQAINLGAVSLASAPATTPAAPAPGPVSLNLSGILDKAGQHAAPEPAEQFSAGRHD
LRPVGRRLRGSEAGDAERHYGPRQDRPAAIPRAAKLXXDKIALLQYHALPSYYTFGQFQTVSNPVRTMAS
GNGGPFGVNVTAVGNSVNISTGLVNTPVNSAVYSQNPVAVYQVDKVLLPEEIFGVKPPAAAPAPEAGAPIS
SPAVSPSGGSASAPISSPAVSPSGGSASGSSSSACGSIEALYKGLYIVFLAAGGLLFMISS

>MA_4482888g0010

VKNELKKLSSEDKESLLEFHALPIYSPRTTLKFSKRPIATMASTGPGKYDIKVSSAGDTVILNTGVDKATITSTL
LDDVPMVILTVDKVLKPKELFALAPAPVPAPSP

>MA_309953g0010

GVANLIRVTMKDCTAVIQIVSCFIFMVGIVAGGVANLIRVTMKDCTAVIQIVSCFIFMVGIVEGSHSHENAG
MVLLAAKITPKLTPTPAPAPTPAPVFTNLKDLLSLAGPFKTLLNLLEASDLLTSIQSQANNTEQERSVVGPLHS
QVLPDEKKEVLLAHCIPKFYTLTDFQNFSNPANTMATGSNGGKYNLNITAMSGAVTVSSGYVSTAITGSVR
VTDPVAVYTMGKVLLPEDIFGLPSPSPAPAPEADNSSPNETPATSSPSANTPSAASFLGAQQSIYLILAIAAG
ALLLL
>MA_89859g0010
MVSLLVHVFHREFGFQVPTMERSGNENGGRRRAIAAYLVPCYYLLICIFLLAMAMEDTPVQHMESPSTDQVFE
SLLAAGYAEMAAILRHADAAGFDLETCRNVTIFAPPDLALLPVVGSPALPHHVLCHVAAQRLSYGDLVNKTSSDR
IPSLALGHPIVVTTGPLTNQGTGKYVVLNGRARVVSPDLYGDANLIVHGIDRVLDNERDEVISNLVRRKAKKAKK
QHKKRHKRSARTRPRMACACRRHHNMIPHSPLVVSSRPLLEDLHMLRGSNRRRGAYSNSNESRIMRRATEVV
GSIPALDYVTVNDTTAATPKFGTPNYKISEGLMDMSPSGLEWLPFEDDIIVKDEDED
>MA_10360g0010
MALSSYYLPLLATMALYLSSSAVTQQEATPIVMISPTQTTTTIKNSSEALDHAIDEMKAASYYAFVMILELVGDRL
PMKGLTFLMPSDEKLAHAAASKVDLLMHHTIPRPLTFHDLLHFPTGSFIPSFLPDYMYSVANDGKNNFSLNNVK
IVTPDLCNGSPAIQCHGISSVITDNTIYSPPPSVNLPLAPHYSSNSASPPLLNDPLNNQHAPNSAMAPASLQQDS
PFQNFTTVFSRAVQSPPRYYVGHIYALILLVLSVGYFLAISDV

Amborellales

Amborella trichopoda

>scaffold00004.170
MDVQKMHGFLQIAVALSLLLCNGPNAAALQKPESIAGATASSSGFNSSPINSNSVLVALLDSHYTELAELLE
KALLLQTLEEAVGKHNITIFAPRNEALERDLDPEFKRFLLEPGNLKSLQSLILFHVIPRRIPAKNWPESDAHSH
DTLSSDRLTLTCKNRVRKVDLASLIHPDSIVRPDGVIHGIERLLIPRSVQAEFNKRRSLTSIQAVLPEGAPEVD
PRTARLKKPAHSAPAPPYTPTLPIYAAMAPGPSLAPAPAPGPGSGHHWFDGESQIKDFIHTLLHYGGYNEL
ADILVNLTSLATEMGKLVSEGYVITVLAPNDEAMAQLTADQLSEPGAPEQIVYYHVIPEYQTEESMYNAVR
RFGKLQYETLRLPHKLVAEEADGSVKFGHGEGSAYLIDPDIYMDGRISVQGIDKVLFPPDERPTPKGKKTELT
SKTAAKAPARRGKLLEVACRMLGAFGQDSRFSTCH
>scaffold00006.127
MRQFPAALLFFLCLISATSGHNITKILAQFHEFSKFNDYLSRTHLASEINRRTTITVLAVDNAAMDDILAKNY
GIYEIKNILSLHILLDYFGAKKLHQITNGTALAATLYQTTGSAPGTSGFVNITDEKGGKVSIGADDNGGVLDA
SYVKSVKELPYNISVLQVSKILNSPSAEAPAPAPTEQNLTAVMTKKGCKLFADQLTATGAEKTFVDAVSGGLT
AFCPTDDIWTKFVPKYKNLSADHKVSLLLFHGVPIYQSMQGLKSNNGPMNTLATDGAASYSVTVQNDGE
QVTLKTKAVTAKITGTIIDEDPLAVFSIDKVLQPKELFAGAPAPAPAPEEADSPSPSSAKVAAGPASDSPDDA
PADDSANDNGSVTIRGGLLGFVITVLLVVVV
>scaffold00024.68
MGNRLQHSLICFSIFLICNAQETLSFSLSPLAAQEFKTTTAYPPFPYYPETHTSLAPILSNLGYKELAMAVPFFY
DSYLSSWTGPATVFGTNDTAIRSCSHCSRTQILKERIVPGLFTYHHLSKLAHGTKIQTMRSDRCITVTTMNS
TENTAKILIDGVQITRPDLFNDDLLVIHGLDDFVFPLSPLSCFSPEKPPLTESANDMPENDTSTMSPTPDNAT
DVGKMTPSSAPATFPAISMRLSLRDAMLRLRASGFGIVALVLRAKYPELVELGNMTIFAPDDASIFSGGYAY
VNNVRFHIVPDRYLKGSDLAQLHLGSSLQTLVRGQELVVTNAGSSVGSLRINYVPLKGPDAVYNSRIVVHSI
FVPFPRIGVPDWVVSNAFEDGLFHEVGSVSEAHGLDLGGMNEPRERRSVSEDDVFFPEVESTAWMAPE
RQVPEPAITDEGL
>scaffold00028.84
MKKPSLSLLIFLSILTLSTSQPTNTSNLQEAIRDMKARSYYTFVVLLQMMVSKHANHRLSHDITFLMPSDKD
LANYNFLPGQLKELMLTHSIPNSMLFKHMLHFPTGTLLPSARHSRFLNVSNHGSYSFYLNNARIVAPNVCG

STNIRCHGLSTIIEYESSSLPVTPVTIPAPQATTPASPVTVPARPSPIPASPLTAPPLPASPEGIPAYFIPTPPSPPP
PPPPVTGGSPPYDGMALPYGEMEEEATP
>scaffold00002.569
MQLFSGSSSFNITSFFWRHPDYTTFNNLLTATNVAEEIGGRSSLTILALSNSLMAPFISGYNGNLPTQQVSDI
LRYHVLLEYLDWDRFSQITSRGTLVTTLYQTTGRAPTSMGAVNITSSNSQIHIGMPLPNAGFNASISGMVD
KFPYIISIFSIDRILIPYGIDIYSAPPPLSLFINITDVLVRAQNFNFLVSMIEASGVVYEFERGEVGAGITIFAPDDD
AFSLLNPETLQGLTADKKALILKYHVLLSYYPLGSLQTILNPVQPTLATEEIGAGTYTLNITRLRGNVAVSSGIVI
APVTQTVFDQKPLAIFTVPKVLLPKEIFGSPPPAPVPAPPPNLGEVPLVSPPHLSKPPSCSPPNTNHHNHHH
HHHHHPPRPPLPPPFSSSPAPKPSEPALHAPFISPVSSFETAPPPSSSSSSWNSSSHGLSPSISPPHMGLPM
QPPASSPSPSPETDRNKGSKGVLRETQFGPLLTCIFIVLVDQIWGRMV
>scaffold00001.234
MKGKMKLSAMAIALAWLLFMDSTRAATVSSPSPSPAPAPAPDYVNLTELLSVAGPFHLFLEKLESTKVIDTF
QNQANNTDQGITIFAPKDKAFSSLKKPSLSNLTSDQLKSLMLYHAIPEYYSLADFKNLSQQGPTSTFAGGLY
ALNFTDNKGSVSLDSGWSQTLISSSVYSTRPVALYQVDSVLLPEAIFGKAPPPAPAPAPAPDTFTPADVSPSG
SQENPFSPKSSPSTSANAAASRPVAMTALFSPVVALVHLLLVAGGVVVL
>scaffold00393.1
MAPSVLVLALTLTALACLSGAHAQAPPNVTGILSRAGQFTVLIRLLQSTQVANQITNTLNNSNQGLTLFAPT
DNAFNSLPPGTLNSLSQQDQVTLILYHVLPQFYSLQGFQTLSNPVRTQASGMGGGQYTLNVTTVASSMQ
VNVTTGLVTTPIANSINVTSPLAVYEVDKVLLPLDMFGVKPPTGAPQAAETPTAGAKTPVAAGPTAAGGG
SNAASSVARGSLLGVVMAVVVWVVQP
>scaffold00007.129
MDFGHAFALSLSLIALLSSATLAQPASAPAPTGPTNITAVLEKAGQYTMLIRLMKATQVADQMNQQLNNS
AQGLTIFAPTDNAFSSLKPGTLNSLTDQQKVSLIQFHIIPSFISVPQFQTVTNPLRTQAGSGEDQFPLNVTTS
GNQVNVSTGINDAPVANTVYTDGQLAVYQVDKVLLPIAIFRPAVPAPAPAPGGKAKKKKKEAGAPATDAD
ATADDSATSGPAVGTAAAAVFAAFMLVVGQGL
>scaffold00069.206
MAIKPPLLLLLLSLIATSASLSSAHNITEMLSAFPDYSLYNSYLTQTKLCDEINTRQTLTVFVLNNAAMSALAK
HPLSVVKNVLSLLIVLDYFDNQKLHSITDGSMLSTTLYQTTGDAKGNFGFVNITDLKGGKVGFGSAAPGSPL
DSTYTKSVKQVPYNISVMEISAPIIAPGLLSAPAPSVSQVNLTATLEKAGCKIFASLLASSGVLKNYQDAMDK
GLTILAPNDEAFKKPEVPDTAGMRVAQLVSLLEYHALPSYSPPGALKAATGPVSTLASGVRKFAFRVATSGD
EVTLDTGVDKSRIASTVLDDAPLCILTVDNVLLPEELFPHAPASAPSPVPVSAPAPSPVAEAPSPVAEVPAPA
AASPPAPTAAGSEASPSEAPAESADSKGASSGDKARPLCTAAALVFGSFACFLLLLL
>scaffold00012.127
MIAASGVAAEFESDEQGAGITVFAPTDEAFSALPPPEKLQDLSADKKAVVLKYHVLHCYYPLGSLESIVNPV
QPTLATEQKGAGQFTLNITRANNTVAIDTGLVQASITRTVFDQNPVAVFGISHVLLPREVFSGVYSSTGGSP
EYAPPPELPLEAPETEAQPTRFSAAPGRDVGASIGRRIGVSLELSLSRCGSAYLEHRVKSPITIPVGNVSLTLGI
DGVK
>scaffold00001.224
MKLSAMAIALAWLLFLDSTKAATVSSPSQSPATAPDYVNLTELLSIAGSFLLFLEKLPKPGQQQLKSLMLYHA
IPKCHSLAEFKNLSEQGPTSTFAGCQYALNFTENKGSV
>scaffold00024.69
MGNCLQLALLCFAIFLLCNAQETLSFFLAPSPTQEFNPTTYLPLSYYPETHTSLAPILSNFGYEELAMAVPFLYDPFS
SSWAGPATVFAINDTAIRSCSHCSRLQILKERIIPGFFTYHHLSKLAHGTKIQTRSSDRCITVTSTNSTQNTAKILIDG
VQITRPDLFNDGLLVIHGLDGFVFPLPPQSCFSPAKSPSWTENSTSTMTLTPENATDTRYMTPARGPKTFPAISM

RLSIRDAMLRLRASGFSIVALALRAKYPELVGLRNMTIFALDDASIFSGGHSYVNNVRFHIVPNRYLKGSDLAQM
NMGSSLRTLIQGQDLVVTNPGLGVDSLRINYVPVKGPDAVLNWRIVVHSIFVPFPRVGVPSDWVVSNAFDHG
LFHEVGSVSDEDVLFPEVERTARIAPEGHVAEREIVDEGL

Eudicots

Arabidopsis thaliana

>AT2G35860
MDSSYGATKFLLLLFLTTSIATALPDNKPVPGQINSNSVLVALLDSHYTELAELVEKALLLQTLEEAVGKHNITI
FAPRNDALERNLDPLFKSFLLEPRNLKSLQSLLMFHILPKRITSPQWPSLSHHHRTLSNDHLHLTVDVNTLK
VDSAEIIRPDDVIRPDGIIHGIERLLIPRSVQEDFNRRRSLRSISAVIPEGAPEVDPRTHRLKKPSPAVPAGAPP
VLPIYDAMSPGPSLAPAPAPGPGGPRGHFNGDAQVKDFIHTLLHYGGYNEMADILVNLTSLATEMGRLVS
EGYVLTVLAPNDEAMAKLTTDQLSEPGAPEQIMYYHIIPEYQTEESMYNAVRRFGKVKYDSLRFPHKVLAQ
EADGSVKFGHGDGSAYLFDPDIYTDGRISVQGIDGVLFPKEETPATEIKPAAPVVKKVSKSRRGKLMEVACR
MMGSRFIPCQ

>AT5G06390
MDRRIYGGSAVIHLFLFFSVLIFSAASALSKNQSPSSGSGQINSNSVLVALLDSRYTELAELVEKALLLQTLED
AVGRHNITIFAPRNEALERDLDPEFKRFLLEPGNLKSLQTLLMFHIIPNRVGSNQWPSEESGRVKHHTLGN
DQVRLSNGQGKKMVDLAEIIRPDDLTRPDGLIHGIERLLIPRSVQEDFNRRRSLQSISAVLPEGAPEVDPRT
NRLKKPAAPVPAGSPPALPIQSAMAPGPSLAPAPAPGPGGKQHHFDGEAQVKDFIHTLLHYGGYNEMAD
ILVNLTSLATEMGRLVSEGYVLTVLAPNDEAMAKLTTDQLSEPGAPEQIVYYHIIPEYQTEESMYNSVRRFGK
VKFDTLRFPHKVAAKEADGSVKFGDGEKSAYLFDPDIYTDGRISVQGIDGVLFPQEEEVVESVKKPVKKIVQ
PRRGKLLEVACSMLGAFGKDTYLSKCR

>AT3G52370
MDDLSKLLFFLLLTISITTALPDKPGSGQINSNSVLVALLDSHYTELAELVEKALLLQTLEEAVGQHNITIFAPR
NDALEKNLDPEFKSFLLQPKNLKSLQSLLMFHILPKRITSPQFSSAVVSHRTLSNDHLHFTNGKVNSAEITKP
DDLTRPDGIIHGIERLLIPRSVQEDFNRRRSLRSIAAVLPEGAPEVDPRTHRLKKKPAPIPAGAPPVLPVYDA
MSPGPSLAPAPAPGPGGPRHHFNGEAQVKDFIHTLLHYGGYNEMADILVNLTSLATEMGRLVSEGYVLTV
LAPNDEAMAKLTTDQLSEPGAPEQIMYYHIIPEYQTEESMYNSVRRFGKIRYDSLRFPHKVEAQEADGSVK
FGHGDGSAYLFDPDIYTDGRISVQGIDGVLFPEEKTPVEKKTGVPVVKKAPKPRRGKLMEVACTMLGSQF
PTCQ

>AT3G11700
MDRCIYGCSVITIFFSFFFLLNASALESGHHNITGSGQINSNSVLVALLDSRYTELAELVEKALLLQTLEDAVG
RHNITIFAPRNEALERDLDPDFKRFLLQPGNLKSLQTLLLSHIIPKRVGSNQWPEENSGRVKHVTLGHDQVL
HLSKLKGTNGKRLVNSAVITRPDDLTRPDGLIHGIERLLIPRSVQEDFNRRRNLRSISAVLPEGAPEIDPRTNR
LKKSATAVSVPAGSPPVLPIESAMAPGPSLAPAPAPGPGGAHKHFNGDAQVKDFIHTLLHYGGYNEMADI
LVNLTSLATEMGRLVSEGYVLTVLAPNDEAMGKLTTDQLSEPGAPEQIMYYHIIPEYQTEESMYNSVRRFG
KVKYETLRFPHKVGAKEADGSVKFGSGDRSAYLFDPDIYTDGRISVQGIDGVLFPEEKEEETVKKPTGPVKK
VVQPRRGKLLEVACSMLGAIGKDSYLSRC

>AT5G55730
MAKKMSSLIIIFNILLLLTTQTHAHNVTRLLANHPSFSSFSHFLTQTHLADEINRRRTITVCAVDNAAMSALT
SKGYTLSTLKNILSLHVLLDYFGTKKLHQIRDGSALAATLFQATGAAPGTSGFVNITDLRGGKVGFGPDGGD
LSSFFVKSIEEVPYNISIIQISRVLPSETAAAPTPAPAEMNLTGIMSAHGCKVFAETLLTNPGASKTYQESLEGG
MTVFCPGDDAMKGFLPKYKNLTAPKKEAFLDFLAVPTYYSMAMLKSNNGPMNTLATDGANKFELTVQN
DGEKVTLKTRINTVKIVDTLIDEQPLAIYATDKVLLPKELFKASAVEAPAPAPAPEDGDVADSPKAAKGKAKG
KKKKAAPSPDNDPFGDSDSPAEGPDGEADDATADDAGAVRIIGGAKAGLVVSLLCLFASSWLL

>AT5G16920
MRAIQTHVITFCMLVIIVTSSTRAQKSPHVPNNHELSIAIEEMEKANYFSFVMLINMLPSVNPRFLANVTFL
MPKDKTLSTSNIINQQDSVTEFLLRHSIPSSLLFEHLNLIPNGSIVPSSLPHYTLEISNGGRSNYFLNNVKIISR
NICSLGSIKCHGIDGILQSPSTVDDDSPRNNHTLPFISCPSSHNNSDQNSHNNSDQNSSRPADHTLTAPPP
VSSPISQPKSDSPSIHDVKMLTSIVIPLFGLLASLIGM
>AT1G15190
MAKISSASCFRAIFLGALIILCLPHPSTGVPLEELERAIAILRVRGRALFANAIITSDLLFDLLSDESLTLFAPTDS
MLFDLDMTHSLPFYVSTLRLHSVPLRLSLSGLRSLPNSSSLPTLLPSHRLLLTKHSSSNDSIFLDGVQLLIPGLF
DGQHIAVHGLADLLPLTAPSSPNRLVEDSTALAKSPWFLGSRFSPAPEPYFAFMDLSPAESPSVEEVSPSPS
WGEGEEDFIVGDEGGPLDGRNNGF
>AT3G46550
MANVISISHFTLLALPYLLLLLSSTAAAINVTAVLSSFPNLSSFSNLLVSSGIAAELSGRNSLTLLAVPNSQFSSA
SLDLTRRLPPSALADLLRFHVLLQFLSDSDLRRIPPSGSAVTTLYEASGRTFFGSGSVNVTRDPASGSVTIGSP
ATKNVTVLKLLETKPPNITVLTVDSLIVPTGIDITASETLTPPPTSTSLSPPPAGINLTQILINGHNFNVALSLLVA
SGVITEFENDERGAGITVFVPTDSAFSDLPSNVNLQSLPAEQKAFVLKFHVLHSYYTLGSLESITNPVQPTLA
TEEMGAGSYTLNISRVNGSIVTINSGVVLAVVTQTAFDQNPVSVFGVSKVLLPKELFPKSGQPVATAPPQEI
SLSPESSSEQPSRLVSPPREIVSSGAVKRPLGFLVLWCWCIAFCYVLV
>AT3G60900
MATSRAFTLFAFTLSLLTVASTVSGHNITQILSDTPEYSSFNNYLSQTKLADEINSRTTITVLVLNNGAMSSLA
GKHPLSVVKNALSLLVLLDYYDPLKLHQLSKGTTLTTTLYQTTGHALGNLGFVNVTDLKGGKVGFGSAAPGS
KLDSSYTKSVKQIPYNISVLEINAPIIAPGILTAPAPSSAGVSNITGLLEKAGCKTFANLLVSSGVIKTFESTVEKG
LTVFAPSDEAFKARGVPDLTNLTQAEVVSLLEYHALAEYKPKGSLKTNKDAISTLATNGAGKYDLTTSTSGDE
VILHTGVGPSRLADTVVDETPVVIFTVDNVLLPAELFGKSSSPAPAPEPVSAPTPTPAKSPSPVEAPSPTAASP
PAPPVDESSPEGAPSDSPTSSENSNAKNAAFHVNAPALFTALVTIAATSLLL
>AT2G45470
MAASQTFSLLAFTFSLLAFASTVSSHNITQILADSPDYSSFNSYLSQTKLADEINSRTTITVLVLNNGAMSALA
GKHPLSVIKSALSLLVLLDYYDPQKLHKISKGTTLSTTLYQTTGNAPGNLGFVNITDLKGGKVGFGSAASGSK
LDSSYTKSVKQIPYNISILEIDAPIIAPGVLTAPAPSASLSNITGLLEKAGCKTFANLLVSSGVLKTYESAVEKGLT
VFAPSDEAFKAEGVPDLTKLTQAEVVSLLEYHALAEYKPKGSLKTNKNNISTLATNGAGKFDLTTSTSGDEVI
LHTGVAPSRLADTVLDATPVVIFTVDNVLLPAELFGKSKSPSPAPAPEPVTAPTPSPADAPSPTAASPPAPPT
DESPESAPSDSPTGSANSKSANAAVGVSTPSLFTALVTIAAIAVSVSLCS
>AT5G03170
MATSRTFIFSNLFIFFLVIATTYGQAPAPGPSGPTNITAILEKAGQFTLFIRLLKSTQASDQINTQLNSSSSNGL
TVFAPTDNAFNSLKSGTLNSLSDQQKVQLVQFHVLPTLITMPQFQTVSNPLRTQAGDGQNGKFPLNITSS
GNQVNITTGVVSATVANSVYSDKQLAVYQVDQVLLPLAMFGSSVAPAPAPEKGGSVSKGSASGGDDGGD
STDSSDAERTGFGFGIRITTVAAIAASSSLWI
>AT5G60490
MEHSLIILLFTVLLLLTTTPGILSQPSPAVAPAPPGPTNVTKILEKAGQFTVFIRLLKSTGVANQLYGQLNNSD
NGITIFAPSDSSFTGLKAGTLNSLTDEQQVELIQFHVIPSYVSSSNFQTISNPLRTQAGDSADGHFPLNVTTS
GNTVNITSGVTNTTVSGNVYSDGQLAVYQVDKVLLPQQVFDPRPPAPAPAPSVSKSKKKDDSDSSSDDS
PADASFALRNVGSVCDAVSFCVMSVMLAWFYL
>AT2G04780
MAKMQLSIFIAVVALIVCSASAKTASPPAPVLPPTPAPAPAPENVNLTELLSVAGPFHTFLDYLLSTGVIETFQ
NQANNTEEGITIFVPKDDAFKAQKNPPLSNLTKDQLKQLVLFHALPHYYSLSEFKNLSQSGPVSTFAGGQYS

LKFTDVSGTVRIDSLWTRTKVSSSVFSTDPVAVYQVNRVLLPEAIFGTDVPPMPAPAPAPIVSAPSDSPSVA
DSEGASSPKSSHKNSGQKLLLAPISMVISGLVALFL
>AT1G03870
MATTRLTLAPLLLIAAVLLATKATAQPAAPAPEPAGPINLTAILEKGGQFTTFIHLLNITQVGSQVNIQVNSSSE
GMTVFAPTDNAFQNLKPGTLNQLSPDDQVKLILYHVSPKYYSMDDLLSVSNPVRTQASGRDNGVYGLNF
TGQTNQINVSTGYVETRISNSLRQQRPLAVYVVDMVLLPGEMFGEHKLSPIAPAPKSKSGGVTDDSGSTK
KAASPSDKSGSGEKKVGLGFGLGLIVLCLKFLF
>AT2G20520
MSSSLFSYVVLLIFLFTIPYIQSQPTAPAPTTEKSPINLTAILEAGHQFTTLIQLLNTTQVGFQVSVQLNSSDQG
MTIFAPTDNAFNKLKPGTLNSLTYQQQIQLMLYHIIPKYYSLSDLLLASNPVRTQATGQDGGVFGLNFTGQ
AQSNQVNVSTGVVETRINNALRQQFPLAVYVVDSVLLPEELFGTKTTPTGAPAPKSSTSSSDADSPAADDE
HKSAGSSVKRTSLGIVVSFALFCCSVIYIA
>AT5G44130
MATTPLLLLLLTAVFLSTEITAQRAAPAPGPAGPINITAILEKGGQFVTLIRLLNTTQIGNQINIQINSSSEGMT
VLAPTDNAFQNLKPGTLNKLSPDDQVKLILYHVSPKFYTLEDLLSVSNPVRTQASGRDVGGVYGLNFTGQG
NQVNVSTGVVETRLSTSLRQERPLAVYVVDMVLLPEEMFGERKISPMAPPPKSKSPDVSDDSESSKKAAA
PSESEKSGSGEMNTGLGLGLGLVVLCLKFLL
>AT3G12660
MSSSLTIFFFFFASTFLYTSSNSFNITNILNEHDDFSNFNQLLSETQLASTINKRQTITVLVVSNGALSSLSGQP
TSVIKKILSLHIVLDYYDQKKLKNLSKKTVLLTTLFQSSGLARGQQGFLNATVMKNGDVAFGSAVPGSSLDA
QLQDTVAALPFNISVLHISSAIMIDVKGDNAPTASPLSPVSSPPRPAESPNDDGQDFDEPPSSAPGAAADE
PSENAGSANGVSRNDSQPAFAFTLLMSFIWWFMARLR
>AT2G24450
MGLKVSSSLLCLTILLAVSSIVSAVNITRVLEKYPEFSTMTELLAKTELTPIINKRQTITVLALNNDAIGSISGRPE
EEVKNILMNHVVLDYFDELKLKALKEKSTLLTTLYQSTGLGQQQNGFLNCTKSNGKIYFGSGVKGAPQTAEY
ITTVFRNPYNLSVVQISMPIVAPGLGSPVKVPPPPPMSSPPAPSPKKGAATPAPAPADEGDYADAPPGLAP
ETAPASAPSESDSPAPAPDKSGKKKMAAADEAEPPSSASNTGLSFGAVLVLGFVASFVGF
>AT4G31370
MGLKASLSLLSLTILLVFSKVVTANNITLAFQKYSKFSTMRDLFIKTKLIAAIDKYQTITVLAVSNDAISSITNRS
EVELRNILMTHVILDYYDELKLQGMREKSIMLTTLYQTTGLGEQMNGFLNVSKSKGRVYFGSEVKNSPLNA
EYVSTVYHNPYNLSIIQITMPIVAPGLSLAIFPPPPPYVHVAPYPTPMDASVVPAPGPAADDNSPDSAVPKT
PPAPATDTPEADSPAPAPSADNEKIEAADKAKPSSSASKAGWSFDVILLLAFLASFAGF
>AT4G12730
MAYLRRAATALVLIFQLHLFLSLSNAHNITRILAKDPDFSTFNHYLSATHLADEINRRQTITVLAVDNSAMSSI
LSNGYSLYQIRNILSLHVLVDYFGTKKLHQITDGSTSTASMFQSTGSATGTSGYINITDIKGGKVAFGVQDDD
SKLTAHYVKSVFEKPYNISVLHISQVLTSPEAEAPTASPSDLILTTILEKQGCKAFSDILKSTGADKTFQDTVDG
GLTVFCPSDSAVGKFMPKFKSLSPANKTALVLYHGMPVYQSLQMLRSGNGAVNTLATEGNNKFDFTVQN
DGEDVTLETDVVTAKVMGTLKDQEPLIVYKIDKVLLPREIYKAVKTSAPAPKSSKKKPKNAEADADGPSAD
APSDDDVEVADDKNGAVSAMITRTSNVVTAIVGLCFGVWLM
>AT5G40940 (FLA20)
MNKKPSKYFPSLNRTDYKNCNFLCNGFSLKPNEKKSSKNASMASKLLTTFFLIFFVLDIDLVATSMTSVSSAVEVL
SDSGYLSMGLTLKLANQDLNLEDWQELTLFAPSDQSFSKFGQPSLLDMKYQLSPTRLPGETLRNLPNGAKIPTLR
SNYSLTVTNSSRFGGKTSINNVVVQDSPVFDDGYVVIYGSDEFFTSPTKISDDSSSSSIPSTTSSTGSIPIPSSATQT
PPSPNIASDSTRNLPNRSKPVNRFNIFESASRLLMSRGFVIIATFLALQLEDNTSGNDTKITVFAPIDEAIPNPTTKF

SDYVTIFRGHVVSQLLLWKDLQKFAKEGSILQTVLKGYEIEISLSGDILLLNGVPLIYPDLYVNDWIAVHGFNQMIV
TKEKQVDVGDSITVLNNGEQEEEGVHGEYSSELGDYGLH*
>AT5G06920
MGCCSSDCFVYFILSIALAFMAISTTLRSPPDSEPTIPIAFSSSSPSLSLNASNTLRQSNFKAIATLLHISPEIFLSSSPN
TTLFAIEDASFFNTSSLHPLFLKQLLHYHTLPLMLSMDDLLKKPQGTCLPTLLHHKSVQISTVNQESRTAEVNHVR
ITHPDMFLGDSLVIHGVIGPFSPLQPHSDHLIHTPLCQSDTTNKTSNNEEVPVSIDWTRIVQLLSSNGFVPFAIGL
HSVLNRIVNDHNHHKNLTGVTILATPNLVSLSSASPFLYEVVRHHILVQRLTYKDFASMSDKATVKTLDPYQDLTIT
RRNVNSSGGDFMISGVEIVDPDMFSSSNFVIHGISHTLEIPHV*
Eucalyptus grandis
>Eucgr.B03801
MDVPIYGVPALPAFFLASLLFLAGTCPALPDHPRPHARPSSPAGGVGGGGGVSGPGQINSNSILVALLDSH
YTELAELVEKALLLQTLEETVGRHNITIFAPKNEALERDLDPEFKSFLLEPRNLKSLQTLLMFHIVPSRIDSAD
WPEPAPSAAGHREELSLSSHRLRLAKRESGEREVDRARVIHADAVARPDGVIHGIERLLIPQSVQDDFNRR
RSLRSITAVKPEGAPEVDPRTHRLKKPAPPAKAGSAPALPIYDAMAPGPSLAPAPAPGPGGPHHHFDGEA
QVKDFIHTLLHYGGYNEMADILVNLTSLASEMGRLVSEGYVLTVLAPNDEAMAKLTTDQLSEPGAPEQIM
YYHLIPEYQTEESMYNAVRRFGKVKYDTLRLPHKMVAQEADGSVKFGQGEGSAYLFDPDIYTDGRISVQGI
DGVLFPPEEETEVKVEKKTPTKVAAKPRRGKLLELTCQMLTAFGQHSRFNSCR
>Eucgr.A02551
MDRRFCGLAAALLLLVLLLPIAAAALPHRAASNATGAQINSNSVLVALLDSHYTELAELVEKALLLQSLEDAV
GRHNLTIFAPRNEALERDLDPEFRRFLLEPRNLRSLQTLLLSHIVPARLGSPQWPLPDSRPARHPTLGSDPVH
LSVAPGSGLPAVDSAEVVRPDDVVRPDGVIHGIQRLLIPRAVQEDFNRRRSLRSIAAVIPEGAPEVDPRTHR
LKKPAPPAPAGSAPALPIYDAMAPGPSLAPAPAPGPDGPRGHFHGEALVKDFIHTLLHYGGYNEMADILVN
LTSLAVEMGRLVSEGYVLTVLAPNDEAMAKLTTDQLSEPGAPEQIVYYHIIPEYQTEESMYNAVRRFGKVRY
DTLRLPHKMVAQEADGSVKFGQGDGSAYLFDPDIYTDGRISVQGIDGVLFPAEETAPASTTAAEKKVVKVV
AKPREVFDGSSVQYARSFWATFSFFLVSIKQ
>Eucgr.F02049
MQVRRTPPHLLAALLLLLVAAATSLARAHNITSLLAGHPDFSTFNHYLSATHLAAEINSRTTITVLAVSNAA
MNDLLAEHPTTTTLKNILSLHVLLDYFGSKKLHQLTDGSALAATLFQSTGSAPGSSGFVNITDLKGGKVGFA
PEEGGSSDAGAYFVKSLEELAYNISVIEISKVLPSAIAAAPAPEPSQVNVTDVMAAHGCKEFADALLNSSDAL
KTYEDNVVGGLTAFCPLDDPFKAFRPKFDNLSAADRASFLEFFATPVYMSMSMLRSNNGLMNTLATDGS
RKFDFTVQNDGQDVTLKTKVNTAKITGTLLDEQPLVIYTLDKVLLPQELFKAAVSPAPAPAPTPEKAAESPK
AAAKHKSPPAPPTSAASDAPADSPDGTVADQKSNANARICCGRFGFMALSLLLVVLAL
>Eucgr.F03689
MQLRRRHLLLPLALSFLAAALTVARAHNITRLLAKHPEFSTFNHYLTETHLAAEINSRTTITVMALDNAAMS
RLLAPHPTIATLKNVLSLHVLLDYFGPKKLHQITNGTALAATLFQATGSAPGSTGFVNITDLKGGKVGFAPED
NNGRLDTYFVKGLEEIPYNISIIQISNALPSDVAAAPTPGPSEMNLTRLMSAHGCKVFADTLDANADAMKT
YEDNVEGGLTTFCPLDDPFKQFLPKYKNLSAPGKASFLEFFAIPMYLSMAMLKSNNGPMNTLATDGASKY
DFTVQNDGEEVTLKTRVNKVKITGTLLDEQPLAIYSIDKVLMPKELFKTAALTPAPAPAPEKAADSPKSSKAK
SPPATADSPADSPESDPADQTADSNGSVRFKSARSGFLVLSLWFGVHFLL
>Eucgr.G02210
MATPVSAIALLLLSLLPLCSPLPLESLKEATHVLSNSGFVSMSLTLNLISKTLMSPQVPSLTVFSPADDSFARFG
QPPLSLLQFHFSPHALSLDTLRSLPRGAKISTMNANHSLMVTTSPSDYQVEINGVKIVGSPVFNDGNLVVF
GIEMFFDPEFQTSAPVQSPVLGLECVAPRYGGLEDLSSGNVFGEACGAMRSKGYFAMASFLGLQLMKLKG
DNQMLTVFTPSDEAMVNHTGSFSDYPSIFLRHVIPCKLSSSDLLEFNNGILVHTHLEGFVIEVTRSGDRLML

NGVPMTSPDIYTSDWLSIHGVLDVIA
>Eucgr.G02206
MATPVSTVALVLLSLLPPRSPLPLESLREATHVLSNSGFVSMSLTLNLIPKTLMSPQVPSLTVFSPSDDSFAQF
GQPPLSLLQFHFSPHALSLDTLRSLPHGAKISTMNANHSLMVTTSPSDYQVQINSVKIIGSPVFNDGNLVIF
GIEMFFDPEFQTSAPVQSPIPGLECVAPRYGGSEDLSGGNVFGEACGAMRSKGYSVMASFLGLQLMKPK
GDNQMLTVFAPFSEYPSVFLRHVVPCKLSASDLLEFNNGTLLHTHLEGFVIEVTRIGDGLLLNGVPMTSPDI
YTSDWLSVHGILDVIAARESRQLLD
>Eucgr.G02208
MATPVSAVALVLLSLLPLCPPLPLESLREATHVLSNSGFVSMSLTLNLISKTLMSPQVPSLTVFSPSDDSFARF
GQRPLSLLQFHFSPHALSLDTLRSLPHGAKISTMNANHSLMVTTSPSDYQVQINGVKIVGSPVFNDGNLV
VFGIEMFFDPEFQTLAPVQSPIPGLECVAPRYGGSEDLSGGNVFGEACGAMRSKGYSVMASFLGLQLMK
PKGDNQMLTVFTPSEPETLGFVGARQLFVKMSQLDL
>Eucgr.K00086
MATFRALFILLVALNSHSPATAQSNSPRNPHLLTAIEEMKQADYFSFVMLINMSPPVLFEGNLTFFMPDDG
MLSRIVLPTNDVSHFVLRHSIPKPLLFDYLEHIPTGSLIPTSAAGYLVRIDNKGCRSYFLDDVRITKPNVCVAG
SSIRCHGIDGVLLPVTNVTESPSPPCPGNTAPVTAMPTPPPPPPPASDVIQSLSSASPAPATAASPFSPREKS
ASSRRLGAKGLVNSLLSGIIISAMMESRRNVL
>Eucgr.B02370
MAERISIPKITPATLLYVLLLTLPAAPTASLNVTALLSTFPELSAFTALLSSTIASDLAQRSAVTVLAVPNAYLASS
DLTRHLSQTSLADVLRYHVLLQFLSADDLHQIPSSGALFTTLFQTTGRATSNFGSVNITRDPSTGAVSVRSPA
PYSPSNASLLEPIKAVPYNLSIFTVNSLLLPYGFDLMASETRPSLDLNITKALIDGHNFNVAASMLAASGVVE
EFQADEGGAGITLFVPTDSAFTELPSSQSLQSLPADKKAVVLKFHVLHSYYPLGSLESIVNPLQPTLATEDM
GAGSYTLNISRVNGSVAIDTGIVQATVTQTVFDENPVAIFGVSRVLLPREIFGKNPSAGSKSGGAIMTAAPP
PDITPSPENSPGGFDGPPPVSREEIRSGSSVAGVQRLMLVLCCIVVYLAV
>Eucgr.H00875
MRPAPPLPTAAAALALALLLCLSLSPAADAHNITRILAKHPEFSTFNHYLTITHLAAEINRRLTITVLAVDNAA
MNAILDKHLSVGTLKNVLSLHVLVDYFGTKKLHQISNGTTLTSTMFQATGAAAGTAGYVNITDLKGGKVGF
GAEDNGSDLNAVFVKSVDEIPYNISVLHISQVLTSPEAEAPTPSPSNLNITAILSKQGCKSFADLISASGALKTF
DDNLEGGLTIFCASDAAVSAFMPKYKNLTNAQKVSFVLYHGVPVYQSLQMLKSNNGVMNTLATDGASKY
DFTIQNDGEDVKLKTKVVTATITGTVIDQDPLIVYKIDKVLQPRELFKAAAGDAPAPPAADSPEGDSADQTA
DSNGVGGGVLSGPRVVVGVVLSLCLSVVFL
>Eucgr.K00711
MKSTTVFLITTAILLLSSPEAKAQTTKSPPPSLAPAPAPTPDHVNLTHLLTVAGSFHTFLGYLESTKVIDTFQEQ
ANSSKDGITIFAPTDKAFSSLKQPSLSNLTQDQLKSLCLFHALSRYYSLADFKNLSQSSPVSTYAGGQYTLNFT
DLSGDVHVNSGWTNTKISSSVLSTDPVAVYQVDHVLLPEAIFGTDIPPMPAPAPSPVADTPPAADTPSGV
NVTTSSPSSSEQNQNSSPQLISLGIWSQLFVAVSSMMIMMSG
>Eucgr.A01074
MGEILIFYSCGHPIDSTLPSNLTHSSAPIGSQMCVLHITTQCARVLIAGPSLLTPSQSFFFSCLFKNSISSLPLAL
YILSSANTRSIPFFIASLSSSLSGTFGLGIGGSIPTYENIMELPFACIISLALLLSISPSSHAQIANAPTLSPTPAPAP
APDFVNLTDLLSVAGPFQTFLSYLESTKVIDTFQNQANNTEEGITLFVPKDGAFASQKKPSLSNLTNDQLKSL
CLFHALPHYYTLADFKNLSNVSPVSTFAGGEYSVNFTDVTGTVRLSSGWTNTKVSSSVHSTDPVAVYQIDK
VLLPEAIFGTDIPPTPAPAPAPDISPAADSPTGGSEAGSSTASKPSSSPKLSNSWGHLVLAVSSGLLVVLL
>Eucgr.C02101
MNSKAFACLFFAVFIFSSSAASAFNITKLLSRYPDFSTFNDFLTQTKLYEQINRRQTITVLALDNSAVGDISGKP

ADVLKSILSAHVVLDYYDIEKLTDLSKKSTLLTTLYQSSGTAQNQQGFLNVTKQSGEIVFGSAVKGASHNAKL
VKAVAAQPYNISVLQVSSPIIAPGIDASNPNGTVHNATTPSASPAATPKKAPASSPAKSPVADTPDSSEAPA
ADSPADAPEADAPEASAPEADAPTSSPPAPSDADADAPAPGKSSSSRVTLPVGAGIVMGLLSAMVA
>Eucgr.A01871
MASSSSSSPALSLILIACIFSLTLSPPQALAQSPSAPAPSPSGPANLTGILDKNGQYTSFIRLLTTTQVANQIEN
QLNTSTEGLTVFAPTDNAFNNLKAGTINDLSNEQQVQLVLNHVAPKYYSLLSFQSVSNPVRTQGNFAGLN
FSSQGNQVNVSTGMVETPINNALYQQSPLAVYQVDKVLLPPELFEAPAPASSTPAAPKSSSSSNSTTSAKAP
SAENSNSGASGKSNMWLGFVVGVGMMCMGFLS
>Eucgr.B02486
MSTTLFPRFQINCHSLPPLSSHNQKPSSSTTSSLSSLHNPPLTKSKAKNMMKRLSFLLLLVLLFQCSTTLAQP
AAAPAPPVIAPAAPATPALGPAPPVLGPAPAGPTDITKVLKKASQFTVLLKLMRATSVDKQINAQLNNSNN
GLTIFAPNDNAFSSLKSGTLNQLSSEQQVELVQFHVVPTYLAVPQFQTVSNPLRTQAGGSGKFEFPLTLTTS
GSSVNISTGVTNATVDQTVYNDGQLAVYMVDKVLLPMSIFGPKPPAPAPAPAKPKKKPALASSPVADSVP
VDTSGEGKLMVPGMMVFIGLSVVGTFYS
>Eucgr.A01158
MKHQLVLPFSCIAIIFLLHIAPITGQGPAGAPAAPASPTNLSSQLSNQKSNQGFTIFAPTDNAFASLKSGTLN
MLSDQEKTQLVQFHIIPSVISLTQFSTISNPLRTQAGNTDNGQFPLNVTTSGNQVNVTTGVVAASVDNAIT
SNSKLAIYQVDQVLLPLAMFGTPAPAPAPGAPKKEVPVPSRAPSGSDSAPVDSSDAVANTFNSRTLAPYVV
AATLGGVLLF
>Eucgr.J00938
MKHQFIVLALLFLINTAPTSAQPAASPTAPMVLPPPPVSLAPVSPAPDSPPPPAPPQAHLLKILQKAGGFTV
LIKLLRGTHVSDQVDSELSDLNDGITFFAPDDAAFSALKAGTLNTLSNEQQVQLLQFHMVPTYLTMSQFQT
VSNPLMTRAGGVGGKEFPLNVTTKGDKVSVFTGVDGASGEDSAQGPAPPTKKPAELSVSGSSEVGKVKT
GDVVRLVVGVGVAGVVASLGL
>Eucgr.A01741
MAISSSLHSTNESLAAPSSSPGLHQVPLNASRALGRAGFTAMAALLPIAPELFFSSPRATIFVIKDLFMANLSSSP
WLMEEVLRYHTSPLKLSVDDLLKMPRGSCLPTLLAKKNVTVTRSDAEQRLVEINGAVITHPNVFLGERLAIHGVL
QPLLHKEQELCLVQSSKRNRDPRSLSVVEKERHLVDWGRVIRSLSSSGFVSFAIGLQSVLDGILDDNADLASVTVF
APADFGFVASNSAILERMVRLHILPHKYPHEELARLPDRASLRTLVPGRDLEIGRKDASCSTDTNQRTRVAINGLE
IGVPDIYKSRNF
>Eucgr.H00590.1
MDEDLFASPQSQARRPELIRDDSLSPTSLLEPILSHLGFHDLAMAVPSLTDSPSFTTWNGPSTLFAPSDSSLKSCA
SCSVPRILSEHIVPGLFSFGYIQKLAFGTKVETMSPGHCLTVTSATHRVTNTTRIFIDGVEISRPDMFNNGIVVVH
GLEGFIAPLSPFSCSIEKMTSLAFPFSLPPPVSSSFSPSPLMRLMLRDAVLRLRTSGFGILALAIKIKYPELVNLQNAT
IFTVDDFSIFTGGISYVNNVRFHIVPNRYLAMADLERLPAGTVMPSLEAGQYLAVTTAGGPATMRINYVRMKSP
DVMRNQKVVVHSLFLPFPHLHPASLDGLINGQDHVEENVAAANQGMKDSCAALEENGDCEVIPP*
Populus trichocarpa
>Potri.010G244900
MAIALSSFNIFFTFLLVSTFHLGFSFSALQENHSNGTYSGQINSNSVLVALLDSHYTELAELVEKALLLQTLED
AVGKHNITIFAPKNEALERDLDPEFKRFLLEPGNLKSLQTLLLYHIVPNRINPSHNSSLQHHSTLCRDRVKLSS
QESGEKLIDSAKIIQVNAVERPDGVIHGIERLLIPRSVQQDFNNRRSLQSISAVKPEGAPEVDPRTHRLKKPA
PPAKPGSAPVLPIYDAMAPGPSLAPAPAPGPGGPHHHFNGEKQVKDFIETLLLYGGYNEMADILVNLTSLA
TEMGRLVSEGYVLTVLAPNDEAMAKLTTDQLSEPGAPEQIIYYHVIPEYQTEESMYNAVRRFGKISYDTLRL
PHKVLAEEADGSVKFGHTENSAYLFDPDIYTDGRISVQGIDGVLFPLEEKEKSETKKEIKSVKAVKPQRRGR

LLEVACRMLGTFGQDSHFTTCQ
>Potri.008G012400
MAMAPSPPCIHIFFASILLLSNFHLGFSSSSTLQENHSNGSYSGQINSNSVLVALLDSHYTELAELVEKALLL
QTLEDAVGKHNITIFAPRNEALERDLDPEFKRFLLEPGNLKSLQTLLLYHIVPNRINLSHNSSLHHHSTLCRDR
IKLGSQSGEKLIDSAKIIQVNAVERPDGVIHGIERLLIPRSVQQDFNNRRSLQSISAVKPEGAPEVDPRTQRL
KKPAPPAKPGSAPVLPIYDAMAPGPSLAPAPAPGPGGPHHHFNGERQVKDFIETLLLYGGYNEMADILVNL
TSLATEMGRLVSEGYVLTVLAPNDEAMAKLTTDQLSEPGAPEQIIYYHVIPEYQTEESMYNAVRRFGKISYD
TLRLPHKVLAQEADGSVKFGHAENSAYLFDPDIYTDGRISVQGIDGVLFPLEEKEKSDTKTEMKSVKVAAKP
QRRGKLLEVACRMLGTFGQDSHFTTCQ
>Potri.006G200300
MDSHIYGVSKKTLLLFTLLCLSVSSISALPHQNKTGNSTGTGQMINSNSVLVALLDSHYTELAELVEKALLLQ
TLEEAVGKHNITIFAPRNEALERQLDPEFKRFLLEPGNLKSLQTLVLFHIIPQRVGSNDWPGHKSNPSRHTTL
CNDHLHLITKNSGKKVVGSADVTRPDDVTRPDGVIHGIERLLVPQSVQEDFNRRRNLRSISAVLPEGAPEV
DPRTHRLKKPEPPVRAGSPPVLPIYDAMAPGPSLAPAPAPGPGGPHHHFDGESQVKDFIQTLLLYGGYNE
MADILVNLTSLATEMGRLVSEGYVLTVLAPNDEAMAKLTTDQLSEPGAPEQIIYYHIIPEYQTEESMYNAVR
RFGKIGYDTLRLPHKVAAQEADGSVKFGSGDGSAYLFDPDIYTDGRISVQGIDGVLFPEVEKESTSVKKSVS
SVKVATTKPRRGKLMEVACIMLGTLGQDSRFTTCQ
>Potri.016G066500
MDSHIYGVSEKTLFLFTLLCFSVASISALPHQNRTGNSTVTGQMINSNSVLVALLDSHYTELAELVEKALLLQ
TLEEAVGKHNITIFAPKNEALERQLDPEFKRFLLEPGNLKSLQTLLLFHIIPQRVGSNDWPGHKSNPTRHTTL
CNDHLHLITKNSGKKLVGAAVLTRPDDVTRPDGVIHGIERLLVPQSVQEDFNRRRNLRSISAVLPEGAPEVD
PRTHRLKKPEPPVRAGSPPVLPVYDAMSPGPSLAPAPAPGPGGPHHHFDGESQVKDFIQTLVHYGGYNE
MADILVNLTSLATEMGRLVSEGYVLTVLAPNDEAMAKLTTDQLSEPGAPEQIIYYHIIPEYQTEESMYNAVR
RFGKIGYDTLRLPHKVVAQEADGSVKFGSGDGSAYLFDPDIYTDGRISVQGIDGVLFPEVEKESTSVKKSVS
SVKVATTTPRRGKLMEVACRMLGSLGQESHFTTCQ
>Potri.011G093500
MQRLTILLSLLFLLSTSTTFTRGHNITHILGKHPSFSTFNHYLTLTHLAGEINSRNTITVCAVDNAAMSELLSKH
PSIATIKNILSLHVLLDYFGTKKLHQIREGTALAATMFQATGSAPGSTGFVNITDVKGGKVAFGPEDNEGNL
DVFYVKSLEEIPYNISVIQISKVLPSDVAAAPTPEPSAMNITDIMSAHGCKVFADTLIANPEASKTYQDSVDG
GLTVFCPLDDPFKAFFPKFKNLTASGKVSFLEFFGVPIYQSLAMLKSNNGIMNTLATDGEKKFDFTVQNDG
EDVTLKTRSITAKIVGTLIDEQPLAIYTIDKVLLPKELFKAAPTPAPAPAPEKEVADAPKSSKHKKPSSDVVPSD
SPADSPDGDLADQTADDNASVTLYGGRLVAMLLSLCSGLLLL
>Potri.001G367900
MQLTVLLSLLFLLSTATTITYGHNITSILGQHPSLSTFNHYLTLTHLAGEINRRTTITVCAVDNAAMSEILSKKP
SISTIKNILSLHVLLDYFGTKKLHQIRDGTALAATMFQATGSAPGSTGFVNITDVKGGKVAFGPKDNGGNLD
VFYVKSVEEIPYNISVIQISKLLPSDVAAAPTPEPSAMNITDIMSAHGCKVFADTLIANPDASKTYQDTIDGG
LTVFCPLDDPFKAFLPKFKNLTASGKESLLQFFGVPVYQSLAMLKSNNGIMNTLATNGDKKFDFTVQNDGE
DVTLKTRGTTAKIVGTLIDEQPLAIYSIDKVLLPKELFKAALTPAPAPAPEEAADAPKSSKHKKPSADDAPSDS
PADSPDGDAADQTADNNASVRLDGGRLVAMVLSLCLGLLML
>Potri.017G111600
MSTMLLFLLILLLISSSVLAASNPFSNAMEILSTSGYLSMALTLEITSKRLHLESSAATIFAPLDIAFARLGQLSV
LDLQYHISPVRLSGYYLDSLPFGTRIPTLLPNHSLIVTTSLSYFDGKLSINGISIEESALVDFGSLIIFGMSEFFNS
SLEISPNLTPAPAPSPSPVTSLGNTSQNESTGLDVDFFGQASHLLMPRGYSIMGTFLDAQLFGIKNQTRLTIF
APVDQAMDAYAKNVSDYSSIFRKHVVPGLFPRQDLEGFNDGTSLPTFSGGFMINLTKSGDVLVLNGVPVIF

PDMYQSDWLIIHGLNQLLTPPLKEEELVGESFSELDGAEDKPDVLDFDDYVYGAP
>Potri.019G049600
MEAFTTLLVLLMIKVLVCATSPTDIPSRSQDLVVASDEMARANYFSFVMLINMAPLDQKFQGNVTFLMPK
DRLLSKIRMHQNAVSDFLLHHSIPSPLLFDHLRHIPPGSLIPSSDPDYMLNISNEGRKSFFLNNVKISSPDLCT
AGSSIRCHGIDGVLLVDTDRHPLPACSNSTSPAIVATPPSPSLPLPDIPSFPSSAPPPGAAAPTDQEHIPKHSG
SSQLESLSLGGLLKFMATSILVLNARVLYTVGQN
>Potri.T118500
QIYNIIDAILGTGDFKNWANALGMADSTTFPISATFFIPSDNSLSPTTTSADPDIFPYHIVPQRLSFADLQQF
KTFSRLPTLLFDKSILITNNSASNFTLDGSRLTHPDIYTNAAITVHCIDNLLDHSVYGTESGKNSSKPDAVGPP
PTPASPPRPTPRTFVPSTADDEEFTVHQHGESDAACLCTEVWTVFLVLCVALASKFQRMILVH
>Potri.019G002300
MGTQNLMINKSTAKILLHLLLLSLLHQITTATLTDQELDFALLSLRSYGYTLFPNAISTSDLRLQLLNQSSNATS
TSTFTLFCPPDSLLFSVDLASTAPHYTKSLFLHVSPSRLSTSDLRNLTAASGGTYIDSLVPNHRLLITNSLAQLN
GTVDGSILVNRVRVSVPDLFLGSDIAVHGLDGILVAGFDEKVEDTSFEAATWSPANAIGSAEQNSPLAGRFP
ARRRKGRNHRHNGRNGGIRRNNHRGRRINGGHRRGVGRNVSGGTRGGGVTRGAFAMYNHRL
>Potri.014G071700
MATLQYSLLLSFTLSALVSTILAHNITDILSGFPEYSEFNKYLTQTKLADEINTRQTITVLALNNGAMTALAAK
HPLSVIKNALSLLVVLDYYDPTKLHQISKGTTLSTTLYQTTGNAPGNLGFVNITDLQGGKVGFGSAAPGSKL
DSSYTKSVKQVPYNISILEISQPIIAPGILTAPAPTPSSVNITALLEKAGCKTFASLLQTSGVIKTYQSAADKGLTI
FAPNDEAFKAAGVPDLSKLTNAEIVSLLQYHATATYSPFGSLKTSKDPISTLASNGAGKFDLTVTSAGDSVTL
HTGIGPSRVAETVLDSTPLVIFTVDNVLLPVELFGKAPSPAPAGEPVSAPSPSPVASSPAPASVEAPSPLAASP
PAPPVETPGGAPAETPFGSENSTADGSAAVHVSVPVQVTVFATVICSILMS
>Potri.012G006200
MVEEIGLPIRKLPKNISLYPIASQSKIRKILLTRLKIKHDVNILSTIVLKPDPVIDPVDPPGHGSDGLTRVNSENH
SNGTYSGQINSNSVLVALLDSHYTELAELVEKALLLQTLEDVVGKHNITIFAPKNEALERDLDPEFKRFLLEPG
NLKSLQTLLLYHIVPNRINPSHNSSLQHHRTLCRDRVKLSSQESGEKLIDSAKIIQVNAVERPDGVIHGIERLLI
PRSVQQDFNNCRSLQSISAVKPEGAPEVDPRTHRMKKPAPPAKPGSAPVLPIYDAMAPGPSLAPAPAPGP
GGPHHHFNGEKQVKDFIETLLLYGGYNEMADILVNLTSLATEMERLVSEGYVLTVLAPNDEAMAKLTTDQL
>Potri.019G008400
MINGFSTKKIQYFFNAIIVLKLDPVIDPVDPPGHGSDGLTRVNSENHSNGTYSGQINSNSVLVALLDSHYTE
LAELVEKALLLQTLEDAVGKHNITIFAPKNEALERDLDPEFQRFLLEPGNLKSLQTLLLYHIVPNRINPSHNSSL
QHNSTLCRDRVKLSSQESGEKLIDSAKIIQVNAVERPDGVIHGIKRLLIPRSVQQDFNNRRSLQSISAVKPEG
APEVDPRTHRLKKPALPAKPGSAPVLPIYDAMAPGPSLAPAPAPGPGGPHHHFNGEKQVKDFIETLLLYGG
YNEMADILVNLTSLATEMGRLVSEGYVLTVLAPNDEAMAKLTTDQLSEPGAPEQIIYGLNIEASKKPKKN
>Potri.018G097000
MAPINPAISHITPTTTLTYFLLLLLATTTTTTPILAITNLTALLSSFPDFSSFTSLITSIPSLTSDLSDRSALTLLVIPN
SYLSSSLDLTRRLSPSALADLLRYHILLQYLSSSDLHQVPPSGALITTLFQTTGRASSNSGSVNITRNPVTNAITI
SSPSPFSSSNATVLSLIMTLPYNVSIISVNSLLVPYGFDLMASETRPTLGLNITKALLDGHSFLVAASLLSASGV
VQEFEADEGGAGITLFVPTDSAFSDLSATAISLQSLPADKKADVLKFHVLHSYYPLGSLESIVNPVQPTLATED
MGAGSFTLNISRVNGSVAIDSGIVQASVTQTVFDQNPVVIFGVSKVLLPKEIFGRNPVLTSKPGNTDMGN
AQPPVSALSPENSPKMLSSAPGVREEIKSGVGGLQWLSTLHLLCVVFVCNCI
>Potri.006G174900
MARINPAISQITPTTTLTYFLLLLLATTTTITPILAITNLTALLSSFPDFSSFTSLLASTPSITSDLSNRSALTLLVIPN
SYLSSSLDFTRRLSPSSLTDLLRYHILLQYLSSSDLHQLPPAGALITTLFQTTGRASSNFGSVNITRNPVTNAITI

SSPSPFSSSNATVLSLIKTLPYNVSIISVNSLLVPYGFDLMASETRPTLGLNITKALLDGRSFFVAASLLSASGVV
EEFEADEGGAGITLFVPTDSAFSDLSETDVSLQSLPADKKADVLKFHVLHSYYPLGSLESIVNPVQPTLATED
MGAGSFTLNISRANGSVAIDSGIVLASVTQTVFDQNPVAIFGVSKVLLPKEIFGRNPVLTSKPGNSDMGNA
LPPAVALSPESSPKMLSSAPGVREEKSGVGGLQRFSTLPLLCALVVWYCI

>Potri.001G320800
MERLQHLLISLYLLILSINLTTTTAQSPAPAPAPPGPTNVIKILKKAGHFKTFIRLLKSTQLDSNLNSQLGNTNN
GLTIFAPSDSAFSALKTGTLRTLTDQEKVELMQFHIVPMFISSSQFDTVSSPLKTHAGSGARFQLNVTASGN
SLNISTGLTNTTISDTVYTDTHLAIYQVDKVLLPLDIFTPKPPPPAPAPAPKLKAESESPDDAVSKKDISSAVSF
VMHHDTVFFTVGTVVAISFSL

>Potri.012G127900
MVPQFLLSFSLILSFLLHCPPTLAQSPAAAPGPPGPTNVTKILEKGGQFSVFIRLLKATQEDVTLNGQLNNT
NNAITIFAPSDNAFSSLKSGTLNSLNDQEKAELVQFHIIPQYLSSSQFQTVSNPLTTQAGSGGRLELNVTTTG
NSVNITTGLTNTSVSGTIYTDNQLAVYQVDKVLLPVDIFTPKPPTPAPAPEKPKKRSKAAESPDAPEDNSGA
VSLTVLNDVVFFGVGIVAAIFSL

>Potri.014G162900
MKFSMIIVLSSTLLFSCTPLAYAQKVASPPAPTPTPSPAPAPSPPYVNLTDLLSVAGPFHNFLNYLESTKVIDTF
QNQANNTDEGITIFVPKDDAFKNLKKASLSNLTQDQLKQLILFHALPHYYSLSDFKNLSQVSPVSTFAGAG
GYALNFTDTSGTVHLDSGWSKTKVSSSVHSTDPVAIYQVDKVLLPEAIFGTNIPPTPAPAPAPDTSPTADSPT
SDDSAGAGSAPGKSPPNSSYRINGVGIWSQLVLAIAGVLVLFL

>Potri.015G129400
MVPQFLFSASFILFFLLHCPPTLAQSPAAAPAPPGPTNVTKVLEKGGQFSVFIRLLKATQEDVTLNGQLNNT
NNAITIFAPSDNAFSSLKSGTLNSLSDQEKAELVQFHIIPQFLSSSQFQTVSNPLTTQAGSGGRLELNVTTTG
NSVNITTGLTNTSVSGTIYTDNQLAVYQVDKVLLPLDIFTPKPPTPAPAPEKPKKRSKAAASPESPADTSGAV
SFTVLNNVVFFGVCMVAAIYSL

>Potri.014G168100
MPRPLPLLTLAISLVLLASTTTVNAHNITRILAKHPQFSTFNHYLTVTHLAAEINRRQTITVLALDNAAMSSLI
SKQLSVYTLRNVLSLHVLVDYFGTRKLHQITNGTELTATMFQATGSAPGASGYVNITDLNGGKVAFGAEDN
DGKLNAVYVKSLEEIPYNISILQISQPLNSAEAEAPTAAPTLNVTAILSNQGCKAFSDLLIASGAHTTFEENVD
GGLTVFCPTDPVINGFMPKYKNLTAPQKVSLLLYHGIPIYQSLQMLKTSNGIMNTLATNGANKYDFTVQN
DGEVVTLETKVTTATITGTVKDEEPLVVYKINKVLLPRELFKAAPEKKAPAPKGEKDVADGPNADAPSDESD
DQTADNDNGVNKMGGGRLAVVAPSFFFGVVMFFLFD

>Potri.004G210600
MRQQSSSLLFSLILFFLHCTKTSGQSPAAAPVMPPPTTPVKAPPTAPSQAPSAQVATSPGPVDVIKILQKAG
HFTVFVRLMQATTEDTELNKELNKTNNGITIFAPSDSAFSNLKAGFLNALSDEDKTELVKFHVLPALISSSQF
QTVSNPVRTQAGTGPRVTLNVTTTGNFVNITTGLTNTSISGTVYTDSQFAIYQIDKVLFPLDIFTPKPPAPAP
APELGKPRKAAPGVESPTAPKDISGALTPLILHNNALLLAVSCMVAAIFS

>Potri.016G088700
MRKQLLSPFVPFLMFFLYSSTTFAQTPSPAPSGPTNITAILAKAGQFTTLIRLLKSTQEADQINTQLNNSNQG
LTVFAPTDNSFANLKAGTLNSLSDQQKVQLVQFHILPNFLSMSNFQTVSNPLRTQAGNSADGEFPLNVTT
SGNQVNITTGVNTATVANTIYTDGQLVVYQVDQVLLPLDLFGTAPAPAPAPSKPEKDVPAKAPAGSKEDAS
VDSSGASIATVSFGVVLIAAISLKL

>Potri.002G223300
MEFSMIIMFSSTLLFLCTPVAYAQTAASPPAPTPTPSSSPAPAPTPPYVSLTDLLSVAGPFHTFLSYLESTKVVD
TFQNQANNTDEGITIFVPKDDAFKNLKKPSLSNLTQDQVKQLILFHALPHYYALADFKNLSQVSPVSTFAGA

GGYALNFTDVSGTVHLDSGWSKTKVSSSVHSTDPVAVYQVDKVLLPEAIFGADIPPAPAPAPAPETSLAAD
SPSSDSTGDGSAPGTSPPNSSYRIFGVDIWSQLVLALIGVLVLFL
>Potri.006G129200
MRKQLLSPFVPFLMFFLYGSTTVAQTPSPAPSGPTNITAILEKAGQFTTLIRLMKSTQEADQINTQLNNSNQ
GLTVFAPPDNAFTNLKAGTLNSLSDQQKVQLVQFHIIPNFFSMSSFQTVSNPLRTQAGNSADGEFPLNVTT
SGNQVNITTGVNTATVANTIFTDGQLVVYQVDQVLLPLDLFGTAAAPAPAPSKPDKDVPAKAPAGSKEDAS
VDASGATIATVSVSVVLIAAISLKL
>Potri.009G012200
MRPQSFILALSLIFFFLHCTKTLCQSPAAAPAMAPPKTPVKAPPADSSQAPSAQVATSPGPVDVNKILQKA
GHFTVFARLMQATTEDTELNKELNTTNNGITILAPTDNAFSSLKAGFLNSLSDEDKTELVKFHVLPAFISTSQ
FQTVSNPVRTQAGTGPRVTLNVTTTGNFVNISSGLTNTSISGTVYTDSQLAIYQLDKVLFPLDIFTPKPPAPA
PEPALGKPRKAAPDAESPTAPKDISGAPALLFLHNNALLLAVSCAFGAIIHS
>Potri.019G122600
SYTAWHHTNVTKILEKAGHFTIFIRLLRSTQEENHLFSALNDSSTGLTIFAPTDSAFSELKSGTLNTLSDGDKS
ELVKFHVVPTFLSTSQFQTVSNPLGTWAGTGSRLPLNVTSYPNSVNITTGLTNTSLSGTVYTDNQLAIYKIEK
VLLPKDIFASNAPAPAPVAPAPEKPTKAVPAVTVESPAAPVDISSAVWFMHNNVVGSVGIVAATVFSL
>Potri.013G151500
MKQQHSLSSFSFFLLLLHCANTFAQSPAATPAQAPAAVVAQPPAATPTQAAQPHGITNVTKILEKAGHFTIF
IRLLRSTQEENHLFSALNDSSSGVTIFAPTDSAFSELKSGTLNTLSDGDKSELVKFHVVPTFLSTSQFQTVSNP
LGTWAGTGSRLPLNVTSYPNSVNITTGLTNTSLSGTVYTDNQLAIYKIEKVLLPKDIFASKAPAPAPVAPAPA
KPTKAVPAATVESPVAPVDISSALMFAHNNVVGSVGIVAAAMFAL
>Potri.013G151400
MKQQYSISSISVFLLFLHYTNTFAQSPAAAPAQAPAVVVAQPPAATPTQAAAPHGITNVTKILEKAGHFTIFI
RLLRSTQEENHLFSALNDSSSGVTIFAPTDSAFSELKSGTLNTLSDGDKSELVKFHVVPTFLSTSQFQTVSNPL
GTWAGTGSRLPLNVTSYPNSVNITTGLTNTSLSGTVYTDNQLAIYKIEKVLLPKDIFASKAPAPAPAPAREKP
TKAVPAANVESPVAPVDISSAVTFMHNNVVVGSLVIVAAAMFACHVEGF
>Potri.013G014200
MKQQYYSLFSFSFFLLFLHCTTTFAQTSPAATPAQAPAVVVAQPPAATPTQAAQPHGITNVTKILEKAGHFTI
FIRLLRSTQEENHLFSALNDSSSGVTIFAPTDSAFSELKSGTLNTLSDGDKSELVKFHVVPTFLSTSQFQTVSN
PLGTWAGTGNRLPLNVTSYPNSVNITTGLTNTSLSGTVYTDNQLAIYKIEKVLLPKDIFASKAPAPAPVAPAP
EKPTKAVPAATVESPVAPVDTSSALMFTQNHVVGSVAIFAAAMFAL
>Potri.013G151300
MKQQYSIFSFSMLLLSLCYINTFAQSPTAAPAQAPAVVVAQPPVATPTQAAAPHGITNVTKILEKAGHFTIFI
RLLRSTQEENHLFSALNDSNTGITIFAPTDSAFSELKSGTLNTLSDGDKSELVKFHVVPTFLSTSQFQTVSNPL
GTWAGTGSRLPLNVTSYPNSVNITTGLTNTSLSGTVYTDNQLAIYKIEKVLLPKDIFGSNAPAPAPVQAPAE
KPTKAVPSANVESPVAPVDISSAVTFMHNNVVGSLVIVAAAMFACNVEGF
>Potri.019G123100
MKPQYLLSSFSIFLLFLHCTNTFAQSPAAAPAQAPAVVASPPAATPTQAAAPHGITNVTKILEKAGHFTIFIRL
LRSTQEENHLFSALNDSSTGLTIFAPTDSAFSELKSGTLNTLSDGDKSELVKFHVVPTFLSTSQFQTVSNPLGT
WAGTGSRLPLNVTSYPNSVNITTGLTNTSLSGTVYTDNQLAIYKIEKVLLPKYIFASNAPAPAPVAAAPEKPT
KAVPAATVESPAASVDISSSLIFTHNLLVGSVGLLASAMFSL
>Potri.019G121200
MKPQYLLSSFSILLLFLHCTNTFAQSPAAAPAQAPAVVASPPAATPTQAAAPHGITNVTIILEKAGHFTIFIRLL
RSTQEENHLFSALNDSSTGLTIFAPTDSAFSELKSGTLNTLSDGDKSELVKFHVVPTFLSTSQFQTVSNPLGT

WAGTGSRLPLNVTSYPNSVNITTGLTNTSLSGTVYTDNQLAIYKIEKVLLPKDIFASNAPAPAPVAAAPEKPT
KAVPAVTVESPAASVDISSALIFTHNLLVGSVGLLASAMFSL
>Potri.019G121300
MKQQLISSFSIFLLFLHCASTFAQIPAAAPAQAPAVVVAPPPAATPTQAAAPHGITNVTKILEKAGHFTIFIRL
LRSTQEESHLFSALNDSSTGLTIFAPTDSAFSELKSGTLNTLRDGDKSELVKFHVVPTFLSTSQFQTVSNPLGT
WAGTGSRLPLNVTSYPNSVNITTGLTNTSLSGTVYTDNQLAIYKIEKVLLPKDIFTSNAPAPAPVAPAPEKPS
KAVPAVTVESPAASVDISSALIFTNNILVGSFGLLASAMFSL
>Potri.019G123200
MKQQLISSFSIFLLFLHCASTFAQIPAAAPAQAPAVVVAPPPAATPTQAAAPHGITNVTKILEKAGHFTIFIRL
LRSTQEESHLFSALNDSSTGLTIFAPTDSAFSELKSGTLNTLRDGDKSELVKFHVVPTFLSTSQFQTVSNPLGT
WAGTGSRLPLNVTSYPNSVNITTGLTNTSLSGTVYTDNQLAIYKIEKVLLPKDIFTSNAPAPAPVAPAPEKPS
KAVPAVTVESPAASVDISSALIFTNNILVGSFGLLASAMFSL
>Potri.019G121100
MKQQSISFFIFLLFLQCTYTFAQSPAAAPAQAPAVVVAQPPAATPTQAAAPHGITNVTKILEKAGHFTIFIRLL
RSTQEENHLFSALNDSSTGLTIFAPTDSAFSELKSGTLNTLSDGDKSELVKFHVIPTFLSTSQFQTVSNPLGT
WAGTGSRLPLNVTSYPNSVNITTGLTNTSLSGTVYTDNQLAIYKIEKVLLPKDIFASNAPAPAPVAPAPEKPA
KAVPAANVESPVAPVDISSAVWFMHNNVAGSVGIVAAAVFAL
>Potri.019G123000
HIAQRHKHEATVNLIFYFLLFLQCTYTFALSPAAAPAQAPAVVVAPPPAATPTQAAAPHGITNVTKILEKAGH
FTIFIRLLRSIQEENHLFSALNDSSTGLTIFAPTDSAFSELKSGTLNTLSDGDKSELVKFHVIPTFLSTSQFQTVS
NPLGTWAGTGSRLPLNVTSYPNSVNITTGLTNTSLSGTVYTDNQLAIYKIEKVLLPKEIFASNAPAPAPVAPA
PEKPAKAVPAANVESPVAPVDISSAVWFMHNNVVGSVGIVVAAVFAL
>Potri.009G012100
MYFFYSVQYRPRLCPRMQPFILLFWLLFLHACSQTFCQSPAQSPAATQTKAPVPPPPPAGPTDTIQILLKAG
RFLSFVRLMKATHVDTQLFSQLNSSTDGITMFAPNDNAFSSLVAGAVGSLNDREKLEFVQFHILPRFLSISD
FQTLSNPVKTLAGSDRKFPLTITTSDNSVTVSSGLTKTSISNTIYTDKQVAIYEVDKVLVPKDLFPPAPPPAPAPA
RPLAEPDPVAPRDASSALVIAWQHRVNVVLFGAGLYIAALVMDP
>Potri.013G120600
MATTPLSFFLLSLLSLSLNAQAQTPTAPAPTPSGPVNFTAVLVKGGQFATLIRLLNNTQTLNQIENQLNSSSE
GMTIFAPTDNAFNNLKAGALNGLNQQEQVQLLQYHTLPKFYTMSNLLLVSNPVPTQASGQDGVWGLNF
TGQSNQVNVSTGLVEVQINNALRQDSPLAVYPVDKVLLPEALFGVKPPTASPPAPSSKSNSTVAAAEPSTG
KNSAGGRNVALGLVVGLGLVCMGILS
>Potri.019G093300
MATSPLSLVLLSLFLSLSLHAQAQAPAAPAPAPSGPVNFTAVLVKGGQFVTFISLLNKTQTFNQIENQINSSS
EGMTIFAPTDNAFSNLKSGALNGLSQQQQVQLLQYHMLPKFYSLSNLLLVSNPVPTQASGQEGVWGLNF
TGQSNQVNVSTGLVEVQVNNALRQDFPLAVYPVDKVLLPDELFGVKPPSASPPAPATKGSSSGKSNSSDTA
AEPSPGKNSAGGRNVALGLIFGLGFVSMGILS
>Potri.019G120800
SPAAAPAHAPAVVVTQPPAATPAQAAAPHGITNIHPRGNHLFSALNDSSTGLTIFAPTDSAFSELKSGTLNT
LSDGDKSELVKFHVIPTFLSTSQLQTVSNPLGTWARTGSRLPLNVTSYPNSVNITTGLTNTSLSGTVYTDNQL
AIYKIEKVLLPKDIFASHAPAPAPVAPAPEKPAKAVPAANVESPVAPVDISSAVWFMHNNVVGFMESVE
>Potri.015G013300
MKQLISFSFSLVLLFLHCTQTLSQPPNAAPAKAPAAATVPPPAATSAQASPPVMVPVQVSKGPVNVIKILQK
AGHFAFFTRLIKSTQEDIQLFSQLNDSRDGVTVFAPTDGAFSAIIKSGVLNSLTDHQKIELVQFHIIPRILTTAN

FQTVSNPITTLAGSGNRFALNVITTENMVNVTTGLTNTSVSAIVYTDSQLAIYQVDKVLLPLDIFAPKPLAPA
PAPPKPKKDDGAESPMVPEDTSGSVICMVHNTLLMFGVGLVAAAIPL

>Potri.012G015000
MKHHFSVFLFPAILLLLHCTQTLSQTPTAAPAKAPAAASAPPPAATSSAQASPPVMVPVQVSKGPVNVIKIL
QKAGGFAVFIRLIKSTQEDIQVFSQLNDSRDGVTIFAPTDGAFSAIIKSGVLNSLSDHQKIELVQFHIIPRILTT
ANFQTVSNPITTLAGSGSRFALNVITTENMVNVTSGLTNTSVSAIVYTDSQLAIYQVDKVLLPLDIFAPKPLA
PAPAPPKPKKDDGAESPLVPEDTSSAVSCIPLNSLIIFGAGMVAAVFTL

>Potri.019G122800
MKPQYLLSSFSIFLLFLHCPNTFAQSQAAAPAQAPAVVASPPAATLTQAAAPHGITNVTKILEKAGHFTIFIRL
LRSTQEENHLFSALNDSSPGLTIFAPTDSAFSELKSGTLNTLSDGDKSQLVKFHVVPTFLSTSQFQTVVGYHL
TSQSYTNSVNITTGLTNTSLSGTVYTDNQLAIYKIEKVLLPKDIFASNAPAPAPVAPAPEKPTKAVPAVTVESP
AASVDISSALIFTHNLVVGSVGLLASAMFSL

>Potri.019G120900
MKPQYLLSSFSIFLLFLHCPNTFAQSPAAAPAQAPAVVASPPAATPTQAAAPHGITNVTKILEKAGHFTIFIRL
LRSTQDENRLFSALNDSSTGLTIFAPTDSAFSELKSGTLNTLSDGDKSELVKFHVVPNSYLLPSSRPLSGTVYT
DNQLAIYKIEKVLLPKDIFASNAPAPAPVASAPEKPTKAVPAVTVESPAASVDISSALIFTHNLVVGSVGLLAS
AMFSL

>Potri.001G037800
MSLKSSSLFALCFSSFLLFNTVRASNITQILSQYPDFSTFSSYLTQTQLAGEINSRQTITVLVVENGNMSPLSG
KPNGEIKNVLSGHVILDYYDVAKLQKLQNKTAMLTTLFQSSGQAKGQQGFLNVTVLGSNSVAFGSAVPGS
SLSSNLVKSVSSQPYNISVLQVSNIIVSAGTGNANSTTSPVPVGPPKTSPTPASSPNKPPSTSNPPPRPSPTTA
DGPAADSPAPSPPAMDGPIAATPAADGPLADAPLDPKSDASVLTTGNNLALLALILLSAFFLA

>Potri.006G276200
MIHQYCHCYCWLKTLSNYRKLSTLLFIPYITRPLILICRLRNSFSTMNSPATHFWSTSLHAYKGLCIISFEFKHK
GSDNNKEGFGFIFLAKMDSKASSLLFIAFLCLISTSTAFNSTKILAQYPEFANFNDLLSQSGLAQEMNSRQTI
TVLVLDNGSIDGLSGRPLDIAKRILSAHVILDYYDQIKLSKLQKASTIVTTLYQASGVADNRQGFLNISRTAEGI
KFGSAMKGAPLVASLVKSIYSQPYNISVLQVSEPIETPGIENMAPPPPPGTAAVPKKAPAPAPSTKTPPAAPP
TAKTPAKSPAKSPSKAPAPSKEGPSTPTKAPAEGPVAADGPVAAGGPVADVPAESPEADTEVAEEAPAVAP
AKAASSRMHVAGATVVIGLFACIMGF

>Potri.013G152200
MASCSHWWHAPVYFIASAVLAFIAISTAMNSPSNNATRPTRPTSNYLSLNASRTLRESGFNIMATLLLISPEMFF
LSPNTTIFAIKDSSLVNTSLPPWFLKNLLQYHTSPLKLSMEDVFKKPQGSCFPTLVDRKKLAVTKIDAKERLAEINH
VLVSHPDMVLERRITIHGVLAPFSSLRSKDVYFGWESIQAPICDANSSLVSDANGPRIILEWTRIIHLLSSHRFVSF
AIGLNSVLDRILADHKNLSSVTIFAPPELEFVASSSPMLEKIVRLHILPQRATYIELAALPDKQRLRTLLPDEDLKITK
GVGVTQGLAINGVEIAAPEIFSSKEFIVHGITQAFKIAKFPNASR*

>Potri.005G079500
MESSPKLSILLILSLYIIISSTSIDGVETTTFSSNLSPQSPQPQISTSDHFHDHSFSSHTNLLAPILSHLGFTQLAMAVP
SLPADSTTTAWSGPSTLFAPSDSSLRTCFSCSIPDLLHEHIVPGLFSIDYLRKLAFGTKIETLSPGRCITVTSTSLKNDS
ATPSTVKVFIGGVEITHPDLFNNGVLIIHGIQGYIAPLSPFSCDFERLSSLSFPFQEGVTPHVTSTTHQQGIGTLVQ
PAIMRLMLRDAMLRLRSNGFTILSLAMRVKYPELTNLVNMTVFALDDVSIFSGSHGYISSVRFHIVPNHYLSTADL
ERLPVGATLPTLERGQALVVTSAGGLTGFNTAVPMRINYVRVKPDVMRNLKIVVHAVYLPFPRIHPTSAAAFD
EMMGIGGEGQNIVAAEDGACSAVFEEDGSCGTVPPMPAQVKPSVVVRSDEDHHGL

>Potri.001G440800.1
MAKYLFSFLLLLTTTTATSTFNPICTFSPTPSPTSATTPAPITNSSTNIALSPTFAPSPIITIPTPAPSSAPTPINNTTTTT

TTTSTTLTPTTTPSTARTPIPTNKITLTPTFAPSRAPTPTIITPILGPSPAPTPISSSAFTTTPTFGPSPAPTPTNSSIFTAT
FSLPPTPPSTFTRQQDDLKFVFQEQIYNIIDAILGTGDFKNWANALGMADSTTFPISATFFIPSDNSLSPTTTSADP
DIFPYHIVPQRLSFADLQQFKTFSRLPTLLFDKSILITNNSASNFTLDGSRLTHPDIYTNAAITVHCIDNLLDHSVYG
TESGKNSSKPDAVGPPPTPASPPRPTPRTFVPSTADDEEFTVHQHGESDAACLCTEVWTVFLVLCVALASKFQR
MILVH

Monocots

Brachipodium distachyon

>Bradi1g06290
MAGAPAYTGAAVVLLLFVLAISGSAADTSPEEPTLPSSSASAAAVDAATAVVNGSGAGISSNSVLVALLDSH
YTELSELVEKALLLQTLEDAVGRHNVTIFAPRNEALERDLDPEFKRFLLEPRNLKSLQSLLLFHVLPSRHPAAS
WPASAAHPTLSGEDLELAAGSNGSMRVAHASVTRPDAVLRPDGVIHGIERLLVPRSVQEDFNRRRSLAAIS
AVLPTGAPEVDPRTHRLKKPAPPVLPGAPPVLPVWDAMAPGPSIAPAPAPGPNSGKLHFDGHSQVKDFI
QTLVLYGGYNELADILVNLTSLATEMGRLVSEGYVLTVLAPNDEAMARLTTDQLSEPGSPENILYYHMIPEY
QTEESMYNAVRRFGKVRYDTLRLPHKVVAREADGSVKFGQGEGSAYLFDPDIYTDGRISVQGIDAVLLPED
DTKKTSTTTPAAPVRKAPAVTGKSKNKLRRGKLMEATCHMAGIFGQRSRLTSCQ

>Bradi1g57040
MGAPLYGAVLLLSLFLAASAAVPQPQKPTTLPSSSSTTAAAAGSTTTTTTTTSSSSAAGVNSNSVLVALLDSR
YTELSELVEKALLLQQLEDAVGRGNVTIFAPRNEALERDLDPEFRAFLLQPRNLRSLQRLLLFHVLPSRLSSSQ
WRSAASLTLSGEHLELSLSDSSTTAMLAGSAAVTHPDAVVRPDGVIHGIEKLLIPRSVQEDFNRRRSLAAISA
VLPTGAPEVDPRTHRLKKPAPPVLPGAPPVLPVWDAMAPGPSIAPAPAPGPNSGKLHFDGHSQVKDFIQ
TLVLYGGYNELADILVNLTSLATEMGRLVSEGYVLTVLAPNDEAMARLTTDQLSEPGSPENILYYHMVPEYQ
TEESMYNAVRRFGTVRYDTLRLPQKVTAREADGSVKFGHGEGSAYLFDPDIYTDGRISVQGIDAVLFPPVD
GDAAAAGKNAGAAPARARKAPAVTGSAKPKLRRGKLMEGACKVIAMFGQRSRYTSCQ

>Bradi4g34420
MDARRRLCLVAAVLLAAAAAAEGYNITKILGEHPEYSQFNKLLTQTRLAADINKRRTITVLVVANGDMGAL
AAGRTLQTIRHILQMHVLVDYYGAKKLHQLAHGVTASSSMFQESGAAPGMSGYVNITQHRGGHVTFTAE
DASDSASPVSFVKSVKEIPYDLSVLQISKVLSSPEAEAPVAAPAPVNITELLSKKYCKSFAGLLAADADAFSTV
NATKDNGLTLFCPVDAAVASFMPKYKNLTAKAKTAILLYHAVPDYYSLQLLTSNSGKVSTLATSSVAKKDYSF
DVSKDGETAALDTKVITASVTATVKDDDPLAVYAVSKFLQPKELFKVAKDLAPAPAPEGPKKKSSKKKPSAAS
SPSDDDDDSAADSPDDDSGSDVDAADKAGAAPPMLARWVTAAAATVAALALAT

>Bradi2g16560
MAPPSRVLLLAALFLAASTMATAQKAKAKPAPAAADGPGAAAADAGPPTNVTSVLEKSGKYGTFLRLLHE
TRVDTQLNSQLYDSYNGLTILAPTDAAFEDLPSGTMNGLSSQDQIQMMLYCVLPRFYSLSMLGTLNGKVS
TQASGSDGPYEYKIKPSGSNVNVSTGVKGNNMLLSTIVSKEFPLAVYSVDKVPLPYALFGPQPPTPAPAPAP
APSKSKSKKKKKVIADPPESDSDDSTASDTKSAAAARVSASRWVAAAALAVLGAAGGVLL

>Bradi4g39640
MASYFAASLLVALVVSVTTIAGAPSPSAVDGDIASTTQEMQQARYFTFVMLIRMVQEQIPRNTTFLMPSD
RLLSTASVPGNQVLDLLLRHSVPAVLMFADLNRLPNGTVVPTRHTNQMVTITKREHRQLYFNNIELTSPDIC
RGGDSFRCHGINGVLRPTATRRGRGAACPRSNAPTTAAPEPASAANQSLGTPSLTPGVVSATSPAVEPTES
SQSSDTSASRSGLSCTTLILVLMISIF

>Bradi2g54620
MGRGVPPDSRPPLGPLVLLVLLAAVLPSPAAGVNVAAVLAAFPNFSDFLRLLTSSPVAGELTGRSSLTLLAVP
NANLPRSPSAFAAASGADLADVLRYHVLLEYLSPSDLARLPASGKLVTTLFQTTGRAPSDLGAVNLTSSAAG
GHSSTVVVHSPAPSFPGSNATVLGAVTREPYNISVLAVGGLIVPSGFDIAASGSESRPAVNITRVLADARGF

NVAASMLEASGVADDFEADERGAGITVFVPTDDAFAAGGLPDAAGRLQSLPADRKAVVLRFHVLHSYYPL
GSLESIVNPVQPTLATECTEAGRFTLNITRSNGSVAIDTGVVQASITRTVFDQNPVAVFAVSKVLLPKEMFG
QGNTASAAAAAAPPAAMAPDASDDGPTPPTKLSSPPVWATARGANWSCIGFVYLQLHLLLLLLLLLPLG
N
>Bradi2g23270
MRGQIPPILLVVLAAAALLPPQPAAAVDVAAVLAPFPDLAGFARLLASSPVGRELAGRSSLTLLAVPDRDLP
QSPSAFAAAAGADLADVLRYHVLLEYLAPADLRRLPASGKLVTTLFQTTGRASSDLGAVNITAGGSSLSSTLA
VVRSPAPFPASNATVLGSITAVPYNLSVLSVSGLIVPSGFSLSASESRPAPAVNITRILSDARGFNVAASMLEA
SGVAEEFEDDERGAGITVFVPTDDAFASLPAGPGDRLQSLPAERKAVVLRYHVLHSYYPLGSLESIVNPLQP
TLATEFSSQAGRFTLNITRANGSVAIDTGVVQATITRTVFDQNPVAVFAVSKVLLPKEMFTRSTADPADIVA
AGVAAIAPPPPAAGSMPPESARTPPTKLSSPPGMRGGDGGGNGNLTALPSSAMGRRARIGWWCIALLC
CF
>Bradi4g33490
MIPPKDLDQGRRSRANSVVMELKGAIFTTAVLATLLCSPALAQKSPPAPASVALPPTMAPAPAPAPDYVDL
AALLGVAGPFHTFLSYLQKTNVIETFQRQANKTKEGITIFVPKDSAFAALKKSTFSNLTSDQLKMLLMYHAL
PEFYSLAQFRNLSVLNPVNTFAGAPYTLNLTDDMGTISVKSMWSKPTISSSVYATDPVAIYSLNKVLLPMQI
FTKDPPLAPAPAPAPESGASDLAPSPGGKAGAGKGKADSTSAGYGLGVGAVNCLLAAGGLMMFLW
>Bradi5g18950
MAAPRPLLFLLALSLAFAATCCAHNITGILDGFSEYSLYNSYLSQTKVCDEINSRSTVTCLVLTNGAMSSLVSN
LSLADIKNALRLLTLLDYYDPKKLHSLHGGSELTTTLYQTTGDASGDMGHVNITNLRGGKVGFASAAPGSKL
QSTYTKSVKEEPYNLSVLEVSDPITFPGLFSSPSAASSNLTALLEKAGCKQFARLIVSSGVIKTYQAAMDKGLT
LFAPNDDAFQAKGLPDLSKLTSANLVTLLEYHALPQYAPKASLKTMKGGIPTLASTGSGKYDLSVVTKGDDV
SMATGMDKSRVASTVLDDTPVAVHTVDSVLLPPELFGGAPSPAPGASADAPASAPTPEASAPAPSPKAGK
KKNKGKSPSHSPPAPPADSPDMAPADAPDGDEEADKVHDKKNGAAALAVSFAAVIASVALVGASFL
>Bradi3g39740
MEFRAGLFTAAMVAAAMIILSSTPALAQKAKDKSPPAPPADILPPSPAPAPSPHIVNLADLLSVAGPFHTFL
DYLEKTDVLKTFQSKANDTKESAEGITIFVPKDSAFSSLRATTFANLTGEELKSLVLYHALPKYYSLAEFNKLSS
LNPVPTFAGSQYTLNLTDNMGSIRVKSMWSNPKISSSVYSTRPVAVYEVDKVLLPMQIFKSDPPLAPAPAP
APVDAKADDDAALSPASGKAASAKAKAGEKSAASQGAGGGYLAVAAAGGVMMMLLW
>Bradi2g46120
MAARRTGIAAVAAAAFFAMVSVSLAQAPAPDATPSGPPNVTAILVKGGQYTTFIRLMKETQQDTQLNSQL
NNSFNGNGYTVFAPTDNAFNNLKPGTLNSLTQQQQVALVQGHVLPQFYSMESFQTASNPVRTQASGQD
GPFTLNITATANNQVNVSSGVSEVTVNNALSDKKPLAVYSVDKVLLPLEFFGAKAPAAAPAASKDGKTKKG
EAASGPAGSDDEASPTSAAAGGARAVAWGVAAMAAVLGYLL
>Bradi2g16570
MATARSVLVAVLLSLALPAVLSQKPAAAPAPSPASTAPNVTAVLDKAGQYTKFMRLMKSTQQDTQLNSQL
NGSDTGFTVFAPTDNAFDSLKAGTLNSLSQQEQVSLVQAHIVPAFFSMESFETASNPVRTQASGADGPYT
VNVTATSNGQVNVSTGLVSTMVGTALRKEKPLAVYSVDKVLLPYDLFGPKPPASAPPAPGKKAPAKGGAK
APAGEEDSAPASKASGGVVGFAWSFLAAVAAACLL
>Bradi5g13100
MNLAVSLLIATALSLSLPLAGAHRRVLADSPPAPPPQSFAHGSRRNLTYILAPGGRFQTFVMYLQQTGLVEV
FEIQAHRTHHGITILVPTDRAFAAIEPSVLSGLKKHQVKSLMMYHALARHYALKEFDALSRVSPVTTFAGGL
YTVNVTYDAGAIRVVSSWADAKVVRPVYEMPPMAVYEIDRVLLPDAIFHAQPAVEAIPPSPDGTTPPSDG
DATKTPGGKAGGTLDAKSSACRALGRAASYVIAAALCAVAL

>Bradi2g34650
MAMQTQTPSMSTPMILLLALLCLSAAARSAHAQTPAATAPAPATAAATPKTIKAVLTKAGQFTKFLQLLQST
QEDSQIDNQLKGKSSSGGLTVFAPPDNAFSALKSGTLNALSDAQKTSLVQFHVVSQLIPMAQFDTASNPLR
TQAGETRPGKYPLNVTADGQQVNISTGVVNASVSGTVYTGDRLVVYQVDKVLLPWALYGPALPPAPAPSP
VEKEKEKGKVGPKAAADAPAADAATASEAAVPARPGGVWGQGGVFGVAVAVAAAWFVV
>Bradi2g00220
MEAMQRSFLVLLVISAAITASAQGPTAAPTTPAPAAPAPAAGTTKTTNITGVLAKAGQFNTFIRLLRSTGVA
AQIDNQLNSSQTGGLTVFAPTDNAFTSLASGTLNSLSDSQKNSLVQFHVLSTAVPMSQFDTVSNPLRTQA
GSSSPGEYPLNVTATGQQVNISTGVVNATVDNTLFTGDQLVVYQVNQVLLPMAIAGKKADAPAPAPLGPA
KKTPVAADAPGGADADTDSTASRAAGGGGGGGVVAVAMALASCVLCWGL
>Bradi5g13110
MELKRAVLVSALLCLALSRGALSQRAPIITIETPAPAPAPRHVDLAELLSLAGPYGTFLDYLTKTDVIKTFQSQA
NDTEEQGGHGITVFAPQDSAFAAVDSAALSNLTADRLRSLMLHHAAPKYYPLSVFSALAASSTPVSMFAYS
VNVTDKAGKTGVVSGWAAAKLVSSVYSTRPVAVYALDRVLLPKEIFPTAPEVAPVPVPAPAPVPGKAMDA
APGAGAKDNAGGKSSSSRVGTGRLLLGSLVLMVSGVLV
>Bradi3g39010
MRRVHLAAVLVLLLPIAVSAAGGAKAPAPAKAPPAPPNITAAMEKGGCKAFAALLSASPDASSTFQSAIDG
GVTAFCPSDGAVKSFLPRYKNLTAAGKAALLLSHAVPVYYTRRALKSNNGVMNTLATDGGAGNFNLTVQN
VGDQVSVKTAGKGKGAARVESTVYDKEPVAVYGVDAVLEPVELFEPAEVESPAPAPAAHKKAAPKKARHG
DDVADAPSPDADDDDEEDDAPPADQRKSSKNNAAAPPGGQCLRWLAVVAVAAAAVLA
>Bradi3g05730
MAPPPPNVKTRPFLITLAAIVLLLPAASADNAAAPFNITKILSAYPEFRVFSSLLNETGLARAIDRCKMVTILA
VNNTGVPDTVLRAPRPLLFDLLALHVVLDYLDPEKLDAMRLGRTGNGSMVTTLLPGPSDKFLRVAGGDKS
RITFSYAGPGGRWPRNATLIRVVTSQAFSVMVLEVSGLILPTAVPATTAARFDIIKILSSYPEFSVYSGLLRETG
LASILDGRRVVTVLAPNNTDIPKVIHTLPRPLLADLLALHVIPDYLDPEKLDALRRGRTGDGSVVMTLLPGP
GLRLLGIAGEGVPADVDTTSPLDITKILSSYPDLTAFNSLLTDSGLARAINARPTVTVLATNNTALADSLRGLR
HLPEPALVDLLALHVVLDYLDPEKLDALRRGRTGGGSIVTTLLQESGRARGRGVGFVRVSGGEDGRITFSCS
TPGGGGPRNATLVKVVTTQAFSVLVLQVSNLILPPGIVAPAPQQPRARRHMFLPPSPAPAPAPRYPPVSGV
PPPVVEEPEPNLTDTAPPPSGVIPLPSAHGGVSAKIPTAAGRHRAAGIWWSGASAALGITAYLLGRL
>Bradi1g76630
MAGERPVVAPGVVVLLLCCLAAPMVRGGGGGGGVQHNITSFLNGHPEYKLYNKYLTETRVCDEINARAA
VTCLVLNDGAMATLVADAGESSLAGIKAALRLHSLLDYWDTKKLRSLTTTNLTDTFFQAAGDAMGTVKIAK
LDSGDFGFASANKGAKFDATLVKAVKQTPYDFAVLEISAPIEFDGLFDVPDAANLTRLLEKAGCKRFAALVA
GNPGVLKAYQAAMATGLTLFAPNDDAFVAKAGTPDVGKMEKDDLVRLLMYHAVPAYEPKPSLKLVKAGA
RPLRTLASTAAGEYNVTVVARGDDVSLDTGVRKSRVAATVLDEVPLCVLTVDSLLMPVELFADAPMPAPAP
APAMAPADAPLGSSSSSPPAPPPADAPSEVADHKAKHVKSTAAALRSIGAVAVALCSVVFASLL
>Bradi4g33040
MHHSQPQLAIALLFVVVVALPLVMAAGKAAPAPAAAAPPNATAAMAKGGCKAFADLIAASPDAASTYQS
AADGGVTVFCPSDAAVRAFMPRYKNLTADGKASLLLFHAVPVYYSPGSLKSNNGVMNTLATDGASKNFN
FTLQNEGEAVAIKTGASPGGGVARVEATVMDRDPVAVYRLDAVVEPLELFKPVPAPAPAPAPAADAPRAG
KGGAARRHPAAPAVADAPGPDADDDTAPADKKKDSKKSAAAPGAPRFRWFAAALAAVAVASVLV
>Bradi1g43531
MSSSGILPCSSLLLLLLLVTLLSVSAPPPCHGAAAGQQPTHNITDILLAANANFTDFSSALASANLTAQIDGR
TPITILAVDNAAVAQLKARRLAPESLAHVLSLHVLLDYFDDARIRHLNGSFNLAATLYQTSGAAPGSAGFVN

VSSAAGSDGRVSFLPSGAAPAYAVFYQKSVKESKYDISVLQVSGVVSSPAAEGKAAPATPPPQAATPAPSPA
PVAAALAPVAKPAPAPAREAPAPSPKGNTTAPPAEAPEPPADEPPADQQKNAASGGTEEWSLGAPVAAV
VLAVVFLMW

>Bradi1g31333

MAPPKLSLLALLFLLFPAAGNAAAAAAADPADTVAAAAPPALFNVTEILGRFPDFTLFNYLLTKTKVSAQI
NRRGTVTILAPVNADVDWLLRRSSRLPRAALVELLAVHVILDYIDAAKLAALPRGRPGAPPVVATTLYQTM
GVAPAGDRSGFLTITPTGNGGAVFASAAPGAPRFGPPPPRRIRSMAVAPSPAPAPVLPAVPPVTMPVEEGT
TETIPVAAPEPSDGNRAVEAMRLWFGAGVVMALACVLVTGL

>Bradi5g04846

MASPWVTTTTALLLLLTLASFSNSAKFNITEIISESPEFKTFNSLLSKTKVAEEINARQDAITVLVVDDKAAGAI
TALPADTQKKVLAVHVILDYYDPMKLDSVHGKTAMLTTLFQTTGAAADSMGLIKYTQGADEQMSFASAE
PGSSASSQLVKVVACRPYNLSVMQVSAAIIPPGIGKGLSGSGNGSSSPTISGPETPPLGTEAIDADAPDQM
PDYASDQDVDGPSSPAEPSPTAKSDKGTTDSLASAGNKLRQDIMGTSSVVGIMGALVMFVIW

Oryza sativa

>LOC_Os03g57490

MGAPVCGALVCFFVLLVARAAAETAPVERPTLPSADVAIDGNGNGARAGAGKAAGAGAVGGGIVSNSVL
VALLDSRYTEMAELVEKALLLQALEDAVAEHNVTILAPRNEALERDIDPEFKRFLLEPRNLNSLQSLLLFHVLP
ARLPSGSWPAAATHPTLAGEDVELAAADGGGGMRVAHAAVSRPDAVVRTDGVIHGIDRLLVPRSVQDEF
NRRRSLADISAMPPTAAPEVDPRTDRLKKPAPPGDSPVLPISDATVPDPSVALASEPSDHYDGDSQVKDFI
QTLLKYGGYNELADIFVNLTSLATELAQLVSEGYALTVLAPNDEAMARLTADQLSEPGSPENILYYHMIPEYQ
TEESMYNAVRRFGKVRYDTLRLPHKVVAREADGSVKFGHGEGSAYLFDPDIFADGRISVQGIDAVLFPPAE
TKTKTKPATARIRTAPAITGVSKIKHRRGKLLEAACQMVGILGQRSQFTRCQ

>LOC_Os03g57460

MGAPVRGALVFLLLLAGAAAETAPEEPTLPSAAADGAASSGTTSGAGAGTGINSNSVLVALLDSHYTELAE
LVEKALLLQTLEDAVGKHNVTIFAPRNEALERDLDPEFKRFLLEPRNLKSLQSLLLFHVLPARLPSGSWSAVS
HPTLSGEEVELAAAANDGAMRVAHAAVTRPDAVLRPDGVIHGIERLLVPRSVQEDFNRRRSLADISAVLPT
GAPEVDPRTHRLKKPAPPVPPGAPPVLPIWDAMAPGPSIAPAPAPGPGSGKHHFDGHSQVKDFIQTLLLY
GGYNELADILVNLTSLATEMGRLVSEGYVLTVLAPNDEAMARLTTDQLSEPGSPENILYYHMIPEYQTEESM
YNAVRRFGKVRYDTLRLPHKVVAREADGSVKFGHGEGSAYLFDPDIYTDGRIAVQGIDAVLFPPDDTKTAP
DTAPVRRAPAVTGTRKKNKLRRGKLLEATCQMAGILGQRLRFASCQ

>LOC_Os07g06680

MGVSALLLLLLAAAATAAPPEQPALSATPSSAAAANNSSGGGGGAASNGVNSNSVLVALLDSHYTELAELV
EKALLLQTLEDAVGKGNVTIFAPRNEALERDLDPEFRRFLLEPRNLRSLQRLLLFHVLPARLHASDSSSPDFPS
SHPTLSGERVDLSASPMRVGAAAVTRPDAVVRPDGVIHGIERLLVPRSVQEDFNRRRSLAAISAVLPTGAP
EVDPRTHRLKKPAPPVPPGAPPVLPIWDAMAPGPSIAPAPAPGPGSGKHHFDGHSQVKDFIQTLLLYGGY
NELADILVNLTSLATEMGRLVSEGYVLTVLAPNDEAMARLTTDQLSEPGSPENILYYHMIPEYQTEESMYNA
VRRFGKVRYDTLRLPNKVTAREADGSVKFGHGEGSAYLFDPDIYTDGRISVQGIDAVLFPPKDTATGGEGS
GSGSSGAAPARKAPAVTAHSKSKLRRGKLLEGACQVMGFLGRRSRFASCQ

>LOC_Os08g38270

MRHLRLAAALLLVVLFPLAVSAADGKAAAAPAKAKAPAAPPPAPPNITASMAKGGCKAFAALVAASPDALS
TFQSAADGGVTAFCPTDDAMRAFMPTYKNLTADGKASLLLFHAVPVYYSLRSLKSNNGVMNTLATDGSA
KNFNFTVQNEGDKVTIKTDASDGVARIKDTVYDKDPIAIYAVDTVLEPVELFEPAESPAPAPAPVADAPKAS
KSKKASHRHVADAPGPAGDDAPPADQRKSSKKNDAAAGASCLRWLAALPVAVAVAAALA

>LOC_Os06g17460

MALAGTLNASTVFFLLLLLLCVSVMSPPFTPASASPMGLTADAKVALLLYHAVAAHFSEEALKAINGEVNTL
ATDGGGGGKVLNLTIEEDDDGAGATVKLSSSSGNVARVTKTIQDADPHAVYLIDAVLMPLDVVVNVSSGG
GAAAPSPAPVTSPAPAPAQATNPSPSPDSKPDNQPAAEQPPENSASRGGMAAWSLLSVVVPAIASLVLR
>LOC_Os01g62380
MRGGAGALPVPGGLALGVVLLLVLAVLPSRAAGVNVTAALAAFPSFADFARLLESSPVAGELAARSSLTLLA
VPNNNLPRSPSAFAAASGADIADVLRYHVLLEYLSPSDLARLPASGKLVTTLFQTTGRAPSDLGAVNLTVGG
NSTVVVRSPAPFPGSSATVLGAVTAVPYNLSVLAVGGLIVPSGLDVAASDSRPAGGVNITHVLADARGFNV
AASMLEASGVADEFTADERGAGITVFVPTDDAFADLPATDRLQSLPADRKATVLRFHVLHSYYPLGSLESIV
NPVQPTLATERFEAGQFTLNITRVNGSVAIDTGIVQASITRTVFDQNPVAVFAVSKVLLPKEMFGRGGADS
DVMAPPPDAMAPDAAENVRTPPTRLSSPPALRGGADSESSSALSTARAVNWWCIRLVLLNLHLLLLPLV
>LOC_Os04g48490
MAATDRRLLFLLAASLAVAAVSSHNITDILDGYPEYSLYNSYLSQTKVCDEINSRSTVTCLVLTNGAMSSLVS
NLSLADIKNALRLLTLLDYYDTKKLHSLSDGSELTTTLYQTTGDASGNMGHVNITNLRGGKVGFASAAPGSK
FQATYTKSVKQEPYNLSVLEVSDPITFPGLFDSPSAASTNLTALLEKAGCKQFARLIVSSGVMKMYQAAMD
KALTLFAPNDDAFQAKGLPDLSKLTSAELVTLLQYHALPQYAPKASLKTIKGHIQTLASTGAGKYDLSVVTKG
DDVSMDTGMDKSRVASTVLDDTPTVIHTVDSVLLPPELFGGAPSPAPAPGPASDVPAASPAPEGSSPAPSP
KAAGKKKKKGKSPSHSPPAPPADTPDMSPADAPAGEEAADKAEKKNGATAAATSVAATVASAAALLAASF
L
>LOC_Os08g39270
MMESKAAMMVTILLCCSSISPAFAQKHKGPPAAAAVSLPPSPAPSPAAPRHVDLADLLSVAGPFHTFLDLL
EKTDVLRTFQSQANGSKDGITVFVPKDAAFASLARSATANLTSDQLKSLALYHALPRYYSLAEFNRLGGAAS
PVPTLAGGEYTVNVTDDMGTVHVGSMWSNPKISSSVYSTRPVAVYEVDRVLLPMQIFRTDPPMAPSPAP
APDAKPASDAASPLPGKSSSAKAKADEKKSSSSPPSSRRGAGIAGYFLALAASASAGLLLLC
>LOC_Os01g06580
MQAMQRLVLCLVVSMAIAASAQGPTAAPAAPAPATPAAPATPAAPAPASTKTTNITGVLAKAGQFNTLIRL
MRSTGAAEQIDNQLNSSRNGLTVFAPTDNAFTSLPSGTLNSLSDQQKNSLVQYHVLSTLIPMSQFDTVSN
PLRTQAGSNSPGQYPLNVTAEGQQVNISTGVVNATVGNALYTGDNLVVYQVDKVLLPMAIYSTPAPAPAP
LSPATKKKGKTPATSVADAPEAADATPDATTPSLAAARVTAGAGVGVVLALASVWLGL
>LOC_Os09g30486
MEFKAAMFAAAVVAVLLSSPSPALAQKKSPPAAPSPVSLPPSLAPAPAPAPHYVDLAELLSVAGPFHTFLNY
LEKTNVIETFQSQANKTKEGVTIFVPKDSAFAAIKQSTFSNLTGDQLKTLLLYHAFPKFYSLAEFKNLSELNPV
NTFAGAPYTLNLTDDMGTISVQSMWSRPKISSSVYATRPVAVYALNKVLLPMQIFSKDPPLAPAPAPAPVS
GASDLAPGPASGKTGAGGGKADSTSAACGVGAGVVNGLVMALAGSLMLLW
>LOC_Os05g38500
MRGRQIPPPTAAASSRVVVAAVAALLLAAALQPLPVASGVDVAAVLAAFPDLAGFARLLASSPVARELAGR
SSLTLLAVPNGNLPQSPSAYAAASGADLADVLRYHVLLEYLAPADLRRLPASGKLVTTLFQTTGRAPADLGA
VNVTAAGPSLAVVRSPAPFPGSNATVLGAITAVPYNLSVLAVDGLIVPSGFDLAASESRPPAAVNITKVLAD
ARAFNVAASMLEASGVADEFEADERGAGITVFAPTDDAFAGLPAGDRLQSLPAERKAVVLRFHVLHSYYPL
GSLESIVNPVQPTLATEFTQAGRFTLNITRANGSVAIDTGVVQATITRTVFDQNPVAVFAVSKVLLPKEMFS
RSDSAIVAVASAPPPAALPAESPESAPTKLSSPPALRDTAGNANHTAAAAAVAAAAATTKPTIGRWCIALLYL
LLLPLLLPLV
>LOC_Os01g47780
MARSVAVVVLLAMAAVAAAQAPGPAATPAAGATGPPNVTAVLEKGGQYTTFIRLMKETQQDTQLNSQL
NNSFNGNGYTVFAPTDNAFNNLKPGTLNSLTQQQQVALVQGHVLPQFYSMDSFQTASNPVRTQASGTD

GPYTLNITSTTNNNVNVSTGVVEVTVTNALSAVKPLAVYSVDKVLLPFELFGVKAPAAAPTASTAKPKKGGS
TEAASGPAGAEDAEPTGAASARAVGWGVAGLAAVVGCLL
>LOC_Os04g39590
MRLAVATFLAIALSLSLLLAGALARPPPAPVRTDAGGGAAPAPQDKGGNLTDVLNVAGPFSTFLMYLRQTN
LVAVFEHQAYRTHQGITIFVPVDMAFAAIEPSVLAGLSRNQLKHLLMYHSLAKHYTLAEFDGLSQSNPVKT
LAGGRYAVNVTYDGGVVHVMSRWSSARVVGSVYESAAMAVYELDTVLLPDALFHAHPPVAATPPVPALP
SPPPHADHPPPDDDDGDIDDYVPAPPPDPAAGKGGAKRSASGPAVVAHKAASYGAAAAMTLLVSCL
>LOC_Os03g03600
MAAAAQRLVAAVVVLVACLALPAARGLNITAMLNGYPDYKMFNKYLSETKVCDEINARESITLLVLGDGP
MSTLVLDADQSLAGIKNALRLHAILDYFDPKKIRGLPDADTMTDTLYQAGGDAAGKMGNVKISTLDTGKI
AFASANPGGKYEATMVKAIKQMPYKLSIMEISAPIEFDGLFDTPSATNLTRLLEKAGCKRFASLITSTGVLKTF
EDAMDKGLTLFAPNDDAFDAKGAPDVKKMPSADLVTLLKYHALPSYNPKPTLKTVSRAMRTLASTASGKY
NITVDTRGDAVTLNTGVDKSRVAATVIDDTPVCVLTVDNLLMPVELFGDAPAAAPSPDDAAPAPSPSVAD
ASPPAPPPADAPSKPADHKEMKASSAVALRSVVLGALAAAVCSFVLL
>LOC_Os09g07350
MAAARLWVVAVAVAVAVAVMAAAVEGKSYNITKILAAHPEFSKFNEMLSKTRLAYDINRRQTITVLAVDN
SAMASLDHFTLPTIRHILSLHILVDYYGSKKLHALSHGATASSSMFQATGSAPGTTGYVNITSHKGGKIDFISE
DADESAKPSRYVKSVKEIPYDISVLQVSSVLSSSEAEAPVPPPAPVNLTELLSKKYCKSFAGLLAANADVFRAV
NETKDNGLTLFCPVDAAVAAFMPSYKNLTAKAKTAILLYHAVPDYFSLQLLKSNNGMVTTLATASESKKDYS
YDVQNKGETVTLDTRVVNSAVTATVGDAEPLAVYAVTKFLKPKELYKVVEAPAPAPEPSKKHKSAADDSSD
DSSDDSGDVKAHKGAAAPAPLARWVTAAAAAAVAALMLMA
>LOC_Os05g07060
MHVAKKSLASMAASATTTILVAAMLVVMAVESPVANGQAAAAAPAPAPAAPKTITAILTKAGQFTKFLQLL
QSTQAGEQINNQIKGKASSSGGLTVFAPPDNAFAALPTGTLNKLSDQQKTSLVQFHVVSALLPMAQFDTV
SNPLRTQAGETAAGKYPLNVTAEGSRVNISTGVVNATVDNTLYSGDRLVVYQVDKVLLPWALYGPPVPAPA
PSPADKAKKKAGPVGVADAPAADTAAGTTTTTVATASEAAARGTVRRGLVGVAVAVAVAWCGM
>LOC_Os08g23180
MELLLRRLAVVVAVVALTAATAAEGYNITKILGDHPEYSQFNKLLTETRLAGDINRRRTITVLVVANGDMGA
LSGGHYTLPTLRHILEMHILVDYYGAKKLHQLARGDTASSSMFQESGSAPGTTGYVNITQHRGGRVSFTAE
DAADSATPSSFVKSVKEIPYDLAVLQISKPLSSPEAEAPVAPPAPVNLTELLSKKYCKNFAGLLASNADVYSNI
NATKDNGLTLFCPVDAAVDAFLPKYKNLTAKGKAAILLYHAVPDYYSLQLLKSNSGKVSTLATASVAKKDYSY
DVSNDRDSVLLDTKVNSASVTATVKDADPLAVYAISKFLQPKELFKVTEDLAPAPAPEGPKKKTKKKKPSTTS
AAAAPSDDSSAADSPDGTPADDVADKAAAAPSVLARWVTAAATVAAALALAA
>LOC_Os05g48890
MAMVARRFLVAAAAVCLALAAVPAAMGQAAAPAPKGAAAAALNVTAILEKGGSYTTFIRLMKSTQQDT
QLNSQLNGTSTGFTVFAPTDGAFSSLKPGTLNSLSAQDQVSLVQAHIVPKFYSMDAFDTASNPVRTQASG
GDGPYTLNITATSTNQVNVSTGVVDTTLGTALRADQPLAVYSVDKVLLPYALFGPKPPPSPPPAPSKKPAKG
DTSASAEAPAGSADHPAGAAPAAARAAGWGVAALLAAACLL
>LOC_Os04g39600
MELKSAVLAVAALCLALALLLPRVALSQRLATADAPAPAPAPRHVDLAELLSLAGPYGTFLGYLTKTGVITTFQ
SQANDTAAGAPGVTVFAPEDSAFAAVGGGAALSNLTADQLRTLMLCHGVPRYHPLSSFSALAASGPAPTF
AGGQQYAVNVTDAAGTVRIQSGWATAKLVSSVYSTSPVAVYALNRVLLPEQIFPTAPKVAPVPAPAPAPVH
GDKANDGAPGAGELGASDVKSSSCRVGAGRLLAILAVMVSSFSDDIVV
>LOC_Os02g20560

MASSPSTVFLVLSLTLATVSVVVVSGAGEAPSPAPTGPLNLTEILTKAGHYNTFVRLLKDTEVTSQVSSLLNN
DRNGDGLTVLAPTDAAFGRLRPGTLNQMDAQAQAELVLYHVLPRYYGFVTFETTTNPVRTQASGQRGVC
TVNVTTAGEDRVRVSSGVVEAELGRPLRDGHPLAVYSLDAVLLPPDMFGPGAKKDYGAADAPAAAGKHG
KPQTASSSSVAAAPDEAPSKEVDATATAAAGRMAPAGWAAAFAGVVTAVVAVSLLSY

>LOC_Os02g20540
MASHTTLILLLPLLAAAAVAAADVAPAPAPPAAQTINLTGILEKGGQYNTLLRLLNATRVGEQLGSQLKTTYD
GLTFFAPTDAAFAALRPGTLNGLSDQEQVQLVLYHVLPRYYTLATFQTASNPLRTQATGPAGVYTVNVTTTT
GQSLVNVSTGVAAVPLGTTLSADFPLAVYSVDGVLLPEQMFGKAKAPAPAAAPASAAGKGANKEHKKGG
AMPKNEVAATAPTAGAGGEDSDDSATTNAVAVAGAAGVVWTAALVGIANLVIA

>LOC_Os09g30010
MRRLRLAAAAAAPHGVLLLLLILLPLVAAAGPAAAKAPAAPPAPPNVTTAMAKGGCKAFADLIAASPDASS
TYQSAAGGGITVFCPTDDAVRAFLPRYKNLTADGKAELLLFHAVPVYYSRGSLKSNNGVMNTLATDGAAK
NYNFTVQNEGDAVTIKTAASGDAARVKSTVVDADPVAIFTVDAVIEPVELFKPAPSPTPAPSPAPAADAPK
ASKPAHHPAPVVADAPGPAATDSPPADQKKEAKKSAAAGAPPCVRWFAAALAAVAMASTLA

>LOC_Os05g48900
MASSRLLLLAALLATAAVLAASQKPKAATPTKATPASPGPAAAAADGPAPTNVTAVLEKSGKYTTFLRLLHE
SRVDTQINSQLMDSYNGLTMFAPTDAAFAALKPGTLNSLSSQDQIQLMLYCVLPRFYSLAMLTTLGGPVN
TQASGADGPYKYKIKPSNNNVNISTGVNWALLSTVVSKDFPLAVYSVDKVPLPYELFGPKPPTPAPAPAPA
PSKSKTKKHKKSAGIAEPPVADDASADDTTKKAAAPATAVSRWVVAAAGVLAGAILAAL

>LOC_Os06g44660
MASRLSLLLLLALVLPASGDDAAAAEAAPPPAPAGGASFNVTEILGRFPEFGLFSYLISKTHVDRDINSRNTV
TVLVPDNSAVDWLLRRSARLPRAALVELLSVHVVLDYFDAAKIAALPPGKPTVSTTLFQTTGNARRRTGFLA
ITPTAKGGAVFASAAPGALVNATLKRVVAAVPYNISVLQISNFVVPPGVLTRPRPGPGPLPLPSPPLPRMKP
MAIAPTPAPVPAPTKMVPIPPSLPLTDPADEDGDEAPAAAPAPSHGNAVKVMSWWSGLGVLVGTMACV
FGYL

>LOC_Os02g26320
MACKNASSSAMPLLLVAGTVVLALLASPAAAFNITRILGEFSDFSTFNHLLTQTKLADEINRRQTITVLALDN
GAAGGVSSLPSDEQRKVLSVHVVLDYYDTEKLGGMKMKNRSAVLTTLFQSSGQATDRMGFLNYTKRSDG
IMVFGSAEPGAQATSQMVKVVVTRPYNISVLQVNSPIVPPGIASVSNSNTGAPPPHPAKSSAPAPSPSKSK
GKKSDAPAPGPSDDVDDDTAADAPGPAVDGPTADGPAADGPAADGPTEADAPAHDKGDVADAPSAAG
RAVASSAGLGVVALVVLIFPSVSL

>LOC_Os12g13160
MASFLAASLLIALVVSGSNVAATPSPSAVVGNGDIASTAQEMQRARYFTFVMLIRMVQEKIPHNTTFLMP
NDRMLSTATIPESQVMEFLSRHSIPAPLMFDDLIKLPNATIVPTAHSSQTITITNVKHQKIYFNNIELISPDVC
RVGDLFRCHGINGVIRPIVPRGKGSACPGHLAPATAAPGPASGANRSLETSSLTSPNMSSATSPSLQPAAES
PQSSDTSTSQIASSYTTLILVLVFSIF
